# Divergent medulloblastoma chromatin states disclose KDM2B as a selective dependency

**DOI:** 10.1038/s41588-026-02745-1

**Published:** 2026-09-24

**Authors:** Ran Tao, Serap Erkek-Ozhan, Beisi Xu, Yurika Matsui, Priya Mittal, Kyle S. Smith, Yiran Li, David Filipovic, Ruijie Xu, Qingsong Gao, Emily Darrow, Rahul Kumar, Nadhir Djekidel, Richa Bajpai, Jennifer Hadley, Melissa Batts, Sara A. Lewis, Taha Soliman, Colleen Reilly, Natarajan V. Bhanu, Leena Paul, Hong Lin, Brian Gudenas, Kim Lowe, Marc Zapatka, Laura Sieber, David T. W. Jones, Marcel Kool, Sebastian M. Waszak, Volker Hovestadt, Ivo Buchhalter, Marina Ryzhova, Andrey Korshunov, Peter Lichter, Lukas Chavez, Lena M. Kutscher, Benjamin A. Garcia, Shondra M. Pruett-Miller, Xin Zhou, Brent A. Orr, Giles W. Robinson, Gang Wu, Jan O. Korbel, Jamy C. Peng, Stefan M. Pfister, Paul A. Northcott

**Affiliations:** 1 Center of Excellence in Neuro-Oncology Sciences, St. Jude Children’s Research Hospital, Memphis, TN USA; 2 Department of Developmental Neurobiology, St. Jude Children’s Research Hospital, Memphis, TN USA; 3 Hopp Children´s Cancer Center Heidelberg (KiTZ), Heidelberg, Germany; 4 Division of Pediatric Neurooncology, German Cancer Research Center (DKFZ) and German Cancer Consortium (DKTK), Heidelberg, Germany; 5 Department of Pediatric Hematology and Oncology, Heidelberg University Hospital and National Center for Tumor Diseases (NCT), Heidelberg, Germany; 6 Izmir Biomedicine and Genome Center, Inciralti, Izmir, Turkey; 7 Genome Biology Unit, European Molecular Biology Laboratory (EMBL), Heidelberg, Germany; 8 Center for Applied Bioinformatics, St. Jude Children’s Research Hospital, Memphis, TN USA; 9 Department of Pathology, St. Jude Children’s Research Hospital, Memphis, TN USA; 10 Department of Neurologic Surgery, Mayo Clinic, Rochester, MN USA; 11 Department of Cell & Molecular Biology, St. Jude Children’s Research Hospital, Memphis, TN USA; 12 Center for Advanced Genome Engineering, St. Jude Children’s Research Hospital, Memphis, TN USA; 13 Department of Computational Biology, St. Jude Children’s Research Hospital, Memphis, TN USA; 14 Department of Biochemistry and Molecular Biophysics, Washington University School of Medicine, St. Louis, MO USA; 15 Division of Molecular Genetics, German Cancer Research Center, Heidelberg, Germany; 16 JRG Developmental Origins of Pediatric Cancers, German Cancer Research Center (DKFZ), Heidelberg, Germany; 17 Division of Pediatric Glioma Research (B360), German Cancer Research Center (DKFZ), Heidelberg, Germany; 18 National Center for Tumor Diseases (NCT), NCT Heidelberg, a partnership between DKFZ and Heidelberg University Hospital, Heidelberg, Germany; 19 Princess Máxima Center for Pediatric Oncology, Utrecht, the Netherlands; 20 University Medical Center Utrecht, Utrecht, the Netherlands; 21 Department of Pediatric Oncology, Dana-Farber Cancer Institute, Boston, MA USA; 22 Broad Institute of MIT and Harvard, Cambridge, MA USA; 23 Omics IT and Data Management Core Facility, German Cancer Research Center (DKFZ), Heidelberg, Germany; 24 Department of Neuropathology, Burdenko Neurosurgical Institute, Moscow, Russia; 25 Clinical Cooperation Unit Neuropathology, German Cancer Research Center (DKFZ), German Cancer Consortium (DKTK), and National Center for Tumor Diseases (NCT), Heidelberg, Germany; 26 Department of Neuropathology, Heidelberg University Hospital, Heidelberg, Germany; 27 Department of Medicine, University of California San Diego, San Diego, CA USA; 28 Sanford Burnham Prebys Medical Discovery Institute, San Diego, CA USA; 29 Rady Children’s Institute for Genomic Medicine, Rady Children’s Hospital and Healthcare Center, San Diego, CA USA; 30 Department of Oncology, St. Jude Children’s Research Hospital, Memphis, TN USA; 31 Bridging Research Division on Mechanisms of Genomic Variation and Data Science, German Cancer Research Center (DKFZ), Heidelberg, Germany

**Keywords:** CNS cancer, Epigenomics, Epigenomics, Epigenetics, Embryonal neoplasms

## Abstract

Medulloblastoma is a biologically heterogeneous childhood cerebellar tumor harboring frequent chromatin-modifying gene alterations. How these alterations promote transcriptional programs governing malignancy remains poorly defined. To address this knowledge gap, we evaluated chromatin states across medulloblastoma subgroups by multi-modal integration of histone modifications with mutational, DNA methylation and transcriptomic profiles. A bivalent/poised enhancer (EnhBiv) state was specifically enriched at the promoters of neurodevelopmental genes in Group 3/4 medulloblastoma. Integrative bioinformatics coupled with chromatin occupancy studies identified aberrant KDM2B binding at EnhBiv-enriched promoters. CRISPR-mediated knockout or acute protein degradation of KDM2B selectively suppressed the growth of medulloblastoma models in vitro and in vivo. Mechanistically, KDM2B promotes sequential recruitment of Polycomb repressive complexes (PRC1/PRC2) and EnhBiv chromatin, thereby repressing neuronal differentiation programs. Collectively, we provide foundational insights into an epigenetic basis of medulloblastoma, nominating KDM2B as a selective dependency in high-risk subgroups that warrants consideration as a candidate therapeutic target.

## Abstract (web summary)

Epigenomic analyses of medulloblastoma identify a bivalent/poised enhancer (EnhBiv) chromatin signature enriched for KDM2B binding in Group 3 and Group 4 tumors. Functional work suggests a selective dependency of these subtypes on KDM2B.

## Main

Medulloblastoma is classified by the World Health Organization as a grade 4 embryonal tumor of the cerebellum and comprises four principal molecular subgroups: WNT, SHH, Group 3 and Group 4 (refs. ^1–4^). Each subgroup can be further divided into distinct subtypes that exhibit diverse demographics and clinical outcomes^5–7^. Molecular subgroups and subtypes of medulloblastoma contain mostly discriminatory transcriptional and epigenetic landscapes^6–9^, which are suspected to arise from their distinct cellular origins in the developing hindbrain. During human cerebellar development, the rhombic lip germinal zone generates all glutamatergic neurons, including granule neurons and unipolar brush cells (UBCs), the presumed lineages of origin for SHH and Group 3/4 medulloblastomas, respectively^10,11^. Acquisition of genetic and/or epigenetic alterations in mitotic progenitors that stall neuronal differentiation is believed to be fundamental to medulloblastoma pathogenesis^12^, despite rudimentary understanding of its mechanism.

Somatic mutations in genes encoding chromatin modulators are widespread across cancer entities, implicating deregulation of the epigenome as a pivotal event during cellular transformation and malignant progression. Chromatin modifiers, including chromatin remodelers and histone readers, writers and erasers, are among the most frequently mutated class of genes in cancer^13,14^. This is also true for medulloblastoma, in which somatic mutations and focal copy-number alterations affecting chromatin-modifying genes are observed in an estimated 50% of all cases^6,15–17^, and often in isolation, consistent with driver events. Despite the prevalence of these alterations, how aberrant activity of chromatin modifiers shapes subgroup-specific chromatin landscapes and promotes tumorigenesis is largely unknown.

Here, we report the chromatin landscape of medulloblastoma through the lens of histone post-translational modifications (PTMs) and discover enrichment of an enhancer bivalent (EnhBiv) state in Group 3/4, which was correlated with histone lysine demethylase 2B (KDM2B) overexpression and occupancy at the same bivalent/poised promoters. Through integrated molecular and functional studies, we resolve the mechanistic basis by which KDM2B-dependent Polycomb deposition drives the EnhBiv state and suppresses neuronal differentiation, substantiating the KDM2B–PRC1.1–PRC2 axis as a Group 3/4 medulloblastoma-selective dependency warranting therapeutic targeting.

## Results

### The chromatin landscape of medulloblastoma subgroups

**Fig. 1:**
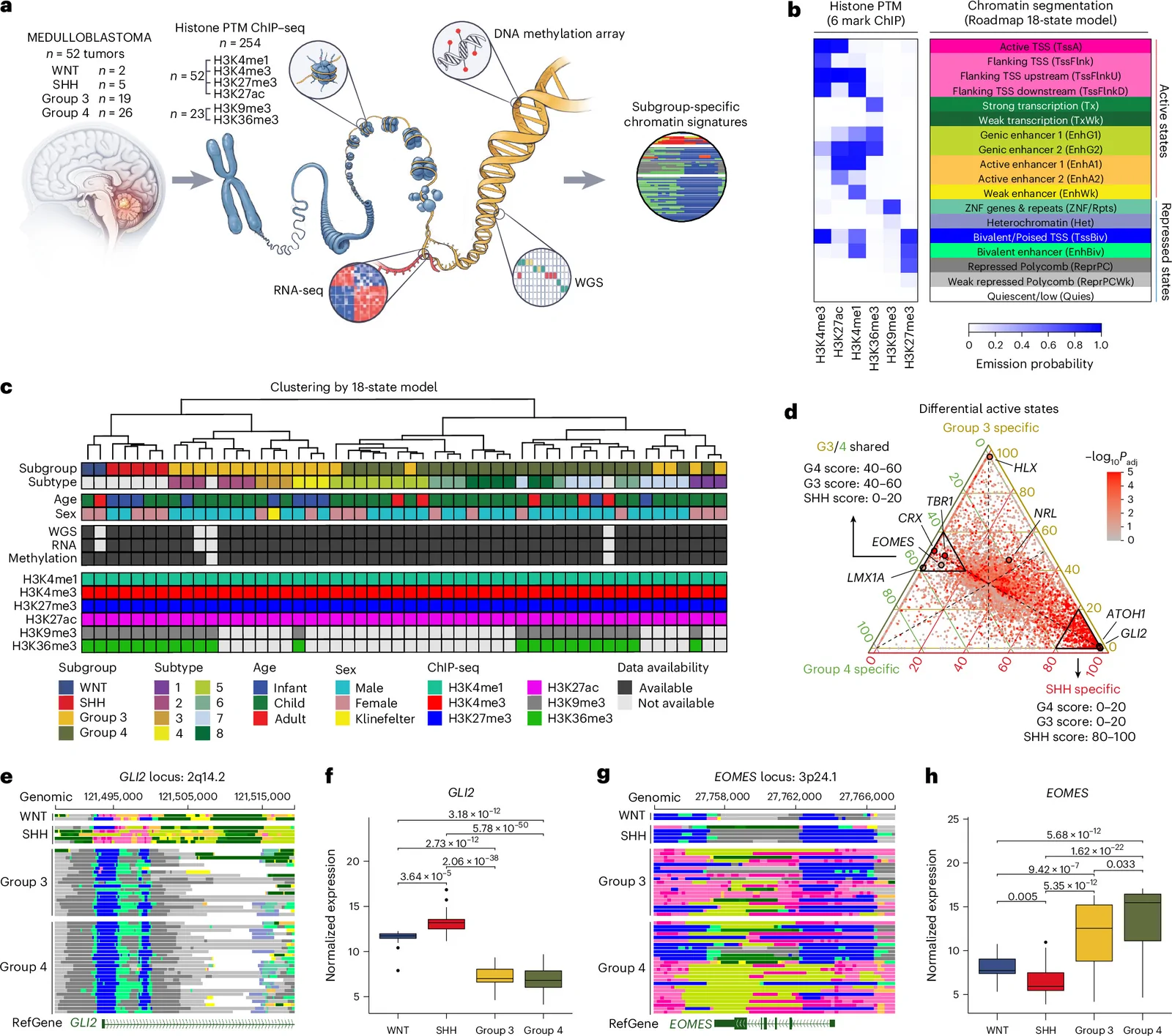
Characterization of the medulloblastoma chromatin landscape. **a**, Schematic overview of the medulloblastoma chromatin state cohort and complementary multi-omic datasets. **b**, Roadmap 18-state chromatin segmentation model applied to medulloblastoma; 11 active states and seven repressive states are shown. **c**, Clustering medulloblastoma patient samples by the 18-state model recapitulated subgroup identity. Essential metadata and available molecular data for each tumor sample are annotated. **d**, Differential enrichment of active chromatin states at promoters (*n* = 18,741) comparing SHH, Group 3 and Group 4. Red, yellow and green lines indicate relative enrichment scores of active states for SHH, Group 3 and Group 4, respectively. Two black triangles indicate the enrichment score thresholds for determining SHH-specific or Group 3/4-shared active states. Representative medulloblastoma signature genes are labeled. Dot color represents statistical significance of subgroup specificity by one-way ANOVA. **e**, Chromatin states at the *GLI2* locus in medulloblastoma subgroups. **f,** *GLI2* expression in medulloblastoma subgroups. **g**, Chromatin states at the *EOMES* locus in medulloblastoma subgroups. **h,** *EOMES* expression in medulloblastoma subgroups. For **e** and **g**, Colors depict chromatin states for each sample using the 18-state model shown in **b**. WNT, *n* = 2; SHH, *n* = 5; Group 3, *n* = 19; Group 4, *n* = 26 samples. For **f** and **h**, boxes represent the interquartile range (IQR; 25th–75th percentiles), the center line indicates the median, whiskers extend to the most extreme data points within 1.5× the IQR, and dots indicate outliers beyond 1.5× the IQR. WNT, *n* = 15; SHH, *n* = 46; Group 3, *n* = 41; Group 4, *n* = 65 samples. Statistical significance was assessed using the two-sided *t*-test, with *P* values adjusted by the Holm method. *P*_adj_ values are shown for significant comparisons. WGS, whole-genome sequencing.

**Extended Data Fig. 1:**
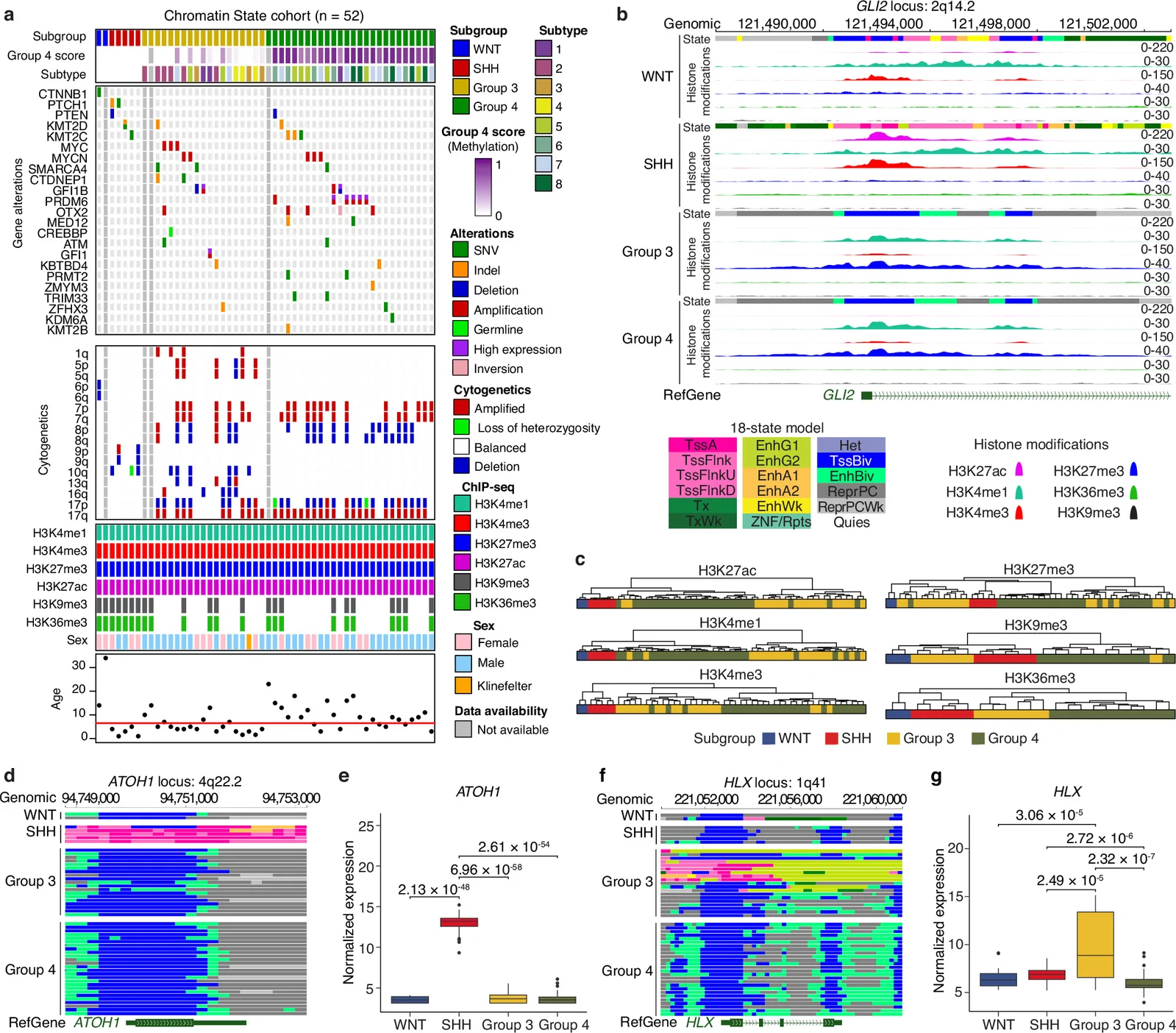
Chromatin state profiling in medulloblastoma subgroups. **a**, Oncoprint summarizing somatic alterations, cytogenetics, patient age and sex, and data availability of the chromatin state cohort. Red line indicates median age. **b**, Merged histone tracks and aggregated chromatin state at the *GLI2* locus in medulloblastoma subgroups demonstrating active chromatin in SHH-medulloblastoma and repressive chromatin in Group 3- and Group 4-medulloblastoma. WNT, n = 2; SHH, n = 5; Group 3, n = 19; Group 4, n = 26 samples. **c**, Clustering medulloblastoma patient samples by individual histone marks predicted subgroup identity. **d**, Chromatin state at the *ATOH1* locus in medulloblastoma subgroups. **e**, *ATOH1* expression in medulloblastoma subgroups. **f**, Chromatin state at the *HLX* locus in medulloblastoma subgroups. **g**, *HLX* expression in medulloblastoma subgroups. For **d** and **f**, colors depict chromatin states for each sample using 18-state model shown in **b**. WNT, n = 2; SHH, n = 5; Group 3, n = 19; Group 4, n = 26 samples. For **e** and **g**, Boxes represent the interquartile range (IQR; 25th–75th percentiles), center line indicates the median, and whiskers extend to the most extreme data points within 1.5 × IQR. Dots indicate outliers beyond 1.5 × IQR. WNT, n = 15; SHH, n = 46; Group 3, n = 41; Group 4, n = 65 samples. Statistical significance was assessed using the two-sided t-test, with *P* adjusted by the Holm method. *P*_adj_ values are shown on the plots for significant comparisons.

Chromatin states are a functional readout of combinatorial PTMs of histone tails. To decipher chromatin states underlying the medulloblastoma subgroups, we profiled a core set of six histone PTMs through chromatin immunoprecipitation coupled with sequencing (ChIP–seq): H3 lysine 4 monomethylation (H3K4me1; enhancer mark); H3 lysine 4 trimethylation (H3K4me3; promoter mark); H3 lysine 27 trimethylation (H3K27me3; Polycomb mark); H3 lysine 27 acetylation (H3K27ac; enhancer/promoter mark); H3 lysine 9 trimethylation (H3K9me3; heterochromatin mark); and H3 lysine 36 trimethylation (H3K36me3; transcription mark)^18^. Using 52 fresh–frozen tumor specimens representing each of the consensus medulloblastoma subgroups (WNT, *n* = 2; SHH, *n* = 5; Group 3, *n* = 19; Group 4, *n* = 26), 254 histone PTM ChIP–seq experiments were performed (H3K4me1, H3K4me3, H3K27me3 and H3K27ac, *n* = 52 tumors per mark; H3K9me3 and H3K36me3, *n* = 23 tumors per mark), of which 229 ChIP–seq profiles were newly generated as part of this study (Supplementary Table 1). Histone ChIP–seq profiles were integrated with mutational (*n* = 223)^6^, DNA methylation (*n* = 223)^6^ and RNA sequencing (RNA-seq; *n* = 167)^6,8^ datasets, which include most samples in the ChIP–seq cohort (Fig. 1a, Extended Data Fig. 1a and Supplementary Table 2). Histone modification tracks accurately recapitulate active chromatin in SHH medulloblastoma and repressed chromatin in Groups 3 and 4 medulloblastoma at the *GLI2* locus, a SHH medulloblastoma signature gene (Extended Data Fig. 1b), which is a testament to the integrity of the data.

To facilitate combinatorial integration of individual histone marks, we implemented ChromHMM, which is based on a multivariate hidden Markov model^18–20^, and used the Roadmap 18-state segmentation model^20^ to annotate genome-wide chromatin states (Fig. 1b). Clustering tumor samples by chromatin states or individual histone marks largely discriminated medulloblastoma subgroups (Fig. 1c and Extended Data Fig. 1c). To examine whether active chromatin states could accurately predict known medulloblastoma subgroup-specific signature genes^21^, we performed differential enrichment analysis for the combined 11 active states (Fig. 1b) across SHH, Group 3 and Group 4 (excluding WNT because of the small sample size). This analysis uncovered genes that were either subgroup-specific or shared by two subgroups, including well-known cerebellar lineage transcription factors and medulloblastoma drivers (Fig. 1d and Supplementary Table 3). For example, ATOH1 and GLI2 are markers of proliferating granule neuron progenitors, the cell of origin for SHH medulloblastoma^10,11,22–29^. Both *ATOH1* and *GLI2* loci were marked by active chromatin specifically in SHH medulloblastoma, concordant with their high expression in this subgroup (Fig. 1d–f and Extended Data Fig. 1d,e). HLX is a master transcription factor regulated by a prominent super-enhancer in Group 3 medulloblastoma^8^. The *HLX* promoter was marked by active chromatin states and highly expressed specifically in Group 3 medulloblastoma (Fig. 1d and Extended Data Fig. 1f,g). EOMES is actively expressed in UBC-fated progenitors of the rhombic lip subventricular zone, the developmental precursor for Group 3 and 4 medulloblastomas^10,11^. *EOMES* was highly expressed and marked by active chromatin in both Group 3 and 4 (Fig. 1d,g,h). Interestingly, although many signature genes were marked by SHH-medulloblastoma-specific active states, Group 3 and 4 tumors exhibited a higher proportion of signature genes that shared active chromatin states (Fig. 1d). These results are consistent with Group 3 and 4 tumors sharing a unified developmental origin in the rhombic lip subventricular zone^10^. Together, histone modifications and their aggregated chromatin states are highly concordant with known medulloblastoma subgroup-specific transcriptional signatures, suggesting that subgroup identity is epigenetically encoded.

### Enrichment of a bivalent enhancer state in Groups 3 and 4

**Fig. 2:**
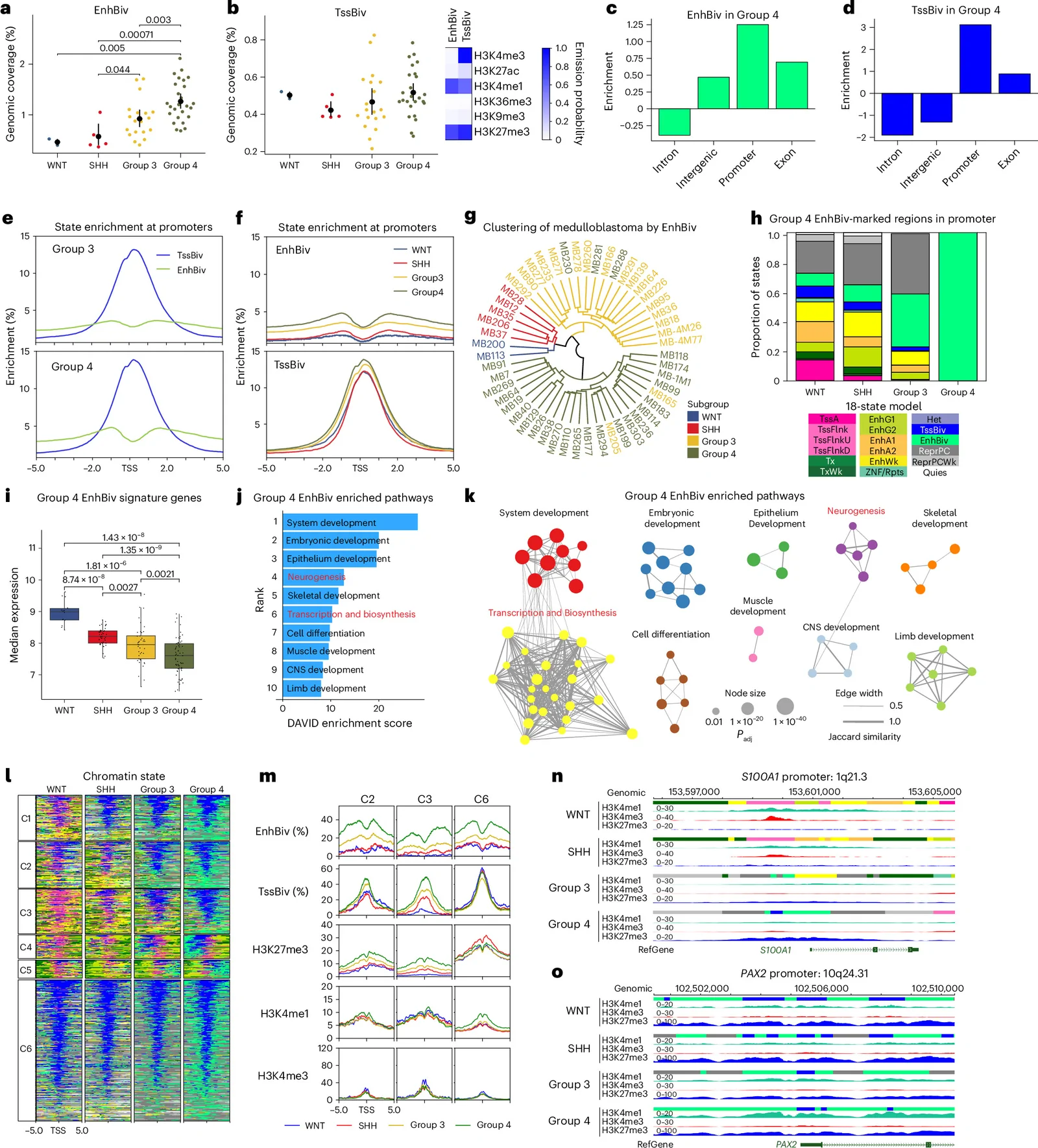
Enrichment of an EnhBiv state associated with transcriptional repression in Group 3 and 4 medulloblastomas. **a**,**b**, Genomic coverage of the EnhBiv state (**a**) and TssBiv state (**b**) among medulloblastoma subgroups. Black dots indicate the median, and bars indicate 1.5× the IQR. WNT, *n* = 2; SHH, *n* = 5; Group 3, *n* = 19; Group 4, *n* = 26 samples. *P* values, determined by the two-sided Mann–Whitney *U*-test, are shown on the plots for significant comparisons. **c**,**d**, Genomic enrichment of the EnhBiv (**c**) and TssBiv (**d**) states in Group 4 medulloblastoma. *n* = 50,945 regions for EnhBiv and *n* = 51,710 for TssBiv. **e**, Distribution of EnhBiv and TssBiv states in the TSS ± 5 kb promoter window of protein-coding genes (*n* = 21,284) in Group 3 (top) and Group 4 (bottom) medulloblastoma. **f**, Enrichment of EnhBiv and TssBiv states in the TSS ± 5 kb promoter window of protein-coding genes (*n* = 21,284) comparing medulloblastoma subgroups. **g**, Clustering samples from patients with medulloblastoma by the EnhBiv state accurately predicted subgroup identity. **h**, Chromatin state proportions of Group 4 EnhBiv-marked promoter regions (*n* = 5,420) in medulloblastoma subgroups. **i**, Expression of Group 4 EnhBiv signature genes (*n* = 666) in medulloblastoma subgroups. Boxes represent the IQR, center line indicates the median, whiskers extend to the most extreme data points within 1.5× the IQR, and dots represent samples (WNT, *n* = 15; SHH, *n* = 46; Group 3, *n* = 41; Group 4, *n* = 65). Statistical significance was assessed using a two-sided Wilcoxon test. *P* values are shown on the plot for significant comparisons. **j**, Top ten enriched pathways of Group 4 EnhBiv signature genes (*n* = 666) from DAVID analysis. Enrichment score represents the geometric mean of EASE scores (modified Fisher’s exact test with Benjamini–Hochberg correction) for all Gene Ontology biological process terms within each annotation cluster. **k**, Visualization of **j**. Nodes represent Gene Ontology terms. Edge width represents Jaccard similarity. Node size indicates significance determined by modified Fisher’s exact test (EASE score) with Benjamini–Hochberg correction. Neurogenesis-related and transcription-related terms are highlighted in red. **l**, Chromatin state in six clusters (C1–C6) of Group 4 EnhBiv signature gene promoters (*n* = 666) in medulloblastoma subgroups. Colors depict chromatin states using the 18-state model shown in **h**. **m**, Profile plots showing enrichment of EnhBiv and TssBiv states or peak intensity for H3K27me3, H3K4me1 and H3K4me3 in C2, C3 and C6 across medulloblastoma subgroups. **n**,**o**, Merged chromatin state and histone marks at representative promoters from C3 (*S100A1*; **n**) and C6 (*PAX2*; **o**) by medulloblastoma subgroup; states colored by the 18-state model in **h**.

**Extended Data Fig. 2:**
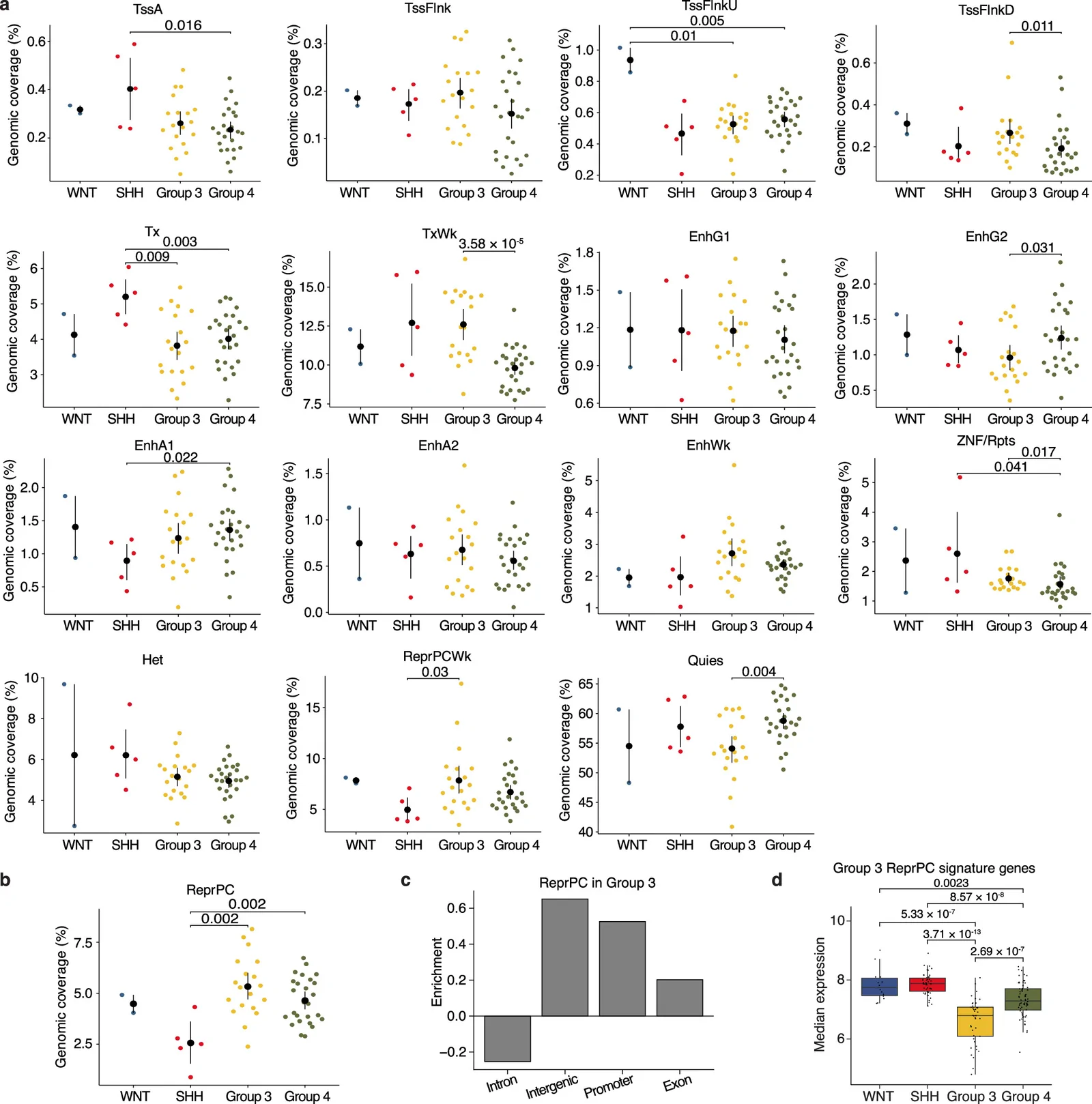
Chromatin state enrichment in medulloblastoma subgroups. **a**, Genomic coverage of chromatin states among medulloblastoma subgroups. **b**, Genomic coverage of Repressed Polycomb (ReprPC) state in medulloblastoma subgroups. For **a** and **b**, Black dots indicate median and bars indicate 1.5 × IQR. WNT, n = 2; SHH, n = 5; Group 3, n = 19; Group 4, n = 26 samples. *P* values determined by the two-sided Mann-Whitney U-test are shown for significant comparisons. **c**, Genomic enrichment of ReprPC state (n = 111,048 regions) in Group 3-medulloblastoma. **d**, Expression of Group 3 ReprPC signature genes (n = 382) in medulloblastoma subgroups. Boxes represent the IQR, center line indicates the median, and whiskers extend to the most extreme data points within 1.5 × IQR. Dots represent samples. WNT, n = 15; SHH, n = 46; Group 3, n = 41; Group 4, n = 65 samples. Statistical significance was assessed using the two-sided Wilcoxon test. *P* values are shown on the plot for significant comparisons.

To investigate how divergent chromatin states may drive medulloblastoma subgroup-specific tumorigenesis, we examined whether chromatin states differed among subgroups (Fig. 2a,b and Extended Data Fig. 2a,b). Notably, the coverage of the active transcription start site (TSS) state (TssA, consisting of H3K4me3 and H3K27ac) was highest in SHH medulloblastoma and underrepresented in Group 4 (Extended Data Fig. 2a). Consistent with previous reports^30^, the repressed Polycomb state (ReprPC; marked by H3K27me3) was highly represented in Group 3 compared to other subgroups and was enriched in intergenic regions (Extended Data Fig. 2b,c). Gene promoters marked by the ReprPC state in Group 3 (Supplementary Table 4) were associated with reduced expression, consistent with a repressive state (Extended Data Fig. 2d).

**Extended Data Fig. 3:**
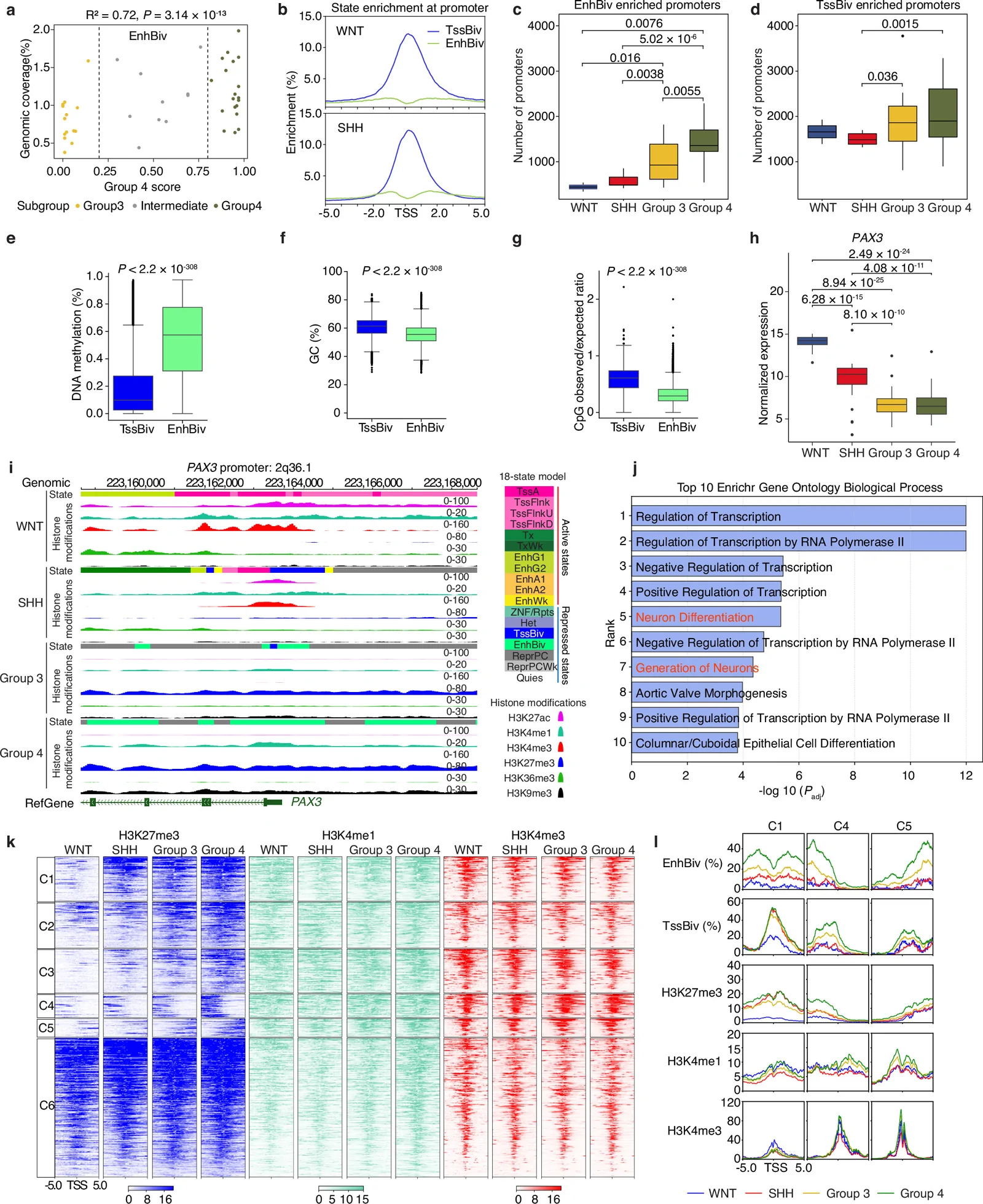
EnhBiv and TssBiv enrichment in medulloblastoma subgroups. **a**, Scatterplot showing bivalent enhancer (EnhBiv) genomic coverage along Group 3 to Group 4 differentiation continuum. Group 3/4 medulloblastoma (n = 43) were ranked by Group 4 score (differentiation score) from left to right. Dashed line indicates cutoff for calling Group 3 (< 0.2), Group 4 (> 0.8), or intermediate (between 0.2 - 0.8). EnhBiv genomic coverage was positively correlated with differentiation score as determined by linear regression analysis. **b**, Distribution of EnhBiv and bivalent transcription start site (TssBiv) states at the transcriptional start site (TSS) ± 5kb promoter window of protein coding genes (n = 21,284) in WNT and SHH medulloblastoma. **c** and **d**, Box plot depicting the number of EnhBiv (**c**) and TssBiv (**d**) enriched promoters in medulloblastoma patient samples. WNT, n = 2; SHH, n = 5; Group 3, n = 19; Group 4, n = 26 samples. *P* values determined by the two-tailed Wilcoxon test are shown for significant comparisons. **e**-**g**, DNA methylation (**e**), GC content (**f**), or CpG observed/expected ratio (**g**) at promoter-enriched TssBiv (n = 8,616) and EnhBiv (n = 11,112)-marked regions in Group 4-medulloblastoma. Statistical significance was determined by the two-sided Mann-Whitney U test. *P* values smaller than 2.2 × 10^−308^ were capped due to numerical underflow. **h**, *PAX3* expression in medulloblastoma subgroups. WNT, n = 15; SHH, n = 46; Group 3, n = 41; Group 4, n = 65 samples. Statistical significance was assessed using the two-sided t-test, with *P* adjusted by the Holm method. *P*_adj_ values are shown for significant comparisons. For **c**-**h**, Boxes represent the IQR, center line indicates the median, and whiskers extend to the most extreme data points within 1.5 × IQR. For **e**-**h**, Dots indicate outliers beyond 1.5 × IQR. **i**, Merged histone tracks and aggregated chromatin state at the *PAX3* promoter in medulloblastoma subgroups. WNT, n = 2; SHH, n = 5; Group 3, n = 19; Group 4, n = 26 samples. **j**, Top 10 enriched pathways of Group 4 EnhBiv signature genes (n = 666) from Enrichr. Neurogenesis-related terms are highlighted in red. *P* values were calculated using the two-sided Fisher’s exact test and adjusted using the Benjamini-Hochberg false discovery rate (FDR) correction. **k**, Heatmap showing histone marks at six clusters (C1-C6) of Group 4 EnhBiv signature gene promoters (n = 666) in medulloblastoma subgroups. **l**, Profile plots showing enrichment of EnhBiv and TssBiv state or peak intensity for H3K27me3, H3K4me1, and H3K4me3 in C1, C4 and C5 comparing medulloblastoma subgroups.

The EnhBiv state, consisting of H3K4me1 and H3K27me3, marked regulatory elements poised for activation^31,32^ and showed the most striking difference among medulloblastoma subgroups. Genomic coverage of the EnhBiv state was highest in Group 4, followed by Group 3, and positively correlated with Group 3/4 differentiation score; WNT and SHH medulloblastomas exhibited the lowest EnhBiv coverage (Fig. 2a and Extended Data Fig. 3a). By contrast, the promoter poised bivalent transcription start site (TssBiv) state, associated with overlapping H3K4me1, H3K4me3 and H3K27me3, showed comparable genomic coverage across subgroups (Fig. 2b). Both EnhBiv and TssBiv overlapped promoter-proximal regions defined by TSS ± 5 kb (Fig. 2c,d), with TssBiv sharply enriched at the immediate TSS (±2 kb) and EnhBiv in regions flanking the TSS (Fig. 2e and Extended Data Fig. 3b). Consistent with a high genomic coverage in Groups 3 and 4, EnhBiv, but not TssBiv, was enriched at promoters in these subgroups compared to WNT and SHH (Fig. 2f and Extended Data Fig. 3c,d). We also observed higher DNA methylation, lower GC content and lower CpG densities in the EnhBiv state-marked regions compared to the TssBiv state-marked regions within TSS ± 5 kb (Extended Data Fig. 3e–g). These findings suggest that although EnhBiv overlaps promoter-proximal regions, its spatial distribution, histone mark composition and genomic and epigenomic features distinguish it from classical poised promoters. To capture this promoter-flanking EnhBiv state, we defined promoters as TSS ± 5 kb in this study, encompassing both canonical promoters and nearby regulatory elements.

Distribution of the EnhBiv state across 200 bp genomic windows robustly discriminated medulloblastoma subgroups (Fig. 2g). Consistently, Group 4 EnhBiv-marked regions in promoters exhibited distinct chromatin states in other medulloblastoma subgroups, with ~40% marked by the ReprPC state in Group 3 (Fig. 2h). *PAX3*, a transcription factor regulating neurogenesis^33^, was repressed in both Groups 3 and 4 compared with WNT and SHH (Extended Data Fig. 3h). The *PAX3* promoter was predominantly occupied by the EnhBiv state in Group 4 but the ReprPC state in Group 3, indicating context-dependent repression mechanisms (Extended Data Fig. 3i).

We identified 666 gene promoters significantly enriched for the EnhBiv state in Group 4 compared to other subgroups, which we designated as ‘Group 4 EnhBiv signature’ genes (Supplementary Table 5). Expression of these genes was lowest in Group 4 compared to other subgroups (Fig. 2i), in agreement with EnhBiv marking a repressive state. Group 4 EnhBiv signature genes were enriched in gene sets associated with neurogenesis and transcription (Fig. 2j,k, Extended Data Fig. 3j and Supplementary Tables 5 and 6), consistent with the established role of bivalency during development. Clustering Group 4 EnhBiv signature genes based on their promoter-associated chromatin state resulted in six clusters (Fig. 2l). Of the three histone marks underlying the EnhBiv state, H3K27me3 was higher in C2 and C3 for Groups 3 and 4 compared to WNT and SHH, whereas H3K4me1 was elevated in Group 4 in C6 (Fig. 2m and Extended Data Fig. 3k). H3K4me3 levels were comparable among subgroups in each cluster (Fig. 2m and Extended Data Fig. 3l). These cluster-level observations are recapitulated at the promoters of neurogenesis genes *S100A1* (C3) and *PAX2* (C6) (Fig. 2n,o and Supplementary Table 5). These results indicate that both H3K4me1 and H3K27me3 contribute to the enriched EnhBiv state in Groups 3 and 4 and are locus-dependent.

### Aberrant KDM2B binding at Group 3/4 EnhBiv-marked promoters

**Fig. 3:**
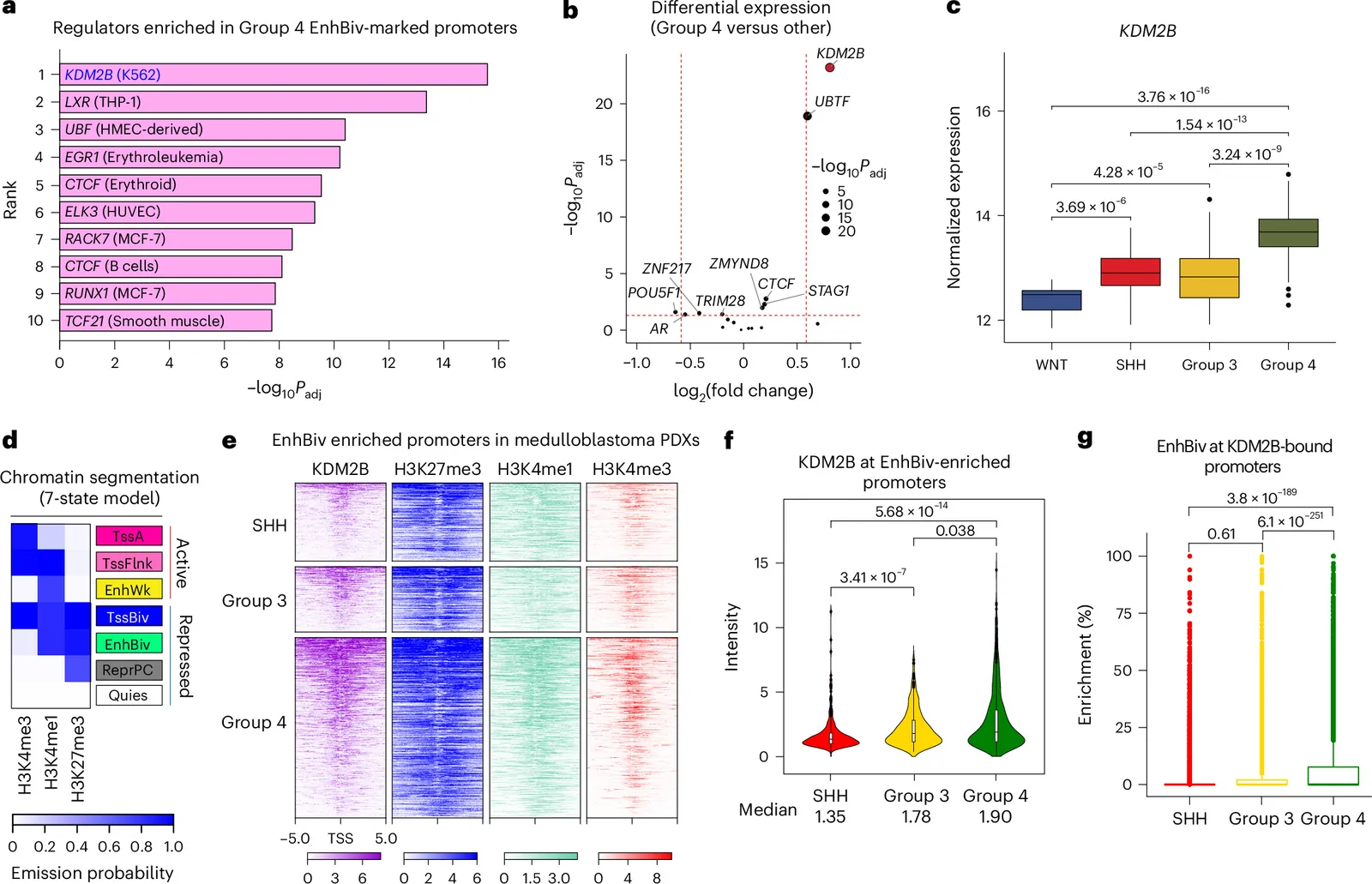
Aberrant KDM2B occupancy at Group 3 and Group 4 EnhBiv-enriched promoters. **a**, Top ten enriched regulators at Group 4 EnhBiv signature gene promoters (*n* = 666) identified by GSEA using the ChEA gene set. *P* value was determined by the two-sided hypergeometric test with FDR correction. **b**, Differential expression of regulators enriched in the six clusters of Group 4 EnhBiv signature gene promoters, comparing Group 4 with other medulloblastoma subgroups. Dot size indicates statistical significance determined by the two-sided Wald test (DESeq2) with Benjamini–Hochberg correction. **c,** *KDM2B* expression in medulloblastoma subgroups. Statistical significance was assessed using a two-sided *t*-test and adjusted by the Holm method. *P*_adj_ values are shown on the plot for significant comparisons. For **b** and **c**, WNT, *n* = 15; SHH, *n* = 46; Group 3, *n* = 41; Group 4, *n* = 65 samples. **d**, Seven-state chromatin segmentation model trained with H3K27me3, H3K4me1 and H3K4me3 marks using patient ChIP–seq data. **e**, Heatmap shows enrichment of KDM2B and indicated marks at EnhBiv-enriched promoters in medulloblastoma PDXs. **f**, Violin plot quantifying KDM2B peak intensity at EnhBiv-enriched promoters in medulloblastoma PDXs. Violin shows kernel probability density estimation. *P* values determined by two-tailed Wilcoxon test are shown on the plot. For **e** and **f**, SHH, *n* = 313; Group 3, *n* = 262; Group 4, *n* = 723 promoters. **g**, Box plot showing enrichment of the EnhBiv state at KDM2B-bound promoters in medulloblastoma PDXs. Only promoters that are marked by the EnhBiv state are included in the analysis: SHH, *n* = 6,574; Group3, *n* = 7,388; Group 4, *n* = 8,228 promoters. *P* values determined by two-sided *t*-tests are shown on the plot. For **c**, **f** and **g**, boxes represent the IQR, the center line indicates the median, whiskers extend to the most extreme data points within 1.5× the IQR, and dots indicate outliers beyond 1.5× the IQR.

**Extended Data Fig. 4:**
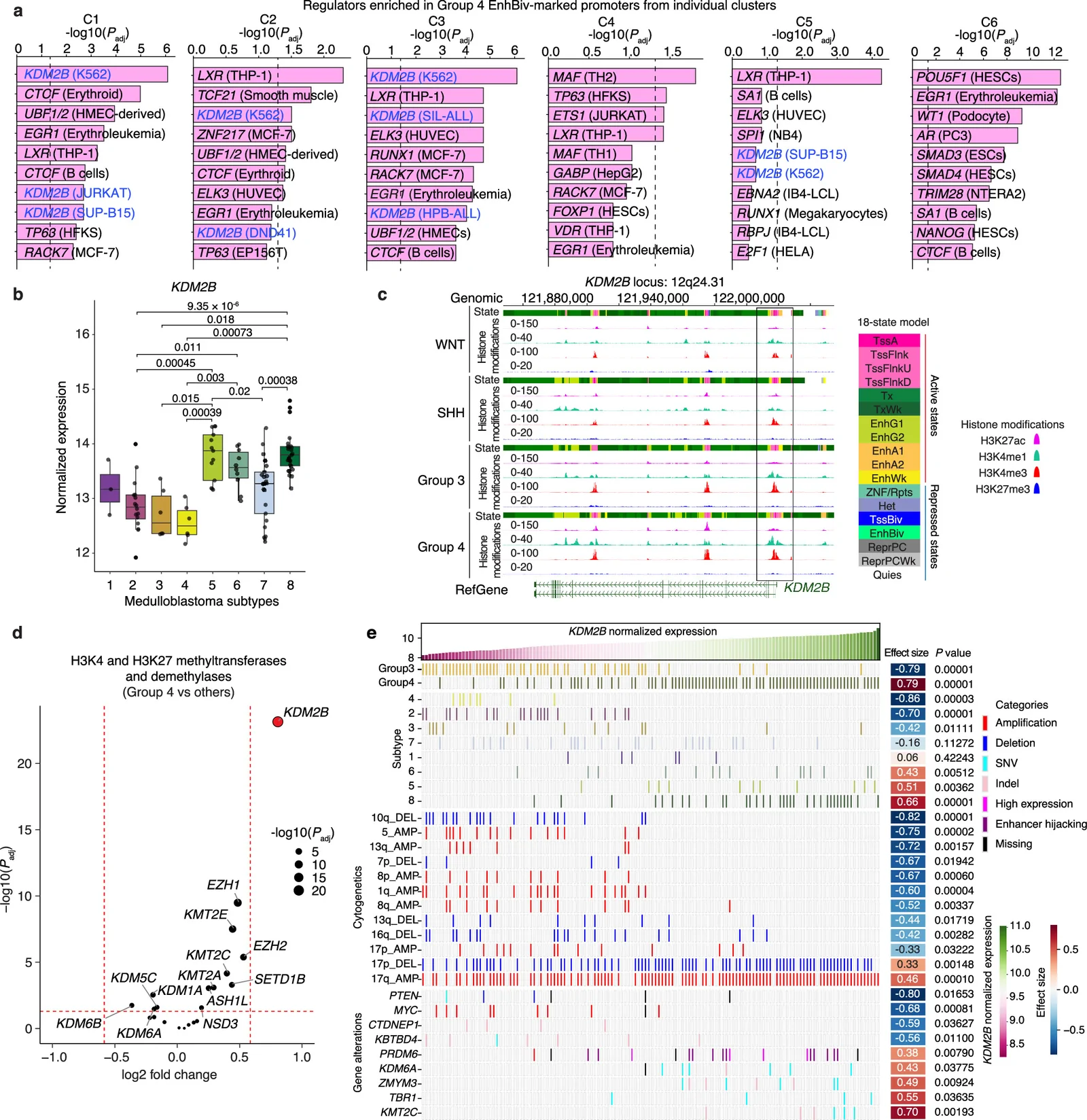
KDM2B is highly expressed in Group 4 and is enriched in the promoter of Group 4 EnhBiv signature genes. **a**, Top 10 regulators identified by gene set enrichment analysis (GSEA) using the ChEA gene set for individual clusters of Group 4 EnhBiv signature gene promoters (n = 666). *P* values were determined by the two-sided hypergeometric test with FDR correction. Dashed lines indicate the significance threshold (*P*_adj_ < 0.05). **b**, *KDM2B* expression in Group 3- and 4-subtypes (1, n = 3; 2, n = 16; 3, n = 6; 4, n = 6; 5, n = 13; 6, n = 10; 7, n = 25; 8, n = 27 samples). Boxes represent the IQR, center line indicates the median, and whiskers extend to the most extreme data points within 1.5 × IQR. *P*_adj_ values are determined by two-sided t-test and adjusted by the Holm method. **c**, Histone tracks and chromatin state at the *KDM2B* locus in medulloblastoma subgroups. WNT, n = 2; SHH, n = 5; Group 3, n = 19; Group 4, n = 26 samples. Black rectangle indicates the region surrounding *KDM2B* TSS. **d**, Differential expression of H3 methyltransferases and demethylases comparing Group 4 to other medulloblastoma subgroups. WNT, n = 15; SHH, n = 46; Group 3, n = 41; Group 4, n = 65 samples. Dot size indicates statistical significance determined by the two-sided Wald test (DESeq2) with Benjamini-Hochberg correction. **e**, Oncoprint showing association of subgroups, subtypes, copy number alterations, and gene alterations with *KDM2B* expression in Group 3- and 4-medulloblastoma patients (n = 136). Samples were ordered by increasing *KDM2B* expression. Effect size > 0 indicates positive correlation between the alteration and *KDM2B* expression, while effect size < 0 indicates a negative correlation. Only alterations present in at least three samples with |effect size| > 0.2 and *P* < 0.05 are shown. *P* values were determined by the two-sided permutation testing (100,000 permutations).

To mechanistically dissect the pathogenic significance of the EnhBiv state in Group 3 and 4 medulloblastoma, we performed enrichment analysis for the 666 Group 4 EnhBiv signature genes using publicly accessible ChIP–seq datasets (ChEA from Enrichr)^34^ to identify potential regulators that confer this state. We identified several chromatin regulators and transcription factors; KDM2B, a member of the histone lysine demethylase 2 (KDM2) family^35–38^, was the most enriched candidate (Fig. 3a). Similar enrichment analysis performed at the individual EnhBiv-marked clusters (C1–C6) revealed significant KDM2B enrichment in C1–C3 (Extended Data Fig. 4a), which were associated with H3K27me3-driven EnhBiv states in Group 4 (Fig. 2m and Extended Data Fig. 3l). Testing the hypothesis that regulators conferring the enriched EnhBiv state may be specifically upregulated in Group 4, we examined the expression of regulators enriched in each cluster, comparing Group 4 to all other subgroups. KDM2B was the most significantly upregulated candidate (Fig. 3b,c and Extended Data Fig. 4b), and its promoter was marked by higher H3K4me1 and H3K4me3 in Groups 3 and 4 (Extended Data Fig. 4c). KDM2B was also the only differentially expressed enzyme among the H3K4 and/or H3K27 methyltransferases or demethylases in Group 4 compared to other subgroups (Extended Data Fig. 4d).

**Extended Data Fig. 5:**
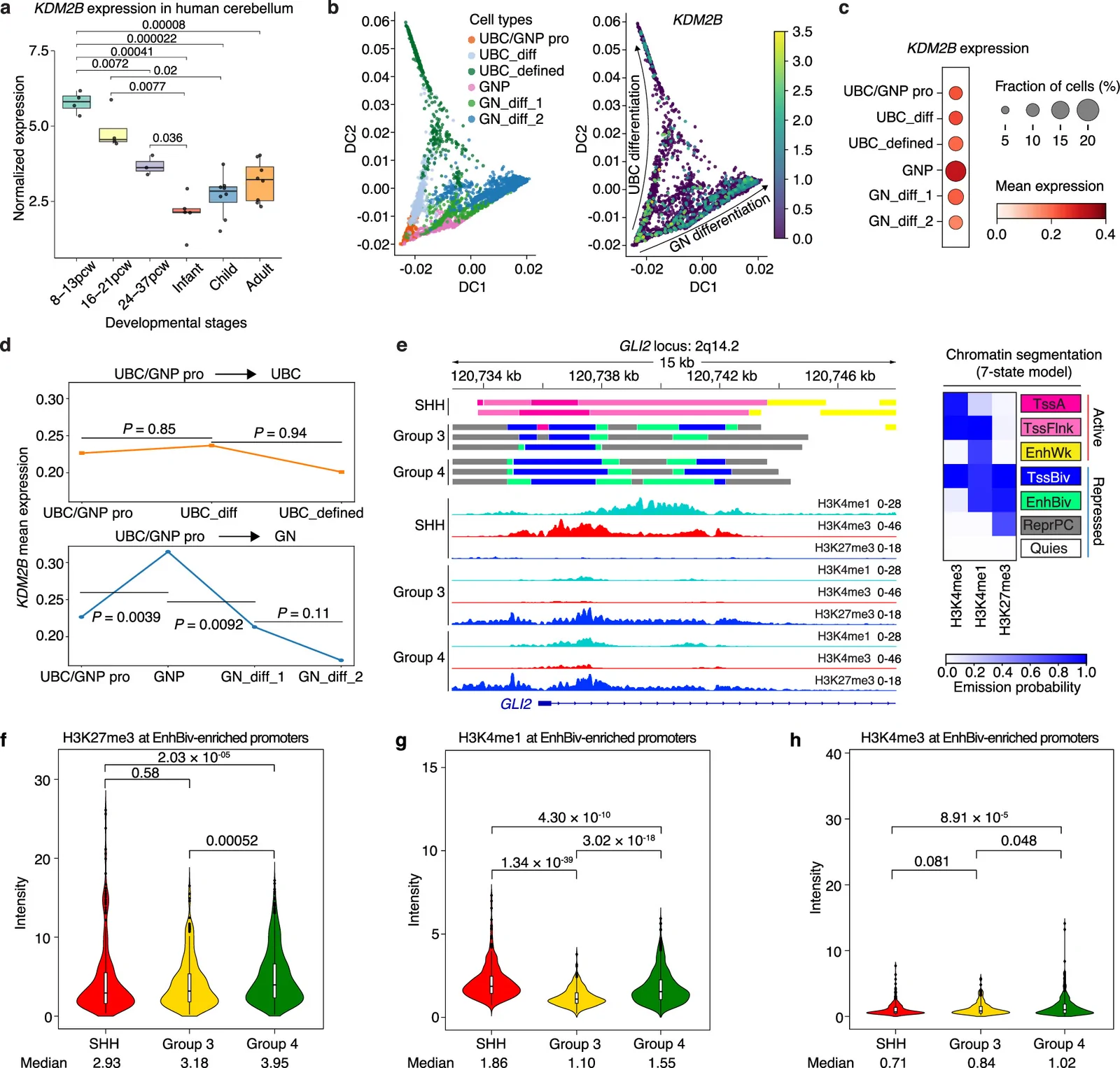
*KDM2B* expression during cerebellar development. **a**, Box plot comparing *KDM2B* expression in human cerebellum samples from BrainSpan across developmental stages (8 - 13 pcw, n = 4; 16 - 21 pcw, n = 4; 24 - 37 pcw, n = 3; Infant, n = 5; Child, n = 8; Adult, n = 8 samples). Boxes represent the IQR, center line indicates the median, and whiskers extend to the most extreme data points within 1.5 × IQR. *P*_adj_ values determined by two-sided t-test and adjusted by the Holm method are shown for significant comparisons. pcw, postconception weeks. **b** UMAP showing human cerebellar rhombic lip-derived lineages (left, n = 4,672 cells) and *KDM2B* expression (right, log1p-normalized counts) from a human cerebellar single-nucleus dataset. **c**, Dot plot showing *KDM2B* expression in rhombic lip-derived lineages. Dots were colored by mean expression and sized according to percentage of cells expressing *KDM2B*. **d**, Line plot showing mean *KDM2B* expression along UBC/GNP progenitor to UBC (top) or to GN (bottom) differentiation trajectories. Statistical significance was determined by the two-tailed Mann-Whitney U test. **e**, Chromatin state and merged histone tracks at the *GLI2* locus in medulloblastoma patient-derived xenografts (PDXs). SHH, n = 2; Group 3, n = 3; Group 4, n = 3 samples. **f**-**h**, Violin plot quantifying CUT&RUN peak intensity of H3K27me3 (**f**), H3K4me1 (**g**) and H3K4me3 (**h**) at EnhBiv-enriched promoters in medulloblastoma PDXs. SHH, n = 313; Group 3, n = 262, Group 4, n = 723 promoters. Violin shows kernel probability density estimation. Boxes represent the IQR, center line indicates the median, and whiskers extend to the most extreme data points within 1.5 × IQR. Dots indicate outliers beyond 1.5 × IQR. *P* values determined by the two-tailed Wilcoxon test are shown for significant comparisons. UBC, unipolar brush cell; GNP, granule neuron progenitor; GN, granule neuron; pro, progenitor; diff, differentiating.

Investigating clinical and molecular features associated with *KDM2B* expression revealed significant correlations between elevated *KDM2B* expression and somatic alterations in chromatin-modifying genes, including *PRDM6*, *KDM6A* and *KMT2C*, suggesting multiple layers of epigenetic dysregulation in these subgroups (Extended Data Fig. 4e). To determine whether high *KDM2B* expression in Group 4 medulloblastoma might be linked to its developmental origin, we examined *KDM2B* expression in bulk^39^ and single-cell transcriptome datasets of cerebellar development^40^. *KDM2B* expression was highest early in cerebellar development and decreased during maturation (Extended Data Fig. 5a), consistent with its established role in brain development^41^. However, *KDM2B* levels were lower in the UBC lineage compared with granule neuron progenitors and did not significantly change during UBC differentiation (Extended Data Fig. 5b–d). Together, these results suggest that elevated *KDM2B* expression in medulloblastoma cannot be explained solely by lineage of origin.

Given that KDM2B has been shown to regulate both H3K4 and H3K27 methylation, either directly or indirectly^37,42,43^, we reasoned that the EnhBiv state may be driven by aberrant KDM2B activity in Groups 3 and 4. To validate the association between KDM2B and the EnhBiv state, we performed CUT&RUN to evaluate KDM2B occupancy as well as H3K27me3, H3K4me1 and H3K4me3 in medulloblastoma patient-derived xenografts (PDXs) representing SHH (*n* = 2), Group 3 (*n* = 3) and Group 4 (*n* = 3) (Supplementary Table 7). Using a seven-state ChromHMM model trained with primary tumor-derived H3K27me3, H3K4me1 and H3K4me3 ChIP–seq data (Fig. 3d), we determined genome-wide chromatin states in PDX samples and identified EnhBiv-enriched promoters in PDXs (Fig. 3e,f and Extended Data Fig. 5e–h). Group 3 and 4 EnhBiv-enriched promoters showed significantly higher KDM2B occupancy than SHH medulloblastoma, with the strongest signal observed in Group 4 (Fig. 3e,f). This was accompanied by a higher H3K27me3 signal in Group 4 than in other subgroups (Fig. 3e and Extended Data Fig. 5f). Consistently, at KDM2B-bound promoters, the enrichment of the EnhBiv state was also higher in Group 4 than in other subgroups (Fig. 3g). Together, these results confirm the association between KDM2B and the EnhBiv state in Group 3 and 4 medulloblastoma.

### KDM2B is a selective dependency in Group 3 and Group 4

**Fig. 4:**
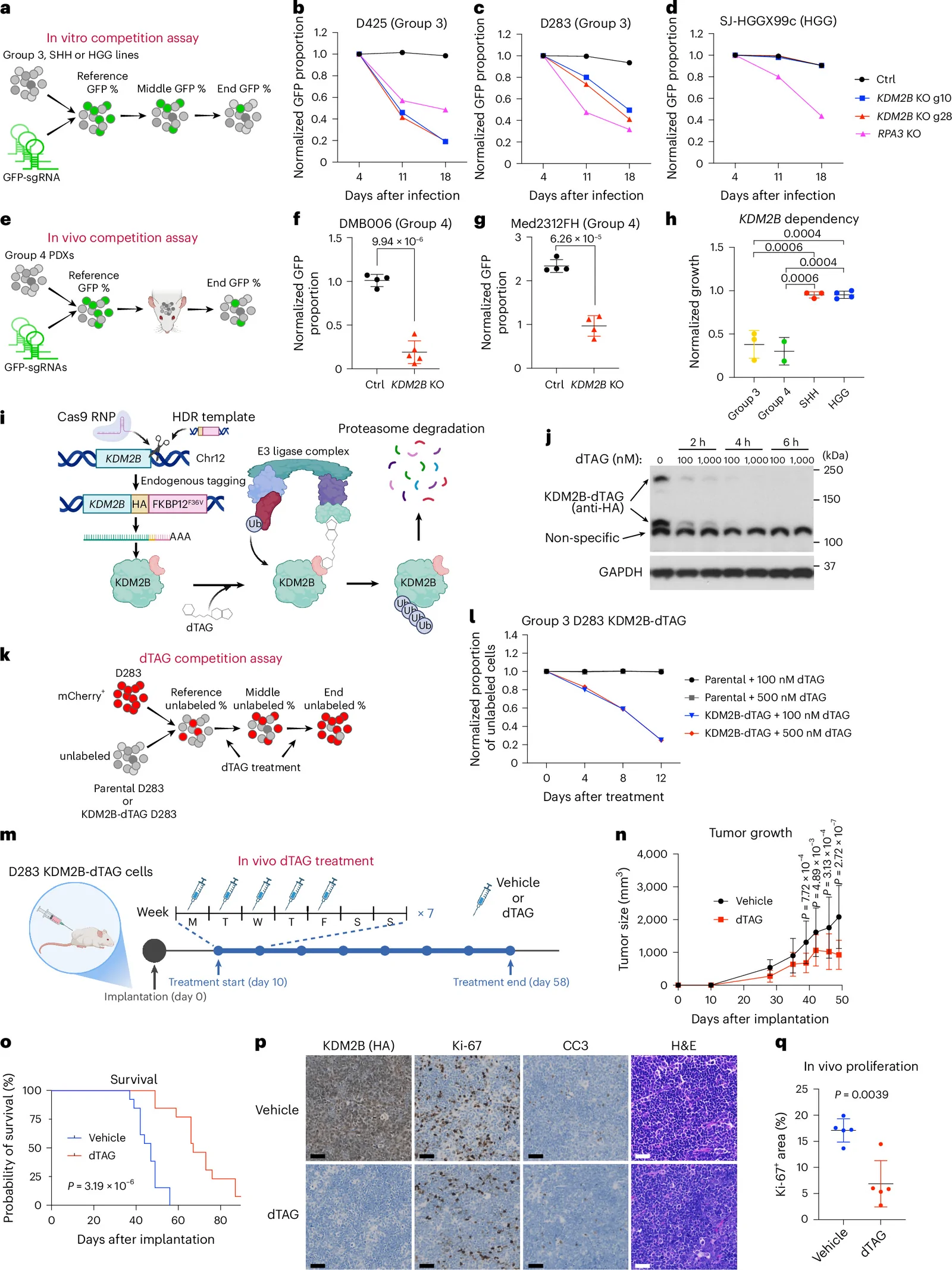
*KDM2B* is a selective dependency in Group 3/4 medulloblastoma. **a**, Schematic of in vitro competition assay. **b**–**d**, In vitro competition assay confirming *KDM2B* as a dependency in Group 3 medulloblastoma cell lines D425 (**b**) and D283 (**c**) but not the high-grade glioma (HGG) cell line SJ-HGGX99c (**d**). *RPA3* gRNA was used as a positive control. Values represent the mean of two biological replicates. The experiments were independently repeated three times for **b** and **c**, and two times for **d**, with similar results. **e**, Schematic of in vivo competition assay. **f**,**g**, In vivo competition assay demonstrating that *KDM2B* is required for growth of Group 4 medulloblastoma PDXs DMB006 (**f**) and Med2312FH (**g**). In **f**, *n* = 4 mice for control; *n* = 5 for *KDM2B* knockout. In **g**, *n* = 4 for both control and *KDM2B* knockout. Center line and error bars indicate mean ± s.d. *P* values determined by a two-tailed *t*-test are shown on the plots. **h**, Summary of *KDM2B* dependency. Data points represent individual cell models (Group 3, *n* = 3; Group 4, *n* = 2; SHH, *n* = 3; HGG, n = 4). Center line and error bars indicate mean ± s.d. Adjusted *P* values generated by one-way ANOVA with Tukey’s multiple-comparison test are shown on the plot for significant comparisons. **i**, Schematic of *KDM2B* endogenous tagging and dTAG-induced degradation of endogenous KDM2B-dTAG protein. **j**, Immunoblotting showing rapid degradation of tagged KDM2B isoforms (detected by HA-Tag antibody) following dTAG treatment. The experiment was independently repeated twice with similar results. **k**, Schematic of in vitro dTAG competition assay. **l**, In vitro dTAG competition assay showing dTAG treatment suppressed the growth of D283 KDM2B-dTAG cells. Values represent the mean of two technical replicates. The experiment was independently repeated three times with similar results. **m**, Schematic of in vivo dTAG treatment. **n**, Tumor growth during in vivo dTAG treatment. Values represent mean ± s.d. (*n* = 13 per group). Statistical significance was determined by two-way ANOVA. **o**, Survival of mice (*n* = 13 per group) receiving in vivo dTAG treatment. Statistical significance was determined by a log-rank Mantel–Cox test. **p**, Hematoxylin and eosin (H&E) and immunohistochemistry staining of KDM2B (by HA-Tag antibody), Ki-67 and cleaved caspase-3 (CC3) for tumors collected after in vivo dTAG treatment. Scale bars, 40 μm. **q**, Quantification of Ki-67 in tumors treated with vehicle or dTAG (*n* = 5 per group) in vivo. Center line and error bars indicate mean ± s.d. *P* value was calculated by two-tailed *t*-test. Schematics created in BioRender: **i**, Tao, R. https://biorender.com/kj1msp9 (2026); **m**, Tao, R. https://biorender.com/qlqs3o0 (2026). Ctrl, control; RNP, ribonucleoprotein; HDR, homology-directed repair; Ub, ubiquitin. Source data

**Extended Data Fig. 6:**
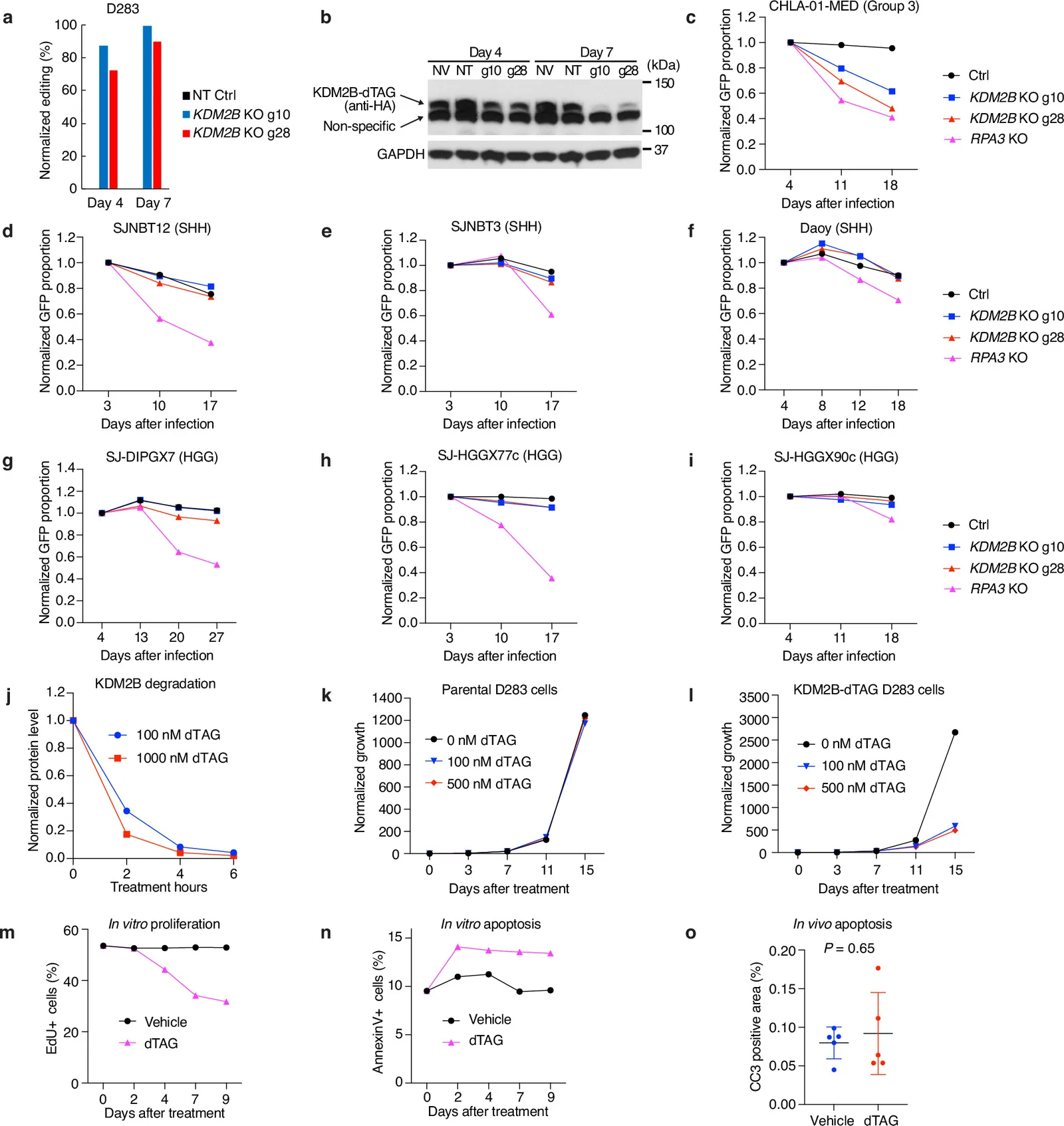
*In vitro* and *in vivo KDM2B* perturbation in medulloblastoma and HGG cell lines. **a**, Normalized editing efficiency for two *KDM2B*-targeting gRNAs: g10 and g28 in D283. **b**, Immunoblot showing depletion of endogenous KDM2B-HA-dTAG protein after CRISPR-mediated editing. KDM2B-HA-dTAG protein was detected using HA-Tag antibody. NV, no virus; NT, non-targeting control; g10 and g28, *KDM2B* knockout gRNAs. Experiment was independently repeated twice with similar results. **c**-**i**, *In vitro* competition assays demonstrating that *KDM2B* is a dependency in Group 3-medulloblastoma CHLA-01-MED (**c**) but is not required for growth of SHH medulloblastoma SJNBT12 (**d**), SJNBT3 (**e**), and Daoy (**f**), or high-grade glioma (HGG) SJ-DIPGX7 (**g**), SJ-HGGX77c (**h**), and SJ-HGGX90c (**i**) cell lines. *RPA3* gRNA was used as a positive control. Values represent mean of two biological replicates. Experiments were independently repeated twice with similar results. Ctrl, control; KO, knockout. **j**, Quantification of KDM2B-dTAG protein level following dTAG treatment from immunoblot in Fig. 4j. Values represent the mean of two biological replicates. Experiment was independently repeated twice with similar results. **k** and **l**, *In vitro* cell growth assay showing growth of parental D283 (**k**) or KDM2B-dTAG D283 cells (**l**) following dTAG treatment. Values represent mean of three technical replicates. Experiment was independently repeated twice with similar results. **m** and **n**, *In vitro* assays of proliferation (**m**) and apoptosis (**n**) for D283 KDM2B-dTAG line. Values represent the mean of three technical replicates. Experiments were independently repeated twice with similar results. **o**, Quantification of cleaved caspase 3 (CC3) in tumors treated with vehicle or dTAG (n = 5/group) *in vivo*. Center line and error bar indicates mean ± s.d. *P* was calculated by the two-tailed t-test. Source data

Aberrant expression of *KDM2B* in Group 4, coupled with its occupancy of EnhBiv state-marked promoters in both Group 3 and Group 4, prompted us to examine whether KDM2B contributes to medulloblastoma pathogenesis. To efficiently disrupt KDM2B function, we designed two gRNAs (g10 and g28) targeting its CXXC DNA-binding domain^44^ and confirmed efficient CRISPR-mediated editing and KDM2B depletion (Extended Data Fig. 6a,b). We examined cell growth following *KDM2B* perturbation in three Group 3 medulloblastoma cell lines (D425, D283 and CHLA-01-MED) using an in vitro competition assay (Fig. 4a). *KDM2B* targeting robustly reduced the proportion of GFP^+^ cells (edited cells) over time (Fig. 4b,c and Extended Data Fig. 6c), suggesting that *KDM2B* is required for in vitro growth of Group 3 medulloblastoma. *KDM2B* knockout (KO) did not affect cell growth of SHH medulloblastoma or high-grade glioma models (Fig. 4d and Extended Data Fig. 6d–i), indicating context-specific dependency. As no authentic Group 4 in vitro culture models are currently available, we targeted *KDM2B* in two Group 4 medulloblastoma PDXs: DMB006 (Group 4) and Med2312FH (Group 3/4 intermediate) (Supplementary Table 7), and performed in vivo competition assays (Fig. 4e), confirming that *KDM2B* is also required for Group 4 medulloblastoma growth in vivo (Fig. 4f,g). These results strongly support *KDM2B* as a selective dependency in Group 3 and Group 4 medulloblastoma (Fig. 4h).

Targeted protein degradation has been successfully used for acutely degrading ‘undruggable’ proteins, including chromatin regulators^45–50^. To independently validate the results of CRISPR-mediated *KDM2B* perturbation and leverage its therapeutic potential, we engineered the degradation tag (dTAG) into the carboxy-terminus of both endogenous *KDM2B* alleles in D283 cells (Fig. 4i). dTAG treatment induced rapid and profound degradation of KDM2B-dTAG protein within 2–4 h (Fig. 4j and Extended Data Fig. 6j). In vitro cell growth assays demonstrated that KDM2B degradation decreased the growth of Group 3 medulloblastoma cells, with a strong reduction (~20%) in proliferation and a slight increase (~5%) in apoptosis (Extended Data Fig. 6k–n). To confirm this result, we developed a dTAG competition assay by mixing either parental or KDM2B-dTAG cells with D283 cells expressing the mCherry fluorescent reporter and measured the proportion of fluorescence-negative cells following dTAG treatment (Fig. 4k). Administration of dTAG rapidly decreased the proportion of KDM2B-dTAG cells but not parental cells, indicating that KDM2B degradation suppressed medulloblastoma growth (Fig. 4l).

To evaluate whether KDM2B targeting prolonged animal survival, we performed an in vivo tumor growth assay. Given that dTAG compounds do not penetrate the blood–brain barrier^51^, we implanted D283 KDM2B-dTAG cells into the flank of mice and treated them with vehicle or dTAG for 7 weeks (Fig. 4m). Mice in the treatment group showed reduced tumor growth and prolonged survival (Fig. 4n,o). Immunohistochemistry characterization of tumors collected after in vivo dTAG treatment identified reduced proliferation without significant changes in apoptosis or histology following KDM2B depletion (Fig. 4p,q and Extended Data Fig. 6o). Collectively, these findings establish *KDM2B* as a selective dependency in Group 3/4 medulloblastoma and validate targeted protein degradation as an effective tool for inhibiting KDM2B.

### KDM2B promotes EnhBiv and represses neuronal differentiation

**Fig. 5:**
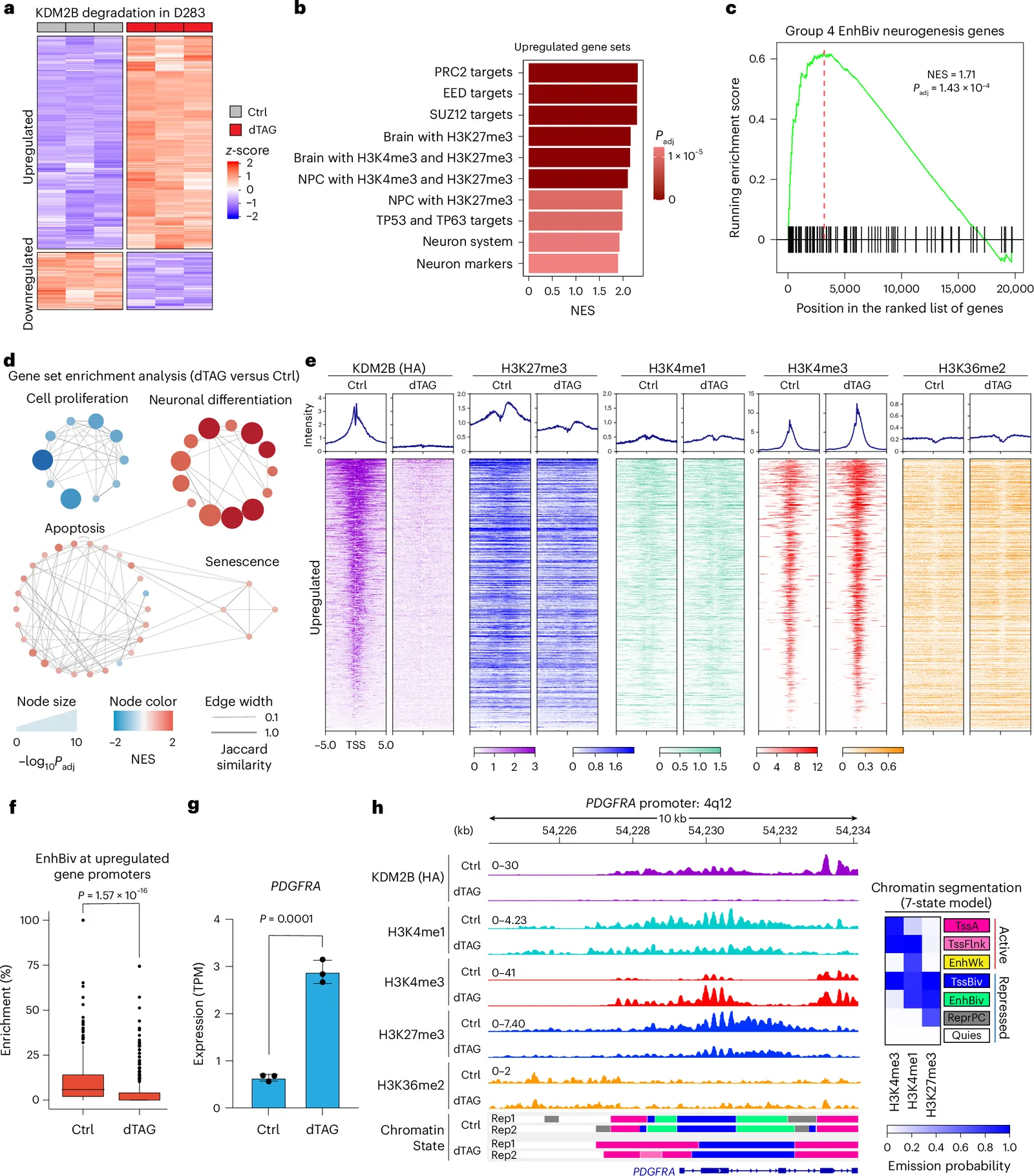
KDM2B promotes the EnhBiv state to repress neuronal differentiation genes. **a**, Heatmap of differentially expressed genes (upregulated, *n* = 1,038; downregulated, *n* = 280) following KDM2B-dTAG degradation in D283. **b**, Top upregulated gene sets by GSEA following KDM2B degradation in D283. **c**, Enrichment analysis showing Group 4 EnhBiv-marked neurogenesis genes (*n* = 134) were predominantly upregulated following KDM2B degradation in D283. For **b** and **c**, significance was determined by a two-sided permutation test (*n* = 1,000). Gene sets with a normalized enrichment score (NES) of >1.5 and *P*_adj_ < 0.05 (Benjamini–Hochberg adjusted) were considered statistically significant. **d**, Enrichment of cell proliferation, neuronal differentiation, apoptosis and senescence gene sets following KDM2B degradation. Nodes were colored by NES and sized by −log_10_*P*_adj_ as determined by two-sided permutation test (*n* = 1,000) with Benjamini–Hochberg FDR correction. Edge width describes Jaccard similarity. **e**, CUT&RUN heatmap of KDM2B (HA-Tag), H3K27me3, H3K4me1, H3K4me3 and H3K36me2 signal at the promoters (TSS ± 5 kb) of upregulated genes (*n* = 1,038) following KDM2B degradation in D283. Curves in the profile plots represent average peak intensity. D283 KDM2B-dTAG cells were treated with either dimethyl sulfoxide control or dTAG for 24 h to assess KDM2B occupancy or for 120 h to assess histone marks. **f**, EnhBiv state enrichment at the promoters of upregulated genes following KDM2B degradation in D283. Only promoters that are marked by EnhBiv state (*n* = 320 promoters) are included in the analysis. Boxes represent the IQR, the center line indicates the median, whiskers extend to the most extreme data points within 1.5× the IQR, and dots indicate outliers beyond 1.5× the IQR. Statistical significance was determined by a two-sided *t*-test. **g,** *PDGFRA* expression following KDM2B degradation in D283. Values represent mean ± s.d. determined from three technical replicates. Statistical significance was determined by two-tailed *t*-test. TPM, transcripts per million. **h**, Chromatin states and merged KDM2B and histone tracks at the *PDGFRA* promoter following KDM2B degradation in D283. Chromatin states are shown for two individual replicates (Rep1 and Rep2). Source data

**Extended Data Fig. 7:**
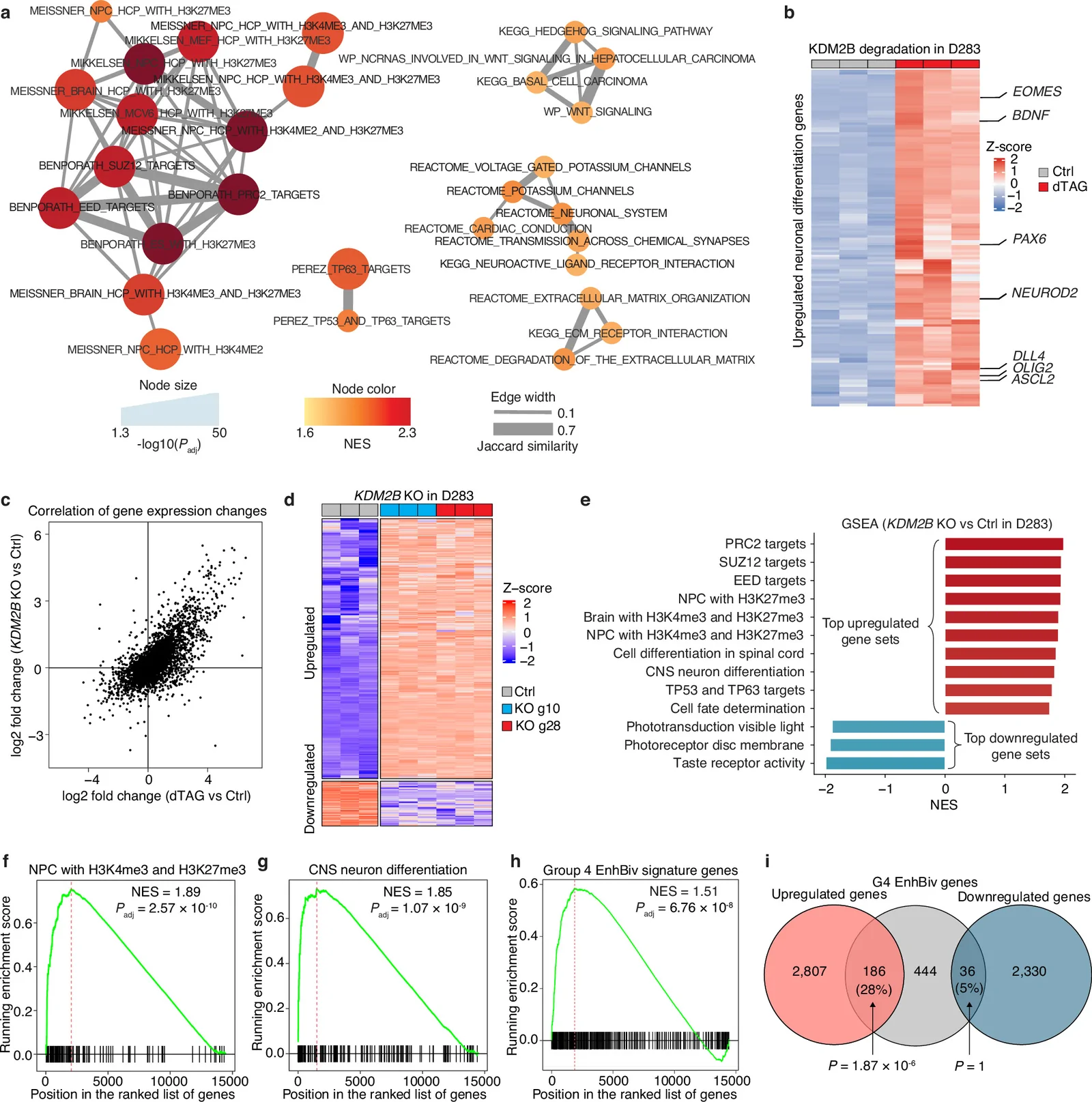
Changes of gene expression following KDM2B perturbation in D283. **a**, GSEA following KDM2B degradation in D283. Nodes represent MSigDB pathway terms (Supplementary Table 9), edge width represents Jaccard similarity, node color represents normalized enrichment score (NES), and node size represents significance determined by the two-sided permutation test (n = 1,000) with Benjamini-Hochberg FDR correction. **b**, Heatmap showing neuronal differentiation genes (n = 140) significantly upregulated following KDM2B degradation in D283. Selected neuronal differentiation genes are labeled. **c**, Positive correlation of gene expression changes (n = 18,087) following KDM2B loss by either CRISPR knockout or degradation as determined by linear regression analysis (R^2^ = 0.55, *P* = 7.77 × 10⁻^5509^). **d**, Heatmap of differentially expressed genes (upregulated, n=671; downregulated, n=116) following *KDM2B* knockout in D283. **e**, Top upregulated and downregulated gene sets by GSEA following *KDM2B* knockout in D283. **f**-**h**, Enrichment analysis showing upregulation of neural progenitor cell (NPC) bivalent state-marked genes (n = 118) (**f**), central nervous system (CNS) neuron differentiation genes (n = 143) (**g**), and Group 4 EnhBiv signature genes (n = 666) (**h**) following *KDM2B* knockout in D283. For **e**-**h**, significance was determined by the two-sided permutation test (n = 1,000). Gene sets with a normalized enrichment score (NES) > 1.5 and *P*_adj_ < 0.05 (Benjamini-Hochberg adjusted) were considered statistically significant. **i**, Venn diagram depicting overlap between 666 Group 4 EnhBiv signature genes and genes upregulated (log2FC > 0, FDR < 0.05) or downregulated (log2FC < 0, FDR < 0.05) upon *KDM2B* knockout in D283 cells. *P* was determined by the two-sided hypergeometric test. Ctrl, control; KO, knockout.

To gain insight into the molecular mechanisms underlying *KDM2B* dependency in Group 3/4 medulloblastoma, we assessed transcriptome-wide changes in D283 cells following KDM2B perturbation. KDM2B degradation predominantly upregulated gene expression (Fig. 5a and Supplementary Table 8), indicating that KDM2B is associated with transcriptional repression in this context. Gene set enrichment analysis (GSEA) identified significant upregulation of PRC2 targets, bivalent state-marked genes and neuronal differentiation genes (Fig. 5b, Extended Data Fig. 7a and Supplementary Table 9). Notably, Group 4 EnhBiv-marked neurogenesis genes identified from DAVID enrichment analysis (Supplementary Table 5) were significantly upregulated after KDM2B inhibition (Fig. 5c). Consistent with the profoundly reduced proliferation observed in vitro and in vivo, KDM2B degradation predominantly upregulated neuronal differentiation genes and reduced the expression of proliferation-associated genes (Fig. 5d and Extended Data Fig. 7b). CRISPR-mediated *KDM2B* knockout in D283 revealed similar transcriptomic changes (Extended Data Fig. 7c–i and Supplementary Table 10). These results suggest that KDM2B contributes to Group 3/4 medulloblastoma maintenance by promoting the EnhBiv state and repressing the expression of neuronal differentiation genes.

**Extended Data Fig. 8:**
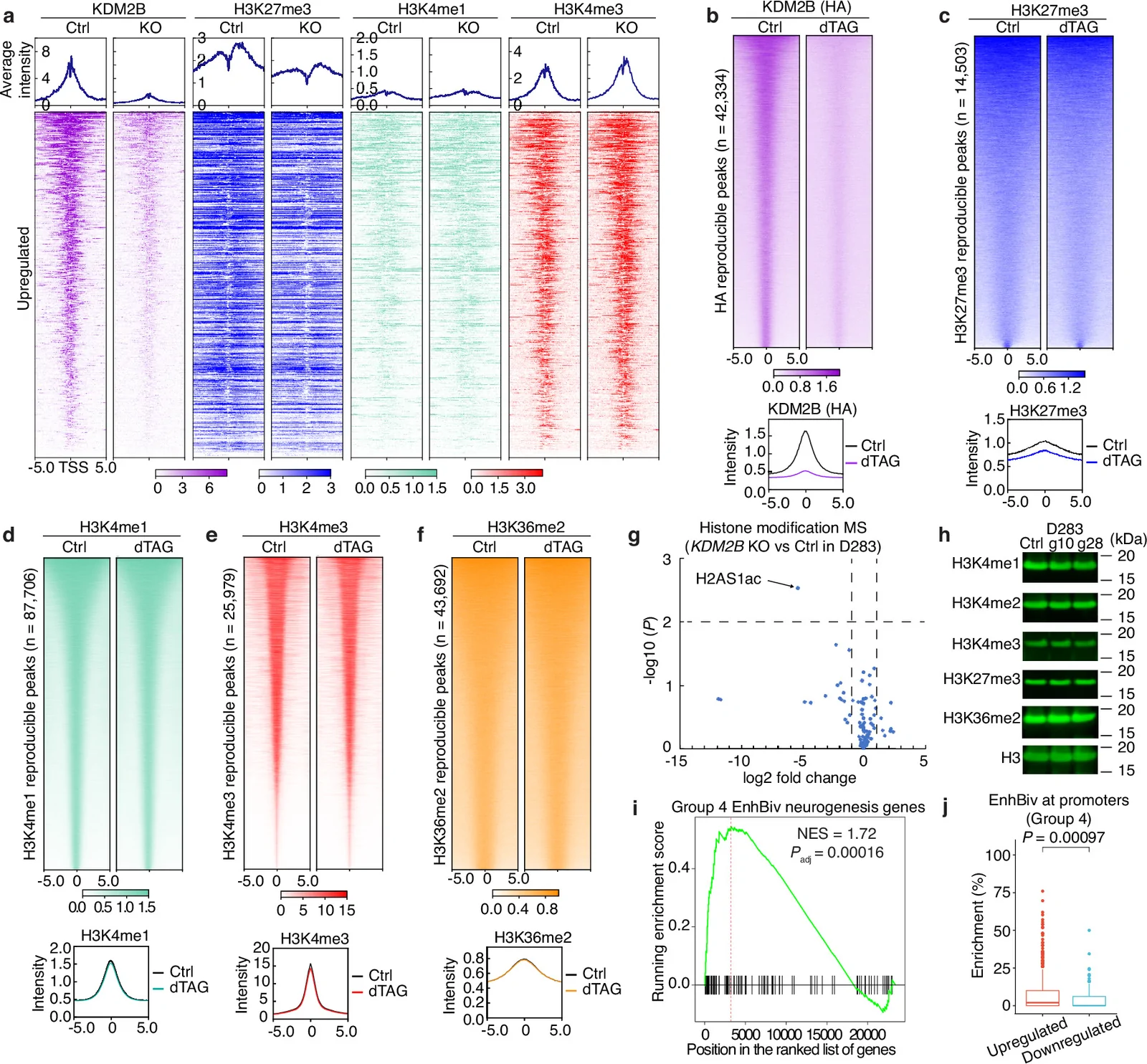
Histone modification changes following *KDM2B* perturbation. **a**, CUT&RUN analysis showing changes of KDM2B, H3K27me3, H3K4me1 and H3K4me3 at the promoters of upregulated genes (n = 671) following *KDM2B* knockout in D283 cells. **b-f**, CUT&RUN heatmaps (top) and profile plots (bottom) showing global changes of KDM2B (**b**), H3K27me3 (**c**), H3K4me1 (**d**), H3K4me3 (**e**) and H3K36me2 (**f**) following KDM2B degradation. Curves in the profile plots represent average peak intensity. D283 KDM2B-dTAG cells were treated with either DMSO control or dTAG for 24 h to assess KDM2B occupancy or for 120 h to assess histone marks. **g**, Volcano plot showing changes of total histone modifications determined by histone mass spectrometry analysis following *KDM2B* knockout in D283. Statistical significance was determined by the two tailed t-test. No adjustments were made for multiple comparisons. **h**, Immunoblot examining histone modifications comparing *KDM2B* knockout (g10 and g28) with control in D283. Experiment was independently repeated twice with similar results. **i**, Enrichment analysis demonstrating a significant upregulation of Group 4 EnhBiv marked neurogenesis genes (n = 134) following *KDM2B* knockout in DMB006. Significance was determined by the two-sided permutation test (n= 1,000) with normalized enrichment score (NES) > 1.5 and *P*_adj_ < 0.05 (Benjamini-Hochberg adjusted) considered statistically significant. **j**, Enrichment of EnhBiv state at the promoters of differentially expressed genes. Differentially expressed genes were determined by RNA-seq following *KDM2B* knockout in DMB006. EnhBiv states were determined by CUT&RUN in wild-type Group 4 PDXs (Fig. 3e). Only promoters that are marked by EnhBiv state are included in the analysis: upregulated, n = 253, downregulated, n = 42 promoters. Boxes represent the IQR, center line indicates the median, and whiskers extend to the most extreme data points within 1.5 × IQR. Dots indicate outliers beyond 1.5 × IQR. Statistical tests were conducted using the two-sided t-test. Ctrl, control; KO, knockout; MS, mass spectrometry. Source data

To confirm that KDM2B regulates EnhBiv, we performed CUT&RUN for KDM2B, H3K27me3, H3K4me1 and H3K4me3 in D283 cells. Upon KDM2B degradation, KDM2B peaks were overwhelmingly lost from the promoters of upregulated genes, concurrent with a profound reduction of H3K27me3 and an increase of H3K4me3. By contrast, H3K4me1 did not change (Fig. 5e). Although KDM2B is also a demethylase for H3K36me2 (ref. ^36^), we did not observe changes in this mark after KDM2B degradation (Fig. 5e). CUT&RUN performed following *KDM2B* knockout in D283 recapitulated these findings (Extended Data Fig. 8a). At the global level, H3K27me3 was slightly reduced, and no other histone marks dramatically changed following KDM2B degradation (Extended Data Fig. 8b–f), suggesting that KDM2B regulated chromatin at specific genomic loci rather than globally. Consistently, mass spectrometry quantification of histone PTMs and immunoblotting confirmed that total histone PTM levels were unchanged after *KDM2B* knockout in D283 (Extended Data Fig. 8g,h and Supplementary Table 11). Applying the ChromHMM seven-state model to this CUT&RUN dataset confirmed a significant reduction of EnhBiv enrichment at promoters of upregulated genes after KDM2B degradation (Fig. 5f), suggesting that reduced H3K27me3 following KDM2B targeting contributes to the loss of the EnhBiv state. *PDGFRA*, a gene regulating brain development^52^, was upregulated after KDM2B degradation (Fig. 5g), concurrent with decreased H3K27me3, increased H3K4me3 and correspondingly reduced EnhBiv state enrichment at its promoter (Fig. 5h).

To validate these observations in Group 4 medulloblastoma, we performed RNA-seq in DMB006 PDX cells following *KDM2B* knockout in vitro (Supplementary Table 12). Group 4 EnhBiv-marked neurogenesis genes were predominantly upregulated (Extended Data Fig. 8i), consistent with observations in Group 3 D283 cells. Overlapping differentially expressed genes detected in *KDM2B*-targeted DMB006 cells with CUT&RUN profiles generated from unmodified PDX confirmed that genes marked by EnhBiv state in Group 4 medulloblastoma were upregulated upon *KDM2B* knockout (Extended Data Fig. 8j). Taken together, these data suggest that KDM2B suppresses the expression of neuronal differentiation genes by promoting H3K27me3 and chromatin bivalency in Group 3/4 medulloblastoma.

### KDM2B promotes PRC1 and PRC2 sequential recruitment

**Fig. 6:**
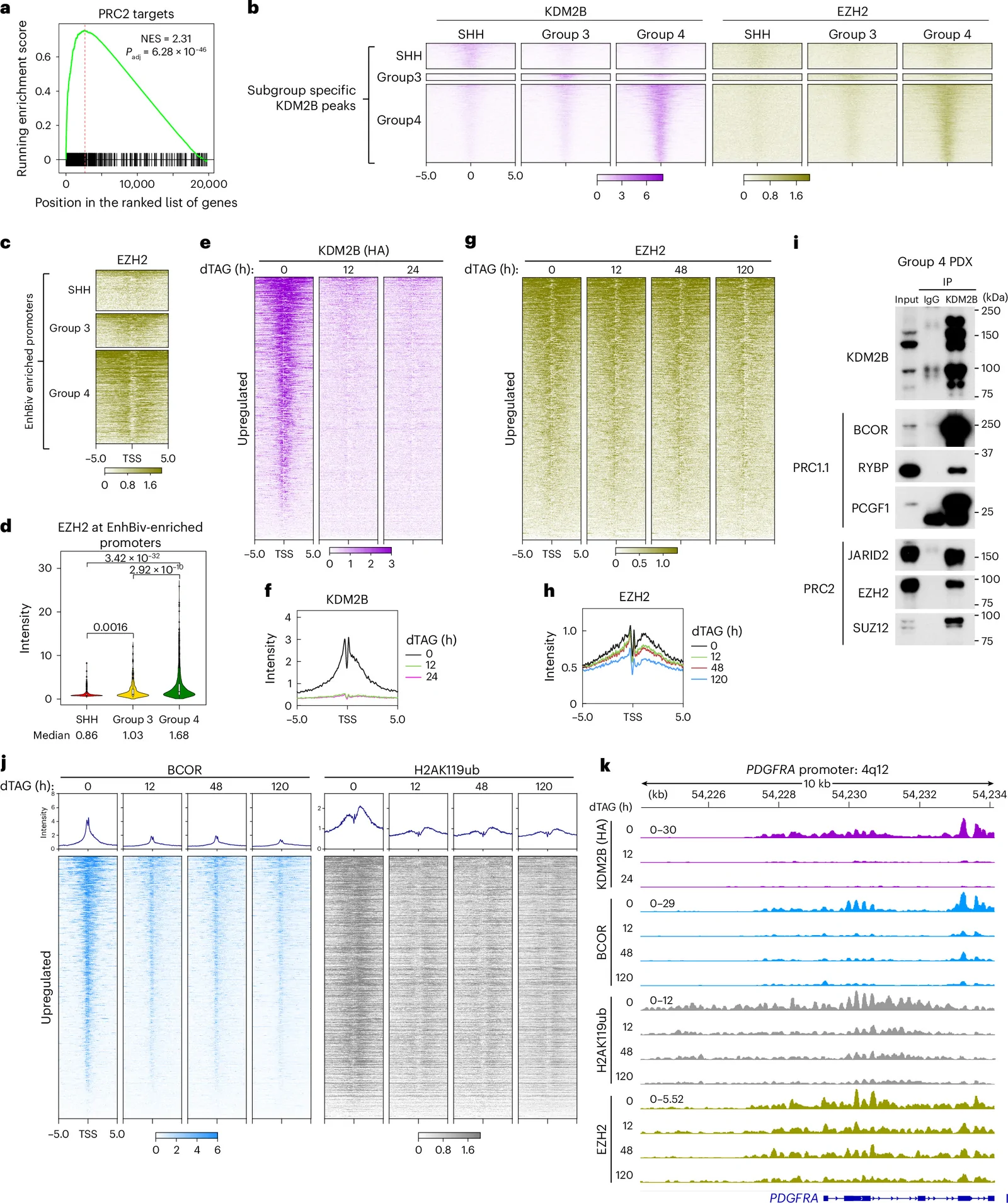
KDM2B promotes PRC1 and PRC2 sequential recruitment to gene promoters. **a**, Enrichment analysis demonstrating that PRC2 target genes (*n* = 432) are significantly upregulated following KDM2B degradation in D283. Significance was determined by two-sided permutation test (*n* = 1,000) with *P* values adjusted using the Benjamini–Hochberg FDR correction. **b**, CUT&RUN heatmap showing KDM2B and EZH2 co-occupancy at subgroup-specific KDM2B-bound regions (SHH, *n* = 1,175; Group 3, *n* = 307; Group 4, *n* = 3,591) in medulloblastoma PDXs. Regions of peak center (0) ± 5 kb are shown. **c**, Heatmap of EZH2 binding at EnhBiv-enriched promoters in medulloblastoma PDXs. **d**, Violin plot quantifying CUT&RUN peak intensity of EZH2 in **c**. Violin shows kernel probability density estimation. Boxes represent the IQR, center line indicates the median, whiskers extend to the most extreme data points within 1.5× the IQR and dots indicate outliers beyond 1.5× the IQR. *P* values determined by the two-tailed Wilcoxon test are shown on the plot. For **c** and **d**, SHH, *n* = 313; Group 3, *n* = 262; Group 4, *n* = 723 promoters. **e**,**f**, CUT&RUN heatmaps (**e**) and profile plot (**f**) showing KDM2B occupancy at the promoters of upregulated genes (*n* = 1,038) following KDM2B degradation in D283 at indicated time points (hours). KDM2B-24 h dTAG treatment data were taken from KDM2B-dTAG treatment in Fig. 5e. **g**,**h**, CUT&RUN heatmaps (**g**) and profile plot (**h**) showing EZH2 occupancy at the promoters of upregulated genes (*n* = 1,038) following KDM2B degradation in D283. **i**, Co-immunoprecipitation (IP) demonstrating the interaction between KDM2B and PRC1.1 components (BCOR, RYBP and PCGF1) or PRC2 components (JARID2, EZH2 and SUZ12) in Group 4 medulloblastoma PDX (DMB006). KDM2B was pulled down from nuclear extract using a KDM2B antibody with an IgG antibody as a control. The experiment was independently repeated twice with similar results. **j**, CUT&RUN analysis showing changes in BCOR and H2AK119ub occupancy at the promoters of upregulated genes (*n* = 1,038) following KDM2B degradation in D283. Curves in the profile plots represent average peak intensity. **k**, KDM2B, BCOR, H2AK119ub and EZH2 tracks at the *PDGFRA* promoter following KDM2B degradation in D283. Source data

**Extended Data Fig. 9:**
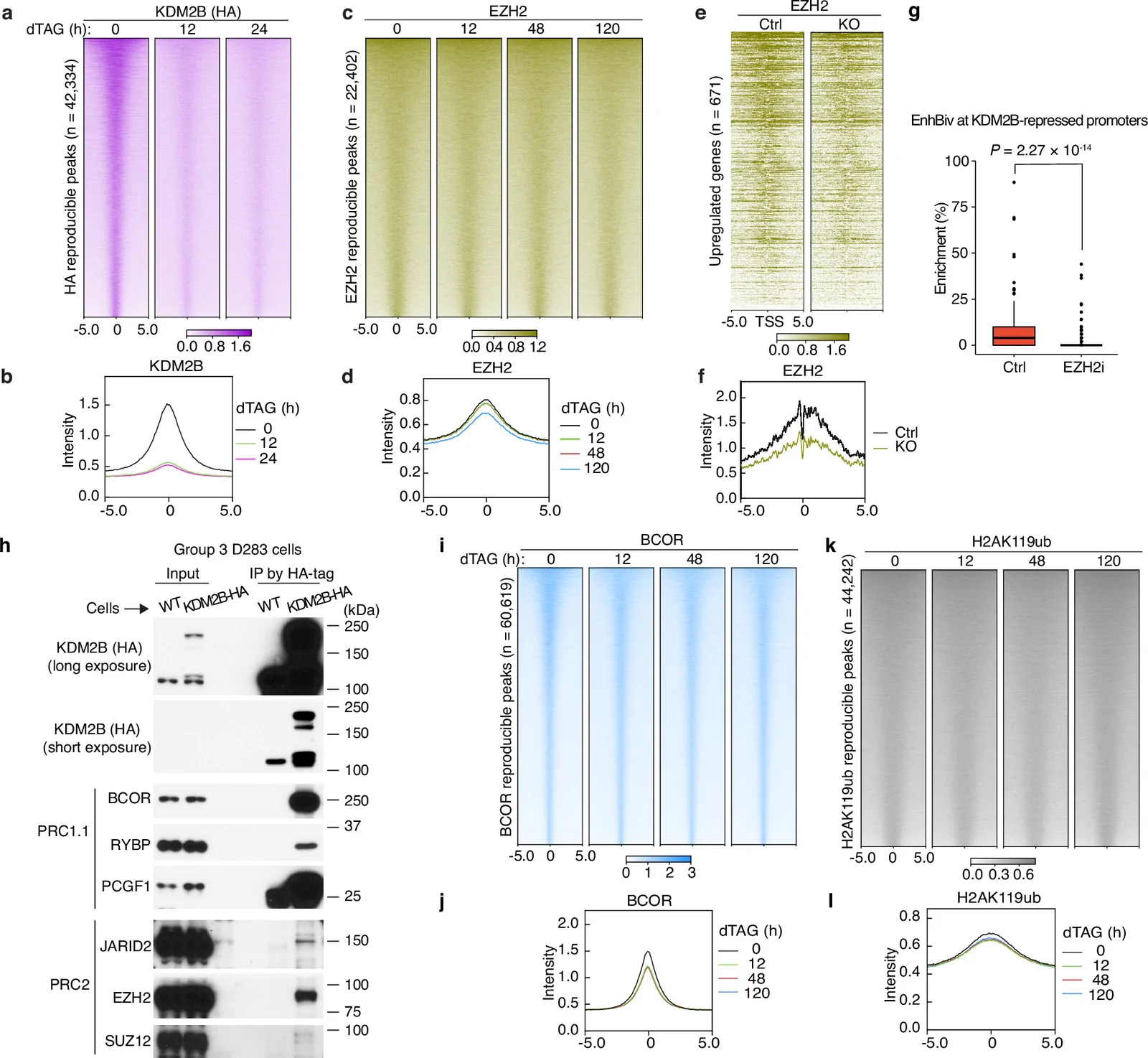
Changes to KDM2B, EZH2, BCOR and H2AK119ub following *KDM2B* perturbation in D283. **a** and **b**, CUT&RUN heatmaps (**a**) and profile plot (**b**) showing global changes of KDM2B occupancy following KDM2B degradation in D283 at indicated time points (h). KDM2B-24 h dTAG treatment data was taken from KDM2B-dTAG treatment in Extended Data Fig. 8b. **c** and **d**, CUT&RUN heatmaps (**c**) and profile plot (**d**) showing global changes of EZH2 occupancy following KDM2B degradation in D283. **e** and **f**, CUT&RUN heatmaps (**e**) or profile plots (**f**) showing changes of EZH2 occupancy at the promoter of upregulated genes (n = 671) following *KDM2B* knockout in D283. Ctrl, control; KO, knockout. **g**, Loss of EnhBiv state at KDM2B repressed promoters (genes upregulated upon KDM2B degradation) after treating D283 cells with EZH2 inhibitor (EZH2i) for 120 h. Only promoters (n = 141) with detected EnhBiv state enrichment are included in the analysis. Boxes represent the IQR, center line indicates the median, and whiskers extend to the most extreme data points within 1.5 × IQR. Dots indicate outliers beyond 1.5 × IQR. *P* value determined by the two-sided t-test was shown. **h**, Co-immunoprecipitation demonstrating the interaction between KDM2B and PRC1 components (BCOR, RYBP, and PCGF1) or PRC2 components (JARID2, EZH2 and SUZ12) in D283 cells. KDM2B was pulled down from wild-type control D283 or D283 KDM2B-HA-dTAG nuclear extracts by HA-Tag antibody. Experiment was independently repeated twice with similar results. **i** and **j**, CUT&RUN heatmaps (**i**) and profile plot (**j**) showing global changes of BCOR occupancy following KDM2B degradation in D283. **k** and **l**, CUT&RUN heatmaps (**k**) and profile plot (**l**) showing global changes of H2AK119ub following KDM2B degradation in D283. Source data

KDM2B promotes PRC1.1-mediated H2AK119ub at unmethylated CpG islands, which subsequently recruit PRC2 to deposit H3K27me3 (ref. ^42^). As loss of KDM2B reduced H3K27me3 (Fig. 5e) and derepressed PRC2 target gene expression in medulloblastoma cells (Figs. 5b and 6a), we reasoned that KDM2B may confer the EnhBiv state by promoting PRC2 recruitment. To test this hypothesis, we performed CUT&RUN for KDM2B and EZH2 in medulloblastoma PDX models and found that they co-occupied genomic regions in Group 4 (Fig. 6b). Like KDM2B, EZH2 intensity was highest at Group 4 EnhBiv-enriched promoters, followed by Group 3, and lowest in SHH medulloblastoma (Fig. 6c,d). To investigate whether KDM2B contributed to EZH2 recruitment, we examined the dynamic changes of EZH2 binding following acute and chronic KDM2B degradation. At promoters of upregulated genes, KDM2B was lost by 12 h post dTAG treatment (Fig. 6e,f). Notably, EZH2 occupancy was reduced as early as 12 h and continued to decrease over time (Fig. 6g,h). Genome-wide, KDM2B was depleted by 12 h, whereas EZH2 recruitment showed only modest reduction at 120 h (Extended Data Fig. 9a–d), suggesting that KDM2B preferentially recruits EZH2 to specific promoters. CUT&RUN performed after *KDM2B* knockout confirmed reduced EZH2 occupancy at promoters of upregulated genes (Extended Data Fig. 9e,f). Likewise, treatment with an EZH2 inhibitor reduced EnhBiv enrichment at promoters of KDM2B-repressed genes (Extended Data Fig. 9g).

**Extended Data Fig. 10:**
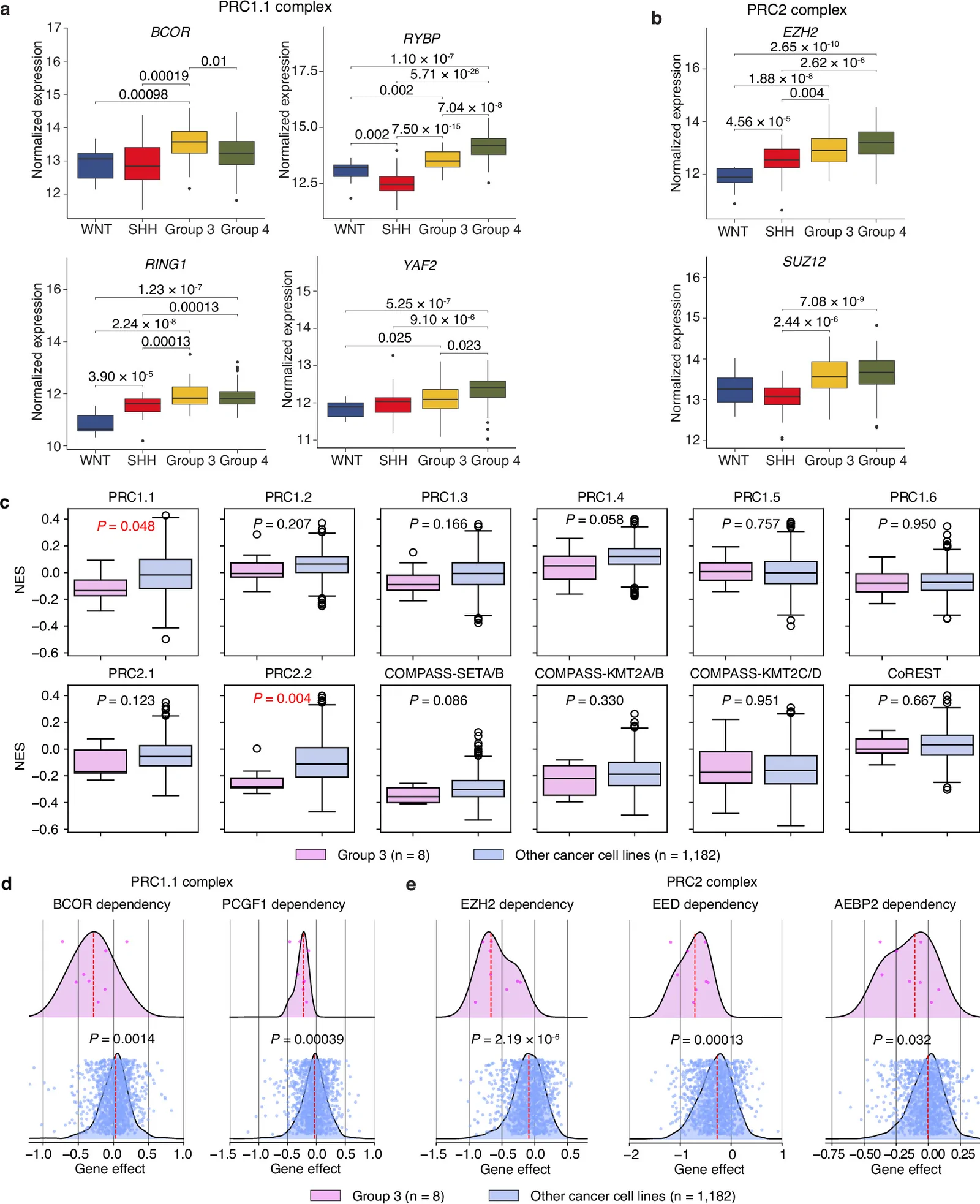
Expression and growth dependency of PRC1 and PRC2 subunits. **a** and **b**, Box plot showing expression of PRC1.1 (**a**) and PRC2 (**b**) subunits across medulloblastoma subgroups. WNT, n = 15; SHH, n = 46; Group 3, n = 41; Group 4, n = 65 samples. Boxes represent the IQR, center line indicates the median, and whiskers extend to the most extreme data points within 1.5 × IQR. Dots indicate outliers beyond 1.5 × IQR. Statistical significance was assessed using the two-sided t-test, with *P* adjusted by the Holm method. *P*_adj_ values are shown for significant comparisons. **c**, Box plot showing growth dependency of chromatin modifying complexes (Supplementary Table 13) comparing Group 3-medulloblastoma (n = 8) with all other cancer cell lines (n = 1,182) in DepMap. Normalized enrichment scores (NES) were computed from DepMap dependency scores for each line, with lower scores indicating stronger dependencies. Boxes represent the IQR, center line indicates the median, and whiskers extend to the most extreme data points within 1.5 × IQR. Dots indicate outliers beyond 1.5 × IQR. Statistical significance was determined by the two-tailed Mann-Whitney U test, with *P* < 0.05 (red text) considered statistically significant. **d** and **e**, Gene effect of PRC1.1 (**d**) and PRC2 (**e**) subunits in Group 3-medulloblastoma (n = 8) and all other cancer cell lines (n = 1,182). A lower gene effect score means that a gene is more likely to be a dependency. The red dashed line indicates median values of gene effect. Statistical significance was determined by the two-tailed Mann-Whitney U test, with *P* < 0.05 indicating Group 3 selective dependency.

To determine whether PRC1.1 mediates EZH2 recruitment in Group 3 and 4 medulloblastoma models, we conducted co-immunoprecipitation and confirmed strong interactions between KDM2B and PRC1.1 (BCOR, PCGF1 and RYBP)^53^ (Fig. 6i and Extended Data Fig. 9h). PRC2 components, including EZH2, SUZ12 and JARID2, also physically associated with KDM2B, albeit weaker than PRC1.1 (Fig. 6i and Extended Data Fig. 9h). Following KDM2B degradation, BCOR and H2AK119ub were dramatically reduced at promoters of upregulated genes by 12 h, indicative of impaired PRC1.1 recruitment (Fig. 6j), whereas the global decrease was modest (Extended Data Fig. 9i–l). At the *PDGFRA* promoter, BCOR, H2AK119ub and EZH2 decreased as early as 12 h post dTAG treatment (Fig. 6k), consistent with the observed reduction in H3K27me3 and EnhBiv state at the same locus (Fig. 5h). These rapid changes in Polycomb dynamics support a model in which KDM2B-dependent PRC1.1 recruitment promotes H2AK119 ubiquitination and subsequently instructs EZH2 to deposit H3K27me3 at KDM2B-repressed promoters. Multiple PRC1.1 and PRC2 components (Supplementary Table 13) were also highly expressed in these subgroups relative to WNT and SHH and were selectively required for growth of Group 3 medulloblastoma cell lines in vitro (Extended Data Fig. 10).

### KDM2B sustains malignancy through its DNA-binding domain

**Fig. 7:**
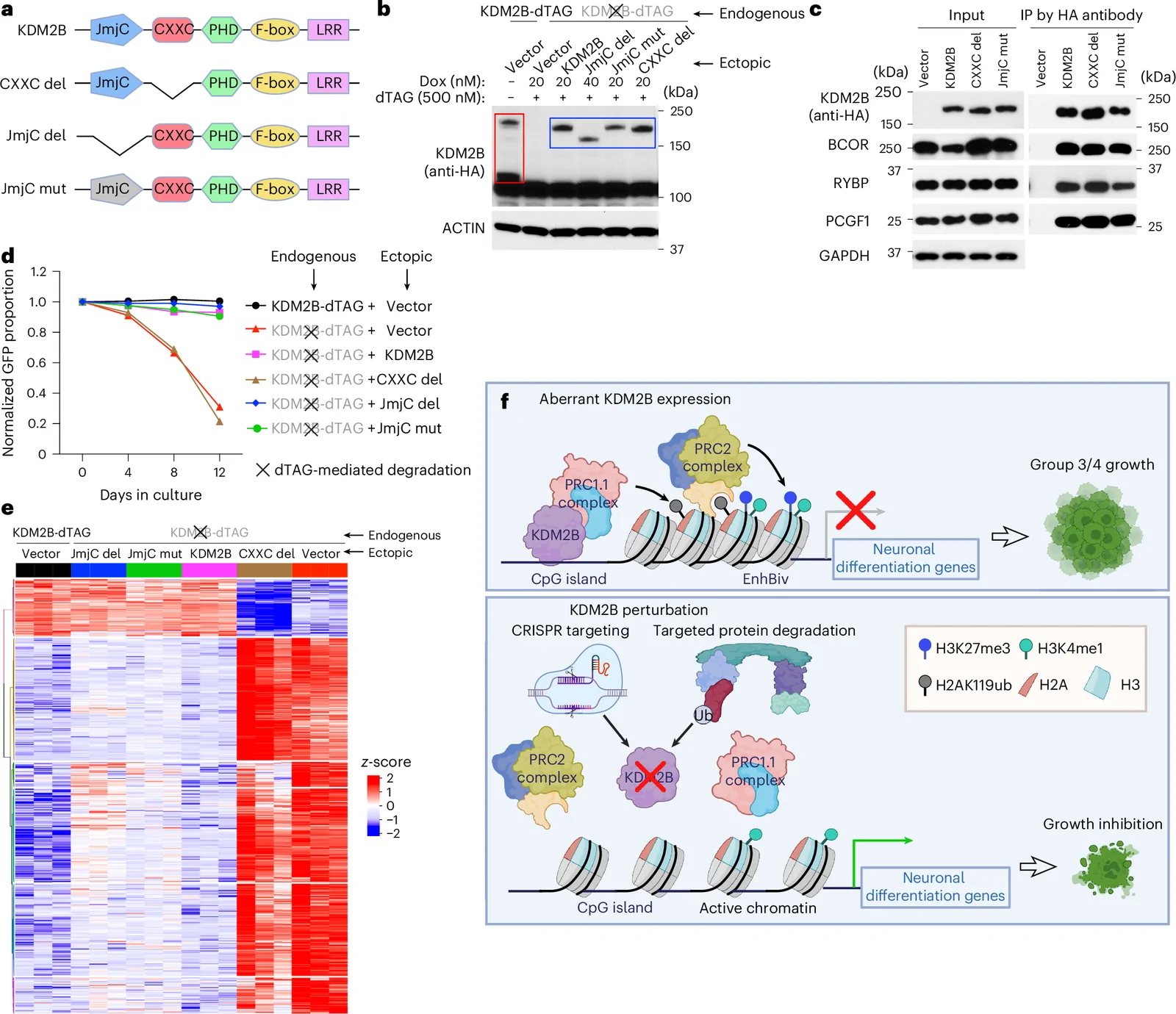
KDM2B-dependent regulation of gene expression and medulloblastoma growth depends on DNA binding but not demethylase activity. **a**, Schematic depicting wild-type and mutant KDM2B. KDM2B protein domains: Jumonji C (JmjC) demethylase domain, CXXC DNA-binding domain, plant homeodomain (PHD) domain, F-box domain and Leucine-Rich Repeat (LRR) domain. KDM2B mutants were generated by deleting the entire CXXC (CXXC del) or JmjC (JmjC del) domain or by introducing mutations in the JmjC domain to abolish its demethylase activity (JmjC mut). **b**, Immunoblot of HA-tagged endogenous KDM2B-dTAG (red rectangle) and Dox-induced ectopic wild-type or mutant KDM2B expression (blue rectangle) in D283 KDM2B-dTAG cells treated with or without dTAG or Dox. **c**, Co-immunoprecipitation of ectopically expressed wild-type or mutant KDM2B and endogenous PRC1.1 subunits (BCOR, RYBP and PCGF1). D283 KDM2B-dTAG cells were treated with Dox and dTAG to ectopically express wild-type or mutant KDM2B and degrade the endogenous KDM2B-dTAG protein. KDM2B was pulled down from nuclear extracts by an HA-Tag antibody. For **b** and **c**, experiments were independently repeated twice with similar results. **d**, In vitro competition assay examining growth rescue of wild-type or mutant KDM2B after degrading the endogenous KDM2B-dTAG protein in D283. Values represent the mean of two technical replicates. The experiment was independently repeated twice with similar results. **e**, Heatmap showing gene expression changes following KDM2B degradation with or without rescue by wild-type or mutant KDM2B in D283 KDM2B-dTAG cells. **f**, Graphical summary depicting a working model of KDM2B in medulloblastoma. Aberrant KDM2B expression maintains Group 3/4 medulloblastoma growth by recruiting the PRC1.1 complex and inducing H2AK119 ubiquitination, which subsequently recruits the PRC2 complex and promotes EnhBiv state formation at the promoter of neuronal differentiation genes. KDM2B perturbation by either CRISPR targeting or targeted protein degradation abolishes PRC1/PRC2-mediated EnhBiv formation, derepresses neuronal differentiation genes and inhibits Group 3/4 medulloblastoma growth. Schematics created in BioRender: **a**, Tao, R. https://biorender.com/o4311w5 (2026); **f**, Tao, R. https://BioRender.com/3fylm2x (2026). Source data

KDM2B-dependent sequential recruitment of PRC1.1 and PRC2 has been shown to be mediated by its CXXC DNA-binding domain^42,54,55^. To functionally validate that KDM2B regulates the EnhBiv state through PRC1/PRC2 recruitment, we generated doxycycline (Dox)-inducible constructs expressing wild-type KDM2B or mutants lacking either the CXXC domain or the JmjC demethylase domain (deletion or point mutation) (Fig. 7a). We titrated Dox concentrations to ectopically express these constructs at levels comparable to endogenous KDM2B in the D283 KDM2B-dTAG line (Fig. 7b). Co-immunoprecipitation confirmed that these mutations did not disrupt interactions with PRC1.1 subunits (Fig. 7c). Competition assays showed that wild-type KDM2B and JmjC mutants fully rescued the growth defect caused by endogenous KDM2B degradation, whereas the CXXC mutant failed to rescue (Fig. 7d). Additionally, KDM2B depletion predominantly upregulated gene expression, which was largely rescued by wild-type or JmjC mutant expression, but not by the CXXC mutant (Fig. 7e). These results indicate that KDM2B regulates gene expression and medulloblastoma growth primarily through CXXC domain-mediated PRC1/PRC2 recruitment, whereas the demethylase activity is dispensable (Fig. 7f).

## Discussion

Chromatin modifier alterations persist in nearly half of all medulloblastoma cases, implicating deregulation of chromatin and downstream transcriptional programs as a fundamental process underlying medulloblastoma pathogenesis. However, the mechanisms by which aberrant chromatin modifier activity drives subgroup-specific tumorigenesis remain unclear. Here, we assessed the chromatin landscape of medulloblastoma subgroups through the lens of combinatorial histone PTM states determined from a large cohort of primary patient tumors. As anticipated from transcriptional and DNA methylation signatures that robustly discriminate inter-tumoral heterogeneity in medulloblastoma^6,7,21,56,57^, molecular subgroups differed by chromatin states. Among differential chromatin states between subgroups, the EnhBiv state was most pronounced, particularly in Group 3 and 4 medulloblastoma, which was associated with aberrant KDM2B expression and promoter occupancy in these subgroups. Through a series of perturbation and mechanistic studies, we demonstrated that KDM2B promotes the EnhBiv state by facilitating PRC1.1 and PRC2 sequential recruitment to repress neuronal differentiation genes. Functional assays rigorously validated KDM2B as a Group 3/4 medulloblastoma-selective dependency and nominated the KDM2B–PRC1.1–PRC2 axis as a rational therapeutic target (Fig. 7f).

KDM2B regulates diverse biological processes, including brain development, embryonic stem cell maintenance, induced pluripotent stem cell generation and tumorigenesis^35,38,41,44,54,58–62^. The mechanisms underlying these functions vary and depend on context. For example, KDM2B exhibits both H3K4me3 and H3K36me2 demethylase activity^36,37,58^ and promotes somatic cell reprogramming through demethylating H3K36me2 (refs. ^38,59^). As a component of PRC1.1, KDM2B also recruits PRC1.1 to chromatin through its CXXC domain, independent of histone demethylase activity^42,53–55,63^. H2AK119 ubiquitination by PRC1.1 is required for subsequent PRC2 recruitment and H3K27me3 deposition to initiate Polycomb domain formation at CpG islands^42,43^, a process critical for embryonic stem cell maintenance and embryonic development^42,54,63^. Although low-frequency (1%) *KDM2B* mutations have been observed in medulloblastoma^11^, their biological significance remains unclear. We assessed Polycomb complex recruitment kinetics and dissected KDM2B domain-specific functions. Empowered by the dTAG system, we observed rapid loss of BCOR, H2AK119ub and EZH2 at promoters of KDM2B-repressed genes within hours of KDM2B degradation, indicating impaired recruitment of PRC1.1/PRC2 by KDM2B rather than indirect effects of prolonged depletion. This mechanism was validated by functional studies of KDM2B domain mutants, which determined a requirement for the CXXC domain, whereas demethylase activity was dispensable. Of note, our study focused on KDM2B-dependent gene regulation but does not exclude PRC1.1-independent mechanisms of Polycomb repression. Together, these findings provide mechanistic insights into how aberrant KDM2B-dependent recruitment of Polycomb promotes chromatin bivalency and sustains malignancy in Group 3 and 4 medulloblastoma.

There are currently no effective targeted therapies for Group 3 and 4 medulloblastoma. Non-specific epigenetic therapies, including certain classes of histone deacetylase and bromodomain inhibitors, have shown encouraging results in Group 3 medulloblastoma model systems^64–69^. However, these drugs and compounds have yet to demonstrate sustained clinical efficacy in patients. Likewise, although Group 3 and 4 medulloblastomas sustain somatic driver alterations in a subset of common genes (for example, *GFI1*, *GFI1B*, *KMT2D*)^6^, these genes are presumed to contribute to only a modest fraction of cases overall, limiting their potential as therapeutic targets. By contrast, the KDM2B-associated EnhBiv state, which promotes tumor growth by suppressing neuronal differentiation, is broadly enriched across Group 3 and 4 medulloblastoma and is therefore predicted to contribute to malignancy in a larger fraction of patients. Consistent with this prediction, KDM2B targeting selectively inhibited both Group 3 and 4 medulloblastoma tumor growth in vitro and in vivo, supporting KDM2B as a promising therapeutic target in these high-risk subgroups. Given that KDM2B demethylase activity is dispensable for tumor maintenance, catalytic inhibitors may not achieve clinical benefit. Instead, as supported by our dTAG studies, proteolysis targeting chimeras or molecular glues that specifically degrade KDM2B could be an attractive approach for achieving robust therapeutic efficacy. Additionally, integration of expression and DepMap dependency data^70–73^ showed that multiple PRC1.1 and PRC2 subunits are highly expressed in Group 3 and 4 medulloblastoma and represented Group 3 selective dependencies, supporting evaluation of PRC1 and PRC2 inhibitors as alternative approaches. Together, these findings suggest that the KDM2B–PRC1.1–PRC2 axis represents a targetable vulnerability in medulloblastoma.

In summary, through deep characterization of the medulloblastoma chromatin landscape across a large cohort of patient tumors, we provide fundamental insights into the epigenetic basis of subgroup-specific pathogenesis, and nominate KDM2B as an epigenetic driver warranting consideration as a rational target in high-risk medulloblastoma subgroups. The unique, high-quality, multidimensional molecular datasets generated for this study will serve as a valuable resource for future investigations into mechanisms governing gene regulation in cancer. We have made these freely accessible through an online interface that is facile to explore (https://proteinpaint.stjude.org/MBEpi).

## Methods

### Ethics and consent statements

This study involves no new patient sample collection, and informed and written consent exists for all patients contributing to the study. All animal experiments were conducted in accordance with the guidelines established by the National Institutes of Health’s Guide for the Care and Use of Laboratory Animals and approved by the St. Jude Children’s Research Hospital Institutional Animal Care and Use Committee (IACUC protocol 589–100536). St. Jude Children’s Research Hospital is registered as a research facility with the US Department of Agriculture and maintains an assurance statement with the Office of Laboratory Animal Welfare of the National Institutes of Health (Animal Assurance Number A3077-01).

### Animal usage

Mice were housed with a 12 h light–dark cycle set with lights on from 06:00 h to 18:00 h, with room temperature kept between 21 °C and 23 °C and humidity between 30 and 80%. Temperature and humidity were continuously controlled and monitored. For all in vivo studies, 8–12-week-old female NOD.Cg-*Prkdc*^scid^*Il2rg*^*tm1Wjl*^/SzJ (NSG) mice were used (The Jackson Laboratory, strain no. 005557). The exact number of animals used for each study is indicated in the figure legends. For in vivo dTAG treatment, the maximal tumor size or burden permitted by the IACUC is 20% of body weight, and this limit was not exceeded. All animal experiments adhered to Institutional guidelines in full.

### Cell lines or PDXs used in this study

D283 (HTB-185, ATCC), D425 (SCC290, MilliporeSigma), CHLA-01-MED (CRL-3021, ATCC) and Daoy (HTB-186, ATCC) are commercially available. Med113FH, Med1712FH, Med2312FH and Med1512FH were acquired from the Brain Tumor Resource Laboratory of Seattle Children’s Research Institute. RCMB20, RCMB28, RCMB37 and DMB006 were acquired from R. Wechsler-Reya. SJNBT12, SJNBT3, SJ-HGGX99c, SJ-HGGX77c, SJ-HGGX90c and SJ-DIPGX7 were derived from the St. Jude PDX core. The cell line and PDX identities were confirmed with DNA methylation profiling or short tandem repeat profiling.

### ChIP–seq

H3K27me3, H3K4me1, H3K4me3, H3K27ac, H3K9me3 and H3K36me3 ChIP was performed at ActiveMotif using antibodies against H3K27me3, H3K4me1, H3K4me3, H3K27ac, H3K9me3 and H3K36me3 (Supplementary Table 14). A subset of the H3K27ac (*n* = 23), H3K27me3 (*n* = 1) and H3K4me1 (*n* = 1) profiles were generated in a previous study^8^ and used directly. The remaining 229 ChIP–seq profiles were newly generated as part of this study (Supplementary Table 1). ChIP experiments for histone modification profiling and preparation of Illumina sequencing libraries were performed using the same methodology as described before^8^. Libraries were sequenced on the Illumina HiSeq 2000 platform using 2 × 101 cycles according to the manufacturer’s instructions. Alignment and downstream processing of ChIP–seq data was performed as previously described^74^. HM450 methylation arrays for each sample were classified using the MNP (v11) classifier to calculate medulloblastoma subgroup score and determine subgroup identity.

### Genomic coordinates and gene annotation

All coordinates used for ChIP–seq data in this study are based on human reference genome assembly hg19, GRCh37 (http://www.ncbi.nlm.nih.gov/assembly/2758/). Gene annotations are based on gencode annotation release 19 (https://www.gencodegenes.org/human/release_19.html), and the annotations that are ‘protein_coding’ were used in the analysis.

Multi-track representations for bigwig files were generated using the bamCoverage function of deepTools (v.3.5.0; RRID:SCR_016366)^75^. Track hubs were constituted for each medulloblastoma separately and visualized in the UCSC genome browser^76^.

### Chromatin segmentation

The Roadmap 18-state chromatin segmentation model^18^ was downloaded from http://egg2.wustl.edu/roadmap/data/byFileType/chromhmmSegmentations/ChmmModels/core_K27ac/jointModel/final (file named model_18_core_K27ac.txt). The downloaded model was applied for the chromatin segmentation of 52 primary medulloblastoma samples (Supplementary Table 1). In brief, initial bed files from both ChIP–seq samples and corresponding genomic DNAs were generated using the bamToBed command of bedtools (v.2.24.0)^77^. Resulting data in bed format were binarized (default 200 bp bin window) and subsequently segmented using ‘BinarizeBed’, ‘MakeSegmentation’ and ‘MakeBrowserFiles’ commands of the chromHMM tool (v.1.24; RRID:SCR_018141)^78^. Binarization was performed using default values, and genomic DNA controls were used as background in the identification of binarization thresholds. Missing histone mark values were imputed by randomly sampling reads in equal proportions from samples of the same medulloblastoma subgroup. The resulting simulated histone marks were incorporated into chromHMM like other samples.

### Differential chromatin state representation across medulloblastoma subgroups

Chromatin state classifications per 200 bp non-overlapping bins, as generated by ChromHMM, were filtered for blacklisted regions and normalized as a percentage of the remaining bins using the genome_info Python package (v.1.0.8; human reference, hg19). Chromatin states were compared between medulloblastoma subgroups by two-tailed Mann–Whitney *U*-tests (ggbeeswarm::geom_quasirandom v.0.7.2).

Differential enrichment analysis of the combined active chromatin states was performed as described in the Supplementary Note.

### Genomic enrichment of chromatin states

The hg19 version of the human genome was classified into exon, intron, intergenic and promoter (TSS ± 5 kb) regions by following the hierarchy of promoter > exon > intron > intergenic (https://github.com/kylessmith/hg19_info). Chromatin state bins within a medulloblastoma subgroup were each assigned to the genome class in which they occur and compared to 1,000 simulations of the same number of regions. Enrichment was determined as the log_2_ ratio of the observed occurrences to the mean of the simulations. All visualizations were generated using the Python matplotlib package (v.3.9.2).

### Characterization of EnhBiv and TssBiv in promoters

To examine the distribution of EnhBiv and TssBiv in promoters, chromatin segmentation bed files were generated from MakeBrowserFiles using the Epigenetics Roadmap 18-state model in ChromHMM (v.1.24; RRID:SCR_018141) for each state. Bed files were first combined by group and converted to bedGraph using genomeCoverageBed in bedtools (v.2.24.0; RRID:SCR_006646). All values were divided by sample number and converted to bigwig files. DeepTools (v.3.5.0, reference-point mode, ‘--skipZeros’ disabled; RRID:SCR_016366) was used to generate profiles at the promoter (TSS ± 5 kb) of all protein-coding genes. The values represent the percentage of chromatin state at each bp across all promoters of protein-coding genes in each medulloblastoma subgroup. DNA methylation, GC content and CpG density at the EnhBiv and TssBiv marked regions were determined as described in the Supplementary Note.

### Identification of Group 4 EnhBiv and Group 3 ReprPC signature genes

First, 200 bp genomic bins with an EnhBiv state for at least half of Group 4 medulloblastoma samples were identified. The resulting regions were merged, and for each of the merged regions, the number of 200 bp bins with an EnhBiv state was identified for each medulloblastoma separately. Afterward, each merged region was tested for the differential representation of EnhBiv state using a Mann–Whitney *U*-test in a one-versus-rest format. The regions with false discovery rate (FDR)-corrected *P* < 0.05 were retained and referred to as Group 4 EnhBivs. Group 3 ReprPCs were identified using the same method.

Promoter clusters were determined using *k*-means clustering of the 200 bp chromatin states within Group 4 EnhBiv-enriched promoters, as implemented in SciPy (v.1.13.0). Cluster number was determined by identifying the elbow of the gap statistic.

To identify regulators enriched in Group 4 EnhBiv-enriched promoters, GSEA was implemented using the Python package GSEApy (v.1.1.3), with the ChEA_regulator-target associations derived from published ChIP–seq data, filtered to only include human-relevant gene sets. Significance was determined using a two-tailed hypergeometric test and FDR correction.

### Functional annotation and pathway enrichment

Functional annotation and pathway enrichment analysis for Group 4 EnhBiv signature genes was performed using the Database for Annotation, Visualization and Integrated Discovery (DAVID; v.6.8)^79,80^. Functional annotation clustering analysis was performed using Gene Ontology biological process terms, which group related annotation terms based on the degree of similarity in their gene membership using a modified kappa statistic^80^. *P* values were determined by modified Fisher’s exact test (EASE score) with a Benjamini–Hochberg correction for multiple testing. Annotation clusters were ranked by enrichment score, which represents the geometric mean of the EASE scores for all enriched Gene Ontology biological process terms within each cluster, providing a measure of overall cluster significance. The top ten annotation clusters with the highest enrichment scores from the DAVID functional annotation clustering output were selected for visualization.

For network visualization, the associated gene lists were extracted from each Gene Ontology biological process term within the top ten clusters. Jaccard similarity between all term pairs was calculated, and terms with a Jaccard similarity of >0.5 were connected by edges in the network, representing functionally related pathways sharing substantial gene overlap (>50% similarity). Edge thickness in the network visualization was proportional to Jaccard similarity, with thicker edges indicating higher similarity. Node sizes were scaled according to the *P*_adj_, with larger nodes representing more significantly enriched terms. Network files (node and edge tables) were imported into Cytoscape (v.3.9)^81^ for visualization.

Additional functional enrichment analysis was performed using Enrichr^34^ (https://maayanlab.cloud/Enrichr). The Group 4 EnhBiv signature gene list was submitted to Enrichr and analyzed against the Gene Ontology biological processes 2025 gene set library. Enrichment *P* values were calculated using two-sided Fisher’s exact test and adjusted for multiple testing using the Benjamini–Hochberg FDR correction. Enriched terms were ranked using Enrichr’s combined score metric, which integrates the *z*-score computed by assessing the deviation from the expected rank with Fisher’s exact test *P* value.

### Gene expression analysis

Differential expression analysis across medulloblastoma subgroups was conducted using 167 RNA-seq samples from a publicly available dataset^6^, which includes all 48 samples with available RNA-seq data from the current cohort. The DESeq2 package (v.1.40.2)^82^ in R (v.4.3) was used for the analysis. Normalization of the count matrix across subgroups was performed using the median-of-ratios method. Subsequent statistical comparisons between subgroups or subtypes were performed pairwise using the two-sided Wald test, with *P* values adjusted using the Benjamini–Hochberg method. Adjustments to log_2_(fold changes) between subgroups were made with the lfcShrink function. Genes exhibiting *P*_adj_ < 0.05 and absolute log_2_(fold change) > 1 were identified as differentially expressed. To compare individual gene expression across samples from different subgroups, each gene was normalized using the variance-stabilizing transformation method across all samples. Statistical significance for differential gene expression between samples from every two subgroups was assessed using the t_test function from the rstatix package (v.0.7.2) in R, with *P* values adjusted by the Holm method. For differential expression analysis of Group 4 EnhBiv signature genes and Group 3 ReprPC signature genes in medulloblastoma subgroups, median expression of the gene set was calculated for each sample and then compared among subgroups. Subgroup-specific statistical tests were conducted using the two-tailed Wilcoxon test, with *P* < 0.05 considered to be significant. ggplot2 (v.3.4.0) was used for plotting.

Correlation of *KDM2B* expression with genetic alterations in medulloblastoma and analysis of *KDM2B* expression during human cerebellum development were performed as described in the Supplementary Note.

### Examining growth dependency using DepMap CRISPR screen dataset

To analyze chromatin complex dependency, Chronos gene dependency scores for 1,190 cancer cell models were obtained from DepMap (v.26Q3; depmap.org)^70,72,73^. Analyses were performed as described in the Supplementary Note.

### CRISPR targeting of *KDM2B*

Two *KDM2B* gRNAs, g10 and g28, were designed to target the CXXC DNA-binding domain. *RPA3* gRNA was used as a positive control. Non-targeting gRNA with no predicted target site in the genome or *AAVS1* gRNA targeting the safe harbor locus were used as negative controls. gRNA sequences designed in this study are listed in Supplementary Table 14. These gRNAs were cloned into the all-in-one LentiCRISPRv2GFP lentiviral vector^83^. Editing efficiency at the *KDM2B* locus was evaluated by targeted deep sequencing of genomic DNA extracted from transduced D283 cells. Editing efficiency was determined by normalizing the proportion of edited cells with transduction efficiency. To examine *KDM2B* depletion at the protein level, KDM2B-dTAG D283 cells were transduced with viruses encoding *KDM2B* gRNAs or non-targeting control gRNA and cultured for 4 or 7 days. HA-Tag antibody was used to detect the endogenous KDM2B–HA-dTAG fusion protein by immunoblotting.

### dTAG tagging of endogenous *KDM2B* and induction of KDM2B degradation

The D283 KDM2B C-terminus HA-dTAG cell line was generated using CRISPR–Cas9 technology in the Center for Advanced Genome Engineering (RRID:SCR_027027) as described in the Supplementary Note.

For in vitro studies, D283 KDM2B-dTAG cells were treated with either dTAG^V^-1 or dTAG13 (Tocris Bioscience), which showed similar efficiency and kinetics for inducing KDM2B degradation. For the in vivo tumor growth assay, dTAG^V^-1 was used to induce KDM2B degradation.

### Ectopic expression of wild-type or mutant *KDM2B*

*KDM2B* long isoform was amplified from GFP-FBXL10 (ref. ^84^) (a gift from M. Pagano; Addgene plasmid 126542) by PCR and then cloned into a Tet-On all-in-one lentiviral vector pINDUCER-21-HOXA9 (ref. ^85^) (a gift from G. Daley; Addgene plasmid 97041) with an HA tag at the C-terminus to replace the original HOXA9 gene. KDM2B mutants were generated by deleting the CXXC DNA-binding domain (CXXC del) or JmjC domain (JmjC del) or introducing H242A/I243A/D244A mutations to the JmjC domain to abolish demethylase activity (JmjC mut)^86^.

To generate cell lines stably integrated with these constructs, D283 KDM2B–HA-dTAG cells were transduced with empty vector or Tet-On *KDM2B* lentiviruses and then sorted using flow cytometry to enrich for transduced cells (GFP^+^). To compare the level of ectopically expressed wild-type or mutant KDM2B with endogenous KDM2B, cells were treated with 20 ng ml^−1^ (wild-type KDM2B, JmjC mut and CXXC del) or 40 ng ml^−1^ (JmjC del) Dox in the presence or absence of 500 nM dTAG for 24 h and then collected for immunoblotting. HA-Tag antibody was used to detect both ectopically expressed and endogenous KDM2B.

### Examining gene expression changes following KDM2B perturbation in D283 or DMB006 cells

For RNA-seq in Group 3 D283 cells following KDM2B degradation, KDM2B-dTAG cells transduced with lentiviruses encoding an empty vector or Dox-inducible *KDM2B* constructs were treated in triplicate with 20 ng ml^−1^ (wild-type KDM2B, JmjC mut and CXXC del) or 40 ng ml^−1^ (JmjC del) Dox in the presence or absence of 500 nM dTAG for 5 days before collection for RNA extraction. Culture media was replaced every 2 days.

For RNA-seq in Group 3 D283 cells following *KDM2B* knockout, wild-type D283 cells were transduced in triplicate with lentivirus encoding either non-targeting control gRNAs or *KDM2B*-targeting gRNAs at a high multiplicity of infection (MOI) to achieve high transduction efficiency (>85%). Cells were cultured for 7 days before collection for RNA extraction. Infection efficiency was determined by flow cytometry analysis of GFP percentage on day 4. Editing efficiency was evaluated by targeted deep sequencing.

For RNA-seq in Group 4 DMB006 PDX, freshly isolated tumor cells were transduced in triplicate with lentivirus encoding either non-targeting control gRNAs or *KDM2B*-targeting gRNAs at a high MOI to achieve high transduction efficiency (>90%). Cells were cultured for 7 days before collection for RNA extraction. Infection efficiency was determined by flow cytometry analysis of GFP percentage on day 4. Editing efficiency was evaluated by targeted deep sequencing.

RNA sequencing and data analysis followed by GSEA were performed as described in the Supplementary Note.

### CUT&RUN in medulloblastoma PDXs or D283 cell line

For CUT&RUN in medulloblastoma PDXs, tumor cells were freshly isolated from tumor-bearing mice, cryopreserved in 10% dimethyl sulfoxide and stored in liquid nitrogen. Before use, cells were thawed, and cell viability was determined using an automated cell counter (Countess, Thermo Fisher).

For CUT&RUN of KDM2B, BCOR, EZH2 and H2AK119ub following KDM2B degradation, Group 3 D283 KDM2B-dTAG cells were treated with either dimethyl sulfoxide (control) or 500 nM dTAG for the indicated times and collected. For CUT&RUN of other histone modifications, cells were treated for 5 days before collection. Culture media was replaced every 2 days.

For CUT&RUN in D283 cells following *KDM2B* knockout, wild-type D283 cells were transduced with lentivirus encoding either non-targeting control gRNAs or *KDM2B*-targeting gRNAs at a high MOI to achieve a high transduction efficiency (>90%). Cells were cultured for 7 days before collection.

For CUT&RUN following EZH2 inhibitor treatment, D283 cells were treated with 5 μM tazemetostat for 5 days before collection.

Details of the CUT&RUN procedure, data processing and visualization are described in the Supplementary Note. Antibodies are described in Supplementary Table 14.

To determine the chromatin states using CUT&RUN data, ChromHMM models with four to eight states were trained using H3K4me1, H3K4me3 and H3K27me3 from primary tumor-derived ChIP–seq data. The seven-state model best recapitulated the EnhBiv state and was applied to CUT&RUN data.

### Characterization of EnhBiv-enriched or TssBiv-enriched promoters in medulloblastoma patient samples or PDXs

The mean percentage of the EnhBiv or TssBiv state was calculated for each promoter (TSS ± 5 kb) by subgroup based on chromatin segmentation bed files from MakeBrowserFiles in ChromHMM (v.1.24; RRID:SCR_018141) using the Epigenetics Roadmap 18-state model (for patient ChIP–seq data) or seven-state model (for PDX CUT&RUN data). Promoters enriched with corresponding states were defined using a >10% cutoff for medulloblastoma PDXs or a >20% cutoff forpatient with medulloblastoma samples.

To compare mark enrichment at EnhBiv-labeled promoters across medulloblastoma PDX subgroups, mark intensity from each promoter was extracted by bigWigAverageOverBed (from UCSC tools https://github.com/ENCODE-DCC/kentUtils) from bigwig data averaged across subgroups. Violin plots were made with ggpubr (v.0.4.0; RRID:SCR_021139). *P* values were determined by a two-tailed Wilcoxon test.

### In vitro competition assay

Cells were transduced with lentiviruses encoding non-targeting control, *KDM2B*, *RPA3* or *AAVS1* gRNA with a 40–60% transduction efficiency. At 3–4 days after infection, an aliquot of cells was collected to examine baseline GFP percentage by flow cytometry. Cells were cultured in triplicate for 2–4 weeks, with cell aliquots collected at regular intervals to determine GFP percentage. GFP percentage at each time point was normalized to the baseline GFP percentage for each sample. Experiments were independently repeated two to three times with similar results. The number of biological replicates for each experiment is provided in the corresponding figure legends. To compare *KDM2B* dependency across SHH, Group 3, Group 4 and high-grade glioma models, the normalized GFP proportion of *KDM2B*-knockout cells at the final time point was calculated relative to the corresponding non-targeting control for each model. Statistical significance was assessed by one-way ANOVA followed by Tukey’s multiple-comparison test, using GraphPad Prism (v.10).

### In vivo competition assay

DMB006 or Med2312FH cells were transduced with non-targeting control or *KDM2B* gRNA viruses overnight. The following day, an aliquot of cells was collected and implanted orthotopically into the cerebellum of NSG mice. The remaining cells were cultured for another 2–3 days before collecting for flow cytometry to determine baseline GFP percentage for each sample. When mice reached the morbidity endpoint, tumors were dissected to determine GFP percentage. Endpoint tumor GFP percentage was normalized to baseline. Statistical significance was determined by a two-tailed *t*-test using GraphPad Prism (v.10).

### In vitro dTAG competition assay

Parental or KDM2B-dTAG D283 cells were mixed with mCherry^+^ D283 and treated with 0, 100 or 500 nM dTAG in duplicate. Cell aliquots were collected at days 0, 4, 8 and 12 for flow cytometry analysis to determine the percentage of mCherry^−^ cells. For data analysis, the percentage of mCherry^−^ cells of dTAG-treated samples at each time point were normalized to the day 0 value of the same sample and also normalized to untreated cells at the same time points to correct for different growth rates of parental and KDM2B-dTAG cell lines. The experiment was independently repeated three times with similar results.

### Rescue competition assay

Wild-type D283 cells were mixed with D283 KDM2B-dTAG cells stably integrated with a Tet-On empty vector, wild-type KDM2B or mutant KDM2B at a ratio of 2:3. Cells were treated with 20 ng ml^−1^ (wild-type KDM2B, JmjC mut and CXXC del) or 40 ng ml^−1^ (JmjC del) Dox in the presence or absence of 500 nM dTAG for 12 days. Cell aliquots were collected at days 0, 4, 8 and 12 for flow cytometry analysis to determine the percentage of GFP^+^ cells. The percentage of GFP^+^ cells at each time point were normalized to the day 0 value of the same sample. The experiment was independently repeated twice with similar results.

### In vitro growth assay

Wild-type or KDM2B-dTAG D283 cells were treated with 0, 100 or 500 nM dTAG in triplicate. At each time point, 40 µl cell aliquots from each well (1 ml total volume) were used to determine cell viability using the CellTiter-Glo assay (Promega). An additional aliquot (one-tenth of the sample) from each well was cultured in fresh medium containing the indicated concentration of dTAG. For data analysis, CellTiter-Glo readings at each time point were multiplied by dilution factors determined from each prior subculture and then normalized to the day 0 reading of the same sample. The experiment was independently repeated twice with similar results.

Cell fate changes following KDM2B degradation were assessed by proliferation or apoptosis assays as described in the Supplementary Note.

### In vivo dTAG treatment

D283 KDM2B-dTAG cells transduced with a YFP-luciferase reporter were implanted into the flank of NSG mice. Then, 10 days after implantation, mice were ranked by luciferase signal and evenly assigned to control and treatment groups (*n* = 13 mice per group). Mice were dosed with vehicle or 40 mg kg^−1^ of dTAG^V^-1 from Monday to Friday for 7 weeks. Dimensions of the flank tumors were measured with a caliper. Survival was defined as the time until tumors reached 10% of body weight or mice reached the morbidity endpoint. Statistical significance for tumor size was determined by two-way ANOVA, and survival difference was assessed by a log-rank Mantel–Cox test using GraphPad Prism (v.10). Histopathology and immunohistochemistry characterization of flank tumors were performed as described in the Supplementary Note.

### Co-immunoprecipitation and immunoblotting

For co-immunoprecipitation to examine the interaction between KDM2B and PRC1.1 or PRC2 subunits in DMB006, freshly isolated DMB006 cells were collected for nuclear extraction and immunoprecipitation as described in the Supplementary Note. KDM2B antibody was used to pull down KDM2B protein with normal rabbit IgG antibodies as a control (Supplementary Table 14).

For immunoprecipitation to examine the interaction between KDM2B and PRC1.1 or PRC2 subunits in D283, wild-type D283 (control) or D283 KDM2B-dTAG cells were collected for nuclear extraction and immunoprecipitation as described in the Supplementary Note. HA-Tag antibody was used to pull down KDM2B–HA-dTAG protein.

For immunoprecipitation to examine the interaction between KDM2B mutants and PRC1.1 subunits, D283 KDM2B-dTAG cells stably expressing a Tet-On empty vector, wild-type or mutant KDM2B were treated with Dox in the presence of 500 nM dTAG for 24 h and collected for nuclear extraction and immunoprecipitation as described in the Supplementary Note. HA-Tag antibody was used to pull down KDM2B–HA-dTAG protein.

Immunoblotting was performed as described in the Supplementary Note. Antibodies are described in Supplementary Table 14.

### Characterization of histone modification changes following *KDM2B* knockout by mass spectrometry

D283 cells were transduced in triplicate with lentivirus encoding either non-targeting control gRNAs or *KDM2B*-targeting gRNAs at a high MOI to achieve high transduction efficiency (>80%). Cells were cultured for 6 days before collection. Histone mass spectrometry was performed as described in the Supplementary Note.

### Statistics and reproducibility

Details of quantification and statistical analyses for specific experiments are provided in the Methods. Sample sizes (*n*), definitions of biological and technical replicates and statistical tests used are indicated in the figure legends. Statistical analyses were performed using R, Python, GraphPad Prism and Excel. Experiments were independently repeated a minimum of two times, with similar results observed across biological replicates. No statistical method was used to predetermine sample size. No data were excluded from the analyses. The experiments were not randomized. For in vivo dTAG treatment using the flank tumor model, mice were ranked by tumor signal and assigned to treatment groups evenly, ensuring equal representation of tumor size between groups. The investigators were blinded to group identity during tumor size measurements and outcome assessments. Immunohistochemistry image quantification of tumor samples was performed in a blinded manner by imaging scientists using QuPath.

### Reporting summary

Further information on research design is available in the Nature Portfolio Reporting Summary linked to this article.

## Online content

Any methods, additional references, Nature Portfolio reporting summaries, source data, extended data, supplementary information, acknowledgements, peer review information; details of author contributions and competing interests; and statements of data and code availability are available at https://doi.org/10.1038/s41588-026-02745-1.

## Supplementary information

Supplementary Information Supplementary Methods

Reporting Summary

Peer Review File

Supplementary Table Supplementary Excel file with Tables 1–14 as separate tabs.

## Source data

Source Data Fig. 4 Uncropped western blot for Fig. 4j.

Source Data Fig. 6 Uncropped western blots for Fig. 6i.

Source Data Fig. 7 Uncropped western blots for Figs. 7b,c.

Source Data Extended Data Fig. 6 Uncropped western blots for Extended Data Fig. 6b.

Source Data Extended Data Fig. 8 Uncropped western blots for Extended Data Fig. 8h.

Source Data Extended Data Fig. 9 Uncropped western blots for Extended Data Fig. 9h.

Source Data Source data for Figs. 4b,c,d,f,g,h,l,n,o,q, 5g, 7d and Extended Data Fig. 6c,d,e,f,g,h,i,j,k,l,m,n,o.

## Data Availability

Patient ChIP–seq and RNA-seq data have been deposited at the European Genome-Phenome Archive (EGA; http://www.ebi.ac.uk/ega/) hosted by the EBI, under accession number EGAS00001000215; whole-genome sequencing, expression microarray and DNA methylation array data under accession number EGAS00001001953; and whole-genome bisulfite sequencing data under accession number EGAS00001000561 (refs. ^6,8,87^). RNA-seq and CUT&RUN data for medulloblastoma cell lines or PDXs were deposited in the Gene Expression Omnibus under accession codes GSE269909 (RNA-seq) and GSE270125 (CUT&RUN). Bulk *KDM2B* expression values normalized as transcripts per million across developmental stages were downloaded from https://www.brainspan.org/static/download.html (ref. ^88^). The mass spectrometry proteomics data have been deposited to the ProteomeXchange Consortium via the MassIVE partner repository, with dataset identifier PXD079139. The single-cell cerebellum data^40^ can be downloaded from https://apps.kaessmannlab.org/sc-cerebellum-transcriptome. DepMap CRISPR dependency data^72,73^ (v.26Q3) can be downloaded from the DepMap portal (https://depmap.org/portal/achilles). Plasmids and cell lines are available upon request. Source data are provided with this paper.

## Code availability

The methods contain all software packages with references, parameters and version numbers needed to replicate the study. Details of the pipeline used and codes for customized plots can be found at https://doi.org/10.6084/m9.figshare.c.8552007 (ref. ^89^).
