# Supplementary material for: Divergent medulloblastoma chromatin states disclose KDM2B as a selective dependency
Source: Nat Genet. 2026 Sep 24;58(10):2590–604. doi: 10.1038/s41588-026-02745-1 (PMC13649585; doi:10.1038/s41588-026-02745-1)
Supplement: Supplementary file 1 [file 41588_2026_2745_MOESM10_ESM.pdf]

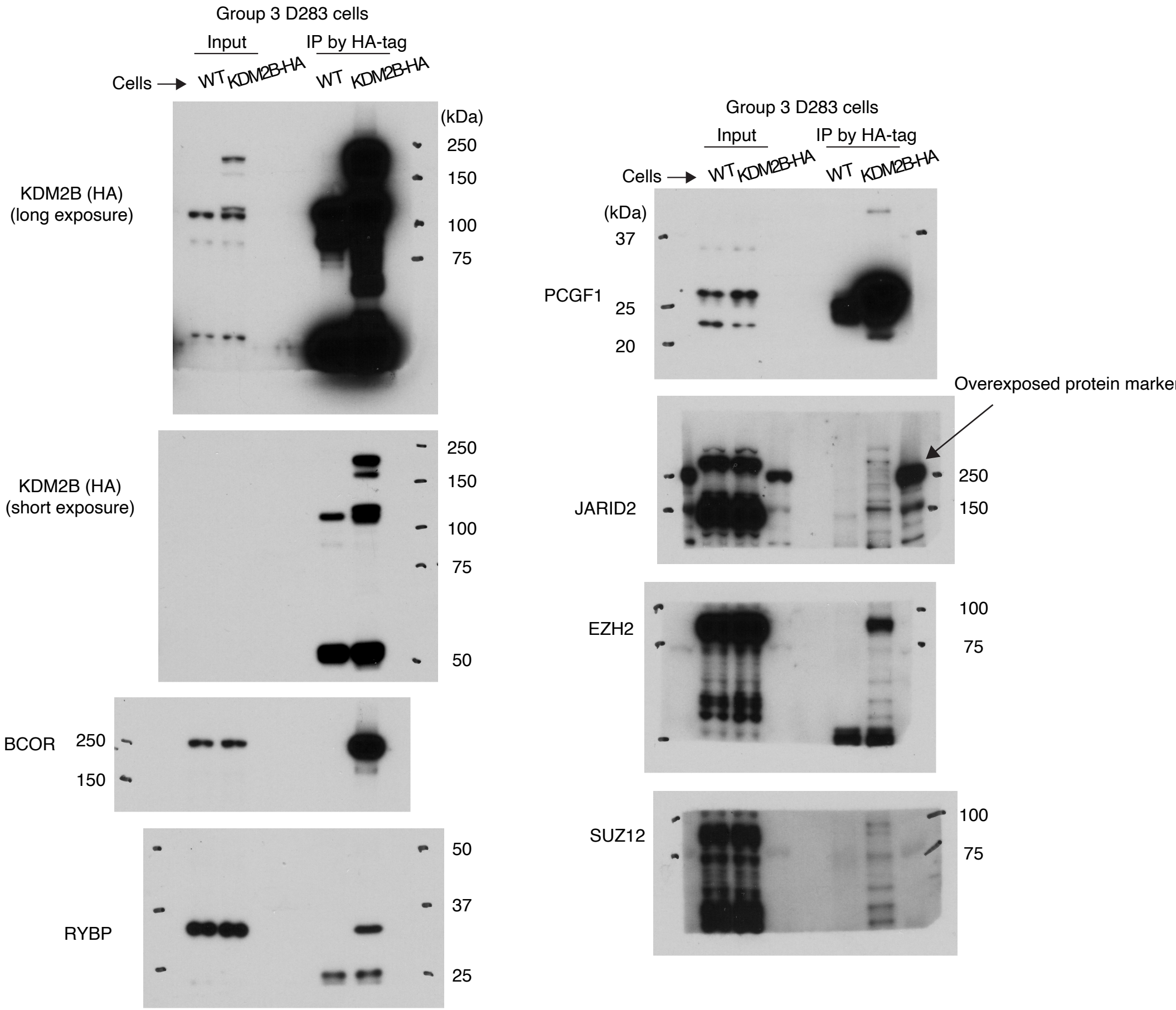

Group 3 D283 cells
Input
IP by HA-tag
Cells →
WT
KDM2B-HA
WT
KDM2B-HA
(kDa)
250
150
100
75
KDM2B (HA) (long exposure)
KDM2B (HA) (short exposure)
250
150
100
75
50
BCOR
250
150
RYBP
50
37
25
Group 3 D283 cells
Input
IP by HA-tag
Cells →
WT
KDM2B-HA
WT
KDM2B-HA
(kDa)
37
25
20
PCGF1
Overexposed protein marker
JARID2
250
150
EZH2
100
75
SUZ12
100
75
