# Supplementary material for: Divergent medulloblastoma chromatin states disclose KDM2B as a selective dependency
Source: Nat Genet. 2026 Sep 24;58(10):2590–604. doi: 10.1038/s41588-026-02745-1 (PMC13649585; doi:10.1038/s41588-026-02745-1)
Supplement: Supplementary file 2 [file 41588_2026_2745_MOESM1_ESM.pdf]

nature genetics

Supplementary information https://doi.org/10.1038/s41588-026-02745-1

Divergent medulloblastoma chromatin states disclose KDM2B as a selective dependency

In the format provided by the authors and unedited

Differential enrichment analysis of active chromatin states comparing SHH, Group 3, and Group 4

11 active states defined by the 18-state model were merged into a single active chromatin state. Enrichment was calculated as percentage active state across transcriptional start site (TSS) ± 5kb promoter region annotated by GENECODE. One representative isoform for each gene (n = 18,741) was selected based on TSS closest to the strongest H3K4me3 peak. Average enrichment across replicates for each subgroup were normalized among subgroups so that the sum of three values was 100% (Supplementary Table 3) before plotting as a triangle plot with ggtern (v3.4.2) in R (v4.3.3). Red, yellow, and green lines indicate relative enrichment scores for SHH, Group 3, and Group 4, respectively. Dots with scores >80 in one subgroup and <20 in the other two subgroups represent genes exhibiting a subgroup-specific active state. Dots with scores of 40–60 in two subgroups and <20 in the remaining subgroup represent genes with a shared active state across two subgroups. Dot color indicates statistical significance of differences between SHH, Group 3, and Group 4 determined by one-way ANOVA test using scipy (v1.9.3, stats.f_oneway, RRID:SCR_008058). Genes with $P_{adj} < 1 \times 10^{-5}$ were set to 1×10^{-5} for better visualization.

DNA methylation, GC content, and CpG density at EnhBiv and TssBiv marked regions

Average DNA methylation (%), GC content (%) and CpG observed/expected ratio were computed for each genomic interval corresponding to TssBiv or EnhBiv states in promoter (TSS ± 5kb) based on gencode annotations for each gene.

Genome-wide CpG methylation beta values for twelve Group 4 samples were obtained from previously published whole-genome bisulfite sequencing (WGBS) data[1]. For each state interval and each sample, the mean sample-level beta value was calculated across all CpGs overlapping the interval. For a given sample and interval, CpGs were considered detected if the methylation beta value for the given sample and CpG was available. If fewer than three CpGs were detected within the interval, the sample-level beta value was set to missing for that sample-interval combination. The interval-level methylation value was calculated as the average of all sample-level beta values for the given interval. Intervals with missing sample-level beta values in more than three samples were excluded from further analysis. Additionally, intervals with fewer than three CpGs overlapping the interval were excluded from further analyses.

Nucleotide counts for each state interval were computed using the nuc command of bedtools (v2.25.0)[2]. These nucleotide counts were used to compute per-interval GC content (%) - percentage of G and C nucleotides; observed CpG content – fraction of CpG dinucleotides; and expected CpG content = $(\#C \cdot \#G)/L$, where #C is the number of C nucleotides in the sequence, #G is the number of G nucleotides in the sequence and L is the length of the sequence. Finally, distributions of average DNA methylation (%),

GC content (%) and CpG observed/expected ratio were compared between intervals belonging to TssBiv and EnhBiv states using a two-tailed Mann–Whitney U test with $P < 0.05$ considered statistically significant.

Correlation of *KDM2B* expression with genetic alterations in medulloblastoma

Annotations describing medulloblastoma subgroup and subtype, chromosome arm copy number alterations, and gene alterations were obtained from a previous study[3]. These annotations were derived from whole genome sequencing, Affymetrix expression microarray, and Illumina 450 k methylation array data. For downstream analyses, all annotations were binarized to indicate the presence (annotation-positive samples) or absence (annotation-negative samples) of a given feature in each tumor sample. Annotations with fewer than 3 annotation-positive samples were excluded from the analysis.

Group 3 (n = 56) and Group 4 (n = 91) primary medulloblastoma samples were sorted in ascending order by normalized *KDM2B* expression. For each binarized annotation, the observed mean of *KDM2B* expression among annotation-positive samples was compared to a theoretical null distribution. The null distribution was created by randomly permuting annotation-positive sample labels and recalculating the mean *KDM2B* expression (n = 100,000 permutations). The effect size was calculated by taking the difference between the observed mean *KDM2B* expression of annotation-positive samples and the mean of the null distribution. Two-sided *P* values were computed as the fraction of permutations yielding an effect size at least as extreme as the observed mean *KDM2B* expression. $P < 0.05$ was considered statistically significant.

Analysis of *KDM2B* expression during human cerebellum development

Normalized human developmental cerebellar bulk RNA-seq expression data with values in Reads Per Kilobase of transcript per Million mapped reads (RPKM) were downloaded from the BrainSpan Atlas[4]. Samples annotated as “cerebellar cortex” (n = 29) and “cerebellum” (n = 3) were included. Developmental stages were grouped based on annotated ages as follows: 8–13 post-conception weeks (pcw; n = 4), 16–21 pcw (n = 4), 24–37 pcw (n = 3), infant (< 3 years; n = 5), child (3 - 15 years; n = 8), and adult (> 16 years; n = 8). Statistical differences across developmental groups were assessed using the t_test function (two-sided) from the rstatix package (v0.7.2) in R, with *P* adjusted using the Holm method.

To examine *KDM2B* expression in human rhombic lip-derived lineages, single-nuclei RNA sequencing data from the developing human cerebellum[5] were subsetted to include only rhombic lip-derived populations based on published annotations. Dimensionality reduction was performed using principal component analysis (50 components), followed by k-nearest neighbor graph construction (n_neighbors = 50, n_pcs = 30) and diffusion map embedding using Scanpy (v1.9.3)[6]. *KDM2B* expression

was extracted from the log1p-normalized expression matrix and visualized across developmental states. For dot plots, dot size represents the percentage of cells expressing *KDM2B* (expression > 0 in log1p-normalized counts) and dot color indicates mean expression level within each cell population. Statistical comparisons between adjacent stages within each lineage were performed using two-tailed the Mann-Whitney U test, with significance set at $P < 0.05$.

Examining growth dependency using DepMap CRISPR screen dataset

For analyzing chromatin complex dependency, Chronos gene dependency scores for 1,190 cancer cell models were obtained from DepMap (v26Q3, depmap.org)[7-9]. Chromatin-modifying complexes directly or indirectly regulating H3K4 and H3K27 methylation were selected: PRC1.1, PRC1.2; PRC1.3, PRC1.4, PRC1.5, PRC1.6; PRC2.1, PRC2.2, COMPASS-SETA/B, COMPASS-KMT2A/B, COMPASS-KMT2C/D, and CoREST. Complex membership was annotated using most recent release (5.2) of the Comprehensive Resource of Mammalian Protein Complexes (CORUM) database[10] (https://mips.helmholtz-muenchen.de/corum/) and refined by manual curation using published literature. For complexes represented by multiple CORUM entries, the original publications referenced in CORUM entries were manually reviewed to assess whether distinct complex assemblies were experimentally supported. Where the cited literature did not explicitly differentiate between sub-complex variants (e.g., PRC1.1a–d), CORUM entries were consolidated into a single complex annotation reflecting the evidence presented in the primary source. Gene membership for each chromatin modifying complex is shown in Supplementary Table 13.

For each complex, member genes were treated as a gene set. Then, single-sample gene set enrichment analysis (ssGSEA) was performed. Chronos dependency scores were used for gene ranking within each cell model. ssGSEA was implemented in Python with gseapy (v1.1.0) tool with the "rank" sample normalization method. Normalized enrichment scores (NES) were compared between Group 3-medulloblastoma cell lines (D283, D341, D425, D458, MB002, HDMB03, SJMB016880TO[11], and MED411FH; n = 8) and all other cell lines (n = 1,182) using a two-tailed Mann-Whitney U test in Python using scipy (v1.15.1, stats. mannwhitneyu). A lower NES value corresponds to a stronger dependency. Statistical significance was defined as $P < 0.05$.

For assessing Group 3 selective dependency of individual PRC1 and PRC2 complex members, median gene effect was calculated per group, and statistical significance was determined using a two-tailed Mann-Whitney U test in Python using scipy (v1.15.1, stats. mannwhitneyu) comparing dependency scores between Group 3-medulloblastoma cell lines (n = 8) and all other cell lines (n = 1,182) for each complex member.

dTAG tagging of endogenous *KDM2B*

1 × 10^6 D283 cells were transiently transfected (4D-Nucleofector™ X-unit, Lonza) with precomplexed ribonuclear proteins (RNPs) consisting of 100 pmol of chemically modified sgRNA (Integrated DNA Technologies), 33 pmol of 3 × NLS SpCas9 protein (St. Jude Protein Production Core), and 250 ng of plasmid donor in a 20mL cuvette using solution SF and program CA-137 according to the manufacturer's recommended protocol. Single cells were sorted five days post nucleofection at the Flow Cytometry and Cell Sorting Shared Resource (St. Jude) and plated into prewarmed (37°C) media into 96-well plates. Clones with desired modification were identified via targeted deep sequencing on a Miseq Illumina sequencer as previously described[12,13]. Cell identity was authenticated using the PowerPlex® Fusion System (Promega) performed at the Hartwell Center (St. Jude), and cells were tested negative for mycoplasma by the MycoAlert™Plus Mycoplasma Detection Kit (Lonza). Editing construct sequences and relevant primers are listed in Supplementary Table 14.

***In vitro* proliferation or apoptosis assay**

D283 KDM2B-dTAG cells were treated with 0 or 500 nM dTAG in triplicate for 9 days. At each time point, a cell aliquot from each well was cultured with fresh medium with or without dTAG. For the proliferation assay, the remaining cells were treated with 10 mM EdU for 30 min and stained with Click-iT™ Plus EdU Pacific Blue™ Flow Cytometry Assay Kit (C10636, Thermo Fisher). For the apoptosis assay, the remaining cells were stained with the PE Annexin V Apoptosis Detection Kit with 7-AAD (640934, Biolegend). Percentage of EdU+ or Annexin V+ cells was determined by flow cytometry. Experiments were independently repeated twice with similar results.

RNA sequencing and data analysis

Paired-end 100-cycle sequencing was performed on Illumina NovaSeq sequencers, per the manufacturer's directions (Illumina). Reads were first trimmed for adapters using trim_galore (parameters: "--paired --retain_unpaired") and then aligned to the reference genome hg38 (Gencode GRCh38.p12) by STAR (v2.7.1a, parameters: "--outFilterMultimapNmax 20 --alignSJoverhangMin 8 --alignSJstitchMismatchNmax 5 -1 5 5 --alignSJDBoverhangMin 10 --outFilterMismatchNmax 999 --outFilterMismatchNoverReadLmax 0.04 --alignIntronMin 20 --alignIntronMax 100000 --alignMatesGapMax 100000 --outSAMmapqUnique 60 --outSAMmultNmax 1 --outSAMstrandField intronMotif --outSAMattributes NH HI AS nM NM MD --outSAMunmapped Within --chimSegmentMin 12 --chimJunctionOverhangMin 12 --chimSegmentReadGapMax 3 --chimMultimapNmax 10 --chimMultimapScoreRange 10 --chimNonchimScoreDropMin 10 --chimOutJunctionFormat 1 --quantMode TranscriptomeSAM GeneCounts --twopassMode Basic --peOverlapNbasesMin 12 --peOverlapMMp 0.1")[14]. Gene level quantification was determined using RSEM (v1.3.2)[15] based on GENCODE (v31) gene

annotation. After normalization by trimmed mean of M-values normalization, Voom[16] was used to identify differentially expressed genes assessed using linear models with empirical Bayes moderation of standard errors. FDR corrected $P < 0.05$ and fold change > 2 were used as the cut off for differentially expressed genes.

Gene Set Enrichment Analysis (GSEA)

GSEA was performed using clusterProfiler (v4.10.0)[17]. Genes were ranked by log2 fold-change from differential expression analyses. For pathway enrichment, the MSigDB C2 canonical pathways collection was filtered to include KEGG, REACTOME, WikiPathways, and Gene Ontology sources, with gene set sizes restricted to 50-400 genes. Pathways were considered significantly enriched if two-sided permutation-test with Benjamini-Hochberg FDR adjusted $P < 0.05$ and |NES| > 1.5. Relationships between significantly enriched pathways were visualized using network analysis based on Jaccard similarity. Pathway pairs with Jaccard similarity ≥ 0.1 were connected by edges. Networks were visualized in Cytoscape (v3.9).

For targeted gene set analyses (Group 4 EnhBiv signature genes, Group 4 EnhBiv-marked neurogenesis genes, PRC2 targets, neural progenitor cell (NPC) bivalent state-marked genes, and central nervous system (CNS) neuronal differentiation genes), GSEA was conducted using clusterProfiler (v4.10.0) with genes ranked by $\log_2$ fold-change. Enrichment scores were calculated using a weighted Kolmogorov-Smirnov statistic, and statistical significance was determined by two-sided permutation testing (1,000 permutations) with Benjamini-Hochberg FDR correction. Enrichment plots display the running enrichment score across the ranked gene list, with vertical dashed lines indicating maximum enrichment.

For enrichment analysis of cell proliferation, neuronal differentiation, apoptosis and senescence gene sets following KDM2B degradation in D283 cells, enriched pathways across these biological themes were identified and curated from MSigDBv2023 (C2 and C5) with size filters of 20-500 genes using keyword-based pattern matching (proliferation = regex("cell.cycle|mitotic|\\bs.phase\\b|\\bg2.m|\\bg1.s|\\bg0.s|\\bg1.m|\\bg2.s|dna.replication|chromosome.segrega|mitosis|proliferat", ignore_case = TRUE), apoptosis = regex("apoptos|programmed.cell.death|caspase.cascade|intrinsic.apoptotic|extrinsic.apoptotic|\\bcaspase\\b|\\bbcl2\\b|\\bbax\\b|\\bbak\\b|\\bp53\\b|\\bdeath.receptor|\\bdeath.domain|\\bdeath.induced|\\bcell.death|\\bautophag|\\bautolysis", ignore_case = TRUE), differentiation = regex("neuron.*differentiat|neural.*differentiat|neurogenesis|neuron.*development|CNS.*development|CENTRAL.*NERVOUS.*SYSTEM.*differentia|axon.*develop|synaps.*project", ignore_case = TRUE), senescence = regex("senescence|cdkn2a|cdkn1a|sasp|\\bp21\\b|\\btelomere|\\btelomeric|\\bDNA.damage|\\bge

nomic.instability|\\bchromosomal.instability", ignore_case = TRUE)). GSEA was performed as described above using clusterProfiler (v4.10.0). For network visualization, pairwise Jaccard similarities were calculated between pathway core enrichment gene sets. Pathway pairs with Jaccard similarity ≥ 0.1 were connected by edges and visualized in Cytoscape (v3.9). For the neuronal differentiation theme, expression of upregulated core enrichment genes was visualized by a heatmap. Gene expression values (Transcripts Per Million, TPM) were log2-transformed, normalized by z-score across samples and displayed with a color scale from -2 to +2.

CUT&RUN procedure

Approximately 5×10^5 live cells were mixed with 5×10^4 Drosophila S2 cells per reaction. CUT&RUN was performed with CUTANA (EpiCypher) following manufacturer's protocols. In brief, we first isolated nuclei by incubating cells on ice for 5 min in Buffer A with protease inhibitor and 0.1% Triton X-100. After centrifugation at 1750 × g for 2 min at 4°C, nuclei were resuspended in Wash buffer. Bio-Mag®Plus Concanavalin-A (Con A) coated beads (BP531, Bangs Laboratories) activated in Binding buffer were then added to the nuclei and rotated for 10 min at room temperature. 1 µg primary antibody with 0.25 µg Spike-in antibody (61686, Active Motif) diluted in Antibody buffer was added to the bead-nuclei mixture and incubated for 2 h at room temperature. Beads were washed twice with Digitonin buffer and incubated with pAG-MNase for 10 min at room temperature. Beads were then washed twice with Digitonin buffer, incubated with 2 mM $CaCl_2$ for 2 h at 4°C, and quenched by adding Stop buffer. DNA was released from the beads by incubating them for 10 min at 37°C and purified by CUTANA DNA purification kit (SKU:14-0050, EpiCypher). Libraries were constructed using xGen ssDNA & Low-Input DNA Prep by following the manufacturer's instructions (10009817, Integrated DNA Technologies). Purified libraries were evaluated with TapeStation (Agilent) using the High Sensitivity D1000 reagents (5067-5585, Agilent) and sequenced on NovaSeq6000 sequencer to generate 50 bp paired-end reads. Antibodies used for CUT&RUN: Control IgG, H3K27me3, H3K4me3, H3K4me1, H3K36me2, H2AK119ub, BCOR, EZH2, HA-Tag and KDM2B (Supplementary Table 14). Buffers for CUT&RUN: Buffer A: 10 mM HEPES (pH 7.9), 10 mM KCl, 1.5 mM $MgCl_2$, 0.34 M sucrose, 10% glycerol; CUT&RUN Wash buffer: 20 mM HEPES (pH 7.5), 150 mM NaCl, 0.5 mM spermidine, protease inhibitor cocktail (11873580001, Sigma-Aldrich); CUT&RUN Binding buffer: 20 mM HEPES-KOH (pH 7.9), 10 mM KCl, 1 mM $CaCl_2$, 1 mM $MnCl_2$; CUT&RUN Digitonin buffer: CUT&RUN Wash buffer with 0.01% digitonin; CUT&RUN Antibody buffer: CUT&RUN Digitonin buffer with 2 mM EDTA; CUT&RUN 2 × Stop buffer: 340 mM NaCl, 20 mM EDTA, 4 mM EGTA; CUT&RUN Stop buffer: Into 1 mL of 2 × Stop buffer stock, add 5 µL of 10 mg/mL RNase A and 3.3 µL of 15 mg/mL GlycoBlue™ Coprecipitant (AM9516, ThermoFisher).

CUT&RUN data processing and visualization

Paired-end reads of 50 bp were mapped to human genome hg38 (Gencode GRCh38.p12) by BWA (v0.7.12-r1039, default parameters, RRID:SCR_010910)[18] after trimming for adapters by Trim_galore (v0.4.4, RRID:SCR_011847). Duplicated reads were marked with biobambam2 (v2.0.87, RRID:SCR_003308) and only non-duplicated reads were kept by samtools (v1.2, parameter "-q 1 -F 1804", RRID:SCR_002105)[19]. All samples confirmed to have > 5 million (M) fragments (range 7-23M, median 15M) as recommended[20].

For PDX CUT&RUN data, raw data were first processed using bbmap (v38.86, RRID:SCR_016965, parameters: "bbsplit.sh build=8 ref=Gencode/r31/bwa-index/0.7.17-r1188/GRCh38.primary_assembly.genome.fa,Gencode/M22/bwa-index/0.7.17-r1188/GRCm38.primary_assembly.genome.fa ambiguous=best ambiguous2=toss") to remove mouse reads before trimming.

Bigwig files were generated using the center 80 bp of fragments smaller than 2000bp and normalized to 10M fragments. Peaks were called by MACS2 (v2.1.1.20160309, parameter "-f BEDPE --keep-dup all", RRID:SCR_013291) using stringent cutoff (FDR corrected $P < 0.05$) and lower cutoff (FDR corrected $P < 0.5$). Reproducible peaks for each group were retained if called by stringent cutoff in ≥1 replicate and also called by lower cutoff in the other replicates with bedtools (v2.24.0, RRID:SCR_006646)[2]. Reproducible peaks from all groups were merged and fragments from each sample were counted at the merged peaks by bedtools. Fragments Per Kilobase of transcript per Million mapped reads (FPKM) were estimated and the top 3000 variable regions (pandas v1.1.5, std function, RRID:SCR_018214) were used for principal component analysis (sklearn v0.24.1, RRID:SCR_019053). Deeptools (v3.5.0, RRID:SCR_016366) were used to generate heatmap[21]. ggpubr (v0.4.0, RRID:SCR_021139) was used to make violin plots based on bigwig average signal extracted (bigWigAverageOverBed from UCSC tools https://github.com/ENCODE-DCC/kentUtils) from gene promoters (TSS ± 5kb). Two-sided t-test was used for comparisons.

Co-immunoprecipitation

Cells were incubated in nuclear extraction buffer (10 mM HEPES (pH 7.9), 10 mM KCl, 1.5 mM $MgCl_2$, 0.34 M sucrose, 10% glycerol, 0.1% Triton x-100, 1 × proteinase inhibitor (Roche)) for 5 min on ice. After centrifugation at 1750 g for 2 min at 4°C, nuclei pellet was incubated in lysis buffer (20 mM HEPES (pH 7.9), 400 mM KCl, 0.4 mM EDTA, 20% glycerol, 0.1% Triton x-100, 1 x protease inhibitor (Roche)) for 30 min at 4°C with rotation to obtain the nuclear fraction. After centrifugation at maximum speed for 10 min, the supernatant was mixed with an equal amount of water plus protease inhibitor. A small aliquot of the nuclear extract was saved as input. The remaining nuclear extract was precleared with 30 µL protein A and

protein G Dynabeads (ThermoFisher Scientific) and then incubated with antibodies overnight at 4°C with rotation. The next day, 30 µL protein A and protein G Dynabeads were added to the nuclear extract. After incubation for 2-4 hr at 4°C with rotation, beads were washed with PBST for three times then boiled at 70°C for 10 min with 2 x sample buffer.

Protein extraction for immunoblotting

Cell pellets were resuspended using RIPA buffer supplemented with 10 mM $MgCl_2$, 1 × Halt™ Protease and Phosphatase Inhibitor Cocktail (Thermo Fisher) and 30 U/mL DNase (Sigma), incubated on ice for 30 minutes, and sonicated for five seconds. After measuring protein concentration using the Pierce BCA kit (Thermo Fisher), samples were prepared with NuPAGE™ LDS Sample Buffer (Thermo Fisher) and heated at 70°C for 10 minutes.

Immunoblotting

Total protein extracts or immunoprecipitated proteins were separated by NuPAGE 4-12% Bis-Tris plus gels (Invitrogen) and transferred onto a PVDF membrane (Thermo Fisher). Membranes were blocked with 5% non-fat dry milk (Cell Signaling) or Odyssey PBS blocking buffer (Licor) at room temperature for 1 h and then incubated with primary antibodies (diluted in 1% non-fat dry milk or Odyssey PBS blocking buffer) overnight at 4°C. The next day, membranes were washed three times in PBST and incubated with secondary antibodies at room temperature for 1 h. After three washes with PBST, the blots were incubated with Pierce™ ECL Plus Western Blotting Substrate and exposed to X-Ray film or were imaged and quantified using Odyssey CLx and ImageStudio v5.2 (Licor).

Primary antibodies used were HA-Tag, GAPDH, beta-Actin, KDM2B, EZH2, JARID2, SUZ12, BCOR, RYBP, PCGF1, H3K4me1, H3K4me2, H3K4me3, H3K27me3, H3K36me2, and Histone H3. Secondary antibodies used were IRDye® 800CW Donkey anti-Rabbit IgG Secondary Antibody, Anti-rabbit IgG, HRP-linked Antibody and Anti-mouse IgG, HRP-linked Antibody (Supplementary Table 14).

Histopathology and immunohistochemistry (IHC) characterization of flank tumors

Freshly isolated flank tumors were fixed in 10% neutral-buffered formalin for 24–72 h at room temperature. Tissues were then washed in PBS, dehydrated, and embedded in paraffin. Paraffin-embedded tissue sections (4 µm thick) were collected, deparaffinized, rehydrated, subjected to antigen retrieval, and stained with hematoxylin and eosin (H&E) or with antibodies against HA-Tag, Ki-67, and cleaved caspase-3 (CC3). IHC staining was performed using automated Ventana BenchMark IHC stainers (Roche Diagnostics) with Discovery ChromoMap DAB detection kits (Roche Diagnostics) at the Comparative Pathology Core Facility at St. Jude Children's Research Hospital (SJCRH).

Stained slides were scanned using a Zeiss AxioScan Z1 slide scanner (Carl Zeiss Microscopy), and images were acquired using a 20×/0.8 NA objective. Quantification of HA, Ki-67, and CC3 positive tumor areas was performed for control or dTAG treated tumors (n = 5/group) in a blinded manner by imaging scientists in the Cell and Tissue Imaging Center (CTIC) at St. Jude Children's Research Hospital. Brightfield whole-slide images of DAB-stained sections were analyzed using QuPath software (v0.6.0)[22]. All sections stained with the same antibody were analyzed using identical settings. Stain vectors were automatically defined using the "Estimate Stain Vectors" function, after which total tissue area and DAB-positive areas were annotated using the "Create Thresholder" tool. Threshold settings were selected based on visual inspection. The ratio of DAB-positive area to total tissue area was calculated using Microsoft Excel, yielding the fraction of tissue positive for DAB staining. *P* was calculated by two-tailed t-test.

Histone mass spectrometry

About 20 mg of purified acid-extracted histones were derivatized using propionic anhydride followed by digestion with 1 mg trypsin for bottom-up mass spectrometry[23]. After desalting, the peptides were separated using Thermo Scientific Acclaim PepMap 100 C18 HPLC Column (250 mm length, 0.075 mm I.D., Reversed Phase, 3 mm particle size) fitted on a Vanquish™ Neo UHPLC System (Thermo Scientific). The HPLC gradient used was: 2% to 35% solvent B (A = 0.1% formic acid; B = 95% MeCN, 0.1% formic acid) over 50 minutes to 99% solvent B in 10 minutes, all at a flow-rate of 300 nL/min. Using a QExactive-Orbitrap mass spectrometer (Thermo Scientific) and a data-independent acquisition, 5 µL of 1 mg/mL sample was analyzed[23]. In brief, full scan MS (m/z 295−1100) in the Orbitrap with a resolution of 70,000 and an AGC target of 1×10^6 was acquired. In the ion trap, tandem MS was acquired in centroid mode using sequential isolation windows of 24 m/z, AGC target of 2×10^5, CID collision energy of 30 and a maximum injection time of 50 msec. The raw data was deconvoluted and analyzed using an in-house software, EpiProfile v2.0[24]. The data was expressed as peptide relative ratios (%) of total area under the extracted ion chromatogram of particular peptide form to the sum of unmodified and modified forms belonging to the same peptide with the same amino acid sequence.

References

1. Hovestadt, V. *et al.* Decoding the regulatory landscape of medulloblastoma using DNA methylation sequencing. *Nature* **510**, 537–41 (2014).
2. Quinlan, A.R. & Hall, I.M. BEDTools: a flexible suite of utilities for comparing genomic features. *Bioinformatics* **26**, 841–2 (2010).

3. Northcott, P.A. *et al.* The whole-genome landscape of medulloblastoma subtypes. *Nature* **547**, 311–317 (2017).
4. Miller, J.A. *et al.* Transcriptional landscape of the prenatal human brain. *Nature* **508**, 199–206 (2014).
5. Sepp, M. *et al.* Cellular development and evolution of the mammalian cerebellum. *Nature* **625**, 788–796 (2024).
6. Wolf, F.A., Angerer, P. & Theis, F.J. SCANPY: large-scale single-cell gene expression data analysis. *Genome Biol* **19**, 15 (2018).
7. Dharia, N.V. *et al.* A first-generation pediatric cancer dependency map. *Nat Genet* **53**, 529–538 (2021).
8. DepMap, Broad (2025). DepMap Public 26Q3. Dataset. depmap.org.
9. Arafeh, R., Shibue, T., Dempster, J.M., Hahn, W.C. & Vazquez, F. The present and future of the Cancer Dependency Map. *Nat Rev Cancer* **25**, 59–73 (2025).
10. Giurgiu, M. *et al.* CORUM: the comprehensive resource of mammalian protein complexes-2019. *Nucleic Acids Res* **47**, D559–D563 (2019).
11. Williams, J.S. *et al.* Patient-derived pediatric brain tumor orthotopic xenografts and tumor organoids faithfully recapitulate primary tumors. *Sci Adv* (2026).
12. Narina, S., Connelly, J.P. & Pruett-Miller, S.M. High-Throughput Analysis of CRISPR-Cas9 Editing Outcomes in Cell and Animal Models Using CRIS.py. *Methods Mol Biol* **2631**, 155–182 (2023).
13. Connelly, J.P. & Pruett-Miller, S.M. CRIS.py: A Versatile and High-throughput Analysis Program for CRISPR-based Genome Editing. *Sci Rep* **9**, 4194 (2019).
14. Dobin, A. *et al.* STAR: ultrafast universal RNA-seq aligner. *Bioinformatics* **29**, 15–21 (2013).
15. Li, B. & Dewey, C.N. RSEM: accurate transcript quantification from RNA-Seq data with or without a reference genome. *BMC Bioinformatics* **12**, 323 (2011).
16. Law, C.W., Chen, Y., Shi, W. & Smyth, G.K. voom: Precision weights unlock linear model analysis tools for RNA-seq read counts. *Genome Biol* **15**, R29 (2014).
17. Yu, G., Wang, L.G., Han, Y. & He, Q.Y. clusterProfiler: an R package for comparing biological themes among gene clusters. *OMICS* **16**, 284–7 (2012).
18. Li, H. & Durbin, R. Fast and accurate short read alignment with Burrows-Wheeler transform. *Bioinformatics* **25**, 1754–60 (2009).
19. Li, H. *et al.* The Sequence Alignment/Map format and SAMtools. *Bioinformatics* **25**, 2078–9 (2009).
20. Skene, P.J., Henikoff, J.G. & Henikoff, S. Targeted in situ genome-wide profiling with high efficiency for low cell numbers. *Nat Protoc* **13**, 1006–1019 (2018).
21. Ramirez, F. *et al.* deepTools2: a next generation web server for deep-sequencing data analysis. *Nucleic Acids Res* **44**, W160–5 (2016).
22. Bankhead, P. *et al.* QuPath: Open source software for digital pathology image analysis. *Sci Rep* **7**, 16878 (2017).
23. Sidoli, S., Bhanu, N.V., Karch, K.R., Wang, X. & Garcia, B.A. Complete Workflow for Analysis of Histone Post-translational Modifications Using Bottom-up Mass Spectrometry: From Histone Extraction to Data Analysis. *J Vis Exp* (2016).
24. Yuan, Z.F. *et al.* EpiProfile 2.0: A Computational Platform for Processing Epi-Proteomics Mass Spectrometry Data. *J Proteome Res* **17**, 2533–2541 (2018).
