# Supplementary material for: Divergent medulloblastoma chromatin states disclose KDM2B as a selective dependency
Source: Nat Genet. 2026 Sep 24;58(10):2590–604. doi: 10.1038/s41588-026-02745-1 (PMC13649585; doi:10.1038/s41588-026-02745-1)
Supplement: Supplementary file 3 [file 41588_2026_2745_MOESM2_ESM.pdf]

nature portfolio

Corresponding author(s): Paul A. Northcott

Last updated by author(s): Jul 20, 2026

Reporting Summary

Nature Portfolio wishes to improve the reproducibility of the work that we publish. This form provides structure for consistency and transparency in reporting. For further information on Nature Portfolio policies, see our Editorial Policies and the Editorial Policy Checklist.

Statistics

For all statistical analyses, confirm that the following items are present in the figure legend, table legend, main text, or Methods section.

n/a	Confirmed	
☐	☒	The exact sample size (n) for each experimental group/condition, given as a discrete number and unit of measurement
☐	☒	A statement on whether measurements were taken from distinct samples or whether the same sample was measured repeatedly
☐	☒	The statistical test(s) used AND whether they are one- or two-sided<br>*Only common tests should be described solely by name; describe more complex techniques in the Methods section.*
☒	☐	A description of all covariates tested
☐	☒	A description of any assumptions or corrections, such as tests of normality and adjustment for multiple comparisons
☐	☒	A full description of the statistical parameters including central tendency (e.g. means) or other basic estimates (e.g. regression coefficient) AND variation (e.g. standard deviation) or associated estimates of uncertainty (e.g. confidence intervals)
☐	☒	For null hypothesis testing, the test statistic (e.g. F, t, r) with confidence intervals, effect sizes, degrees of freedom and P value noted<br>*Give P values as exact values whenever suitable.*
☒	☐	For Bayesian analysis, information on the choice of priors and Markov chain Monte Carlo settings
☒	☐	For hierarchical and complex designs, identification of the appropriate level for tests and full reporting of outcomes
☒	☐	Estimates of effect sizes (e.g. Cohen's d, Pearson's r), indicating how they were calculated

Our web collection on statistics for biologists contains articles on many of the points above.

Software and code

Policy information about availability of computer code

Data collection	ChIP-seq libraries were sequenced on the Illumina HiSeq 2000 platform according to the manufacturer's instructions.<br>Sequencing of RNA-seq libraries for MB cell lines or PDXs was performed on Illumina NovaSeq sequencers, per the manufacturer's directions.<br>CUT&RUN libraries were sequenced on NovaSeq6000 sequencer.
Data analysis	Individual software components are as follows: deepTools v3.5.0; bedtools v2.24.0 or v2.25.0; UCSC genome browser; chromHMM v.1.24; Python (genome_info v0.1.0 or v1.0.8, scipy v1.9.3 or v1.13.0, matplotlib v3.9.2, GSEApy v1.1.3, scanpy v1.9.3, gseapy v1.1.0, pandas v1.1.5, sklearn v0.24.1); R (ggtern v3.4.2, DESeq2 v1.40.2, rstatix v 0.7.2, ggplot v3.4.0, ggpubr v0.4.0); DAVID v6.8; Cytoscape v3.9; Enrichr (https://maayanlab.cloud/Enrichr/); clusterProfiler v4.10.0; GraphPad Prism v10; Microsoft Excel; STAR v2.7.1a; RSEM v1.3.2; GENCODE v31; Voom; BWA v0.7.12-r1039; Trim_galore v0.4.4; samtools v1.2; biobambam2 v2.0.87; bbmap v38.86; MACS2 v2.1.1.20160309; QuPath v0.6.0; ImageStudio v5.2 (Licor); EpiProfile v2.0

For manuscripts utilizing custom algorithms or software that are central to the research but not yet described in published literature, software must be made available to editors and reviewers. We strongly encourage code deposition in a community repository (e.g. GitHub). See the Nature Portfolio guidelines for submitting code & software for further information.

Data

Policy information about availability of data

All manuscripts must include a data availability statement. This statement should provide the following information, where applicable:

- Accession codes, unique identifiers, or web links for publicly available datasets
- A description of any restrictions on data availability
- For clinical datasets or third party data, please ensure that the statement adheres to our policy

Patient ChIP-seq and RNA-seq data have been deposited at the European Genome-Phenome Archive (EGA, http://www.ebi.ac.uk/ega/) hosted by the EBI, under accession number EGAS00001000215; whole genome sequencing, expression microarray and DNA methylation array data under accession number EGAS00001001953; and whole genome bisulfite sequencing data under accession number EGAS00001000561. RNA-seq and CUT&RUN data for MB cell lines or PDXs was deposited in GEO under accession code GSE269909 (RNA-seq) and GSE270125 (CUT&RUN). Bulk KDM2B expression values normalized as Transcripts Per Million across developmental stages were downloaded from: https://www.brainspan.org/static/download.html. The mass spectrometry proteomics data has been deposited to the ProteomeXchange Consortium via the MassIVE partner repository with the dataset identifier PXD079139. The single cell cerebellum data can be downloaded from: https://apps.kaessmannlab.org/sc-cerebellum-transcriptome/. DepMap CRISPR dependency data (v26Q3) can be downloaded from DepMap portal (https://depmap.org/portal/achilles/). Plasmids and cell lines are available upon request.

Research involving human participants, their data, or biological material

Policy information about studies with human participants or human data. See also policy information about sex, gender (identity/presentation), and sexual orientation and race, ethnicity and racism.

Reporting on sex and gender	Sex was not considered in the study design and no sex-based analysis was performed. Tumor samples from 34 males, 17 females and 1 individual with Klinefelter syndrome were included in the study. Cell lines derived from both male and female patients were used in the study. Male: D283, D425, CHLA-01-MED, Daoy, RCMB28, SJNBT3, SJ-HGGX99c, SJ-HGGX77c, SJ-HGGX90c. Female: Med113FH, Med1712FH, Med2312FH, Med1512FH, RCMB37, SJ-DIPGX7. Sex not disclosed: RCMB20, DMB006, SJNBT12. Due to availability, a limited number of tumor samples and cell models were included in the study, which prevents robust sex-based analysis.
Reporting on race, ethnicity, or other socially relevant groupings	Race, ethnicity, or other socially-relevant groupings were not considered when designing this study.
Population characteristics	Information about the data portal can be found in Zhang, J., Bajari, R., Andric, D. et al. The International Cancer Genome Consortium Data Portal. Nat Biotechnol 37, 367–369 (2019). https://doi.org/10.1038/s41587-019-0055-9. Population characteristics were not considered for cohort inclusion or exclusion. No population characteristics outside of sex are reported.
Recruitment	Recruitment was performed outside the scope of the current study. Patients provided informed consent and were recruited at contributing ICGC centers. The cohort was chosen from available samples based on a medulloblastoma molecular diagnosis.
Ethics oversight	This study involves no new patient sample collection and informed and written consent exists for all patients contributing to the study.

Note that full information on the approval of the study protocol must also be provided in the manuscript.

Field-specific reporting

Please select the one below that is the best fit for your research. If you are not sure, read the appropriate sections before making your selection.

☒ Life sciences ☐ Behavioural & social sciences ☐ Ecological, evolutionary & environmental sciences

For a reference copy of the document with all sections, see nature.com/documents/nr-reporting-summary-flat.pdf

Life sciences study design

All studies must disclose on these points even when the disclosure is negative.

Sample size	Sample size was determined by sample availability. No sample-size calculation was performed.
Data exclusions	No data was excluded from the analysis.
Replication	At least three replicates were performed for the RNA-seq analysis following KDM2B perturbation in MB cells and results from independent replicates were combined in the downstream summarization. Two to three replicates were performed for the CUT&RUN analysis following KDM2B perturbation in D283 cells. At least two biological replicates were performed for functional assays using multiple cell lines and models when available. Results are consistent among replicates.

Randomization	The study design did not warrant randomization.
Blinding	For in vivo dTAG treatment using the flank tumor model, the investigators were blinded to group identity during tumor size measurements and outcome assessments. Immunochemistry image quantification of tumor samples was performed in a blinded manner by imaging scientists using QuPath.

Reporting for specific materials, systems and methods

We require information from authors about some types of materials, experimental systems and methods used in many studies. Here, indicate whether each material, system or method listed is relevant to your study. If you are not sure if a list item applies to your research, read the appropriate section before selecting a response.

Materials & experimental systems

n/a	Involved in the study	
☐	☒	Antibodies
☐	☒	Eukaryotic cell lines
☒	☐	Palaeontology and archaeology
☐	☒	Animals and other organisms
☒	☐	Clinical data
☒	☐	Dual use research of concern
☒	☐	Plants

Methods

n/a	Involved in the study	
☐	☒	ChIP-seq
☐	☒	Flow cytometry
☒	☐	MRI-based neuroimaging

Antibodies

Antibodies used	For ChIP-seq: H3K27me3 (07-449, MilliporeSigma,lot#JBC1854858, 4 µg), H3K4me1 (39297, ActiveMotif, lot#1, 5 µl), H3K4me3 (39159, ActiveMotif, lot#4, 3 µl), H3K9me3 (39161, ActiveMotif, , lot#2, 5 µl), H3K27ac (39133, ActiveMotif,4 µg) and H3K36me3 (61101, ActiveMotif, ,lot#2, 4µg).<br>For CUT&RUN (1 µg): Control IgG (AB-105-C, R&D systems), H3K27me3 (07-449, MilliporeSigma), H3K4me3 (C15410003, Diagenode), H3K4me1 (39298, Active Motif), H3K36me2 (MA5-14867, Thermo Fisher, Clone T.571.7), H2AK119ub (8240S, Cell Signaling, clone D27C4), BCOR (12107-1-AP, Proteintech), EZH2 (39076, Active Motif, Clone AC22), HA-Tag (3724S, Cell Signaling, Clone C29F4) and KDM2B (09-864, MilliporeSigma). (0.25 µg):Spike-in antibody (61686, ActiveMotif).<br>For immunoprecipitation: Control IgG (AB-105-C, R&D system or NI01, MilliporeSigma, 5µg), KDM2B (09-864, MilliporeSigma, lot#3827475, 5µg), and HA-Tag (3724S, Cell Signaling, Clone C29F4, lot#13, 1:50).<br>For immunoblotting: HA-Tag (3724S, Cell Signaling, Clone C29F4, lot#13, 1:1000), GAPDH (2118L, Cell Signaling, Clone 14C10, 1:1000), beta-Actin (8457S, Cell Signaling, Clone D6A8, lot#8, 1:1000), KDM2B (09-864, MilliporeSigma, lot#3827475, 1:1000), EZH2 (3147S, Cell Signaling, Clone AC22, lot#4, 1:1000), JARID2 (1NB100-2214, Novus Biologicals, lot#G-10, 1:1000), SUZ12 (sc-271325, Santa Cruz, Clone D-10, lot#F0922, 1:200), BCOR (sc-514576, Santa Cruz, Clone C-10, lot#D3025, 1:200), RYBP (sc-374235, Santa Cruz, Clone A-1, lot#A2622, 1:500), PCGF1 (sc-515371, Santa Cruz, Clone E-8, lot#B0325, 1:100), H3K4me1 (ab8895, Abcam, 1:500), H3K4me2 (ab7766, Abcam, 1:800), H3K4me3 (ab8580, Abcam, 1:1000), H3K27me3 (9733S, Cell Signaling, Clone C36B11, 1:1000), H3K36me2 (ab9049, Abcam, 1 µg /mL), and Histone H3 (ab1791, Abcam, 1:1000). Secondary antibodies used were IRDye® 800CW Donkey anti-Rabbit IgG (926-32213, Licor, 1:16000), Anti-rabbit IgG, HRP-linked Antibody (7074S, Cell Signaling, lot#33, 1:5000) and Anti-mouse IgG, HRP-linked Antibody (7076S, Cell Signaling, lot#38, 1:5000).<br>For immunohistochemistry staining: HA-Tag (3724s, Cell Signaling, Clone C29F4), Ki-67 (RM-9106, Thermo Fisher, Clone SP6), and cleaved caspase-3 (9664, Cell Signaling, Clone 5A1E).
Validation	All antibodies used are commercially available and validated by the vendor; validation information is posted on the product pages.

Eukaryotic cell lines

Policy information about cell lines and Sex and Gender in Research

Cell line source(s)	D283 (male, HTB-185, ATCC), D425 (male, SCC290, MilliporeSigma), CHLA-01-MED (male, CRL-3021, ATCC), and Daoy (male, HTB-186, ATCC) are commercially available. Med113FH (female), Med1712FH (female), Med2312FH (female), and Med1512FH (female) were acquired from the Brain Tumor Resource Laboratory of Seattle Children's Research Institute. RCMB20 (sex not disclosed), RCMB28 (male), RCMB37 (female), and DMB006 (sex not disclosed) were acquired from Robert Wechsler-Reya. SJNBT12 (sex not disclosed), SJNBT3 (male), SJ-HGGX99c (male), SJ-HGGX77c (male), SJ-HGGX90c (male), or SJ-DIPGX7 (female) were derived from St Jude PDX core.
Authentication	Cell line authentication was performed by short tandem repeat (STR) profiling or DNA methylation array.
Mycoplasma contamination	Cell lines tested negative for mycoplasma contamination using the Universal Mycoplasma Detection Kit from ATCC (30-1012K).
Commonly misidentified lines (See ICLAC register)	No commonly misidentified cell lines were used in the study.

Animals and other research organisms

Policy information about studies involving animals; ARRIVE guidelines recommended for reporting animal research, and Sex and Gender in Research

Laboratory animals	8-12 weeks old female NSG mice (The Jackson Laboratory - 005557) were used for in vivo experiments.
Wild animals	No wild animals were used in the study.
Reporting on sex	Only female NSG mice were used in the study for ease of handling for the experimental procedures. No studies have shown any difference in MB PDX growth between male and female NSG mice.
Field-collected samples	No field collected samples were used in the study.
Ethics oversight	All experiments were conducted in accordance with the guidelines established by the National Institute of Health's Guide for the Care and Use of Laboratory Animals and approved by the St. Jude Children's Research Hospital (SJCRH) Institutional Animal Care and Use Committee (IACUC protocol #589–100536). SJCRH is registered as a research facility with the USDA and maintains an assurance statement with the Office of Laboratory Animal Welfare of the NIH (Animal Assurance Number A3077-01).

Note that full information on the approval of the study protocol must also be provided in the manuscript.

Plants

Seed stocks	n/a
Novel plant genotypes	n/a
Authentication	n/a

ChIP-seq

Data deposition

☒ Confirm that both raw and final processed data have been deposited in a public database such as GEO.

☒ Confirm that you have deposited or provided access to graph files (e.g. BED files) for the called peaks.

Data access links<br>*May remain private before publication.*	Part of the H3K27ac ChIP-seq dataset included in this study has been previously published by our group: Lin et al., Nature 2016. All raw ChIP-seq data can be accessed via EGA (http://www.ebi.ac.uk/ega/) hosted by the EBI, under accession number EGAS00001000215. The ChIP-seq tracks are also available at St Jude Data Portal: https://proteinpaint.stjude.org/MBEpi/
Files in database submission	ChIP-seq files included in the study are accessible in the database listed above.
Genome browser session<br>(e.g. UCSC)	https://proteinpaint.stjude.org/MBEpi/

Methodology

Replicates	Number of biological replicate depend on sample availability. WNT, n = 2; SHH, n = 5; Group 3, n = 19; Group 4, n = 26 samples.
Sequencing depth	Samples were sequenced using 100bp paried-end reads sequenced at >1 million reads
Antibodies	H3K27ac (#39133, AciveMotif), H3K27me3 (#07-449, Millipore), H3K4me1 (AM#39297, ActiveMotif), H3K4me3 (AM#39159, ActiveMotif), H3K9me3 (AM#39161, ActiveMotif) and H3K36me3 (AM#61101, ActiveMotif).
Peak calling parameters	MACS2 was used using default parameters
Data quality	The number of reads in peaks was determined as a general quality control metric for samples generated by ActiveMotif.
Software	deepTools v3.5.0, bedtools v2.24.0 and v2.25.0, MACS2 v2.1.1.20160309, ChromHMM v1.24, R, and genome_info (v.0.1.0) in Python were used.

Flow Cytometry

Plots

Confirm that:

- [ ] The axis labels state the marker and fluorochrome used (e.g. CD4-FITC).
- [ ] The axis scales are clearly visible. Include numbers along axes only for bottom left plot of group (a 'group' is an analysis of identical markers).
- [ ] All plots are contour plots with outliers or pseudocolor plots.
- [ ] A numerical value for number of cells or percentage (with statistics) is provided.

Methodology

Sample preparation	D283 and D425 cells were cultured in suspension and dissociated by pipetting up and down before collecting for flow cytometry. CHLA-01-MED cells were cultured in suspension and dissociated by Accutase before collecting for flow cytometry. SJNBT3, SJNBT12, Daoy, SJ-HGGX77c, SJ-HGGX99c, SJ-HGGX90c, and SJ-DIPGX7 were cultured in attached condition and were dissociated using Accutase before collecting for flow cytometry. PDX cells collected from mice cerebellum were dissociated into single cell using papain and then analyzed by flow cytometry.
Instrument	FACSAria Fusion and FACSymphony A1 (Becton Dickinson)
Software	BD FACSDiva 8.01 and FlowJo
Cell population abundance	For each sample, 20,000 live single cells were analyzed to determine the percentage of GFP or mcherry+ cells.
Gating strategy	The cells were gated based on FSC/SSC and DAPI/FSC to select live cells. After doublets were removed, GFP+ or mcherry+ cells were gated by using negative control cells.

- [ ] Tick this box to confirm that a figure exemplifying the gating strategy is provided in the Supplementary Information.
