# Supplementary material for: Divergent medulloblastoma chromatin states disclose KDM2B as a selective dependency
Source: Nat Genet. 2026 Sep 24;58(10):2590–604. doi: 10.1038/s41588-026-02745-1 (PMC13649585; doi:10.1038/s41588-026-02745-1)
Supplement: Supplementary file 4 [file 41588_2026_2745_MOESM3_ESM.pdf]

nature portfolio

Peer Review File

Divergent medulloblastoma chromatin states disclose KDM2B as a selective dependency

Corresponding Author: Dr Paul Northcott

Version 0:

Decision Letter:

24th Jul 2024

Dear Dr Northcott,

I hope you're well.

Your Article, "Divergent medulloblastoma chromatin states disclose KDM2B as a selective dependency" has now been seen by 3 referees. You will see from their comments copied below that while they find your work of considerable potential interest, they have raised quite substantial concerns that must be addressed. In light of these comments, we cannot accept the manuscript for publication, but would be very interested in considering a revised version that addresses these serious concerns.

We hope you will find the referees' comments useful as you decide how to proceed. If you wish to submit a substantially revised manuscript, please bear in mind that we will be reluctant to approach the referees again in the absence of major revisions.

If you choose to revise your manuscript taking into account all reviewer and editor comments, please highlight all changes in the manuscript text file. At this stage we will need you to upload a copy of the manuscript in MS Word .docx or similar editable format.

We are committed to providing a fair and constructive peer-review process. Do not hesitate to contact us if there are specific requests from the reviewers that you believe are technically impossible or unlikely to yield a meaningful outcome.

If revising your manuscript:

*1) Include a “Response to referees” document detailing, point-by-point, how you addressed each referee comment. If no action was taken to address a point, you must provide a compelling argument. This response will be sent back to the referees along with the revised manuscript.

*2) If you have not done so already please begin to revise your manuscript so that it conforms to our Article format instructions, available here.
Refer also to any guidelines provided in this letter.

*3) Include a revised version of any required Reporting Summary: https://www.nature.com/documents/nr-reporting-summary.pdf
It will be available to referees (and, potentially, statisticians) to aid in their evaluation if the manuscript goes back for peer review.
A revised checklist is essential for re-review of the paper.

Please be aware of our guidelines on digital image standards.

You may use the link below to submit your revised manuscript and related files:

Link Redacted

Note: This URL links to your confidential home page and associated information about manuscripts you may have submitted, or that you are reviewing for us. If you wish to forward this email to co-authors, please delete the link to your homepage.

If you wish to submit a suitably revised manuscript we would hope to receive it within 6 months. If you cannot send it within this time, please let us know. We will be happy to consider your revision so long as nothing similar has been accepted for publication at Nature Genetics or published elsewhere. Should your manuscript be substantially delayed without notifying us in advance and your article is eventually published, the received date would be that of the revised, not the original, version.

Please do not hesitate to contact me if you have any questions or would like to discuss the required revisions further.

Nature Genetics is committed to improving transparency in authorship. As part of our efforts in this direction, we are now requesting that all authors identified as 'corresponding author' on published papers create and link their Open Researcher and Contributor Identifier (ORCID) with their account on the Manuscript Tracking System (MTS), prior to acceptance. ORCID helps the scientific community achieve unambiguous attribution of all scholarly contributions. You can create and link your ORCID from the home page of the MTS by clicking on 'Modify my Springer Nature account'. For more information please visit please visit www.springernature.com/orcid.

Thank you for the opportunity to review your work.

Sincerely,

Safia Danovi, PhD
Senior Editor, Nature Genetics
ORCiD: 0009-0007-7822-5479

Referee expertise:

Referee #1: MB epi/genomics

Referee #2: brain tumour epigenetics

Referee #3: MB targets

Reviewers' Comments:

Reviewer #1:
Remarks to the Author:
In the manuscript, Tao et al. identified a chromatin state of a bivalent/poised enhancer (EnhBiv) enriched at the promoters of neurodevelopmental genes in Group 4 medulloblastoma (MB). They further determined that KDM2B, a histone lysine demethylase, is highly expressed in Group 4 MB and can occupy the promoter regions of EnhBiv-regulated genes. CRISPR-mediated knockout and protein degradation of KDM2B inhibited MB cell growth both in vitro and in vivo. Further validations revealed that KDM2B controls the EnhBiv chromatin state in Group 4 MB by binding to EZH2. The manuscript provides new insights into the epigenetic mechanisms of subgroup determination in MB to some extent. The following comments may improve the quality of the manuscript:
1) It is confusing that a bivalent enhancer state, based on H3K4me1 and H3K27me3, was enriched in Group 4 MB but was marked at the gene promoter regions. What are the promoter-poised states across the four subgroups of MB based on H3K4me3 and H3K27me3?
2) The conclusion in Figure 2 suggests that the EnhBiv state in Group 4 represents a less differentiated state, keeping cells in a progenitor status. Group 3 is generally considered to stem from neural progenitor cells, and both Group 3 and Group 4 share a unified cell of origin but exist along a continuum of developmental steps. What chromatin state trajectory determines the subgroup tumorigenesis? For instance, what is the Group 3-specific chromatin state, and how is it related to the EnhBiv state in Group 4?
3) KDM2B has been reported to encode an H3K36me2-specific demethylase. What is the H3K36me2 signal across the subgroups of MB, particularly in Group 4?
4) Why is there no difference in the H3K27me3 mark among the three subgroups in Fig 3e?
5) Whether the significant enrichment of the EnhBiv state at KDM2B-bound promoters compared with KDM2B-deprived promoters occurred in Group 3 and SHH groups?
6) What is the cell fate after KDM2B deletion in the in vitro and in vivo competition assays shown in Figure 4? It would be helpful to examine cell differentiation, apoptosis/death, senescence, and so on.
7) What is the mouse survival difference between the controls and KDM2B KO groups? Additionally, what are the pathological changes observed in these tumors after KDM2B KO, as shown in Figures 4f and 4g?
8) In vivo competition assay should be used to test dTAG-mediated KDM2B protein degradation in two Group 4 models (DMB006 and Med2312FH).
9) How many genes overlap between KDM2B-regulated genes (Figure 5a) and Group 4 EnhBiv signature genes? Can altering KDM2B expression change the Group 4 EnhBiv signature genes and affect the tumor subgroup? For example, one might wonder if KDM2B overexpression in Group 3 and SHH cell lines could switch these cell lines to a Group-4-like

subgroup.
10) KDM2B deletion in Group 3 cell line D283 showed modest changes in histone marks and EnhBiv enrichment (Fig 5d-f). Global gene expression and chromatin landscape changes (cut&run) can be analyzed in Group 4 PDX tumors (DMB006 and Med2312FH-derived tumors following KDM2B deletion, which may better recapitulate the Group 4 context.
11) What are the H3K26me2 peaks in D283 cells following KDM2B deletion in CUT&RUN experiments?
12) It is unclear that subgroup specific KDM2B peaks were enriched for H3K4me1 (Fig 3e), but KDM2B KO significantly influenced H3K27me3 rather than H3K4me1 in Fig 5.
13) KDM2B has been extensively linked to the polycomb repressive complexes, including PRC1 and PRC2 in gene regulation. Why PRC2 rather than PRC1 was involved in the KDM2B-controlled chromatin state in Group 4?
14) Since KDM2B is a key epigenetic factor in determining MB subgroups, genetic KDM2B knockout and/or transgenic KDM2B mouse models would be useful to illustrate lineage differentiation and induced MB formation, thereby validating tumor type specificity.
15) Whether genetic deletion of EZH2 or EZH2 inhibitors can dismantle KDM2B-controlled EnhBiv state in Group 4 MB?

Reviewer #2:
Remarks to the Author:
In this study, the authors acquired 254 histone modification maps, including H3K4me1, H3K4me3, H3K27ac, H3K36me3, H3K27me3, H3K9me3 in 52 primary fresh-frozen human medulloblastoma tissue samples and stratified the genomes by chromatin state. They identified an enhancer bivalent state (occupied by H3K27me3 and H3K4me1) specifically enriched in Group 4 medulloblastomas and provided evidence that this state is promoted by KDM2B through its recruitment of PRC2 to chromatin to repress neurodevelopmental genes. The study further validated KDM2B, which could be a novel therapeutic target, as a selective dependency in Group 3/4 medulloblastomas.
This new tumor dataset is important, and the computational methods employed are effective in simplifying the comparison of chromatin states between tumors. The novel finding of a tumor dependency mediated by bivalent chromatin through KDM2B polycomb recruitment, along with the promising selectivity of this dependency, adds significant value to the field. However, the importance of KDM2B in MB and the mechanistic link between KDM2B, PRC2, and neurodevelopmental genes needs additional evidence.
Major Comments
1. Selectivity of KDM2B Dependency: The selectivity of KDM2B dependency in group 4 MB should be substantiated. Could the authors use DepMap or similar databases to compare KDM2B sensitivity between group 4 and other subtypes of medulloblastoma lines. Could the authors leverage additional cancer genomics datasets to correlate KDM2B expression with patient survival, tumor stage, and other clinical parameters. Alternatively, the authors can perform KDM2B knockdown in additional cell line to rescue or validate its importance in group 4 MB to confirm the selectivity of this dependency.
2. Enhancer Bivalent vs. Promoter Bivalent Distinction: The distinction between enhancer bivalent and promoter bivalent states is unclear. Figure 2C shows the enhancer bivalent state mostly enriched at promoters, and Figure 3F focuses on KDM2B-bound promoters, not enhancers. In Figures 3A/S3A, cluster C6 highlights the major difference as the presence of the enhancer bivalent state, while all four groups exhibit the promoter bivalent state. However, KDM2B is not enriched in Group 4 for this cluster; instead, KDM2B enrichment is observed in clusters where Group 4 also selectively has the promoter bivalent state. Please provide views of additional representative loci and promoter regions with ChIP-seq tracks for H3K4me1, H3K4me3, and H3K27me3 at enhancer bivalent loci. Also can the authors explain their particular preference for bivenh over bivprom based on some epigenomic or genomic (CG-density?) features?
3. Mechanistic Link Between KDM2B and PRC2: The mechanistic link between KDM2B and PRC2 is incomplete. KDM2B is known to recruit PRC1 instead of PRC2. Although the paper presents the co-localization of KDM2B and EZH2 in the genome, the physical interaction between KDM2B and EZH2 (Fig. 6i) and the reduction of EZH2 seems weak (Fig. 6f). Can authors quantify their co-IP results and compare to PRC1 component (Fig. 6f)? The proposed recruitment could be indirect, resulting from long-term KDM2B knockout rather than direct recruitment of PRC2 by KDM2B. To address this, the authors can use their dTag cell line to show through ChIP-qPCR or ChIP-seq that PRC2 components (EZH2 or EED) quickly disappear from EnhBiv chromatin within hours of KDM2B degradation. The authors should also plot EZH2 binding in KDM2B KO cells in Figure 6d, matching the same set of KDM2B binding sites, to show the degree of EZH2 binding loss. If there is no strong association between PRC2 recruitment and KDM2B, please address alternative explanations for these effects in the discussion section. As KDM2B is well-known to regulate K4me and K36me, is there a change of K36me over up-regulated genes upon KDM2B KO?
4. Neurodevelopmental Gene Impact: While the authors proposed that the repression of the neurodevelopmental genes is the major effect of KDM2B-mediated bivalent states, the direct impact of KDM2B on neurodevelopmental genes should be solidified. It is unclear how enriched neurodevelopment genes are in the current analysis in figure 2F. Authors should include a more standard GO analysis (e.g. DAVID, geneontology) with appropriate statistics to better support its enrichment of neurodevelopment genes and have the gene list provided. What percentage of enhBiv genes are neurodevelopment genes? In Figure 5, gene expression analyses should be subsetted to look specifically at neurodevelopmental enhancer bivalent signature genes instead of all enhancer bivalent signature genes. This would strengthen the link between KDM2B and repression of neurodevelopmental gene regulation.

Minor Comments

1. Figure 1A: The coloration for the methylation score is confusing as it is similar to other colors used.
2. Figure 2D: Clarify the proportion of all promoters with the enhancer bivalent state in each group. Are Group 4 promoters broadly enriched for the enhancer bivalent state?
3. Figure 2E: The changes are subtle, and the significance appears amplified by using each gene as a separate data point

instead of averaging the gene set and comparing each sample. Please note this or use a different representation.
4. Figure 2H: The representation is hard to interpret. Increase the alpha and/or move red dots to the front or use a different representation to show density.
5. Figure S4D: Shouldn't this be labeled KDM2B wild type, not dTAG?
6. Figure 4K: Should the x-axis be dTag treatment, not infection?

Reviewer #3:
Remarks to the Author:
The manuscript by Tao and colleagues utilizes the chromatin landscape definition to investigate and identify unique features and vulnerabilities in medulloblastoma (MB) subgroups. This builds on prior data and publications from their group and others. They find that the bivalent/poised enhancer (EnhBiv) chromatin signature was specifically enriched at Grp4-MB neurodevelopmental gene promoters. These EnhBiv spots are also enriched in KDM2B binding, which the authors find is highly expressed in Grp4-MB. Removal of KDM2B through different mechanisms inhibits in vitro and in vivo growth of MB models, showing their selective dependency on KDM2B.

Overall the manuscript is well written and presented, walking readers through the overall map of chromatin marks, differences among MB subgroups, and then focuses on the EnhBiv marks that are enriched in Grp3/4-MB. It then focuses on KDM2B, performing loss of function studies to demonstrate its role in supporting Grp3/4-MB tumor growth and maintenance, as well as mechanistic studies for its role at these EnhBiv locations, and repression of neurodevelopmental genes.

I didn't have any major concerns with the manuscript or data presented. The one main obvious question that remains from the manuscript is what causes the elevated expression (or maintained high expression) of KDM2B in Grp4-MB (and maybe in Grp3?). It is touched on in the discussion, but additional data related to KDM2B expression in human brain development may help further support how it comes about if there are no GOF activation mutations. Do the proposed cellular origin of Grp3/4-MBs display high expression of KDM2B? Is it a gene that just doesn't get turned off?

Moreover, does KDM2B expression correlate or overlap with samples that have other known mutations mentioned in the discussion (GFI, KMT2D, etc)? Again, this is mentioned in the discussion, so there may be some correlation (or anti-correlation) with expression or mutations.

Last, one suggestion, would be gain of function studies to further support the activity of KDM2B. These may complement the more in depth LOF studies that the authors have performed, but also may be somewhat redundant and difficult to accomplish in a relevant model system. Or expression of the different isoforms which the authors discuss may have varying activities?

Overall the manuscript provides a detailed story that encompasses going from genetic/epigenetic profiling to mechanistic studies which reveal a therapeutic vulnerability (KDM2B). While targeting this vulnerability still remains a challenge, future research and advancements can address this and other challenges and questions that remain.

Version 1:

Decision Letter:

Our ref: NG-A65725R

29th Apr 2026

Dear Paul,

Thank you for submitting your revised manuscript "Divergent medulloblastoma chromatin states disclose KDM2B as a selective dependency" (NG-A65725R). It has now been seen by the original referees and their comments are below. The reviewers find that the paper has improved in revision, and therefore we'll be happy in principle to publish it in Nature Genetics, pending minor revisions to satisfy the referees' final requests (please note the request from Reviewer #1 to integrate reviewer figures in the paper) and to comply with our editorial and formatting guidelines.

If the current version of your manuscript is in a PDF format, please email us a copy of the file in an editable format (Microsoft Word or LaTex)-- we can not proceed with PDFs at this stage.

We are now performing detailed checks on your paper and will send you a checklist detailing our editorial and formatting requirements soon. Please do not upload the final materials and make any revisions until you receive this additional information from us.

Thank you again for your interest in Nature Genetics Please do not hesitate to contact me if you have any questions.

Sincerely,

Safia Danovi, PhD
Senior Editor, Nature Genetics

ORCiD: 0009-0007-7822-5479

Reviewer #1 (Remarks to the Author):

The authors have addressed my questions and concerns. I suggest that the rebuttal figures be appropriately integrated into the manuscript, as they would provide a more comprehensive presentation of the study and offer additional insight into the function of KDM2B in medulloblastoma.

Reviewer #2 (Remarks to the Author):

This study by Northcott and colleagues is an important contribution to pediatric cancer and cancer epigenetics. I appreciate the authors extensive efforts to respond to reviewer comments. The core finding of KDM2B dependency in MB subtype is a strong and important result. The authors might consider softening mechanistic claims on downstream mechanisms, given the complexity of the area. I recommend acceptance to NG.

Reviewer #3 (Remarks to the Author):

The authors have done a thorough job of addressing the minor concerns I brought up during the initial review. I don't have any other comments or concerns. The revised manuscript includes new experimental data that strengthens the story for readers.

Open Access This Peer Review File is licensed under a Creative Commons Attribution 4.0 International License, which permits use, sharing, adaptation, distribution and reproduction in any medium or format, as long as you give appropriate credit to the original author(s) and the source, provide a link to the Creative Commons license, and indicate if changes were made.
In cases where reviewers are anonymous, credit should be given to 'Anonymous Referee' and the source.
The images or other third party material in this Peer Review File are included in the article's Creative Commons license, unless indicated otherwise in a credit line to the material. If material is not included in the article's Creative Commons license and your intended use is not permitted by statutory regulation or exceeds the permitted use, you will need to obtain permission directly from the copyright holder.
To view a copy of this license, visit https://creativecommons.org/licenses/by/4.0/

Table of Contents

Reviewer #1 (Remarks to the Author) **2**
Comment 1: EnhBiv vs TssBiv distinction **2**
Comment 2: Group 3-specific chromatin state **4**
Comment 3: H3K36me2 signal across the subgroups of MB **6**
Comment 4: H3K27me3 difference among SHH, Group 3 and Group 4 PDXs **6**
Comment 5: EnhBiv and KDM2B correlation across subgroups **8**
Comment 6: Cell fate after KDM2B deletion **9**
Comment 7: Impact of KDM2B perturbation on mouse survival **11**
Comment 8: Effect of KDM2B-dTAG degradation on growth of Group 4 models **13**
Comment 9: Overlap of KDM2B-regulated genes and EnhBiv signature genes **13**
Comment 10: Chromatin state profiling in Group 4 PDX models **16**
Comment 11: H3K36me2 changes following KDM2B deletion **17**
Comment 12: The impact of KDM2B perturbation on H3K27me3 and H3K4me1 .... **19**
Comment 13: The role of PRC1 on KDM2B-controlled EnhBiv state **21**
Comment 14: KDM2B genetic knockout and/or transgenic mouse models **26**
Comment 15: The impact of EZH2 perturbation on KDM2B-controlled EnhBiv state **26**

Reviewer #2 (Remarks to the Author) **27**
Comment 1 – Selectivity of KDM2B Dependency **28**
Comment 2 – EnhBiv vs TssBiv distinction **32**
Comment 3 – Mechanistic Link Between KDM2B and PRC2 .... **36**
Comment 4 – Neurodevelopmental Gene Impact **46**
Minor comments **48**

Reviewer #3 (Remarks to the Author) **51**
Comment 1 – Causes of high KDM2B expression in Group 4 MB **52**
Comment 2 – Correlation between KDM2B expression and genetic alterations **54**
Comment 3 – KDM2B gain-of-function studies **55**
References **59**

Referee expertise:

Referee #1: MB epi/genomics

Referee #2: brain tumour epigenetics

Referee #3: MB targets

Reviewers' Comments:

Reviewer #1:
Remarks to the Author:
In the manuscript, Tao et al. identified a chromatin state of a bivalent/poised enhancer (EnhBiv) enriched at the promoters of neurodevelopmental genes in Group 4 medulloblastoma (MB). They further determined that KDM2B, a histone lysine demethylase, is highly expressed in Group 4 MB and can occupy the promoter regions of EnhBiv-regulated genes. CRISPR-mediated knockout and protein degradation of KDM2B inhibited MB cell growth both in vitro and in vivo. Further validations revealed that KDM2B controls the EnhBiv chromatin state in Group 4 MB by binding to EZH2. The manuscript provides new insights into the epigenetic mechanisms of subgroup determination in MB to some extent. The following comments may improve the quality of the manuscript:

Response:
We thank Reviewer 1 for the detailed and constructive comments and appreciate the positive remarks regarding our study. In response to these comments, we performed extensive additional experimental and computational analyses, which strengthen the mechanistic framework, clarify the scope of our conclusions, and substantially improve the manuscript. Importantly, our interpretation is intentionally limited to subgroup-specific epigenetic mechanisms and vulnerabilities in medulloblastoma (MB) rather than subgroup determination. The new data do not support a role for KDM2B in determining subgroup identity, but instead further reinforce KDM2B as a selective dependency in Group 3 and Group 4 MB and strengthen the mechanistic link between KDM2B, EnhBiv state, and Polycomb-mediated regulation of gene expression.

1) It is confusing that a bivalent enhancer state, based on H3K4me1 and H3K27me3, was enriched in Group 4 MB but was marked at the gene promoter regions. What are the promoter-poised states across the four subgroups of MB based on H3K4me3 and H3K27me3?

Response:

1. We thank the reviewer for this question. In the Roadmap 18-state model, the bivalent enhancer state (EnhBiv) is defined by co-enrichment of the enhancer mark H3K4me1 and the repressive mark H3K27me3 but lacks the H3K4me3 mark associated with the transcriptional start site (TSS). In contrast, the bivalent TSS state (TssBiv; akin to 'promoter-poised' state) is defined by co-enrichment of H3K4me3, H3K27me3, and H3K4me1[1,2]. Using this framework, we found that EnhBiv was significantly enriched in Group 3 and Group 4 MB relative to WNT and SHH tumors,

whereas TssBiv showed comparable genome-wide coverage across all four subgroups (Rebuttal Letter Fig. 1a,b).

2. To address the reviewer's point regarding the apparent promoter localization of EnhBiv, we performed additional genomic enrichment and metagene analyses in the revised manuscript. When promoters were defined as TSS ± 5 kb, both EnhBiv and TssBiv overlapped promoter-proximal regions (Rebuttal Letter Fig. 1c,d). However, examination of average signal distributions revealed that TssBiv was sharply enriched at the immediate TSS (±2 kb), whereas EnhBiv was preferentially enriched in regions flanking the TSS (Rebuttal Letter Fig. 1e). Importantly, the abundance of bivalent promoter states in the TSS ± 5 kb region did not differ across subgroups, while EnhBiv showed increased coverage in Group 3 and Group 4 tumors (Rebuttal Letter Fig. 1f). These findings suggest that, although EnhBiv overlaps promoter-proximal regions, its spatial distribution and histone mark composition distinguish it from classical poised promoters. To capture this promoter-flanking EnhBiv state, we defined promoters as TSS ± 5 kb in this study, encompassing both canonical promoters and nearby regulatory elements.

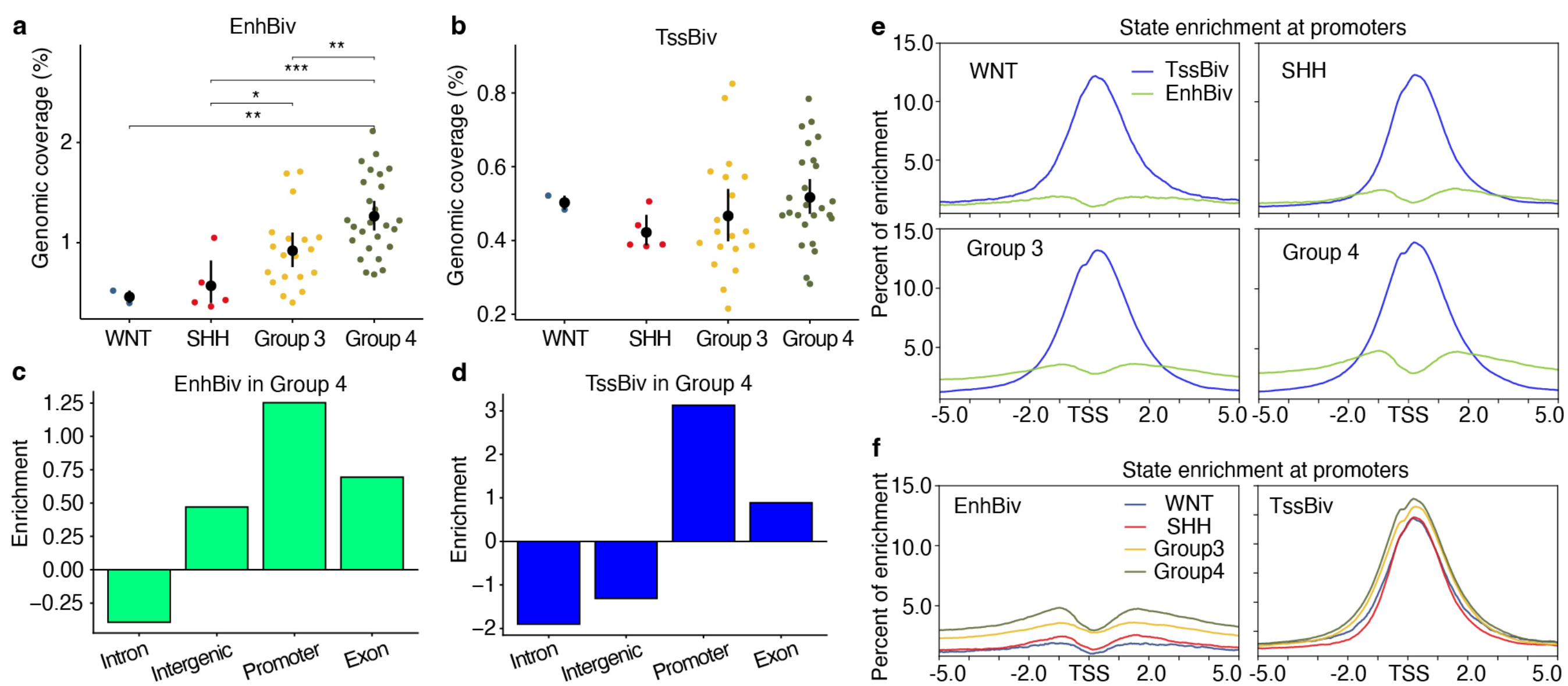

Rebuttal Letter Fig. 1 (from Fig. 2 and Extended Data Fig. 3). a and **b**, Genomic coverage of the EnhBiv (**a**) and TssBiv (**b**) states among MB subgroups. Black dots indicate median and bars indicate 1.5 × interquartile range (IQR; 25th–75th percentiles). WNT, n = 2; SHH, n = 5; Group 3, n = 19; Group 4, n = 26. *, $P_{adj} < 0.05$; **, $P_{adj} < 0.01$; ***, $P_{adj} < 0.001$ determined by Mann-Whitney U-test. **c** and **d**, Genomic enrichment of the EnhBiv (**c**) and TssBiv (**d**) states in Group 4-MB. n = 50,945 regions for EnhBiv and n = 51,710 for TssBiv. **e**, Distribution of EnhBiv and TssBiv states at the TSS ± 5kb promoter window of protein coding genes (n = 21,284) in MB subgroups. **f**, Enrichment of EnhBiv and TssBiv states at the TSS ± 5kb promoter window of protein coding genes (n = 21,284) comparing MB subgroups.

2) The conclusion in Figure 2 suggests that the EnhBiv state in Group 4 represents a less differentiated state, keeping cells in a progenitor status. Group 3 is generally considered to stem from neural progenitor cells, and both Group 3 and Group 4 share a unified cell of origin but exist along a continuum of developmental steps. What chromatin state trajectory determines the subgroup tumorigenesis? For instance, what is the Group 3-specific chromatin state, and how is it related to the EnhBiv state in Group 4?

Response:

1. We appreciate the insightful suggestion to examine how chromatin states differ across the Group 3 and Group 4 developmental continuum. Chromatin states control lineage specification, and the aberrant regulation of these states has been recurrently linked to tumorigenesis[2-5]. Unfortunately, due to the extreme scarcity of fetal cerebellar tissue, it is not feasible for us to profile chromatin states during human rhombic lip (RL) development to directly evaluate the chromatin state trajectory in developmental lineages underlying Group 3/4-MB.

2. We identified EnhBiv and ReprPC as the only chromatin states significantly enriched in Group 3 and Group 4 compared to SHH MB. Group 4 showed the highest enrichment of the EnhBiv state while Group 3 had the highest enrichment of the ReprPC state (Rebuttal Letter Fig. 2a,b). Although both states are repressive and share enrichment of H3K27me3, they preferentially localized to different genomic regions: Group 4 EnhBiv was enriched in promoters and Group 3 ReprPC was enriched in intergenic regions (Rebuttal Letter Fig. 2c,d). We focused on Group 4 EnhBiv-marked regions overlapping promoters and examined how chromatin state diverged in other subgroups. In Group 3, ~40% of these regions were marked by ReprPC state (Rebuttal Letter Fig. 2e). *PAX3*, a transcription factor regulating neurogenesis[6-8], was repressed in both Group 3 and Group 4 compared with SHH and WNT (Rebuttal Letter Fig. 2f). The promoter of *PAX3* was predominantly occupied by EnhBiv state in Group 4 and ReprPC state in Group 3. Although this region was covered by H3K27me3 in both subgroups, we observed a stronger H3K4me1 deposition in Group 4 compared to Group 3, which contributed to a bivalent state in Group 4 (Rebuttal Letter Fig. 2g).

3. Although transcriptionally similar, Group 3 and Group 4 exhibit a differentiation continuum: Group 3 is more progenitor-like and Group 4 is more differentiated based on molecular profiling[9-11]. We ranked Group 3 and Group 4 by Group 4 score determined by DNA methylation array and examined the enrichment of EnhBiv and ReprPC states along the Group 3-Group 4 differentiation continuum. EnhBiv coverage positively correlated with Group 4 score. In contrast, ReprPC was negatively correlated with the score (Rebuttal Letter Fig. 2h).

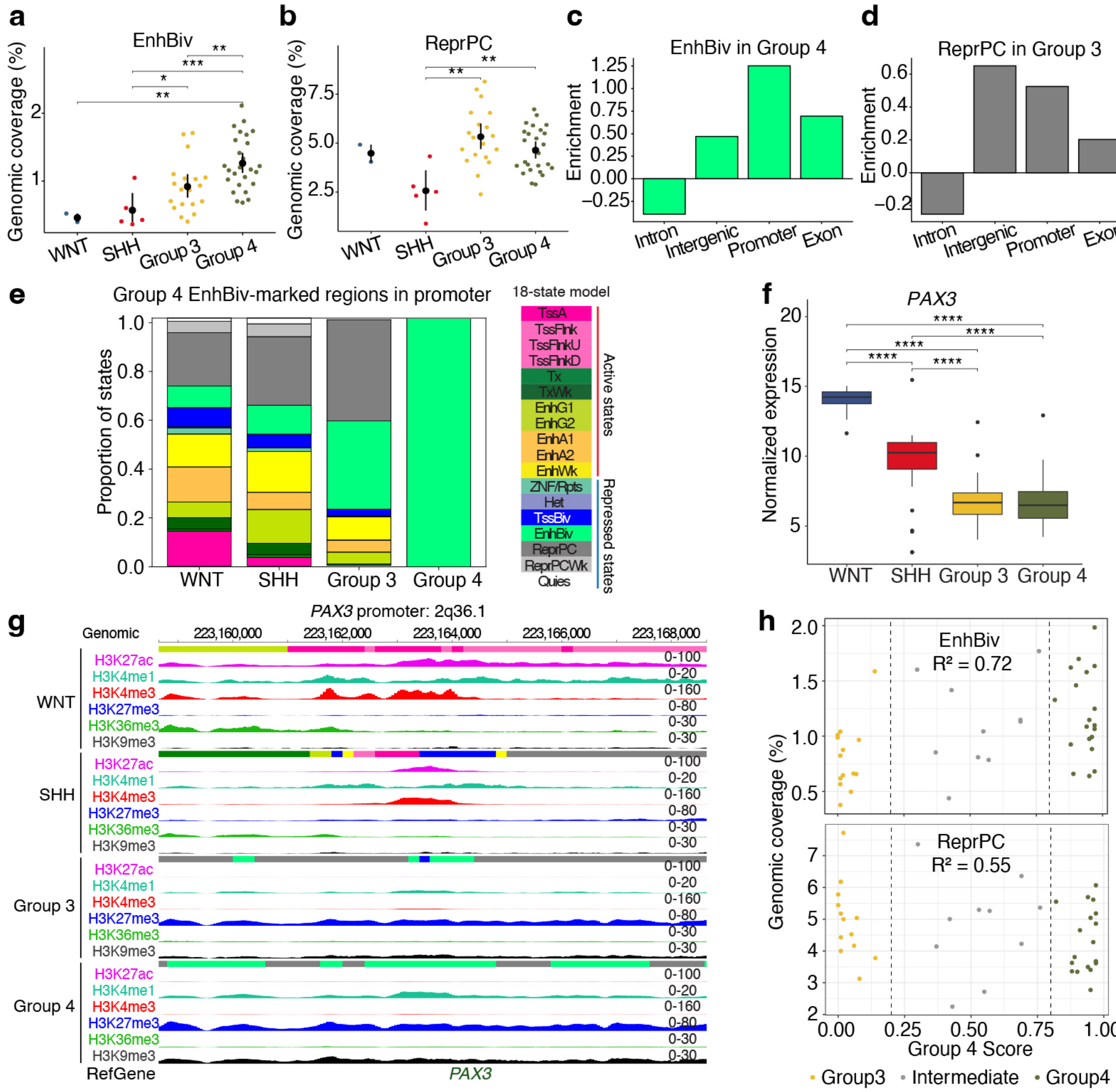

Rebuttal Letter Fig. 2 (from Fig. 2 and Extended Data Fig. 2 and 3). **a** and **b**, Genomic coverage of the EnhBiv (**a**) and ReprPC (**b**) states among MB subgroups. Black dots indicate median and bars indicate 1.5 x IQR. WNT, n = 2; SHH, n = 5; Group 3, n = 19; Group 4, n = 26. *, $P_{adj} < 0.05$; **, $P_{adj} < 0.01$; ***, $P_{adj} < 0.001$ determined by Mann-Whitney U-test. **c**, Genomic enrichment of the EnhBiv state in Group 4-MB. n = 50,945 regions. **d**, Genomic enrichment of the ReprPC state in Group 3-MB. n = 111,048 regions. **e**, Chromatin state proportions of Group 4 EnhBiv-marked regions (n = 5,420) within the promoter window for MB subgroups. **f**, *PAX3* expression in MB subgroups. WNT, n = 15; SHH, n = 46; Group 3, n = 41; Group 4, n = 65. Boxes represent the IQR, center line indicates the median, and whiskers extend to the most extreme data points within 1.5 × IQR. Dots indicate outliers beyond 1.5 × IQR. Statistical significance was assessed using the two-sided t-test, with *P* adjusted by the Holm method. ****, $P_{adj} < 0.0001$. **g**, Merged histone tracks and aggregated chromatin state at the *PAX3* promoter in MB subgroups (WNT, n = 2; SHH, n = 5; Group 3, n = 19; Group 4, n = 26). **h**, Scatterplot showing EnhBiv (top) and ReprPC (bottom) genomic coverage along Group 3 to Group 4 differentiation continuum. Group 3/4 MBs (n = 43) were ranked by Group 4 score from left to right. Dashed line indicates cutoff for calling Group 3 (< 0.2), Group 4 (> 0.8), or intermediate (between 0.2-0.8). EnhBiv genomic coverage was positively correlated with Group 4 score as determined by linear regression analysis ($R^2 = 0.72$, $P = 3.14 \times 10^{-13}$). ReprPC genomic coverage was negatively correlated with Group 4 score ($R^2 = 0.55$, $P = 7.42 \times 10^{-9}$).

3) KDM2B has been reported to encode an H3K36me2-specific demethylase. What is the H3K36me2 signal across the subgroups of MB, particularly in Group 4?

Response:

We thank the reviewer for their suggestion that we addressed by performing CUT&RUN for H3K36me2 in SHH (n=2), Group 3 (n=3), and Group 4 (n=3) patient-derived xenograft (PDX) models. Localization and enrichment of this mark did not correlate with KDM2B peaks in MB subgroups (Rebuttal Letter Fig. 3a). H3K36me2 genome-wide peak intensity was higher in Group 3 than SHH and Group 4 PDXs (Rebuttal Letter Fig. 3b).

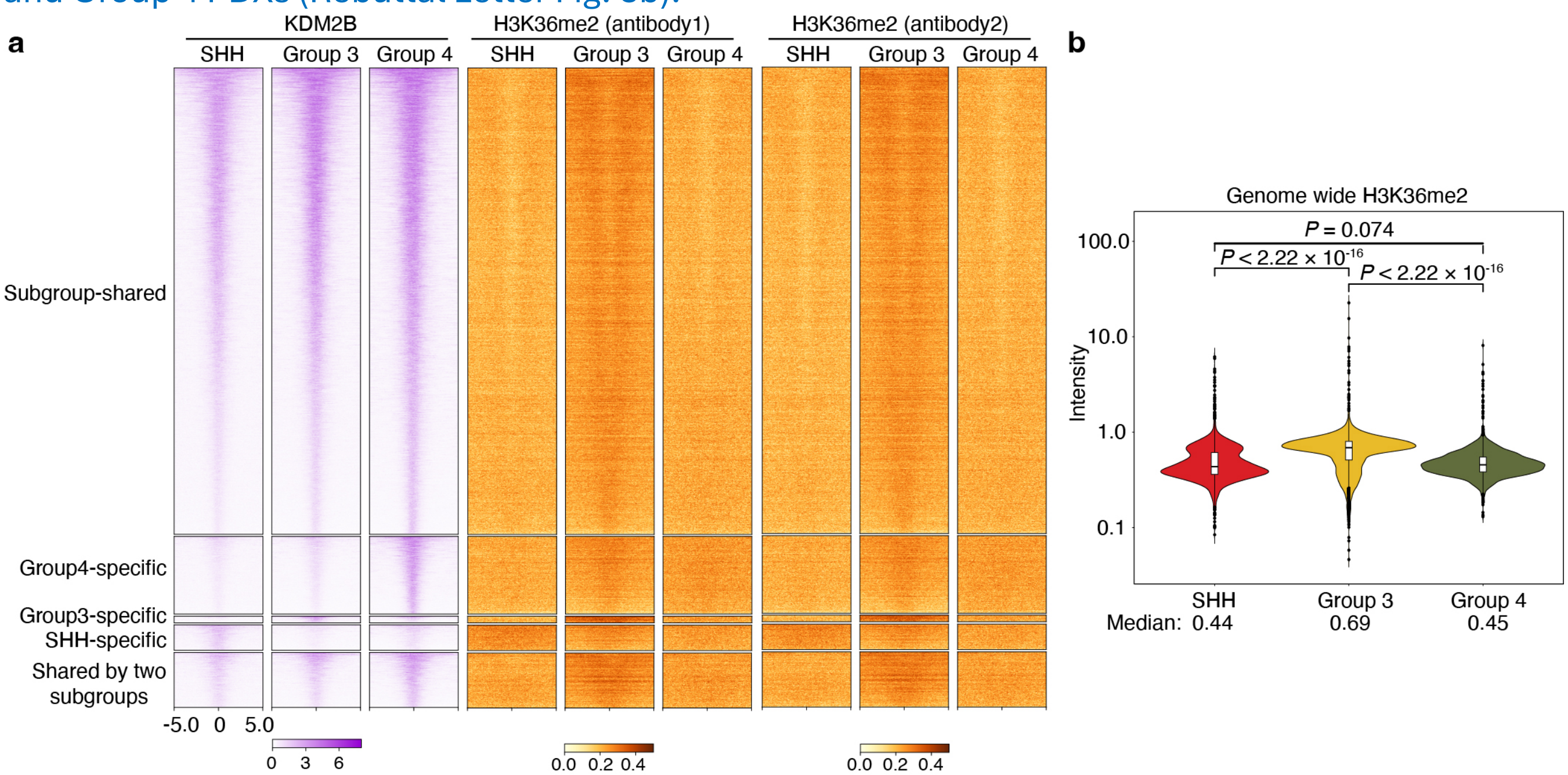

Rebuttal Letter Fig. 3. **a**, CUT&RUN analysis of KDM2B and H3K36me2 (determined by two different antibodies) in MB PDXs (SHH, n = 2; Group 3, n = 3; Group 4, n = 3). Heatmap shows enrichment of the indicated marks at KDM2B-bound regions: subgroup-shared, n = 21,590; Group 4-specific, n = 3,591, Group 3-specific, n = 307; SHH-specific, n = 1,175; shared by two subgroups, n = 2,551. **b**, Violin plot quantifying global H3K36me2 peak intensity in MB PDXs (SHH, n = 2; Group 3, n = 3; Group 4, n = 3). Violin shows kernel probability density estimation. Boxes represent the IQR, center line indicates the median, and whiskers extend to the most extreme data points within 1.5 × IQR. Dots indicate outliers beyond 1.5 × IQR. *P* values were determined by Wilcoxon test.

4) Why is there no difference in the H3K27me3 mark among the three subgroups in Fig 3e?

Response:

1. We are grateful for the opportunity to clarify a potential point of confusion and improve the manuscript. The original Figure 3e showed enrichment of H3K4me1, H3K4me3, and H3K27me3 at 29,214 subgroup-shared or subgroup-specific KDM2B-bound regions detected in MB PDXs. At these regions, H3K27me3 did not show strong correlation with KDM2B binding and was not selectively enriched in any MB subgroup. We believe this is because KDM2B broadly occupies non-methylated CpG-enriched promoters, where it can contribute to both gene activation and

repression[12-14], but only a subset of these promoters are subject to KDM2B-dependent repression via PRC2 recruitment, H3K27me3 deposition, and EnhBiv state formation. Consistent with this model, our gene expression and CUT&RUN analyses revealed derepression of 1,038 genes, concurrent with a profound reduction in EZH2 recruitment and H3K27me3 deposition at these specific promoters following KDM2B degradation in D283 cells. In contrast, global reduction of EZH2 or H3K27me3 was modest (discussed in questions #12 and 13).

2. To examine the association between KDM2B, H3K27me3, and EnhBiv in the cellular context of MB, we characterized EnhBiv-enriched promoters (defined by >10% EnhBiv coverage at the TSS ± 5kb region) in SHH, Group 3 and Group 4 MB using CUT&RUN data derived from MB PDX models (Rebuttal Letter Fig. 4a). We calculated average peak intensity of H3K27me3, KDM2B, and EZH2 in these regions. We found Group 4 EnhBiv enriched promoters had significantly higher average H3K27me3 intensity than those annotated in SHH or Group 3 (Rebuttal Letter Fig. 4a,b). Consistently, the average intensity of both KDM2B and EZH2 was highest in Group 4, followed by Group 3, and SHH regions showed the lowest signal (Rebuttal Letter Fig. 4a,c,d). These results confirmed the strong association among KDM2B, EZH2, H3K27me3, and the EnhBiv state in Group 3 and Group 4 relative to SHH MB. We have removed the original Fig. 3e and added this analysis to Fig. 3e,f, Fig. 6c,d and Extended Data Fig. 5f-h.

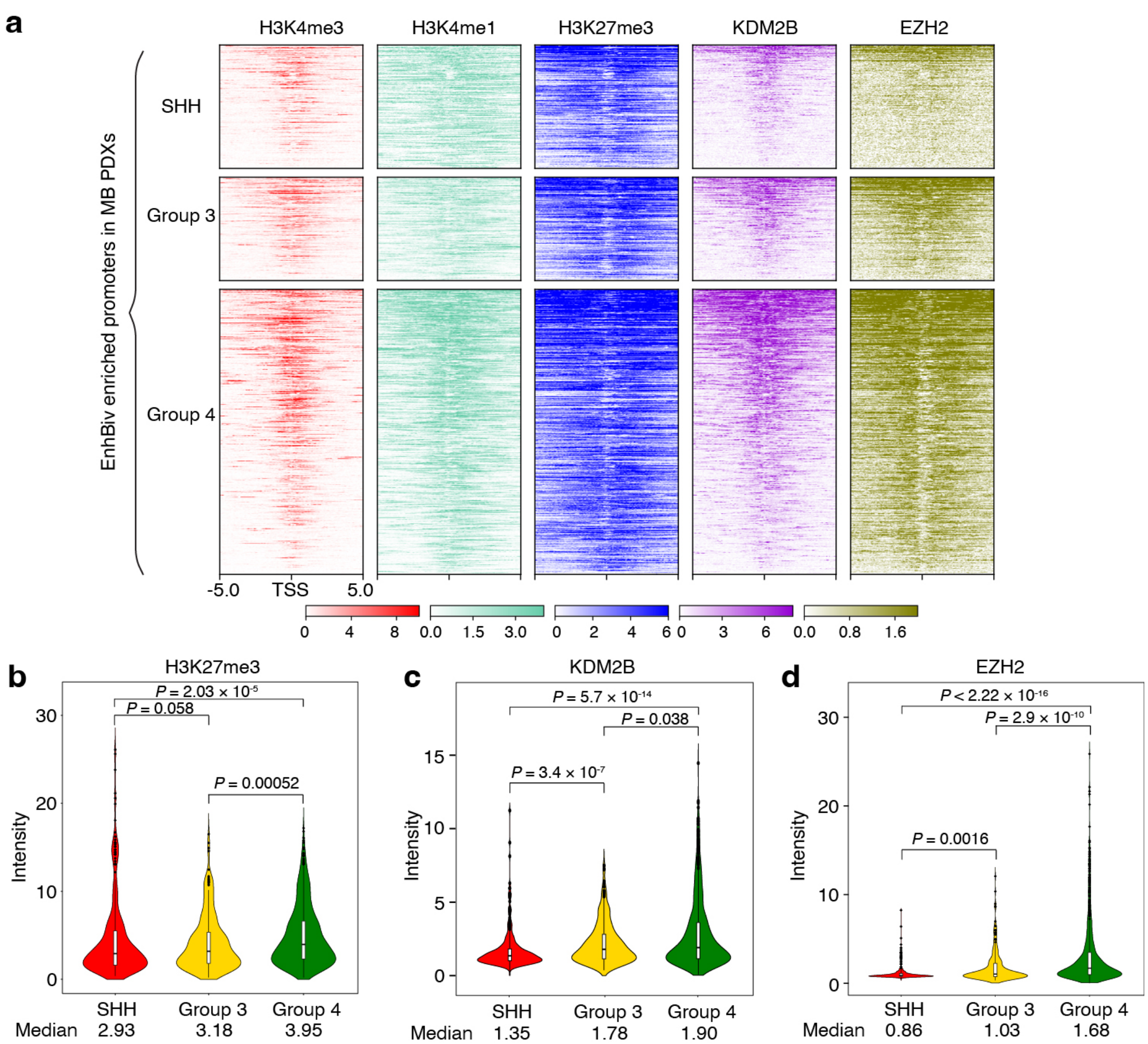

Rebuttal Letter Fig. 4 (from Fig. 3 and 6, and Extended Data Fig. 5). a, Heatmap shows enrichment of the indicated marks at EnhBiv-enriched promoters (SHH, n=313; Group 3, n=262, Group 4, n=723) in MB PDXs. **b-d**, Violin plot quantifying CUT&RUN peak intensity of H3K27me3 (**b**), KDM2B (**c**), and EZH2 (**d**) at EnhBiv-enriched promoters from **a**. Violin shows kernel probability density estimation. Boxes represent the IQR, center line indicates the median, and whiskers extend to the most extreme data points within 1.5 × IQR. Dots indicate outliers beyond 1.5 × IQR. *P* values were determined by Wilcoxon test.

5) Whether the significant enrichment of the EnhBiv state at KDM2B-bound promoters compared with KDM2B-deprived promoters occurred in Group 3 and SHH groups?

Response:

We appreciate the reviewer's suggestion to examine the enrichment of EnhBiv at KDM2B-bound promoters compared to KDM2B-deprived promoters across MB subgroups. Although EnhBiv was significantly enriched at KDM2B-bound promoters compared to KDM2B-deprived promoters regardless of subgroup, this enrichment was highest for Group 4, followed by Group 3, and finally SHH (Rebuttal Letter Fig. 5). This supports our model where KDM2B promotes the EnhBiv state, with the highest *KDM2B* expression and EnhBiv state enrichment observed in Group 4 MB. This analysis clarified the association between KDM2B and EnhBiv state enrichment across MB subgroups. We have replaced the orginal Fig. 3f with a modified figure focusing on the comparison of EnhBiv enrichment at KDM2B-bound promoters among MB subgroups (new Fig. 3g).

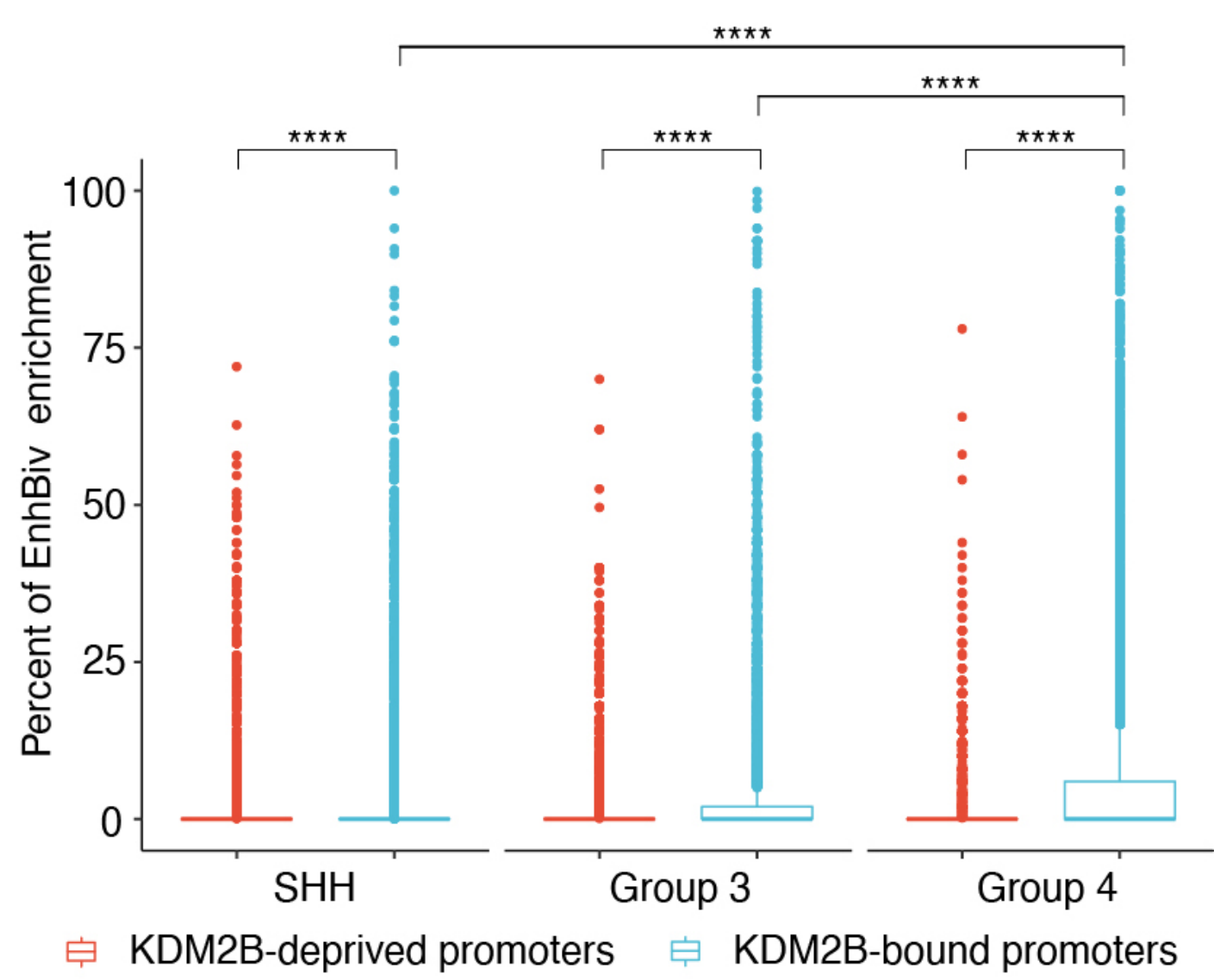

Rebuttal Letter Fig. 5 (adapted from Fig. 3g). Box plot showing enrichment of the EnhBiv state at KDM2B-bound promoters compared with KDM2B-deprived promoters in MB PDXs. Only promoters that are targeted by EnhBiv state are included in the analysis. KDM2B-bound promoters: SHH, n = 6,574; Group3, n = 7,388; Group 4, n = 8,228. KDM2B-deprived promoters: SHH, n = 2,966; Group 3, n = 2,152; Group 4, n = 1,312. KDM2B signal and EnhBiv state were derived from CUT&RUN analysis of KDM2B, H3K4me3, H3K4me1, and H3K27me3 in MB PDXs (SHH, n = 2; Group 3, n = 3; Group 4, n = 3). ****, $P < 0.0001$ determined by two-sided t-test. Boxes represent the IQR, center line indicates the median, and whiskers extend to the most extreme data points within 1.5 × IQR. Dots indicate outliers beyond 1.5 × IQR.

6) What is the cell fate after KDM2B deletion in the in vitro and in vivo competition assays shown in Figure 4? It would be helpful to examine cell differentiation, apoptosis/death, senescence, and so on.

Response:

1. We thank the reviewer for the excellent recommendation to examine how loss of KDM2B impacts cell viability in the Group 3/4 MB context. Our *in vitro* and *in vivo* competition assays indicate KDM2B is a strong Group 3 and Group 4 selective dependency. Competition assays are not ideal for assessing cell fate changes, as *KDM2B* knockout (KO) was only induced in a proportion of cells (40-60%), which were depleted over time due to their reduced viability compared with control cells. Therefore, to answer this question, we turned to our powerful D283 KDM2B-dTAG system, which allows rapid and homogenous KDM2B depletion, and performed RNA-seq following KDM2B degradation. Consistent with our original *KDM2B* KO RNA-seq dataset, we observed robust de-repression of gene expression following KDM2B degradation and significant correlation between the two RNA-seq datasets (Rebuttal Letter Fig. 6a,b). We systematically examined the enrichment of neuronal differentiation, proliferation, apoptosis, and senescence gene sets using this new RNA-seq data and found that KDM2B degradation predominantly induced differentiation and reduced cell proliferation (Rebuttal Letter Fig. 6c,d). Only modest changes were observed in genes related to apoptosis or senescence (Rebuttal Letter Fig. 6c).

2. To experimentally validate our findings, we conducted *in vitro* assays for proliferation (EdU incorporation) and apoptosis (Annexin V staining). Following KDM2B degradation, we observed a strong decrease in the percentage of proliferating cells (~21% reduction compared to controls at day 9) (Rebuttal Letter Fig. 6e). Apoptotic cells increased ~4% compared to controls and remained low overall (<15%) (Rebuttal Letter Fig. 6f). These results suggest that KDM2B depletion mainly inhibits proliferation, consistent with Gene set enrichment analysis (GSEA) findings.

3. It is technically challenging to assess cell fate *in vivo* by CRISPR KO because *KDM2B* is a strong dependency depleting the targeted cells over time. To confirm the *in vitro* findings, we characterized tumors collected after *in vivo* dTAG treatment (experimental details in question #7). We identified reduced Ki-67 (proliferation marker) staining following KDM2B depletion (Rebuttal Letter Fig. 6g,h). Cleaved caspase 3 (CC3, apoptosis marker) staining was not significantly different between dTAG-treated and control cells (Rebuttal Letter Fig. 6g,i). These results corroborated our extensive *in vitro* findings. Together, these results indicate that KDM2B maintains tumor growth through suppressing differentiation.

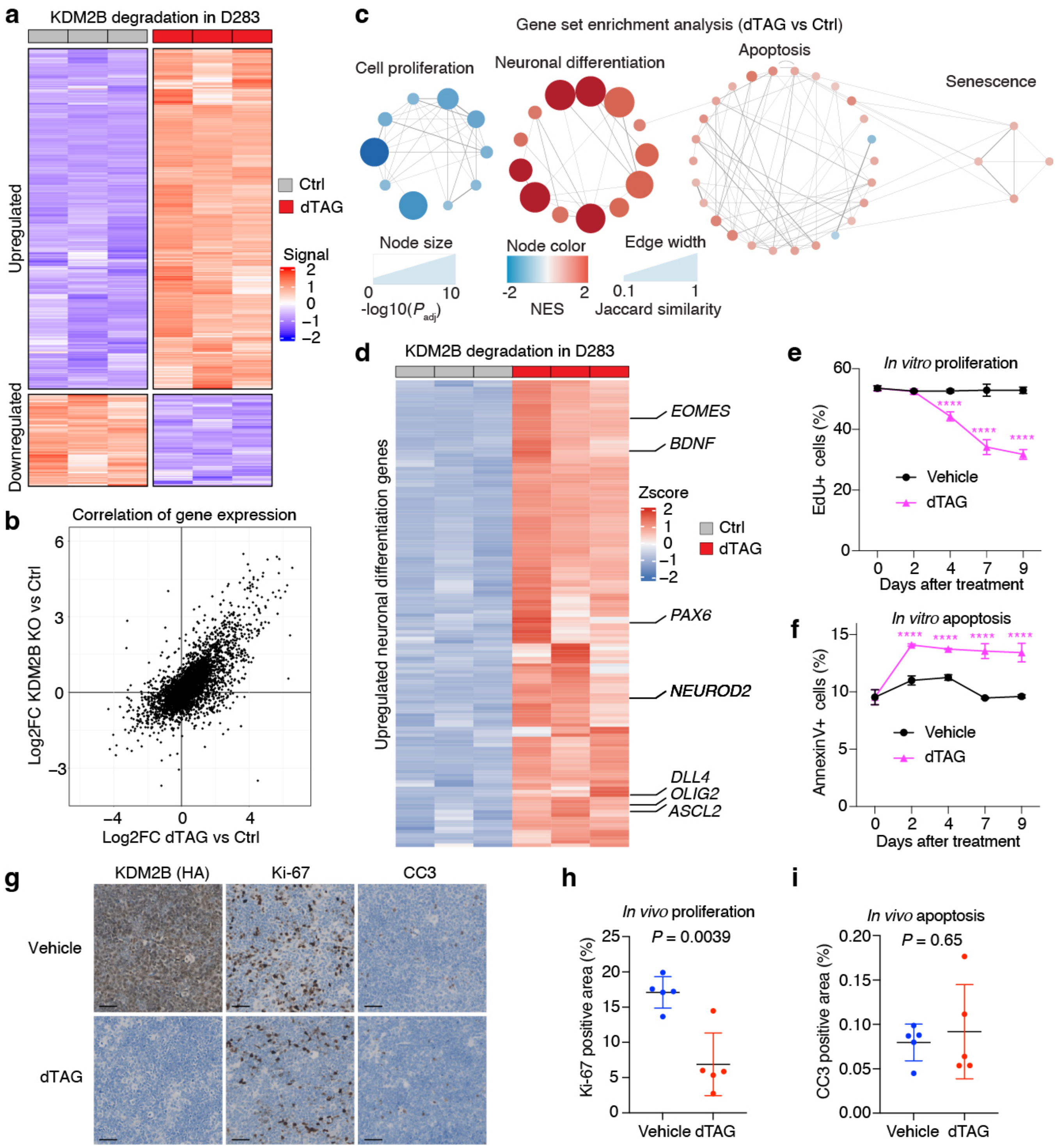

a
KDM2B degradation in D283
Upregulated
Downregulated
Ctrl
dTAG
Signal
2
1
0
–1
–2
b
Correlation of gene expression
Log2FC KDM2B KO vs Ctrl
Log2FC dTAG vs Ctrl
6
3
0
–3
–4
0
4
c
Gene set enrichment analysis (dTAG vs Ctrl)
Cell proliferation
Neuronal differentiation
Apoptosis
Senescence
Node size
0
10
-log10(P adj)
Node color
-2
2
NES
Edge width
0.1
1
Jaccard similarity
d
KDM2B degradation in D283
Upregulated neuronal differentiation genes
EOMES
BDNF
PAX6
NEUROD2
DLL4
OLIG2
ASCL2
Zscore
2
1
0
–1
–2
Ctrl
dTAG
e
In vitro proliferation
EdU+ cells (%)
60
40
20
0
****
****
****
Vehicle
dTAG
0
2
4
7
9
Days after treatment
f
In vitro apoptosis
AnnexinV+ cells (%)
15
10
5
0
****
****
****
****
Vehicle
dTAG
0
2
4
7
9
Days after treatment
g
KDM2B (HA)
Ki-67
CC3
Vehicle
dTAG
h
In vivo proliferation
P = 0.0039
Ki-67 positive area (%)
25
20
15
10
5
0
Vehicle
dTAG
i
In vivo apoptosis
P = 0.65
CC3 positive area (%)
0.20
0.15
0.10
0.05
0.00
Vehicle
dTAG

Rebuttal Letter Fig. 6 (from Fig. 4 and 5, and Extended Data Fig. 6 and 7). a, Heatmap of differentially expressed genes (upregulated, n = 1038; downregulated, n = 280) following KDM2B-dTAG degradation in D283. **b**, Positive correlation of gene expression changes (n = 18,087) following KDM2B loss by either CRISPR KO or degradation as determined by linear regression analysis ($R^2 = 0.55$, $P = 7.77 \times 10^{-5509}$). **c**, Enrichment of cell proliferation, neuronal differentiation, apoptosis and senescence gene sets following KDM2B degradation. Nodes were colored by NES and sized by -log10(P_{adj}) determined by permutation test (n = 1,000) with Benjamini-Hochberg FDR correction. Edge width describes Jaccard similarity. **d**, Heatmap of neuronal differentiation genes (n = 140) significantly upregulated following KDM2B degradation in D283. Selected neuronal differentiation genes are labeled. **e** and **f**, *In vitro* assays of proliferation (**e**) and apoptosis (**f**) for D283 KDM2B-dTAG line. Values represent mean ± standard deviation (SD) determined from three technical replicates. Adjusted *P* was generated by two-way ANOVA with Šidák correction. ****, $P_{adj} < 0.0001$. Experiments were repeated at least two times. **g**, Immunohistochemistry staining of KDM2B (by HA-Tag antibody), Ki-67 and Cleaved caspase 3 (CC3) for tumors collected after *in vivo* dTAG treatment. Scalebar - 40 μm. **h** and **i**, Quantification of Ki-67 (**h**) and CC3 (**i**) in tumors treated with vehicle or dTAG (n=5/group) *in vivo*. Center line and error bar indicates mean ± SD. *P* was calculated by two-tailed t-test.

7) What is the mouse survival difference between the controls and KDM2B KO groups?

Response:

1. We thank the reviewer for this question. In our initial *in vivo* competition assays, only a subset of Group 4 MB cells (~40-60%) were designed to be transduced, allowing uninfected cells to rapidly outcompete *KDM2B* KO cells when loss of *KDM2B* reduced cellular fitness, making this approach incompatible with survival analyses. To overcome this limitation, we maximized transduction efficiency to ~90% prior to implantation. However, because Group 4 MB cells do not proliferate and survive poorly during culture, the transduced cells cannot be enriched *in vitro* prior to implantation. Tumors were still initiated with mixed populations. During long-term tumor growth, the remaining ~10% of untargeted cells progressively repopulated the tumor, as evidenced by a marked depletion of GFP+ cells (10–20% GFP+ remaining) in the endpoint tumors from the *KDM2B* KO group, whereas control tumors retained ~90% GFP+ cells (Rebuttal Letter Fig. 7a). As a result of these technical limitations, we did not observe a significant difference in mouse survival between DMB006 control and *KDM2B* KO groups over 4–8 months of *in vivo* growth (Rebuttal Letter Fig. 7b). However, the massive depletion of GFP+ cells following *KDM2B* KO confirms that *KDM2B* is a strong dependency in DMB006 despite the absence of a survival effect.

2. To directly assess the requirement for KDM2B for *in vivo* tumor growth under conditions that bypass these technical constraints, we used an orthogonal Group 3 D283 KDM2B-dTAG flank tumor model. Pharmacologic degradation of KDM2B significantly reduced tumor growth and prolonged survival (Rebuttal Letter Fig. 7c–e), providing independent *in vivo* validation that KDM2B is required for MB tumor maintenance.

Additionally, what are the pathological changes observed in these tumors after KDM2B KO, as shown in Figures 4f and 4g?

Response:

Endpoint tumors collected from *KDM2B* KO DMB006 mice contained only a small fraction of targeted cells (10–20%), precluding reliable assessment of pathological changes directly attributable to *KDM2B* loss. As an orthogonal approach, we performed H&E staining on tumors collected following *in vivo* KDM2B degradation using the dTAG system. In this setting, we did not observe overt histological differences compared to controls (Rebuttal Letter Fig. 7f).

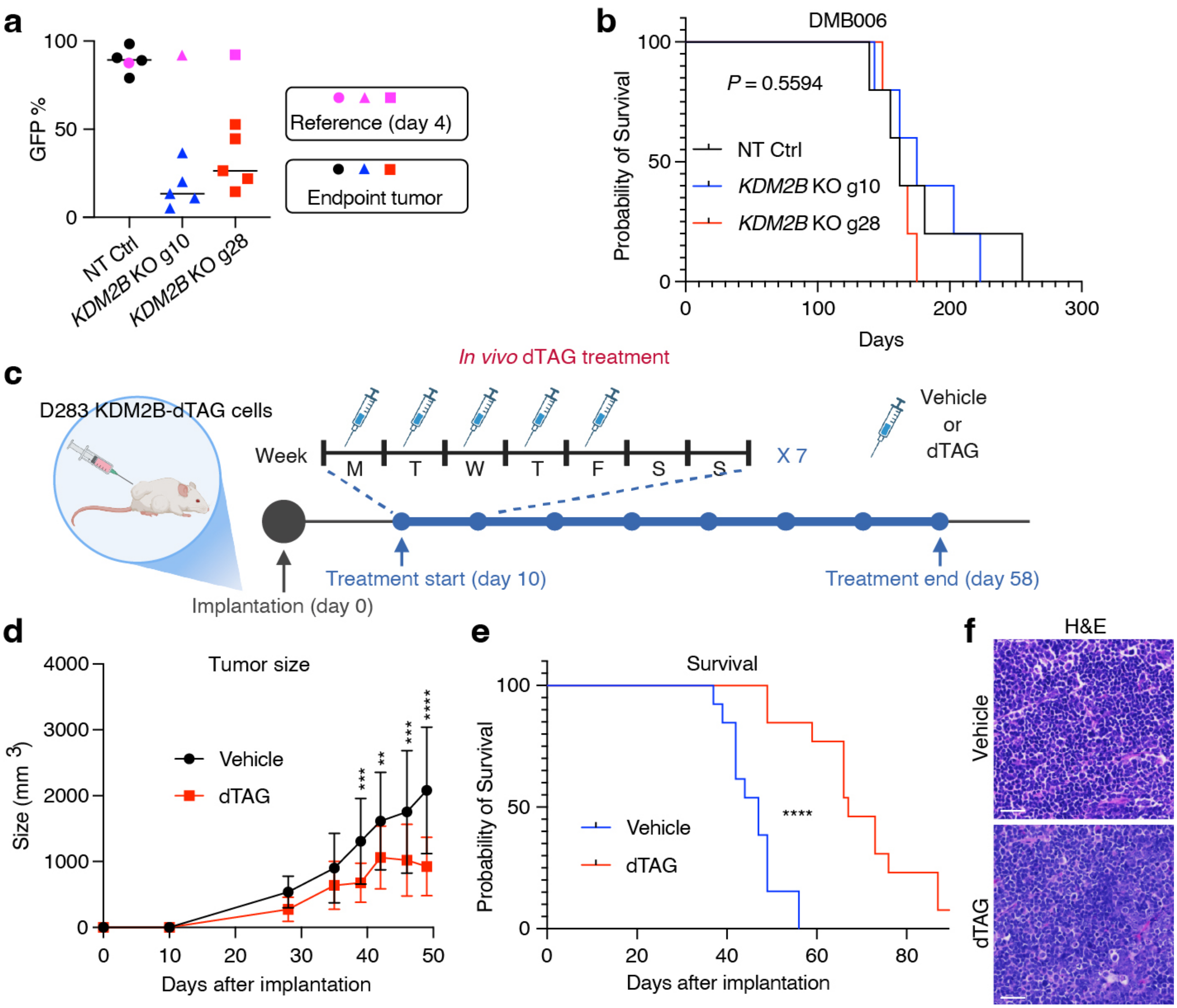

Rebuttal Letter Fig. 7 (from Fig. 4). a, Proportion of GFP+ cells from tumors collected at endpoint compared to transduced cells for implantation (reference). Group 4 DMB006 cells transduced with lentiviruses encoding either non-targeting (NT) control or *KDM2B* KO gRNAs (~90% transduction efficiency) were implanted into the cerebellum of immunodeficient mice. **b**, Survival of mice in **a**. *P* was determined by Log rank Mantel-Cox test. n=5 mice/group. **c**, Schematic of *in vivo* dTAG treatment. **d**, Tumor growth during *in vivo* dTAG treatment. Values represent mean ± SD (n = 13/group). Statistical significance was determined by two-way ANOVA. **, $P < 0.01$; ***, $P < 0.001$; ****, $P < 0.0001$. **e**, Survival of mice (n = 13/group) receiving *in vivo* dTAG treatment. ****, $P < 0.0001$ by Log rank Mantle-Cox test. **f**, H&E staining for tumors collected after *in vivo* dTAG treatment. Scalebar - 40 µm.

8) In vivo competition assay should be used to test dTAG-mediated KDM2B protein degradation in two Group 4 models (DMB006 and Med2312FH).

Response:

1. We appreciate the reviewer's interest in confirming KDM2B as a dependency in Group 4 MB and agree that the dTAG system is a powerful technique for examining this relationship. Current tagging technology is based on CRISPR-mediated homology-directed repair (HDR), which requires transfection of Cas9-gRNA RNP and HDR template into the cells, followed by clonal selection *in vitro*. As discussed in question #7 above, Group 4 MB models can only be propagated *in vivo*. Therefore, it was not technically feasible to generate dTAG lines and perform degradation studies in Group 4 models.

2. Based on the suggestion in question #7 above, we tested dTAG-mediated KDM2B protein degradation in the D283 flank tumor model and added this data corroborating KDM2B as a dependency *in vivo* to the manuscript. In the revised manuscript, we emphasize that the EnhBiv state was enriched in both Group 3 and Group 4 relative to SHH and WNT, and we expanded functional studies to establish *KDM2B* as a strong selective dependency in both subgroups.

9) How many genes overlap between KDM2B-regulated genes (Figure 5a) and Group 4 EnhBiv signature genes?

Response:

1. We thank the reviewer for this suggestion. As shown in Rebuttal Letter Fig. 8a,b, Group 4 EnhBiv signature genes showed increased expression after *KDM2B* KO in D283 (Group 3) and DMB006 (Group 4), suggesting that many of these genes might be regulated by KDM2B. Additionally, median expression of Group 4 EnhBiv signature genes was significantly increased following *KDM2B* KO in D283 (Rebuttal Letter Fig. 8c).

2. We checked the overlap between the 666 Group 4 EnhBiv signature genes and KDM2B-regulated genes (defined as differentially expressed following *KDM2B* KO in D283), including 2,993 upregulated (Log2FC > 0 and FDR < 0.05) and 2,366 downregulated (Log2FC < 0 and FDR < 0.05) genes. 186 (28%) of the EnhBiv signature genes overlapped with *KDM2B* KO upregulated genes, which was significantly higher than predicted by chance ($P = 1.87 \times 10^{-6}$, hypergeometric test). In contrast, only 36 (5%) of EnhBiv signature genes overlapped with *KDM2B* KO down-regulated genes, which was not significantly higher than by chance ($P = 1$; Rebuttal Letter Fig. 8d). These data support the hypothesis that KDM2B regulates EnhBiv-targeted genes in MB.

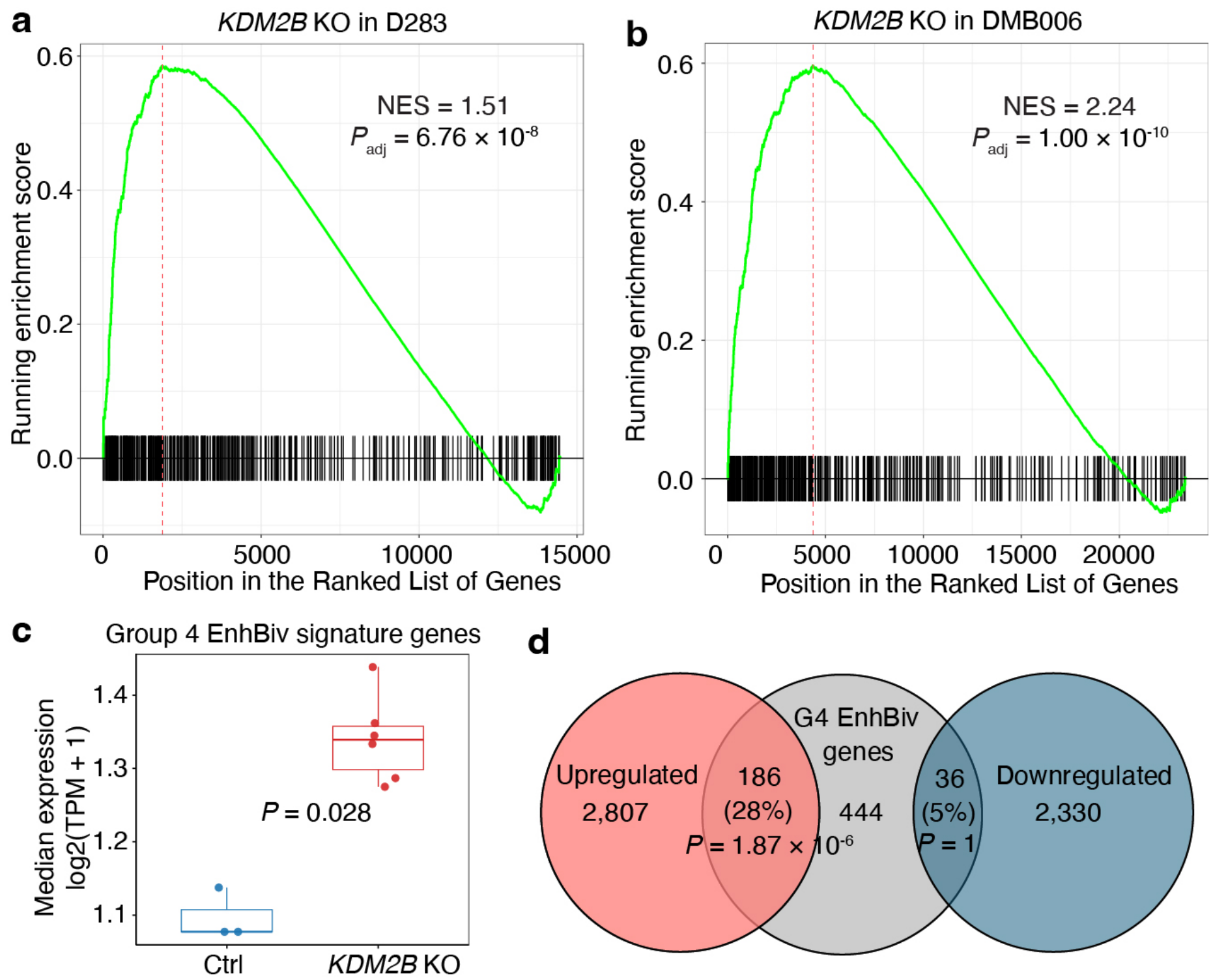

Rebuttal Letter Fig. 8. a and **b,** Enrichment analysis showing Group 4 EnhBiv signature genes (n = 666) were significantly upregulated following *KDM2B* KO in D283 (**a**) or in DMB006 (**b**). Significance was determined by permutation test with normalized enrichment score (NES) > 1.5 and $P_{adj} < 0.05$ (Benjamini-Hochberg correction) considered statistically significant. **c**, Median expression of Group 4 EnhBiv signature genes (n = 666) comparing control with *KDM2B* KO in D283 cells. Boxes represent the IQR, center line indicates the median, and whiskers extend to the most extreme data points within 1.5 × IQR. TPM: Transcripts Per Million. **d**, Venn diagram depicting overlap between 666 Group 4 EnhBiv signature genes and genes upregulated (log2FC > 0, FDR < 0.05) or downregulated (log2FC < 0, FDR < 0.05) upon *KDM2B* KO in D283 cells. *P* determined by hypergeometric test.

Can altering KDM2B expression change the Group 4 EnhBiv signature genes and affect the tumor subgroup? For example, one might wonder if KDM2B overexpression in Group 3 and SHH cell lines could switch these cell lines to a Group-4-like subgroup.

Response:

1. We thank the reviewer for this interesting suggestion. To examine whether *KDM2B* overexpression can change EnhBiv signature gene expression and affect subgroup identity, we cloned *KDM2B* with an HA tag at its C terminus into a doxycycline (Dox) inducible (Tet-On) lentiviral vector expressing a GFP reporter and tested this overexpression system in our Group 3 D283 KDM2B-dTAG cell line. After viral infection and sorting GFP+ cells, we treated the cells with different concentrations of Dox in the presence of 500 nM dTAG for 24 h to ectopically induce KDM2B expression and degrade the endogenous KDM2B-dTAG protein. Immunoblotting showed that KDM2B was robustly induced by Dox and reached the highest expression at ~500 ng/ml (Rebuttal Letter Fig. 9a).

2. After validating the overexpression system, we transduced SHH-MB PDX (SJNBT3) cells with the Tet-On *KDM2B* lentivirus and sorted GFP+ cells. We treated the cells with 0 or 500 ng/mL doxycycline for six days and collected cell pellets for immunoblotting, RNA-seq, and DNA methylation array. Immunoblotting confirmed robust Dox-induced KDM2B expression in this cell line (Rebuttal Letter Fig. 9b). Analysis of DNA methylation array data indicated overexpression of *KDM2B* did not change subgroup score (based on MNP v12.8[15]) or clustering in UMAP space (Rebuttal Letter Fig. 9c,d). This data suggests that *KDM2B* overexpression alone is insufficient to shift tumor cells from SHH to Group 4. We only detected two differentially expressed genes in this system: Dox-induced *KDM2B* and *SYNJ2BP-COX16* (Rebuttal Letter Fig. 9e). These data suggest that KDM2B regulates gene expression in a context-dependent manner.

3. To check whether *KDM2B* overexpression affected cell growth, we performed a competition assay in SJNBT3 and found no impact on cell growth during 13 days of *in vitro* culture (Rebuttal Letter Fig. 9f). Because KDM2B overexpression did not alter SHH tumor profiles and KDM2B KO/degradation selectively suppresses growth of Group 3 and 4 tumors, we conclude that *KDM2B* does not determine subgroup identity but instead promotes tumor maintenance in Group 3 and Group 4 MB.

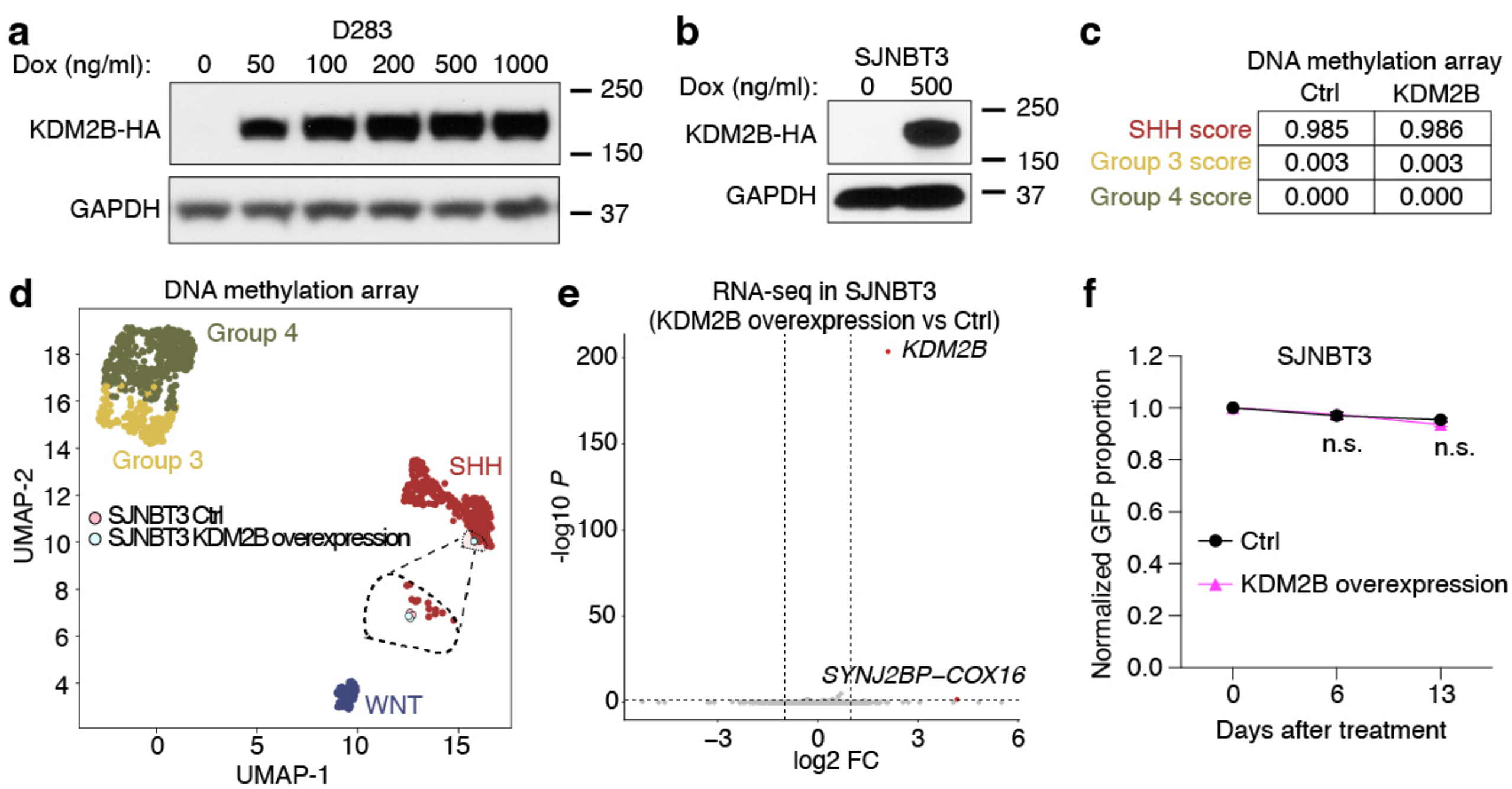

Rebuttal Letter Fig. 9. **a**, Immunoblot showing ectopically expressed KDM2B (detected by HA-Tag antibody). **b**, Immunoblot detecting Dox-induced KDM2B overexpression in SHH MB SJNBT3 cells transduced with Tet-on *KDM2B* lentivirus. **c**, DNA methylation-derived subgroup scores for SJNBT3 cells with or without *KDM2B* overexpression. **d**, UMAP depicting DNA methylation-based clustering of patient tumors and SJNBT3 cells with or without *KDM2B* overexpression. Clustering was based on 2,000 most variable probes. WNT, n = 104; SHH, n = 320; Group 3, n = 209; Group 4, n = 472. **e**, Volcano plot showing differentially expressed genes determined by RNA-seq in SJNBT3 cells with or without *KDM2B* overexpression. **f**, *In vitro* competition assay in SJNBT3 cells with or without *KDM2B* overexpression. Values represent mean ± SD determined from two biological replicates. Adjusted *P* was generated by two-way ANOVA with Dunnett's correction. n.s. represents not significant.

10) KDM2B deletion in Group 3 cell line D283 showed modest changes in histone marks and EnhBiv enrichment (Fig. 5d-f). Global gene expression and chromatin landscape changes (cut&run) can be analyzed in Group 4 PDX tumors (DMB006 and Med2312FH-derived tumors following KDM2B deletion, which may better recapitulate the Group 4 context.

Response:

1. We appreciate the reviewer's suggestion to compare changes in histone marks between the Group 3 and Group 4 models following KDM2B depletion. As mentioned above, Group 4 MB PDX models create technical challenges due to poor survival *in vitro*. Med2312 cells die within 3-5 days during *in vitro* culture. DMB006 cells do not proliferate and only survive 7-10 days. We examined H3K4me1, H3K4me3, and H3K27me3 by CUT&RUN, comparing DMB006 cells that were freshly isolated from tumors and frozen in DMSO versus cells collected after 7 days of *in vitro* culture. While uncultured DMB006 yielded strong signals with very low background noise for these marks, the CUT&RUN performed in cultured DMB006 cells produced poor signals (Rebuttal Letter Fig. 10a,b). Due to these technical challenges, we were unable to perform CUT&RUN and directly evaluate the chromatin state in Group 4 PDXs after *in vitro* culture, which is necessary for KDM2B perturbation.

2. Although assessing chromatin state with KDM2B depletion is technically challenging in Group 4 PDX, we were able to assess transcriptomic changes using RNA-seq in DMB006 following *KDM2B* KO. We found the Group 4 EnhBiv-targeted neurogenesis genes (n = 134) identified from the chromatin state profiling in patient samples were predominantly upregulated after *KDM2B* KO in Group 4 context (Rebuttal Letter Fig. 10c), recapitulating the findings obtained in Group 3 D283 cells following KDM2B degradation (Rebuttal Letter Fig. 10d). We determined EnhBiv state using CUT&RUN in fresh frozen Group 4 PDXs (Original Fig. 3e) and examined EnhBiv enrichment in the promoter of differentially expressed genes characterized using the aforementioned DMB006 *KDM2B* KO RNA-seq. EnhBiv was significantly enriched in the promoter of upregulated genes compared to downregulated genes, supporting a functional relationship between KDM2B and EnhBiv state in Group 4 MB (Rebuttal Letter Fig. 10e). Although indirect, these results suggest that KDM2B regulates EnhBiv-controlled gene expression in both Group 3 and Group 4 MB.

3. Using the robust KDM2B-dTAG system to overcome limitations associated with the CRISPR-mediated KO in molecular profiling experiments, we observed a more pronounced reduction in H3K27me3 and EnhBiv enrichment at upregulated gene promoters following KDM2B degradation (Rebuttal Letter Fig. 12a-c) compared with *KDM2B* KO in D283 (Original Fig. 5d,f). We describe these findings in detail in points #12 below.

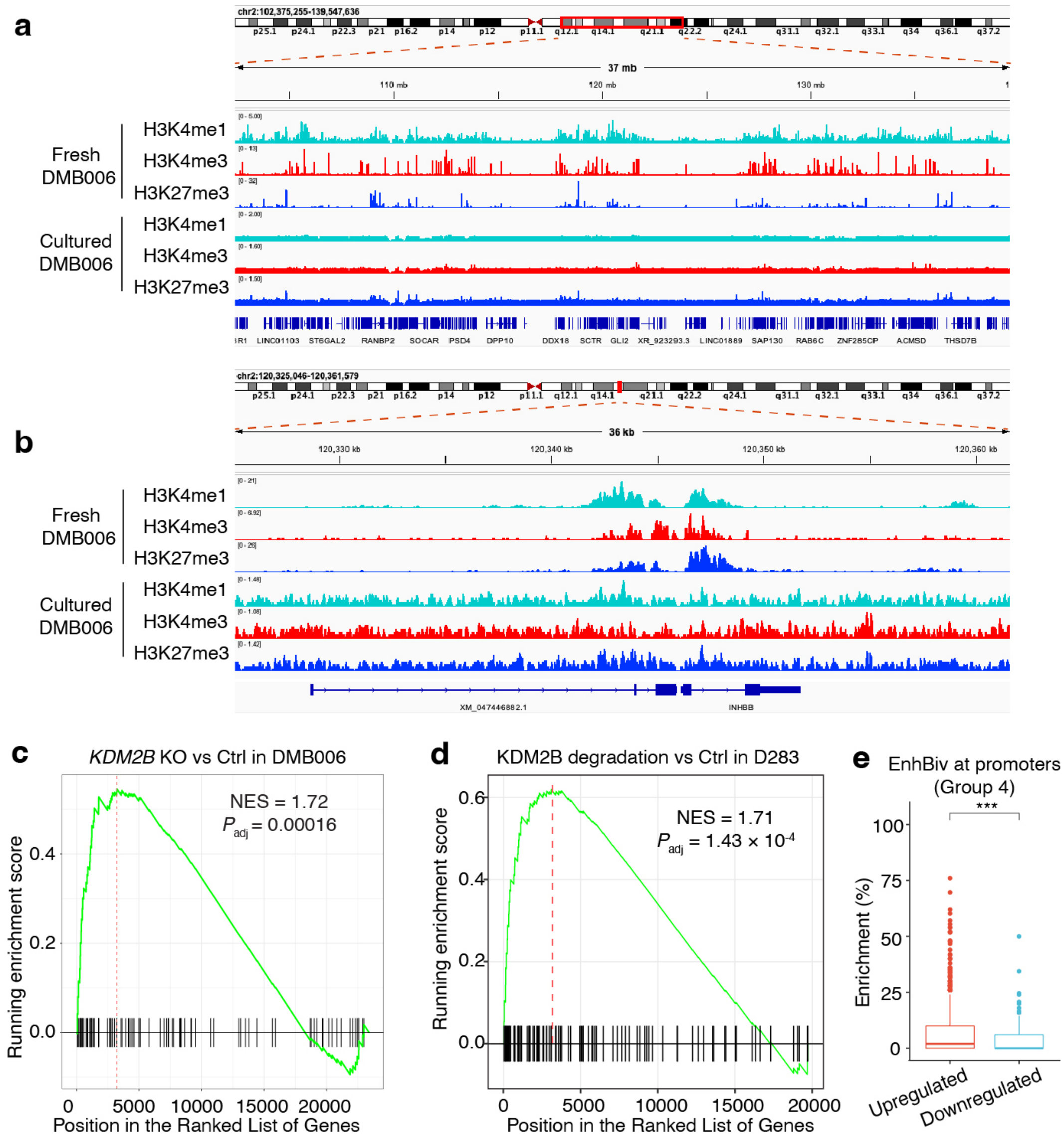

Rebuttal Letter Fig. 10 (from Fig. 5 and Extended Data Fig. 8). a and **b**, Histone marks from CUT&RUN performed using freshly isolated Group 4 DMB006 cells and DMB006 cells after 7 days *in vitro* culture for a 37 Mb region in chromosome 2 (**a**) or the *INHBB* locus in this region (**b**). **c** and **d**, Enrichment analysis showing Group 4 EnhBiv-targeted neurogenesis genes (n = 134) were significantly upregulated following *KDM2B* KO in DMB006 (**c**) or KDM2B degradation in D283 (**d**). Significance was determined by permutation test with NES > 1.5 and $P_{adj} < 0.05$ (Benjamini-Hochberg correction) considered statistically significant. **e**, Enrichment of EnhBiv state at the promoters of upregulated genes compared with downregulated genes in Group 4 PDXs. Only promoters that are targeted by EnhBiv state (upregulated, n = 253; downregulated, n = 42) are included in the analysis. Boxes represent the IQR, center line indicates the median, and whiskers extend to the most extreme data points within 1.5 × IQR. Dots indicate outliers beyond 1.5 × IQR. Statistical tests were conducted using the two-sided t-test. ***, $P < 0.001$.

11) What are the H3K26me2 peaks in D283 cells following KDM2B deletion in CUT&RUN experiments?

Response:

1. We thank the reviewer for raising this important point. Our data in the first version of the manuscript suggested KDM2B regulates gene expression through PRC2-mediated H3K27me3, and we did not initially explore contributions of KDM2B demethylase activity[14,16]. In the original manuscript, we used *KDM2B* targeting gRNAs to deplete KDM2B in Group 3 D283 cells. At seven days post-targeting, we isolated histones and performed quantitative mass spectrometry. We did not detect significant changes in global H3K36me2 abundance (Rebuttal Letter Fig. 11a), which was corroborated by immunoblotting (Rebuttal Letter Fig. 11b).

2. To determine whether KDM2B may regulate H3K36me2 chromatin occupancy, we performed CUT&RUN in Group 3 D283 KDM2B-dTAG cells five days post-dTAG treatment. We did not identify changes in H3K36me2 either globally or at the promoters of upregulated genes (Rebuttal Letter Fig. 11c,d,g,h).

3. In contrast to the lack of H3K36me2 changes, we observed slightly reduced H3K27me3 globally and profoundly reduced H3K27me3 at the promoters of genes upregulated upon KDM2B degradation (Rebuttal Letter Fig. 11e,f,i,j). These findings suggest KDM2B does not regulate gene expression through H3K36me2 demethylation in this cellular context, but instead primarily promotes H3K27me3 deposition at specific loci.

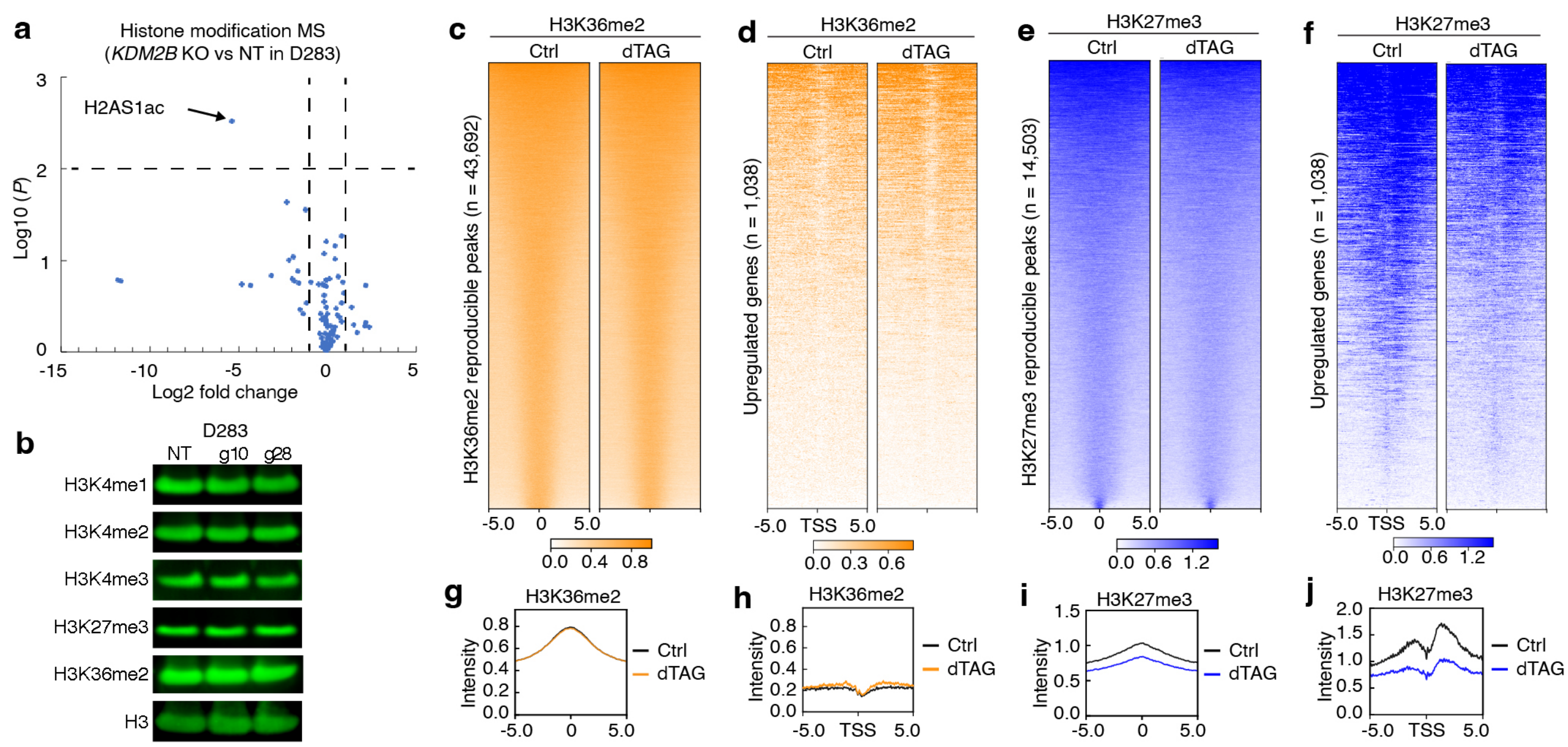

Rebuttal Letter Fig. 11 (from Fig. 5 and Extended Data Fig. 8). a, Volcano plot showing changes of total histone post-translational modifications (PTMs) determined by histone mass spectrometry analysis following *KDM2B* KO in D283. **b**, Immunoblot examining histone PTMs comparing *KDM2B* KO (g10 and g28) with non-targeting (NT) control in D283. Histone H3 was used as an internal control. **c** and **d,** CUT&RUN heatmaps showing H3K36me2 either globally (**c**) or at the promoters of upregulated genes (**d**) following KDM2B degradation in D283. **e** and **f**, CUT&RUN heatmap showing H3K27me3 either globally (**e**) or at promoters of upregulated genes (**f**) following KDM2B degradation in D283. **g-j** Profile plots showing average peak intensity of marks in **c-f**. In the global heatmap, 0 represents peak center.

12) It is unclear that subgroup specific KDM2B peaks were enriched for H3K4me1 (Fig 3e), but KDM2B KO significantly influenced H3K27me3 rather than H3K4me1 in Fig 5.

Response:

1. We acknowledge the need for rigorous assessment of how KDM2B regulates histone modifications. In our original CUT&RUN experiments in D283, CRISPR-mediated *KDM2B* KO resulted in reduction of H3K27me3 at promoters of upregulated genes, with little to no effect on H3K4me1, suggesting that KDM2B primarily regulates gene expression through H3K27me3 rather than H3K4me1 (Original Fig. 5d). Although CRISPR is a robust and widely used approach for genetic perturbation, there are several limitations. CRISPR-mediated KO is asynchronous and relatively slow, relying on DNA repair processes that generate heterogeneous editing outcomes and variable protein depletion, which can attenuate the magnitude and consistency of chromatin changes.

2. To overcome these limitations and rigorously validate the impact of KDM2B on H3K27me3 and H3K4me1, we employed the powerful dTAG system, which enables rapid, inducible, and near-synchronous KDM2B degradation, producing a more homogeneous perturbation. We performed CUT&RUN for KDM2B, H3K27me3, H3K4me1, and H3K4me3 in Group 3 D283 KDM2B-dTAG cells five days post-dTAG treatment. KDM2B degradation resulted in a stronger reduction in H3K27me3 deposition and EnhBiv enrichment at promoters of upregulated genes compared with CRISPR-mediated KO (Rebuttal Letter Fig. 12a-c versus Original Fig. 5d,f). In contrast, H3K4me1 levels remained largely unchanged at these promoters (Rebuttal Letter Fig. 12a,b), recapitulating the patterns observed in the D283 *KDM2B* KO experiment (original Fig. 5d).

3. At the global level, we found a modest reduction in H3K27me3 signal but no detectable change in H3K4me1 signal (Rebuttal Letter Fig. 12d,e). These lines of evidence indicate that KDM2B promotes H3K27me3 deposition and EnhBiv state enrichment at KDM2B-repressed promoters rather than globally. Our findings therefore suggest that H3K4me1 is unlikely to be directly regulated by KDM2B or a primary contributor to KDM2B-dependent gene regulation in D283 cells. We replaced the original *KDM2B* KO RNA-seq and CUT&RUN with the dTAG-based analyses in Fig. 5 and 6, which confirm and strengthen our conclusions using an orthogonal and higher-resolution perturbation strategy. These findings are consistent with a KDM2B-mediated regulatory mechanism acting through coordinated PRC1.1 and PRC2 activity in MB (see Question 13), providing a mechanistic explanation for the selective effects on H3K27me3.

4. Based on these findings, we propose that the enrichment of H3K4me1 at KDM2B-bound regions (shown in original Fig. 3e) may reflect shared chromatin context or genomic features that promote both H3K4me1 deposition and KDM2B recruitment, rather than direct regulation of H3K4me1 by KDM2B.

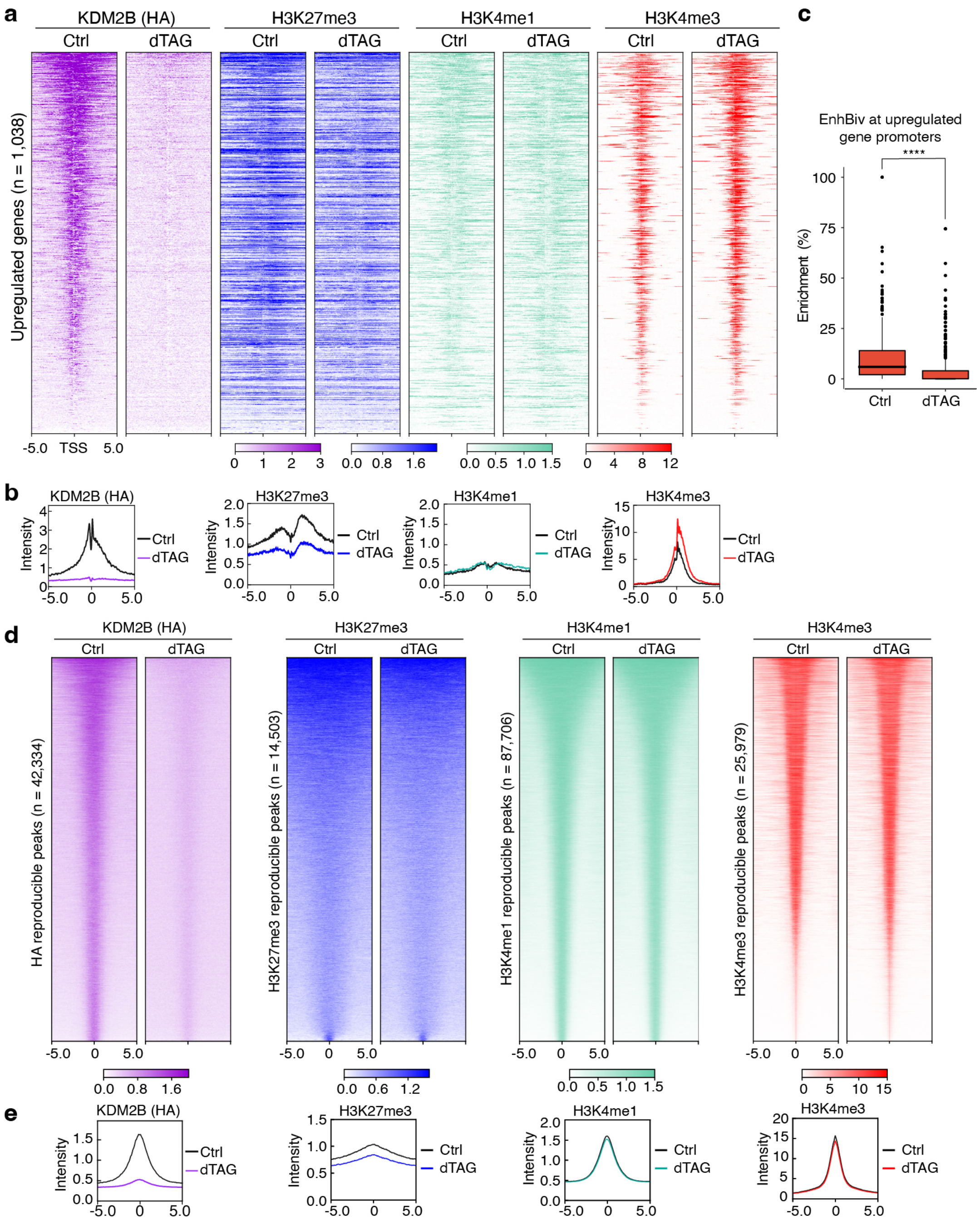

Rebuttal Letter Fig. 12 (from Fig. 5 and Extended Data Fig. 8). a, CUT&RUN heatmap of KDM2B (HA-Tag), H3K27me3, H3K4me1, and H3K4me3 signal at the promoters of upregulated genes following KDM2B degradation in D283. **b**, Profile plots showing average peak intensity of marks in **a**. **c**, EnhBiv state enrichment at the promoter of upregulated genes following KDM2B degradation in D283. Only promoters that are targeted by EnhBiv state (n = 320) are included in the analysis. Boxes represent the IQR, center line indicates the median, and whiskers extend to the most extreme data points within 1.5 × IQR. Dots indicate outliers beyond 1.5 × IQR. Statistical significance was determined by two-sided t-test. ****, $P < 0.0001$. **d**, CUT&RUN heatmaps showing global changes of KDM2B, H3K27me3, H3K4me1, and H3K4me3 in ± 5kb regions around peak center (0) following KDM2B degradation in D283. **e**, Profile plots showing average peak intensity of marks in **d**.

13) KDM2B has been extensively linked to the polycomb repressive complexes, including PRC1 and PRC2 in gene regulation. Why PRC2 rather than PRC1 was involved in the KDM2B-controlled chromatin state in Group 4?

Response:

1. We appreciate the reviewer's suggestion to elaborate on PRC1 contributions to KDM2B-dependent histone modifications and regulation of gene expression. We initially focused on PRC2 due to reduced H3K27me3 (the mark written by PRC2) and increased PRC2 target gene expression upon *KDM2B* KO. KDM2B is a component of the PRC1.1 complex[12,17], which recruits PRC2 through PRC1.1-mediated H2AK119ub[18]. To determine whether PRC1.1 might mediate EZH2 recruitment in MB cellular context, we first conducted co-IP experiments and confirmed a strong association between KDM2B and PRC1.1 components BCOR, PCGF1, and RYBP in Group 3 and 4 MB models (Rebuttal Letter Fig. 13), as expected for members of the same complex[12,17]. Surprisingly, PRC2 components, including EZH2, SUZ12, and JARID2, also physically associated with KDM2B, although interactions were weaker than observed between KDM2B and PRC1.1 components (Rebuttal Letter Fig. 13).

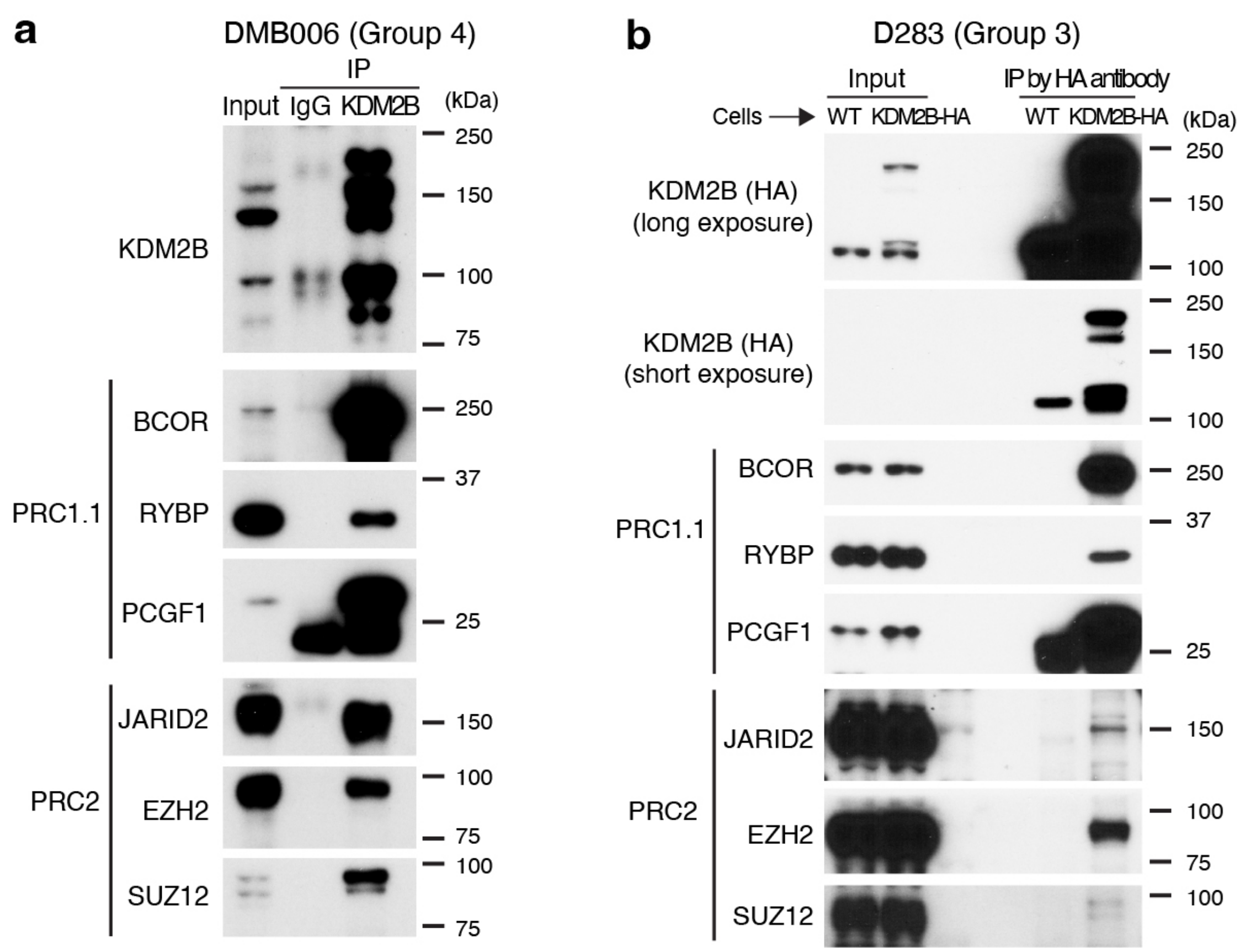

Rebuttal Letter Fig. 13 (from Fig. 6 and Extended Data Fig. 9). Immunoprecipitation demonstrating the interaction between KDM2B and PRC1 components (BCOR, RYBP, and PCGF1) or PRC2 components (JARID2, EZH2 and SUZ12) in Group 4 DMB006 (**a**) or Group 3 D283 cells (**b**). In **a**, KDM2B was pulled down from DMB006 nuclear extract using KDM2B antibody with IgG antibody as a control. In **b**, KDM2B was pulled down from wild-type D283 (control) or D283 KDM2B-HA-dTAG nuclear extracts by HA-Tag antibody.

2. Next, we performed CUT&RUN for KDM2B (by HA-Tag antibody), BCOR (PRC1.1-specific subunit), H2AK119ub (the mark written by PRC1), and EZH2 (enzymatic component of PRC2) in D283 following KDM2B acute and prolonged degradation. We found KDM2B loss as early as 12 h following dTAG treatment, both at the promoter of upregulated genes and globally (Rebuttal Letter Fig. 14a,b,e). BCOR was severely depleted at upregulated gene promoters, suggesting KDM2B-dependent PRC1.1 recruitment. The global decrease of BCOR was modest (Rebuttal Letter Fig. 14a,c,f), indicating either KDM2B-independent PRC1.1 formation[19] or compensation by KDM2B isoforms not targeted by dTAG. H2AK119ub was dramatically reduced at the promoter of upregulated genes as early as 12 h with no change observed globally (Rebuttal Letter Fig. 14a,d,g).

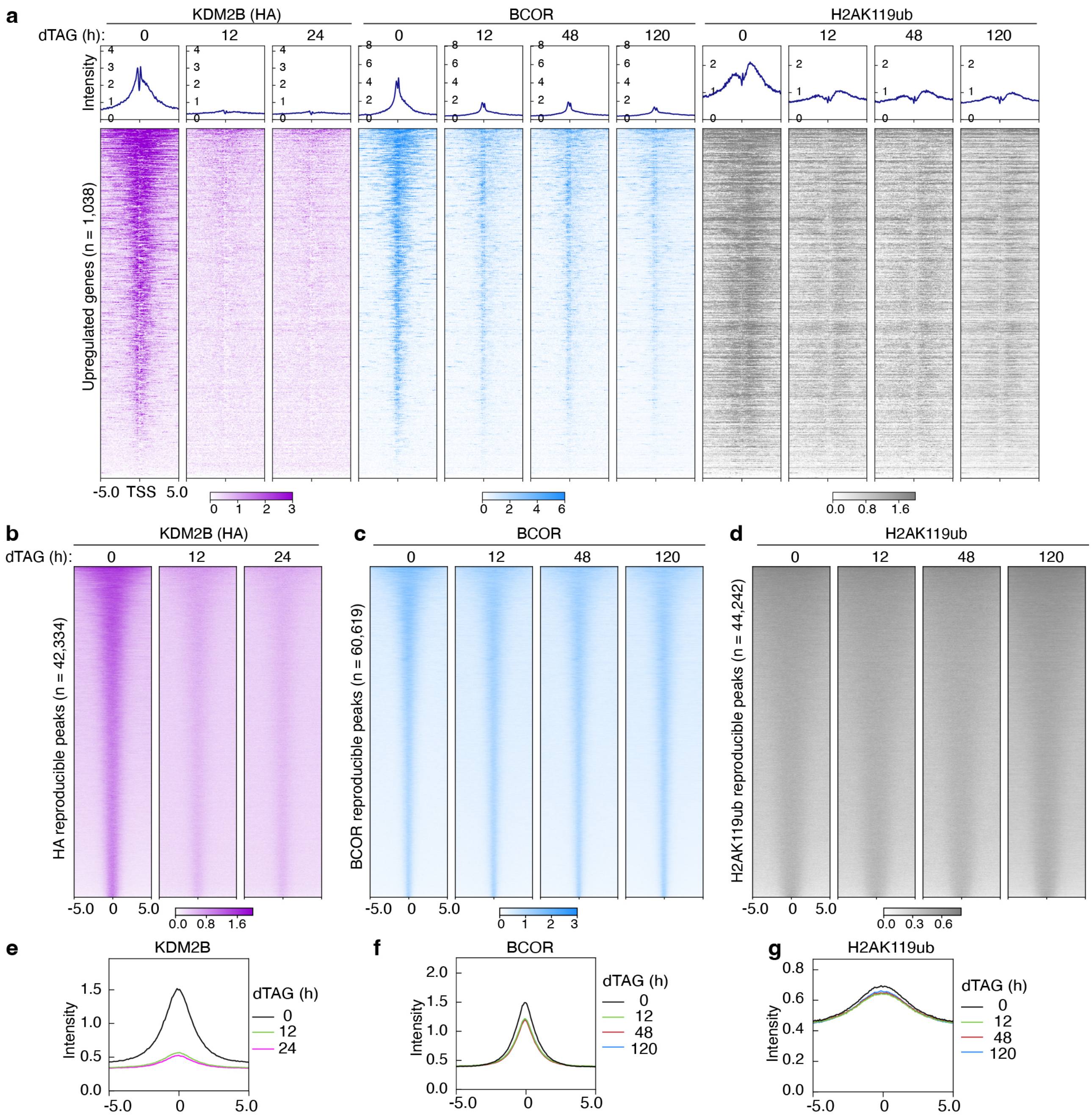

Rebuttal Letter Fig. 14 (from Fig. 6 and Extended Data Fig. 9). a, CUT&RUN analysis showing changes of KDM2B (HA-Tag), BCOR and H2AK119ub at the promoters of upregulated genes following KDM2B degradation in D283. Curves in the profile plots represent average peak intensity. **b-d**, CUT&RUN heatmaps showing global changes of KDM2B (**b**), BCOR (**c**) and H2AK119ub (**d**) at ± 5kb regions around peak center (0) following KDM2B degradation in D283. **e-g**, Profile plots quantifying average peak intensity of marks in **b-d**.

3. At 12 h post-dTAG treatment, EZH2 recruitment was partially reduced at upregulated gene promoters and further depleted following chronic KDM2B depletion (Rebuttal Letter Fig. 15a,b). The reduction of global EZH2 recruitment was minor (Rebuttal Letter Fig. 15c,d). Together, these data suggest that KDM2B represses gene expression by recruiting PRC1.1 to specific gene promoters, promoting H2AK119ub, and subsequently recruiting PRC2/EZH2. These new data have been integrated into the manuscript, and we are grateful to the reviewer for proposing this important line of investigation.

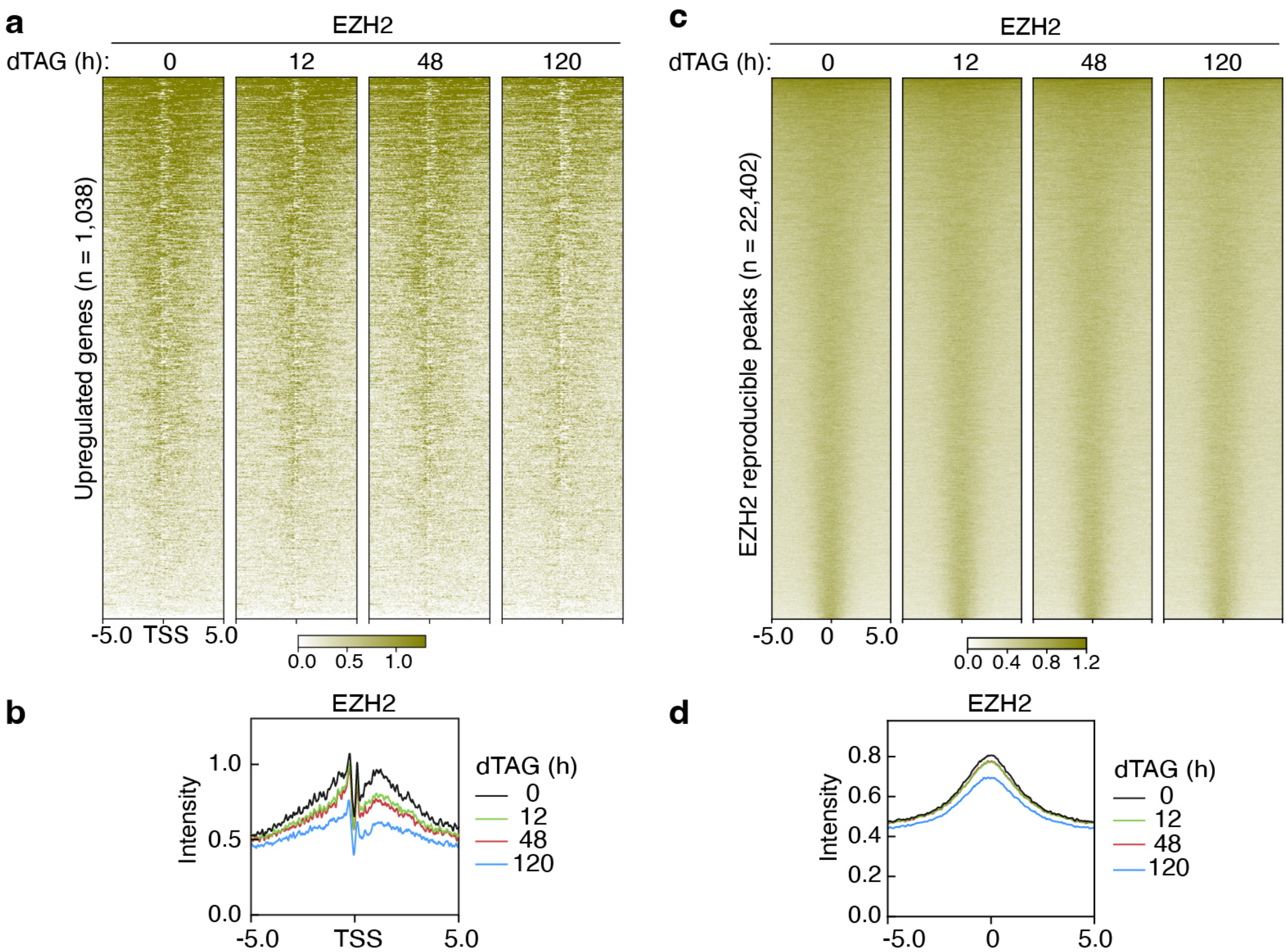

Rebuttal letter Fig. 15 (from Fig. 6 and Extended Data Fig. 9). **a**, CUT&RUN analysis showing changes of EZH2 at the promoters of upregulated genes following KDM2B degradation in D283. **b**, Profile plot showing average peak intensity of EZH2 in **a**. **c**, CUT&RUN heatmaps showing global changes of EZH2 at ± 5kb regions around peak center (0) following KDM2B degradation in D283. **d**, Profile plots showing average peak intensity of EZH2 in **c**.

4. To determine whether PRC1.1 and PRC2 complexes are functionally important for Group 3 and Group 4 MB, we examined expression and dependency of their subunits. We found that multiple PRC1.1 (Rebuttal Letter Fig. 16a) and PRC2 components (Rebuttal Letter Fig. 16b) were highly expressed in Group 3 and Group 4 relative to WNT and SHH MB. We next checked the dependency of several histone-modifying complexes by mining the Cancer Dependency Map (DepMap), which systematically identifies genetic dependencies through genome-scale CRISPR loss-of-function screens across a broad panel of pediatric and adult cancer cell lines[20]. We examined the dependency of complexes that regulate H3K4 and H3K27 methylation either directly or indirectly, including all six PRC1 subcomplexes, two PRC2 subcomplexes, three COMPASS complexes, and the CoREST complex, comparing eight Group 3 MB cell lines (D341, D425, D458, D283, MB002, HDMB03, MED411FH and SJMB016880TO) to 1,182 other cancer cell lines. Only the KDM2B-containing PRC1.1 complex and PRC2.2 complex, which is recruited by PRC1.1-mediated H2AK119ub[18], represented Group 3 selective dependencies (Rebuttal Letter Fig. 16c).

5. In addition, several subunits of PRC1.1 (Rebuttal Letter Fig. 16d) and PRC2 complexes (Rebuttal Letter Fig. 16e) had significantly lower Chronos dependency scores (gene effect) in Group 3 MB relative to all other cancer cell lines, indicating they were selectively required for growth of Group 3 MB. As Group 4 MB is not amenable to *in vitro* culture and is thus not available in DepMap, we were unable to evaluate the dependency of these subunits in Group 4.

In summary, PRC1.1 and PRC2 subunits interact with KDM2B, localize to promoters of KDM2B-repressed genes, exhibit elevated expression in Group 3 and Group 4 MB, and represent selective dependencies in Group 3 MB. These data are consistent with the high *KDM2B* expression and selective dependency in Group 3 and Group 4 models in the current study, supporting a paradigm where KDM2B-coordinated PRC1.1 and PRC2 activity is essential for Group 3/4 MB maintenance. Based on the reviewer's insightful suggestion, we have clarified PRC1.1 as the intermediate step for recruiting PRC2 in the Results and Discussion sections.

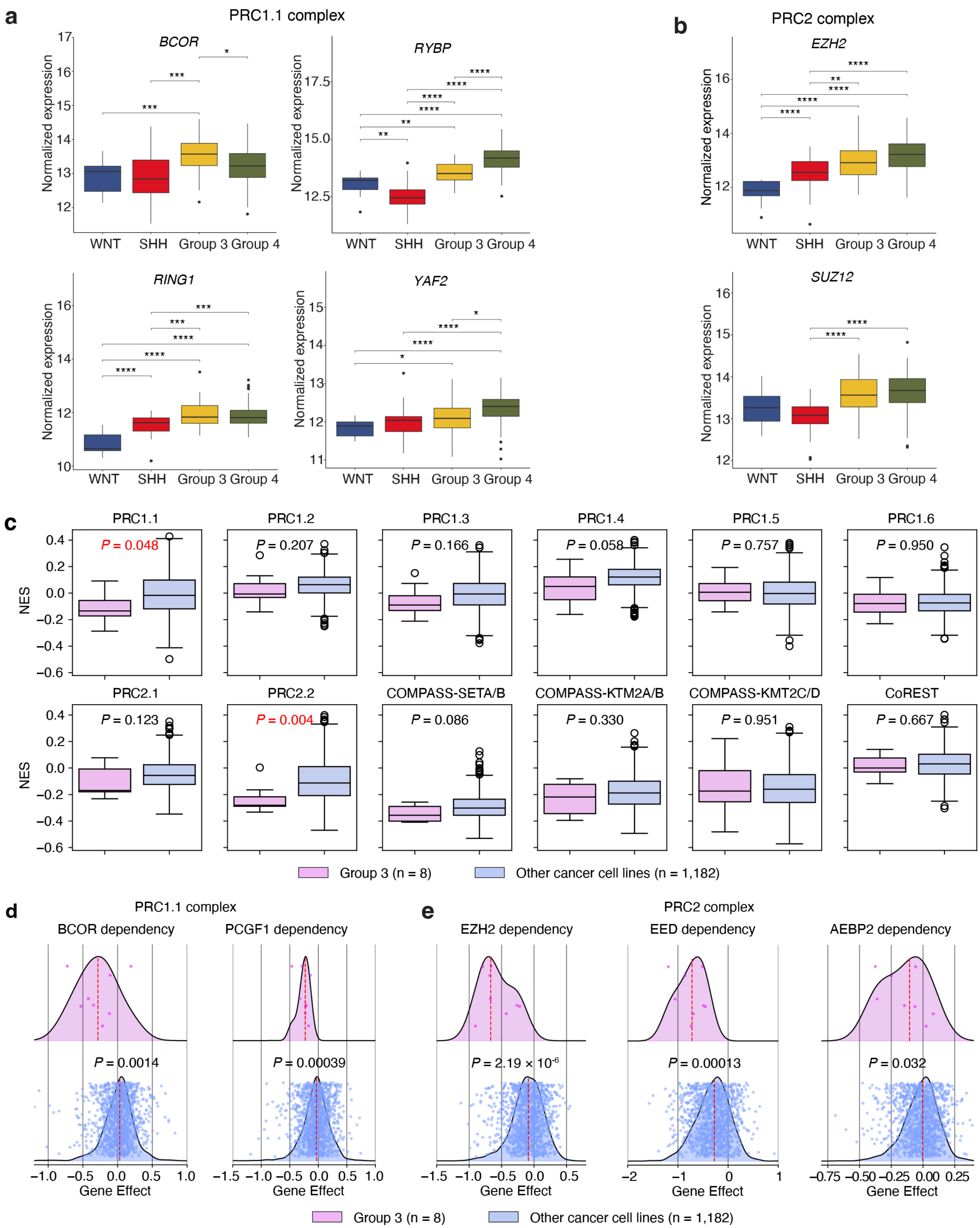
a
PRC1.1 complex
BCOR
RYBP
RING1
YAF2
Normalized expression
WNT
SHH
Group 3
Group 4
b
PRC2 complex
EZH2
SUZ12
c
PRC1.1
PRC1.2
PRC1.3
PRC1.4
PRC1.5
PRC1.6
P = 0.048
P = 0.207
P = 0.166
P = 0.058
P = 0.757
P = 0.950
PRC2.1
PRC2.2
COMPASS-SETA/B
COMPASS-KTM2A/B
COMPASS-KMT2C/D
CoREST
P = 0.123
P = 0.004
P = 0.086
P = 0.330
P = 0.951
P = 0.667
NES
Group 3 (n = 8)
Other cancer cell lines (n = 1,182)
d
PRC1.1 complex
BCOR dependency
PCGF1 dependency
P = 0.0014
P = 0.00039
e
PRC2 complex
EZH2 dependency
EED dependency
AEBP2 dependency
P = 2.19 × 10-6
P = 0.00013
P = 0.032
Gene Effect
Group 3 (n = 8)
Other cancer cell lines (n = 1,182)

Rebuttal Letter Fig. 16 (from Extended Data Fig. 10). a and **b**, Boxplots showing expression of PRC1.1 (**a**) and PRC2 (**b**) subunits across MB subgroups (WNT, n = 15; SHH, n = 46; Group 3, n = 41; Group 4, n = 65). Boxes represent the IQR, center line indicates the median, and whiskers extend to the most extreme data points within 1.5 × IQR. Dots indicate outliers beyond 1.5 × IQR. Statistical significance was assessed using the two-sided t-test, with *P* adjusted by the Holm method. *, $P_{adj} < 0.05$; **, $P_{adj} < 0.01$; ***, $P_{adj} < 0.001$; ****, $P_{adj} < 0.0001$. **c**, Boxplots showing growth dependency of chromatin modifying complexes comparing Group 3-MB (n = 8) with all other cancer cell lines (n = 1,182) in DepMap. Normalized enrichment scores (NES) were computed from DepMap dependency scores for each line, with lower scores indicating stronger dependencies. Boxes represent the IQR, center line indicates the median, and whiskers extend to the most extreme data points within 1.5 × IQR. Dots indicate outliers beyond 1.5 × IQR. Statistical significance was determined by two-tailed Mann-Whitney U test, with $P < 0.05$ considered statistically significant. **d** and **e**, Gene effect of PRC1.1 (**d**) and PRC2 (**e**) subunits in Group-3 MB (n = 8) and all other cancer cell lines (n = 1,182). A lower gene effect score means that a gene is more likely to be a dependency. The red dashed line indicates median values of gene effect. $P < 0.05$ indicates Group 3 selective dependency.

14) Since KDM2B is a key epigenetic factor in determining MB subgroups, genetic KDM2B knockout and/or transgenic KDM2B mouse models would be useful to illustrate lineage differentiation and induced MB formation, thereby validating tumor type specificity.

Response:

1. We agree that creating new mouse models and examining the tumorigenic role of *KDM2B* in early cerebellar development, either by overexpressing *KDM2B* or knocking it out in combination with driver mutations, would be of interest for future experiments. The generation of faithful Group 3 and Group 4 MB mouse models in the pediatric neuro-oncology community has been severely hampered by the lack of conservation between human and mouse cerebellum. Indeed, recent cross-species comparison of the human and mouse RL, the germinal zone from which Group 3 and Group 4 MB are suspected to originate, has determined that the mouse lacks equivalency of the mitotic RL^{SVZ} subpopulations underlying Group 3/4 MB[21-23]. As such, there are currently no accurate genetically engineered mouse models of Group 4 MB, and extant Group 3 models near universally rely on aberrant Myc over-expression, which can transform most cell types in the developing mouse cerebellum[24]. Given the significant challenges experienced by the community with respect to modeling Group 3/4 MB in the mouse, we believe that experimentally addressing the reviewer's suggestion would constitute an entirely separate study that is beyond the scope of the extensive mechanistic and functional studies included in the current manuscript.

15) Whether genetic deletion of EZH2 or EZH2 inhibitors can dismantle KDM2B-controlled EnhBiv state in Group 4 MB?

Response:

1. We appreciate the suggestion to test the requirement of EZH2 for KDM2B regulation of the EnhBiv state. *EZH2* KO or EZH2 inhibitor treatment requires cells to be cultured *in vitro* for several days. As explained above (Question 10 and Rebuttal Letter Fig. 10), poor survival of Group 4 PDX in culture creates significant technical challenges for obtaining quality histone

signal detection with CUT&RUN. Chromatin state profiling in Group 4 PDXs following *EZH2* KO or EZH2 inhibitor treatment is thus not currently feasible.

2. As an alternative approach, we performed CUT&RUN of H3K4me1, H3K4me3, and H3K27me3 in D283 cells treated with EZH2 inhibitor (Tazemetostat). We defined EnhBiv using the ChromHMM 7-state model and found that EZH2 inhibition significantly depleted EnhBiv state enrichment at the promoters of KDM2B-repressed genes (Rebuttal Letter Fig. 17).

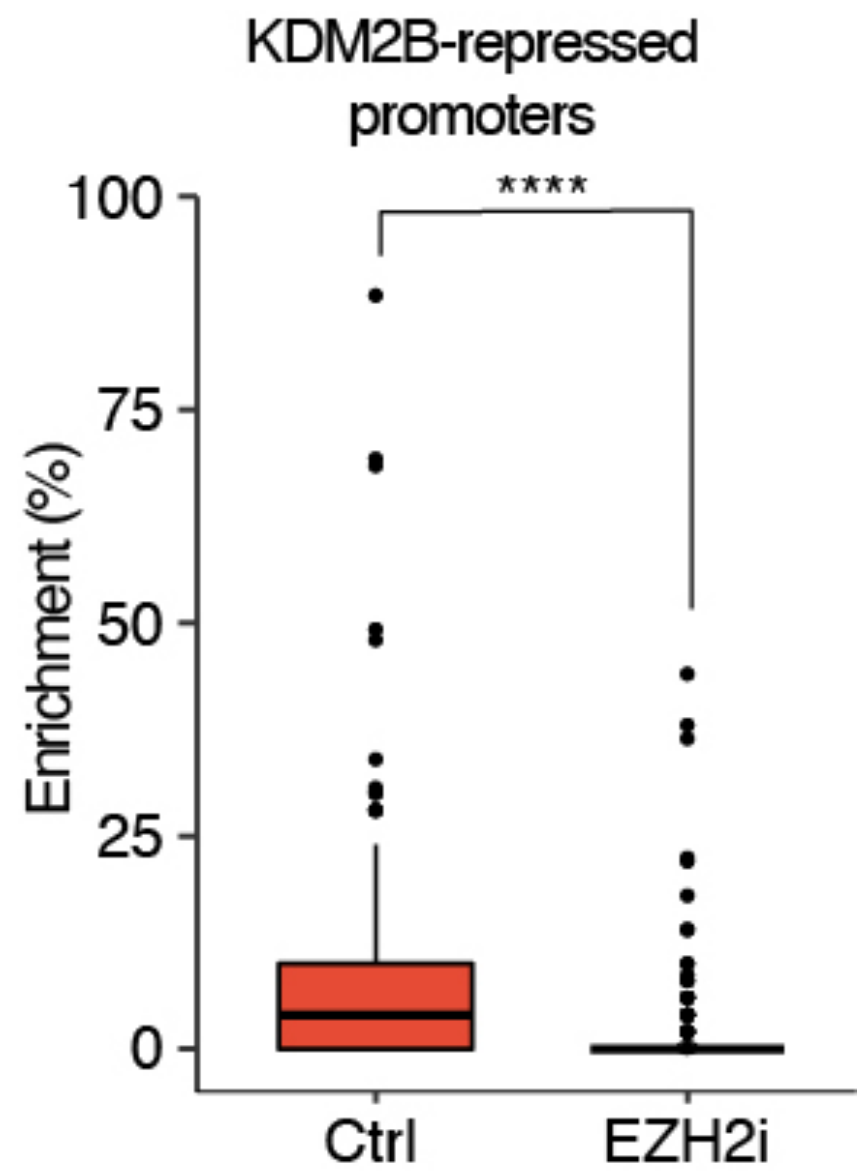

Rebuttal Letter Fig. 17. Box plot showing changes of EnhBiv state enrichment following EZH2 inhibitor (EZH2i) treatment at the promoters of genes upregulated upon KDM2B degradation (n = 141). Only promoters with detected EnhBiv state enrichment are included in the analysis. D283 cells were treated with 5 μM Tazemetostat for 120 h prior to collection. Boxes represent the IQR, center line indicates the median, and whiskers extend to the most extreme data points within 1.5 × IQR. Dots indicate outliers beyond 1.5 × IQR. Statistical significance was determined by two-sided t-test. ****, $P < 0.0001$.

In summary, we thank the reviewer for their support of this work by suggesting studies to strengthen the manuscript and improve clarity. In response, we have added extensive mechanistic studies and heavily revised the text to reflect the new insights gained from these comprehensive experiments.

Reviewer #2:

Remarks to the Author:

In this study, the authors acquired 254 histone modification maps, including H3K4me1, H3K4me3, H3K27ac, H3K36me3, H3K27me3, H3K9me3 in 52 primary fresh-frozen human medulloblastoma tissue samples and stratified the genomes by chromatin state. They identified an enhancer bivalent state (occupied by H3K27me3 and H3K4me1) specifically enriched in Group 4 medulloblastomas and provided evidence that this state is promoted by KDM2B through its recruitment of PRC2 to chromatin to repress neurodevelopmental genes. The study further validated KDM2B, which could be a novel therapeutic target, as a selective dependency in Group 3/4 medulloblastomas. This new tumor dataset is important, and the computational methods employed are effective in

simplifying the comparison of chromatin states between tumors. The novel finding of a tumor dependency mediated by bivalent chromatin through KDM2B polycomb recruitment, along with the promising selectivity of this dependency, adds significant value to the field. However, the importance of KDM2B in MB and the mechanistic link between KDM2B, PRC2, and neurodevelopmental genes needs additional evidence.

Response:
We thank Reviewer 2 for the thoughtful and constructive evaluation of our work and for recognizing the significance of the dataset, the computational framework, and the potential therapeutic implications of our findings. In response, we performed extensive additional experimental and computational analyses and substantially revised the manuscript. The new data provide rigorous validation of KDM2B as a selective dependency in Group 3 and Group 4 medulloblastoma (MB) and further delineate the mechanistic basis underlying KDM2B-associated enhancer bivalency and tumor growth. These findings support a model in which KDM2B promotes direct recruitment of the Polycomb complexes to chromatin, thereby repressing neurodevelopmental gene expression. Together, these additions substantially strengthen the mechanistic framework and clarify the functional importance of KDM2B in the maintenance of Group 3 and Group 4 MB.

Major Comments
1) Selectivity of KDM2B Dependency: The selectivity of KDM2B dependency in group 4 MB should be substantiated.
a. Could the authors use DepMap or similar databases to compare KDM2B sensitivity between group 4 and other subtypes of medulloblastoma lines. Alternatively, the authors can perform KDM2B knockdown in additional cell line to rescue or validate its importance in group 4 MB to confirm the selectivity of this dependency.

Response:
We appreciate the reviewer's thoughtful comments regarding the selectivity of *KDM2B* dependency. To address this directly, we combined interrogation of the DepMap database with expanded functional validation across multiple pediatric brain tumor models.

1. Existing pediatric dependency resources such as the Cancer Dependency Map (DepMap) are limited in their ability to assess subgroup-specific dependencies in MB. Available MB cell lines are heavily biased toward *MYC*-amplified Group 3 tumors, and faithful *in vitro* models of non-Group 3 MB remain scarce. DepMap reports no significant *KDM2B* dependency in Group 3 MB models (Rebuttal Letter Fig. 18a). This result is likely confounded by gRNA design. The genome-wide CRISPR libraries used in DepMap were not optimized to target functional protein domains (Rebuttal Letter Fig. 18b). Prior work has shown that only gRNAs targeting the CXXC DNA-binding domain of KDM2B induce a strong loss-of-fitness phenotype[25]. Accordingly, we designed domain-specific gRNAs that generate deletions spanning the CXXC domain (Rebuttal Letter Fig. 18b), which revealed a strong *KDM2B* dependency in both Group 3 and Group 4 MB (Original Fig. 4).

2. To rigorously test the selectivity of this dependency, we expanded our *in vitro* and *in vivo* competition assays from six to a total of twelve pediatric brain tumor models, including three Group 3 MB (D425, D283, and CHLA-01-MED) (Rebuttal Letter Fig. 18c-e), three SHH MB (SJNBT3, SJNBT12 and Daoy) (Rebuttal Letter Fig. 18f-h), three H3.3 G34R high-grade gliomas (HGGs) (SJ-HGGX99c, SJ-HGGX77c, and SJ-HGGX90c) (Rebuttal Letter Fig. 18i-k), one H3.3 K27M HGG (SJ-DIPGX7) (Rebuttal Letter Fig. 18l), and two Group 4 MB (DMB006 and Med2312FH) (Rebuttal Letter Fig. 18m,n). Across this expanded panel, *KDM2B* loss consistently impaired tumor cell fitness in Group 3 and Group 4 MB, while SHH MB and HGG models showed minimal or no dependency (Rebuttal Letter Fig. 18c-o). Together, these analyses demonstrate that KDM2B dependency is selective to Group 3 and Group 4 MB, rather than representing a general vulnerability of pediatric brain tumors.

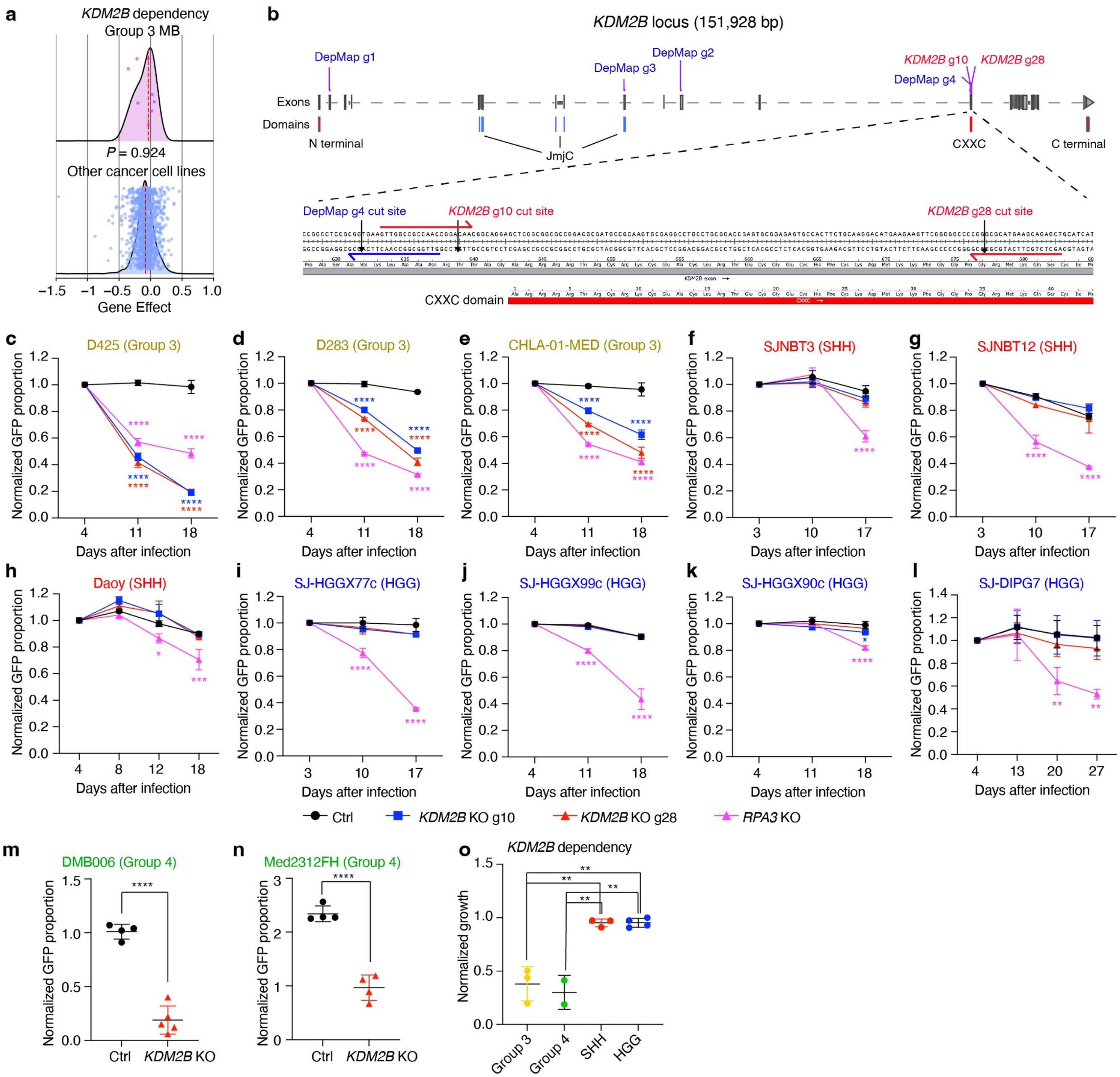

Rebuttal letter Fig. 18 (from Fig. 4 and Extended Data Fig. 6). a, Gene effect of KDM2B in Group-3 MB (n = 8) and all other cancer cell lines (n = 1,182). A lower gene effect score means that a gene is more likely to be a dependency. The red dashed line indicates median values of gene effect. $P > 0.05$ indicates that the difference between Group 3 MB and other cancer cell lines is not significant. **b**, Schematic of *KDM2B* locus depicting *KDM2B* exon and functional domains. gRNAs used in this study (g10, g28; red) or in DepMap (g1-g4; blue) are shown. **c-l**, *In vitro* competition assay confirming *KDM2B* as a dependency in Group 3-MB cell lines (**c**-**e**) but not in SHH MB (**f**-**h**) or HGG (**i**-**l**) cell lines. Values represent mean ± standard deviation (SD) determined from two biological replicates. Adjusted *P* was generated by two-way ANOVA with Dunnett's correction. *, $P_{adj} < 0.05$; **, $P_{adj} < 0.01$; ***, $P_{adj} < 0.001$; ****, $P_{adj} < 0.0001$. **m** and **n**, *In vivo* competition assay demonstrating *KDM2B* is required for growth of Group 4-MB PDXs. n = 4-5 mice per group. Center line and error bar indicates mean ± SD. *P* was determined by two-tailed t-test. ****, $P < 0.0001$. **o**, Summary of *KDM2B* dependency. Data points represent individual cell models (Group 3, n = 3; Group 4, n = 2; SHH, n = 3; HGG, n = 4). Center line and error bar indicates mean ± SD. Adjusted *P* was generated by two-way ANOVA with Tukey's correction. **, $P_{adj} < 0.01$.

b. Could the authors leverage additional cancer genomics datasets to correlate KDM2B expression with patient survival, tumor stage, and other clinical parameters.

Response:

1. We thank the reviewer for the recommendation to expand upon the clinical relevance of KDM2B in MB. To address the reviewer's suggestion, we analyzed additional publicly available multi-modal MB datasets[26] to assess associations between *KDM2B* expression and patient sex, tumor stage, age, and overall survival in Group 3 and Group 4 MB. Across these variables, *KDM2B* expression was not associated with patient survival, tumor stage, or age in either Group 3 or Group 4 MB. The only significant clinical association observed was higher *KDM2B* expression in male patients within Group 4 MB (Rebuttal Letter Fig. 19).

2. We further examined the relationship between *KDM2B* expression and molecular features using a previously published Group 3 and Group 4 MB cohort[27]. Overall, the gradient of *KDM2B* expression mirrored the biological continuum, with increasing expression along the gradient from Group 3 to Group 4 (Rebuttal Letter Fig. 20a,b). Higher *KDM2B* expression was significantly correlated with somatic alterations in chromatin-modifying genes, including *PRDM6*, *KDM6A*, and *KMT2C*, supporting a link between elevated *KDM2B* expression and epigenetic regulatory programs characteristic of these subgroups.

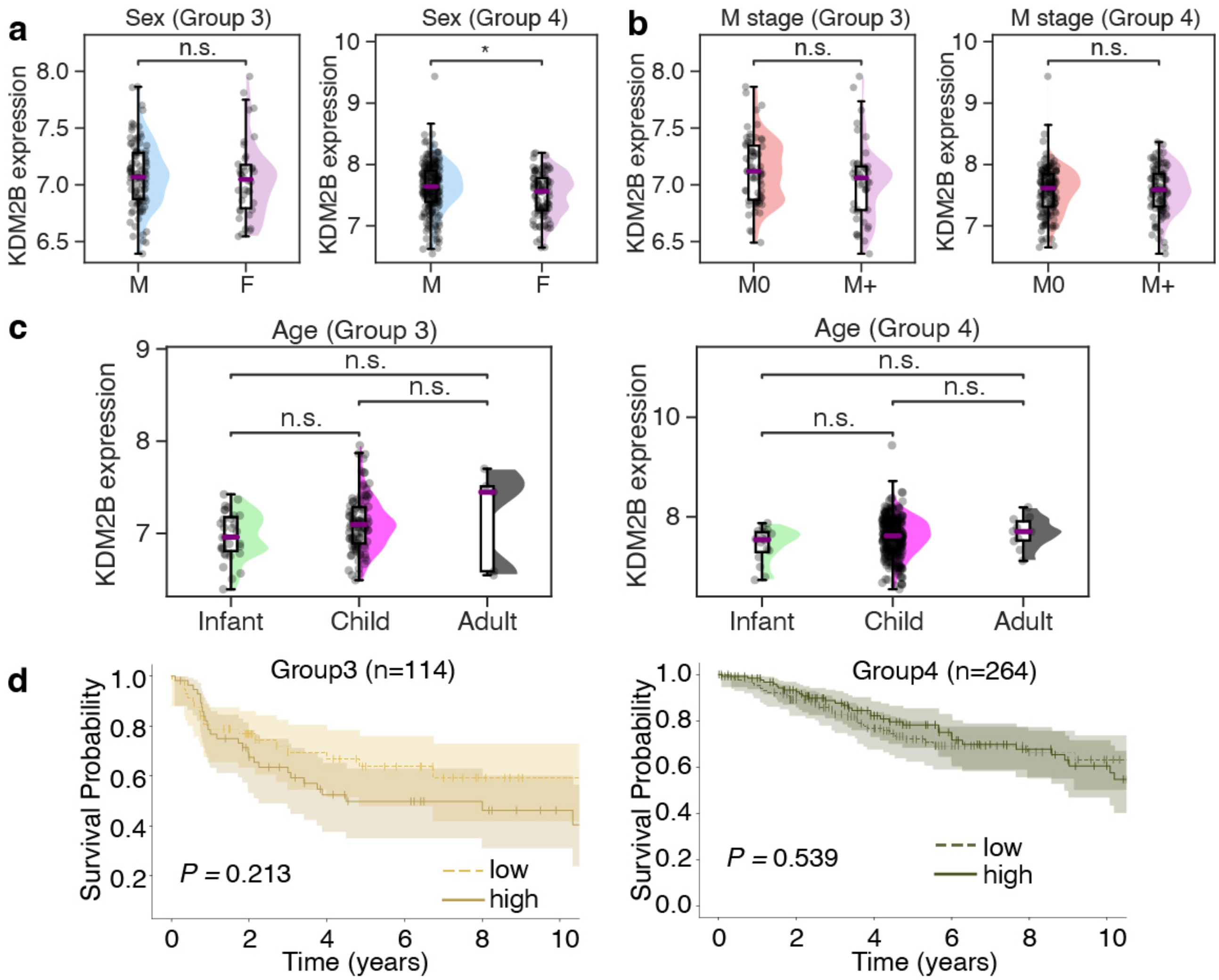

Rebuttal Letter Fig. 19. Clinical relevance of *KDM2B* expression in Group 3 and Group 4 MB. **a**, Patient sex. Group 3: male (M, n = 99); female (F, n = 38). Group 4: male (M, n = 216); female (F, n = 92). **b**, Metastasis stage. Group 3: local tumor (M0, n = 66); metastatic tumor (M+, n = 43). Group 4: local tumor (M0, n = 154); metastatic tumor (M+, n = 101). **c**, Patient age. Group 3: Infant, < 3 years (n = 31); child, 3-15 years (n = 100); adult, > 16 years (n = 5). Group 4: Infant, < 3 years (n = 16); child, 3-15 years (n = 288); adult, > 16 years (n = 10). **d**, Survival probability. High and low groups describe patients with *KDM2B* expression above or below the median value. Group 3: KDM2B high, n = 57; KDM2B low, n = 57. Group 4: KDM2B high, n = 132; KDM2B low, n = 132. $P > 0.05$ indicates statistically not significant (n.s.) as determined by Mann Whitney 2-tailed U-test or by Mantle-Cox log rank test for survival.

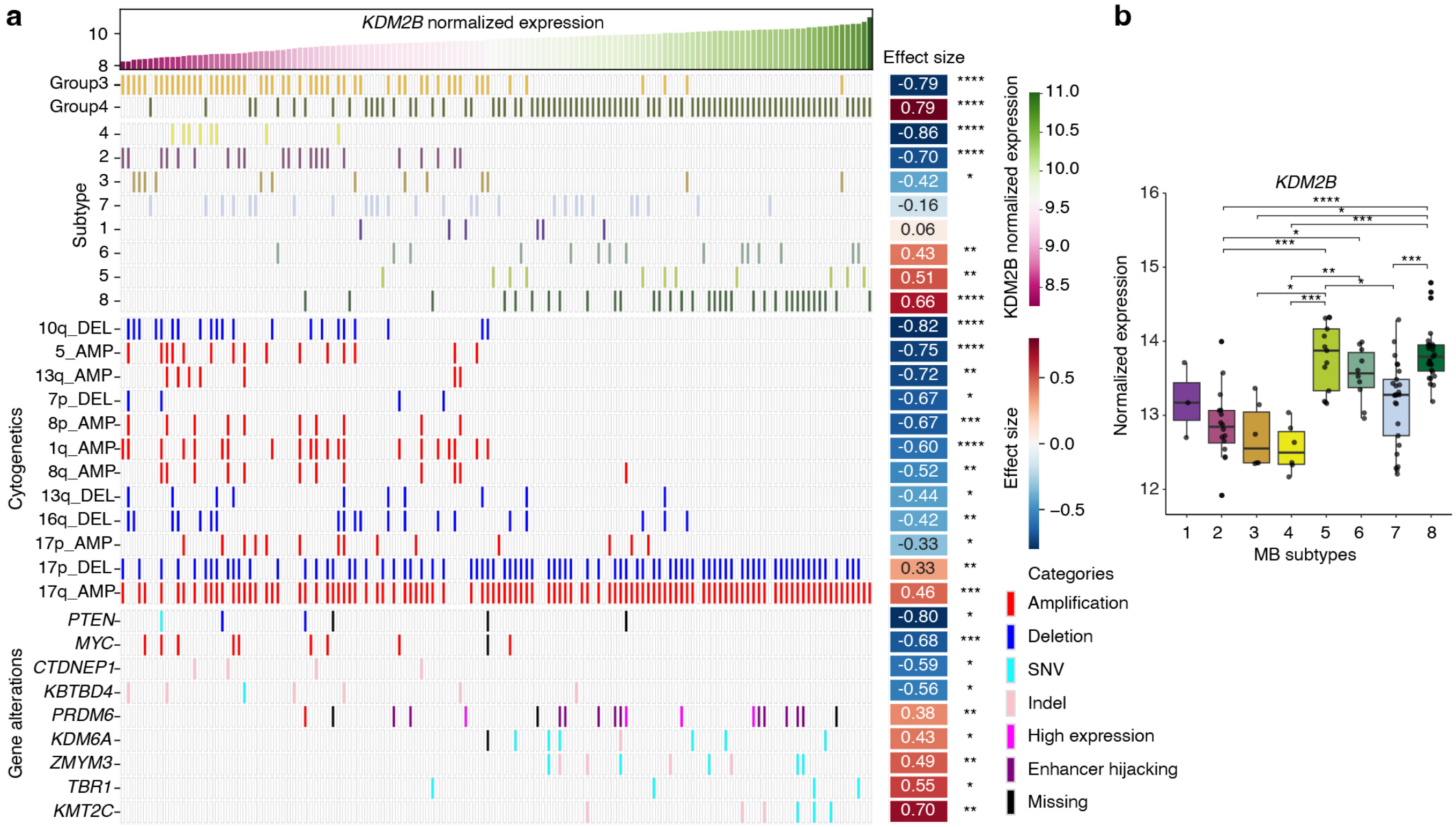

Rebuttal Letter Fig. 20 (From Extended Data Fig. 4). a, Oncoprint showing association of subgroups, subtypes, copy number alterations, and gene alterations with *KDM2B* expression in a cohort of Group 3- and Group 4-MB patients (n = 136). Samples were ordered by increasing *KDM2B* expression. Effect size > 0 indicates positive correlation between the alteration and *KDM2B* expression, while effect size < 0 indicates a negative correlation. Only alterations present in at least three samples with |effect size| > 0.2 and $P < 0.05$ are shown. Two-sided P values were determined by permutation testing (100,000 permutations). * $P < 0.01$; ** $P < 0.01$; *** $P < 0.001$; **** $P < 0.0001$. **b**, *KDM2B* expression in eight Group 3 and Group 4 subtypes (1, n = 3; 2, n = 16; 3, n = 6; 4, n = 6; 5, n = 13; 6, n = 10; 7, n = 25; 8, n = 27). Boxes represent the interquartile range (IQR; 25th–75th percentiles), center line indicates the median, and whiskers extend to the most extreme data points within 1.5 × IQR. Statistical significance was assessed using two-sided t-test, with P adjusted by the Holm method. * $P_{adj} < 0.05$; ** $P_{adj} < 0.01$; *** $P_{adj} < 0.001$; **** $P_{adj} < 0.0001$.

2) Enhancer Bivalent vs. Promoter Bivalent Distinction:
a. The distinction between enhancer bivalent and promoter bivalent states is unclear. Figure 2C shows the enhancer bivalent state mostly enriched at promoters, and Figure 3F focuses on KDM2B-bound promoters, not enhancers.

Response:

1. We thank the reviewer for this question. In the Roadmap 18-state model, the bivalent enhancer state (EnhBiv) is defined by co-enrichment of the enhancer mark H3K4me1 and the repressive mark H3K27me3 but lacks the H3K4me3 mark associated with the transcriptional start site (TSS). In contrast, the bivalent TSS state (TssBiv, akin to bivalent promoter) is defined by co-

enrichment of H3K4me3, H3K27me3, and H3K4me1[1,2]. Using this framework, we found that EnhBiv was significantly enriched in Group 3 and Group 4 MB relative to WNT and SHH tumors, whereas TssBiv showed comparable genome-wide coverage across all four subgroups (Rebuttal Letter Fig. 21a,b).

2. To address the reviewer's query regarding the apparent promoter localization of EnhBiv, we performed additional genomic enrichment and metagene analyses in the revised manuscript. When promoters were defined as TSS ± 5 kb, both EnhBiv and TssBiv overlapped promoter-proximal regions (Rebuttal Letter Fig. 21c,d). However, examination of average signal distributions revealed that TssBiv was sharply enriched at the immediate TSS (±2 kb), whereas EnhBiv was preferentially enriched in regions flanking the TSS (Rebuttal Letter Fig. 21e). Importantly, the abundance of bivalent promoter states in the TSS ± 5 kb region did not differ across subgroups, while EnhBiv showed increased coverage in Group 3 and Group 4 tumors (Rebuttal Letter Fig. 21f). These findings suggest that, although EnhBiv overlapped promoter-proximal regions, its spatial distribution and histone mark composition distinguished it from classical poised promoters. To capture this promoter-flanking EnhBiv state, we defined promoters as TSS ± 5 kb in this study, encompassing both canonical promoters and nearby regulatory elements.

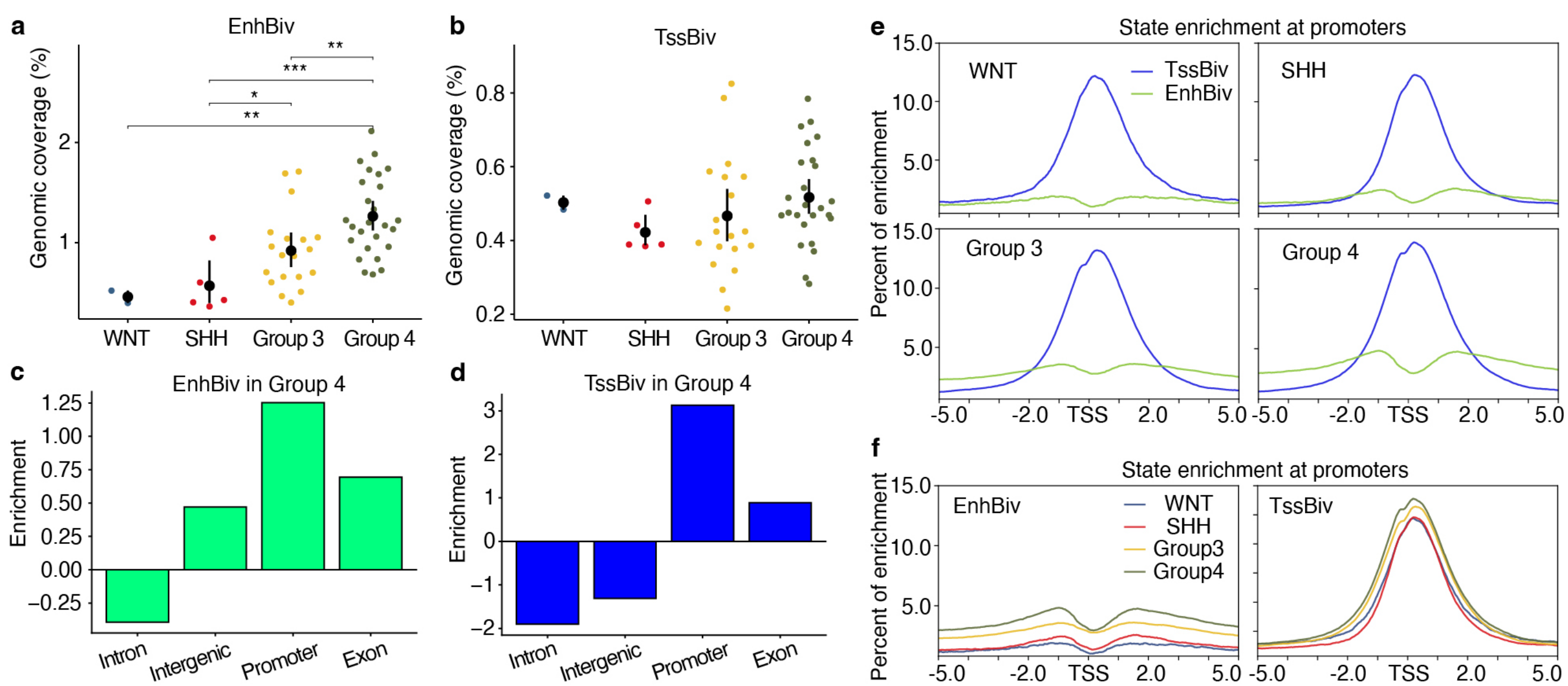

Rebuttal Letter Fig. 21 (from Fig. 2 and Extended Data Fig. 3). a and **b**, Genomic coverage of the EnhBiv (**a**) and TssBiv (**b**) states among MB subgroups. Black dots indicate median and bars indicate 1.5 × IQR. WNT, n = 2; SHH, n = 5; Group 3, n = 19; Group 4, n = 26. *, $P_{adj} < 0.05$; **, $P_{adj} < 0.01$; ***, $P_{adj} < 0.001$ determined by Mann-Whitney U-test. **c** and **d**, Genomic enrichment of the EnhBiv (**c**) and TssBiv (**d**) states in Group 4-MB. n = 50,945 regions for EnhBiv and n = 51,710 for TssBiv. **e**, Distribution of EnhBiv and TssBiv states at the TSS ± 5kb promoter window of protein coding genes (n = 21,284) in MB subgroups. **f**, Enrichment of EnhBiv and TssBiv states at the TSS ± 5kb promoter window of protein coding genes (n = 21,284) comparing MB subgroups.

b. In Figures 3A/S3A, cluster C6 highlights the major difference as the presence of the enhancer bivalent state, while all four groups exhibit the promoter bivalent state. However, KDM2B is not enriched in Group 4 for this cluster; instead, KDM2B enrichment is observed in clusters where Group 4 also selectively has the promoter bivalent state. Please provide views of additional representative loci and promoter regions with ChIP-seq tracks for H3K4me1, H3K4me3, and H3K27me3 at enhancer bivalent loci.

Response:

1. We thank the reviewer for this insightful comment and agree that the relationship between enhancer bivalency, promoter bivalency, and KDM2B enrichment requires clearer explanation. To address this, we re-analyzed cluster features, quantified the histone marks contributing to the EnhBiv state, and added representative locus-level ChIP-seq visualizations.

2. The clustering analysis was performed on 666 Group 4 EnhBiv signature genes, defined by significantly enriched EnhBiv state at promoter regions in Group 4 relative to other MB subgroups. Clustering of 10 kb promoter-centered regions confirmed that EnhBiv enrichment is higher in Group 4 across all six clusters (C1–C6), although the underlying histone features vary by cluster (Rebuttal Letter Fig. 22a,b). Quantification of histone marks revealed that H3K4me3 levels were comparable across subgroups, whereas H3K27me3 was selectively elevated in Group 4 in clusters C2–C5 and H3K4me1 was enriched in Group 4 in clusters C4–C6 (Rebuttal Letter Fig. 22a,b). Analysis of publicly available ChIP-seq datasets (ChEA within Enrichr) showed that KDM2B is significantly enriched in clusters C1–C3 and correlates more closely with elevated H3K27me3 rather than H3K4me1-driven EnhBiv states, explaining the limited KDM2B enrichment in clusters such as C6 (Rebuttal Letter Fig. 22b).

3. Representative ChIP-seq tracks at neurogenesis-related loci support these observations: promoters such as *IQSEC1* (C2) and *S100A1* (C3) show strong Group 4–specific H3K27me3 enrichment, whereas the *PAX2* promoter (C6) displays prominent H3K4me1 enrichment in Group 4 (Rebuttal Letter Fig. 22c–e).

Together, these data demonstrate that while EnhBiv states showed higher promoter coverage in Group 3 and Group 4 tumors, distinct histone mechanisms underlie this enrichment, with KDM2B preferentially associated with H3K27me3-driven EnhBiv states.

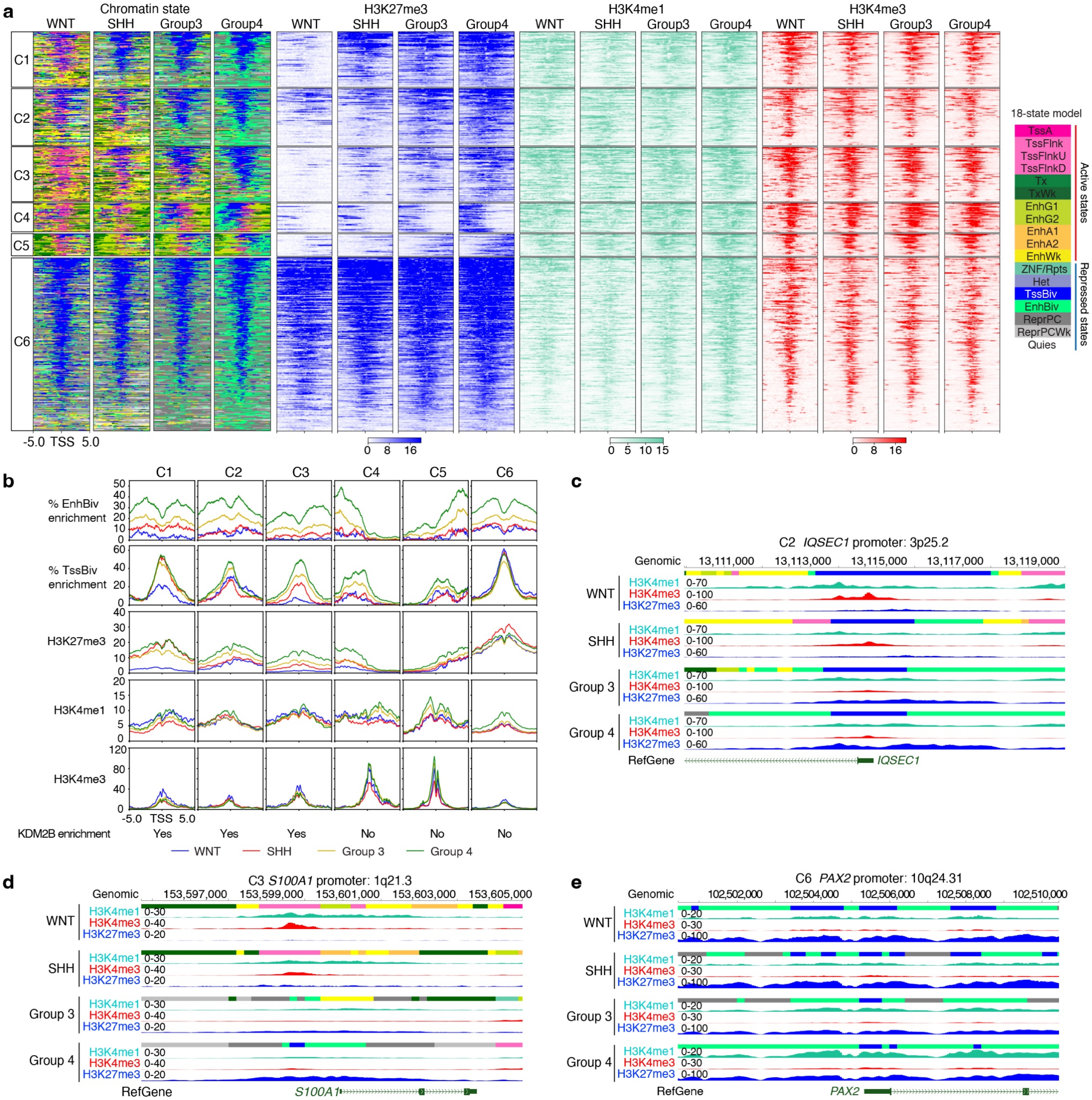

Rebuttal Letter Fig. 22 (from Fig. 2 and Extended Data Fig. 3). a, Chromatin state and histone ChIP-seq tracks in six clusters (C1-C6) of Group 4 EnhBiv signature gene promoters in MB subgroups. **b**, Profile plots showing enrichment of EnhBiv and TssBiv state or peak intensity for H3K27me3, H3K4me1, and H3K4me3 in C1-C6 across MB subgroups. The enrichment of KDM2B binding sites in C1-C3 was determined by enrichment analysis using ChEA gene set. **c-e**, Merged chromatin state and histone marks at representative promoters from C2: *IQSEC1* (**c**), C3: *S100A1* (**d**), and C6: *PAX2* (**e**) by MB subgroup.

c. Also can the authors explain their particular preference for bivenh over bivprom based on some epigenomic or genomic (CG-density?) features?

Response:

We thank the reviewer for this suggestion. In our study, we focused on EnhBiv as it was the most differentially represented state among subgroups with a strong enrichment in Group 3 and Group 4 MB; in contrast, there was no difference in TssBiv coverage across subgroups. Within the TSS ± 5kb region, TssBiv signal localized to the center of the TSS and EnhBiv was more distal and enriched in regions flanking the TSS. Consistent with features associated with enhancers versus promoters, we observed higher DNA methylation, lower GC content, and lower CpG observed/expected ratio in the EnhBiv state compared to the TssBiv state marked regions within TSS ± 5kb in Group 4 (Rebuttal Letter Fig. 23).

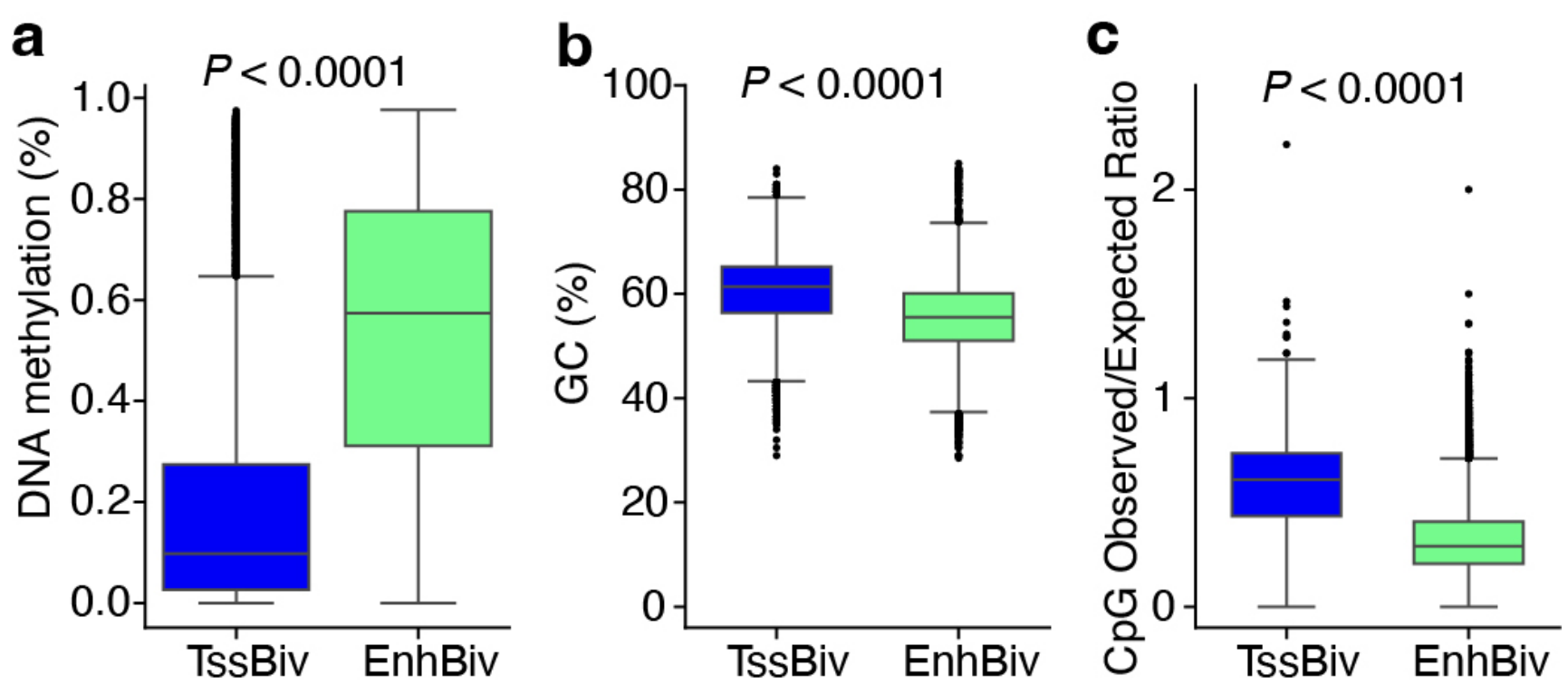

Rebuttal Letter Fig. 23 (from Extended Data Fig. 3). DNA methylation (**a**), GC content (**b**), or CpG observed/expected ratio (**c**) at promoter-enriched TssBiv (n = 8,616) and EnhBiv (n = 11,112) marked regions in Group 4-MB. Significance was determined by two-sided Mann-Whitney U test. Boxes represent the IQR, center line indicates the median, and whiskers extend to the most extreme data points within 1.5 × IQR. Dots indicate outliers beyond 1.5 × IQR.

3) Mechanistic Link Between KDM2B and PRC2: The mechanistic link between KDM2B and PRC2 is incomplete. KDM2B is known to recruit PRC1 instead of PRC2. Although the paper presents the co-localization of KDM2B and EZH2 in the genome, the physical interaction between KDM2B and EZH2 (Fig. 6i) and the reduction of EZH2 seems weak (Fig. 6f).

a. Can authors quantify their co-IP results and compare to PRC1 component (Fig. 6f)?

Response:

1. We thank the reviewer for the suggestion to add studies examining the mechanisms of KDM2B-dependent PRC2 recruitment. We conducted co-IP experiments using Group 4 patient-derived xenograft (PDX) nuclear extract (DMB006) to examine the interaction between KDM2B and multiple components of the PRC1.1 and PRC2 complexes. We identified strong association between KDM2B and PRC1.1 components BCOR, PCGF1, and RYBP, as expected for members of the same complex[12,17]. Consistent with prior reports describing physical interactions between subunits of PRC1 and PRC2[28], PRC2 components, including EZH2, SUZ12, and JARID2, also

physically associated with KDM2B, although interactions were weaker than observed between KDM2B and PRC1.1 components (Rebuttal Letter Fig. 24a).

2. We validated the interactions in Group 3 D283 KDM2B-HA-dTAG cells by pulling down endogenous KDM2B using an HA-Tag antibody. While KDM2B exhibited strong interactions with PRC1.1 subunits, the physical association between KDM2B and PRC2 subunits was weaker (Rebuttal Letter Fig. 24b).

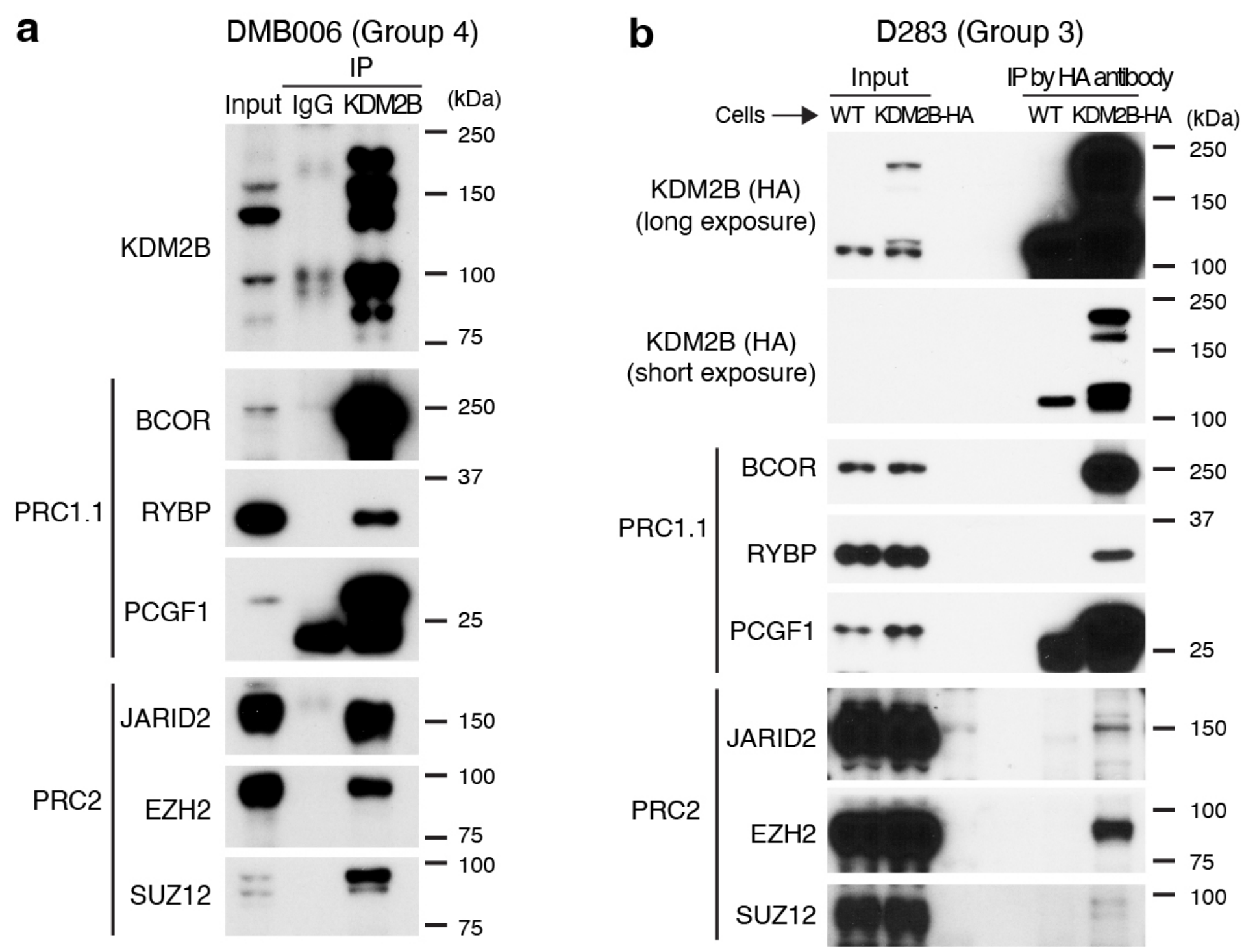

Rebuttal Letter Fig. 24 (from Fig. 6 and Extended Data Fig. 9). Immunoprecipitation demonstrating the interaction between KDM2B and PRC1 components (BCOR, RYBP, and PCGF1) or PRC2 components (JARID2, EZH2 and SUZ12) in Group 4 DMB006 (**a**) or Group 3 D283 cells (**b**). In **a**, KDM2B was pulled down from DMB006 nuclear extract using KDM2B antibody with IgG antibody as a control. In **b**, KDM2B was pulled down from wild-type D283 (control) or D283 KDM2B-HA-dTAG nuclear extracts by HA-Tag antibody.

b. The proposed recruitment could be indirect, resulting from long-term KDM2B knockout rather than direct recruitment of PRC2 by KDM2B. To address this, the authors can use their dTag cell line to show through ChIP-qPCR or ChIP-seq that PRC2 components (EZH2 or EED) quickly disappear from EnhBiv chromatin within hours of KDM2B degradation.

Response:

1. We are grateful for the fantastic recommendation to further dissect the mechanism(s) of KDM2B-dependent PRC2 recruitment. Employing the powerful dTAG system, endogenous KDM2B can be effectively depleted within a few hours, providing a unique opportunity to distinguish direct versus indirect recruitment mechanisms that cannot be addressed using conventional perturbation methods, such as CRISPR-mediated knockout (KO) or RNAi. To

assess the dynamics of PRC2 recruitment, we examined EZH2 occupancy by CUT&RUN following KDM2B acute and prolonged degradation. At promoters of genes upregulated upon KDM2B loss, KDM2B was depleted by 12 hours post-dTAG treatment, accompanied by a reduction in EZH2 recruitment as early as 12 hours, with further decreases observed upon chronic KDM2B depletion (Rebuttal Letter Fig. 25a–d). In contrast, at a global level, although KDM2B was efficiently lost by 12 hours, EZH2 occupancy was only modestly reduced at 120 hours (Rebuttal Letter Fig. 25e–h). These data indicate that KDM2B preferentially promotes EZH2 recruitment at promoters of KDM2B-repressed genes rather than globally.

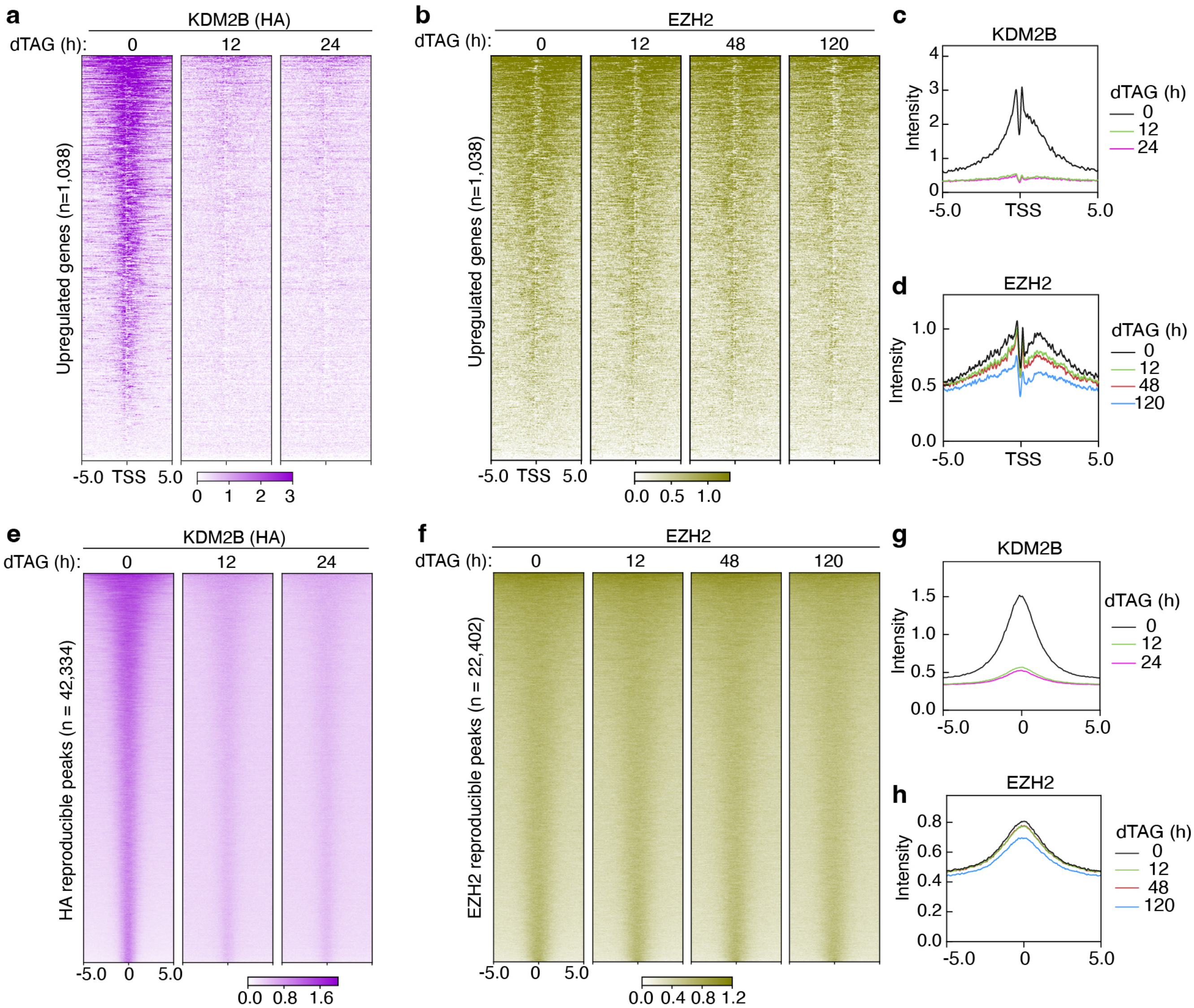

Rebuttal Letter Fig. 25 (From Fig. 6 and Extended Data Fig. 9). a and **b**, CUT&RUN heatmaps showing changes of KDM2B (**a**) and EZH2 (**b**) at the promoters of upregulated genes following KDM2B degradation in D283. **c** and **d**, Profile plot showing average peak intensity of KDM2B (**c**) and EZH2 (**d**) in **a** and **b**. **e** and **f**, CUT&RUN heatmaps showing global changes of KDM2B (**e**) and EZH2 (**f**) in ± 5kb regions around peak center (0) following KDM2B degradation in D283. **g** and **h**, Profile plots showing average peak intensity of KDM2B (**g**) and EZH2 (**h**) in **e** and **f**.

2. As a component of PRC1.1 complex, KDM2B recruits the complex to chromatin and promotes deposition of H2AK119ub, which in turn facilitates PRC2 recruitment and subsequent H3K27me3 deposition[18]. To test whether this mechanism contributes to EZH2 recruitment in MB, we performed CUT&RUN for BCOR (PRC1.1-specific subunits) and H2AK119ub (the mark written by PRC1) at 12, 48, and 120 hours post-KDM2B degradation in D283 cells. By 12 hours, BCOR occupancy was markedly reduced and H2AK119ub was dramatically depleted at promoters of upregulated genes, indicating that PRC1.1 recruitment and H2AK119 ubiquitination at these loci are KDM2B dependent (Rebuttal Letter Fig. 26a,b). In contrast, global BCOR occupancy was only modestly reduced and global H2AK119ub levels remained largely unchanged (Rebuttal Letter Fig. 26c,d), indicating either KDM2B-independent PRC1.1 formation[19] or compensation by KDM2B isoforms not targeted by dTAG. Collectively, the rapid loss of BCOR, H2AK119ub, and EZH2 within 12 hours of KDM2B degradation supports a model in which KDM2B promotes locus-specific recruitment of the BCOR/PRC1.1 complex, leading to H2AK119 ubiquitination and subsequent PRC2 recruitment and H3K27me3 deposition at KDM2B-repressed genes.

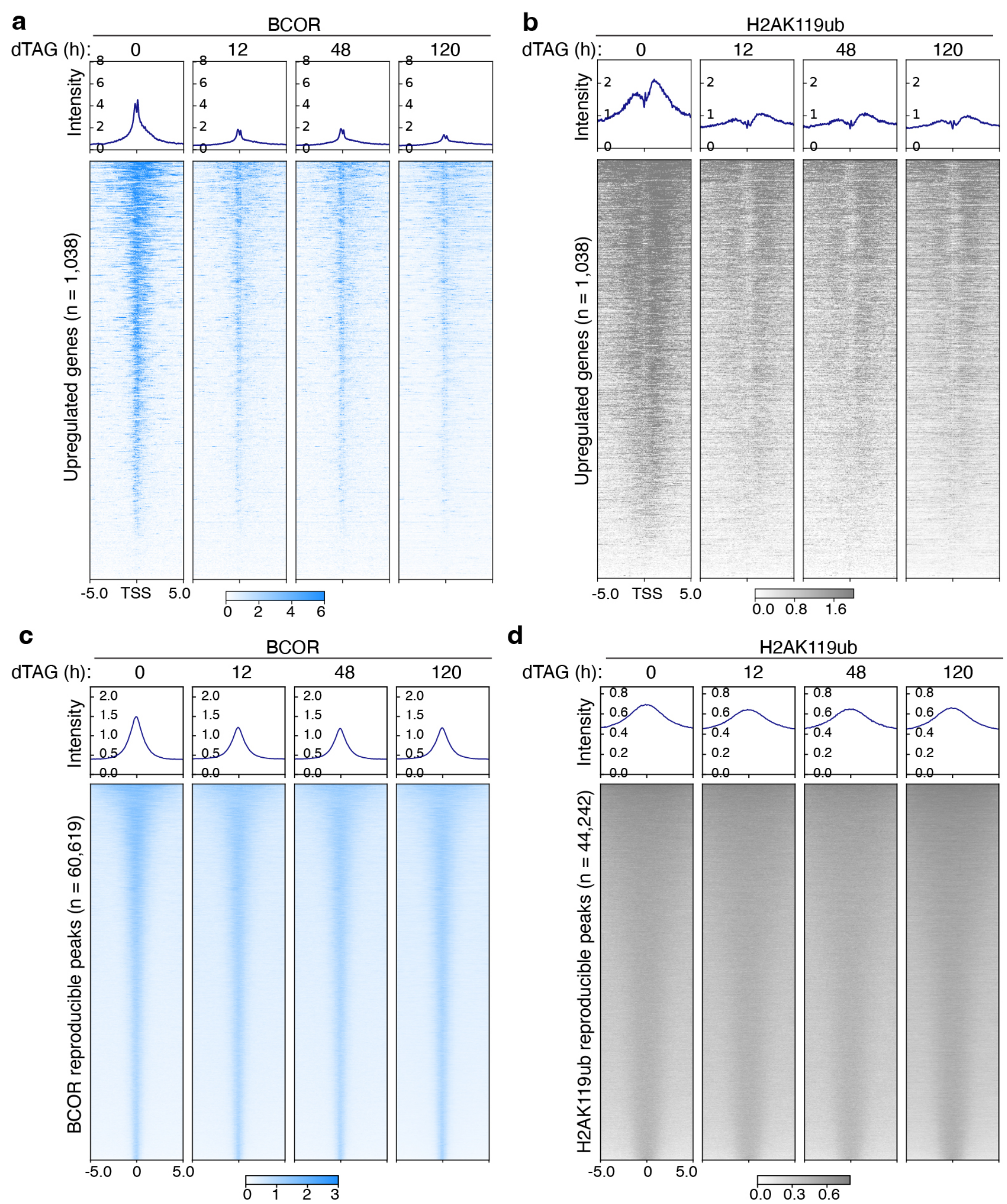

Rebuttal Letter Fig. 26 (From Fig. 6 and Extended Data Fig. 9). a and **b**, CUT&RUN analysis showing changes of BCOR (**a**) and H2AK119ub (**b**) at the promoters of upregulated genes following KDM2B degradation in D283. **c** and **d**, CUT&RUN analysis showing global changes of BCOR (**c**) and H2AK119ub (**d**) in ± 5kb regions around peak center (0) following KDM2B degradation in D283. Curves in the profile plots represent average peak intensity.

3. Next, we evaluated whether PRC1.1 and PRC2 complexes are both functionally important for Group 3 and Group 4 MB. We first examined expression of their subunits and found that multiple PRC1.1 (Rebuttal Letter Fig. 27a) and PRC2 components (Rebuttal Letter Fig. 27b) are highly expressed in Group 3 and Group 4 relative to WNT and SHH MB.

4. We next checked the dependency of relevant histone-modifying complexes by mining DepMap, which systematically identifies genetic dependencies through genome-scale CRISPR loss-of-function screens across a broad panel of pediatric and adult cancer cell lines. We examined the dependency of complexes that regulate H3K4 and H3K27 methylation either directly or indirectly, including all six PRC1 subcomplexes, two PRC2 subcomplexes, three COMPASS complexes, and the CoREST complex, comparing eight Group 3 cell lines (D341, D425, D458, D283, MB002, HDMB03, MED411FH and SJMB016880TO) to 1,182 other cancer cell lines. Only the KDM2B-containing PRC1.1 complex and PRC2.2 complex, which is recruited to chromatin by PRC1.1-mediated H2AK119ub[18], represented Group 3 selective dependencies (Rebuttal Letter Fig. 27c).

5. Consistently, several subunits of PRC1.1 (Rebuttal Letter Fig. 27d) and PRC2 complexes (Rebuttal Letter Fig. 27e) had significantly lower Chronos dependency scores (gene effect) in Group 3 MB relative to all other cancer cell lines, indicating they were selectively required for growth of Group 3 MB. As Group 4 MB is not amenable to *in vitro* culture and is thus not available in DepMap, we were unable to evaluate the dependency of these subunits in Group 4.

In summary, PRC1.1 and PRC2 subunits interacted with KDM2B, localized to promoters of KDM2B-repressed genes, exhibited elevated expression in Group 3 and Group 4 MB, and represented selective dependencies in Group 3 MB. These data are consistent with the high *KDM2B* expression and selective dependency in Group 3 and Group 4 models presented in the current study, supporting a paradigm where KDM2B-coordinated PRC1.1 and PRC2 activity is essential for Group 3/4 MB maintenance. Based on the reviewer's insightful suggestion, we have clarified PRC1.1 as the intermediate step for recruiting PRC2 in the Results and Discussion sections.

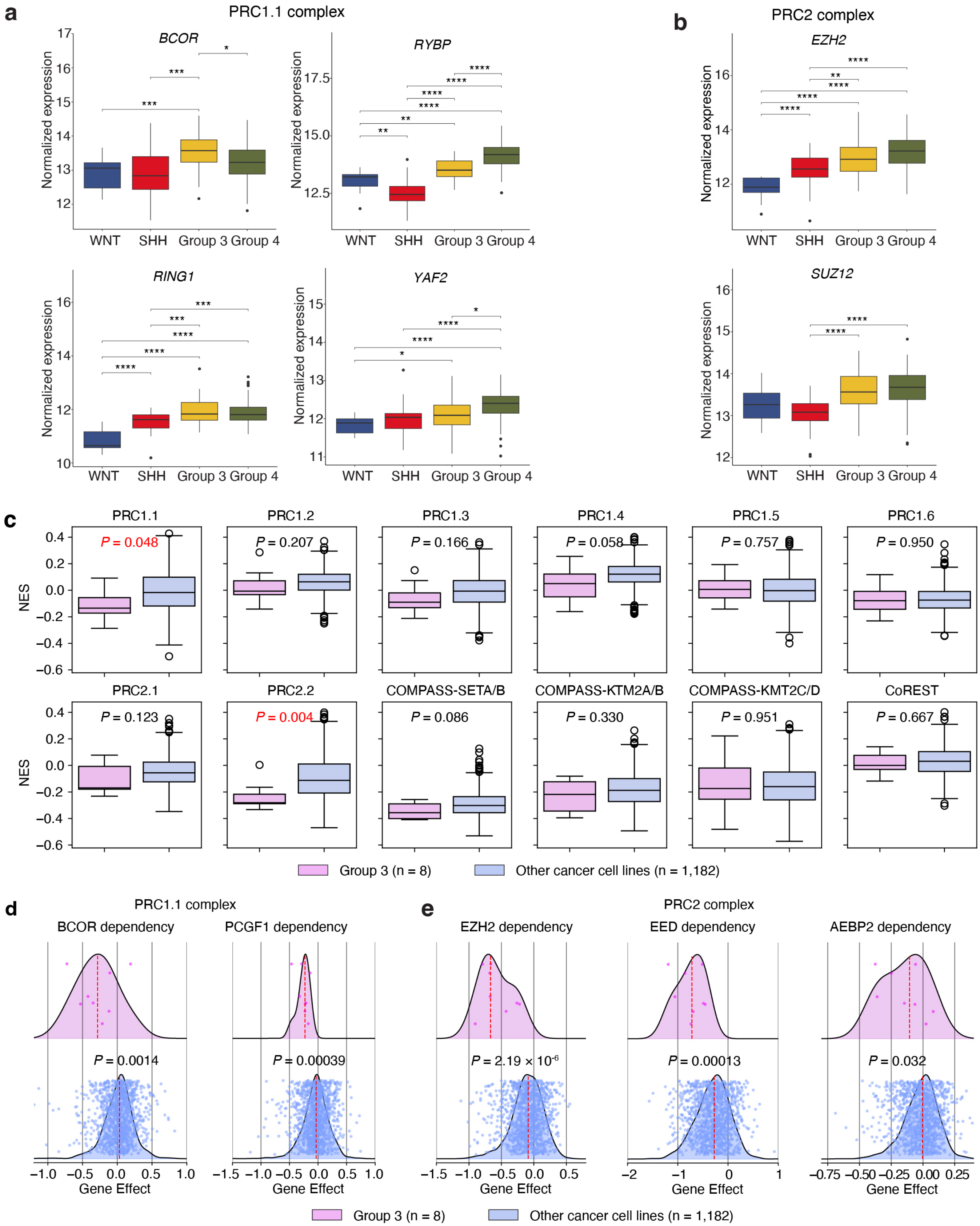
a
PRC1.1 complex
BCOR
RYBP
RING1
YAF2
Normalized expression
WNT
SHH
Group 3
Group 4
b
PRC2 complex
EZH2
SUZ12
c
PRC1.1
P = 0.048
PRC1.2
P = 0.207
PRC1.3
P = 0.166
PRC1.4
P = 0.058
PRC1.5
P = 0.757
PRC1.6
P = 0.950
PRC2.1
P = 0.123
PRC2.2
P = 0.004
COMPASS-SETA/B
P = 0.086
COMPASS-KTM2A/B
P = 0.330
COMPASS-KMT2C/D
P = 0.951
CoREST
P = 0.667
NES
Group 3 (n = 8)
Other cancer cell lines (n = 1,182)
d
PRC1.1 complex
BCOR dependency
P = 0.0014
PCGF1 dependency
P = 0.00039
e
PRC2 complex
EZH2 dependency
P = 2.19 × 10-6
EED dependency
P = 0.00013
AEBP2 dependency
P = 0.032
Gene Effect
Group 3 (n = 8)
Other cancer cell lines (n = 1,182)

Rebuttal Letter Fig. 27 (from Extended Data Fig. 10). a and **b**, Boxplots showing expression of PRC1.1 (**a**) and PRC2 (**b**) subunits across MB subgroups (WNT, n = 15; SHH, n = 46; Group 3, n = 41; Group 4, n = 65). Boxes represent the IQR, center line indicates the median, and whiskers extend to the most extreme data points within 1.5 × IQR. Dots indicate outliers beyond 1.5 × IQR. Statistical significance was assessed using the two-sided t-test, with P adjusted by the Holm method. *, $P_{adj} < 0.05$; **, $P_{adj} < 0.01$; ***, $P_{adj} < 0.001$; ****, $P_{adj} < 0.0001$. **c**, Boxplots showing growth dependency of chromatin modifying complexes comparing Group 3-MB (n = 8) with all other cancer cell lines (n = 1,182) in DepMap. Normalized enrichment scores (NES) were computed from DepMap dependency scores for each line, with lower scores indicating stronger dependencies. Boxes represent the IQR, center line indicates the median, and whiskers extend to the most extreme data points within 1.5 × IQR. Dots indicate outliers beyond 1.5 × IQR. Statistical significance was determined by two-tailed Mann-Whitney U test, with P <0.05 considered statistically significant. **d** and **e**, Gene effect of PRC1.1 (**d**) and PRC2 (**e**) subunits in Group 3-MB (n = 8) and all other cancer cell lines (n = 1,182). A lower gene effect score means that a gene is more likely to be a dependency. The red dashed line indicates median values of gene effect. $P < 0.05$ indicates Group 3 selective dependency.

c. The authors should also plot EZH2 binding in KDM2B KO cells in Figure 6d, matching the same set of KDM2B binding sites, to show the degree of EZH2 binding loss.

Response:

1. We thank the reviewer for this suggestion. In the original Fig. 6d, KDM2B binding sites were defined using SHH-, Group 3-, or Group 4-specific peaks identified from MB PDX samples. To address the reviewer's request, we examined occupancy of EZH2 as well as KDM2B, BCOR, H2AK119ub, and H3K27me3 at these same subgroup-specific KDM2B-bound regions in D283 comparing Ctrl with five days post-KDM2B degradation. Consistent with the Group 3 identity of D283, robust KDM2B enrichment was observed specifically at Group 3-defined peaks, while SHH-and Group 4-specific regions showed minimal KDM2B signal. Following KDM2B degradation, BCOR and H2AK119ub occupancy decreased at Group 3-specific KDM2B-bound regions, whereas no changes were observed at SHH- or Group 4-specific regions. EZH2 occupancy at these subgroup-defined regions did not change appreciably (Rebuttal Letter Fig. 28a,b), suggesting that its recruitment at these regions is largely independent of KDM2B in D283 cells or are compensated by alternative mechanisms (e.g. KDM2B isoforms not targeted by dTAG).

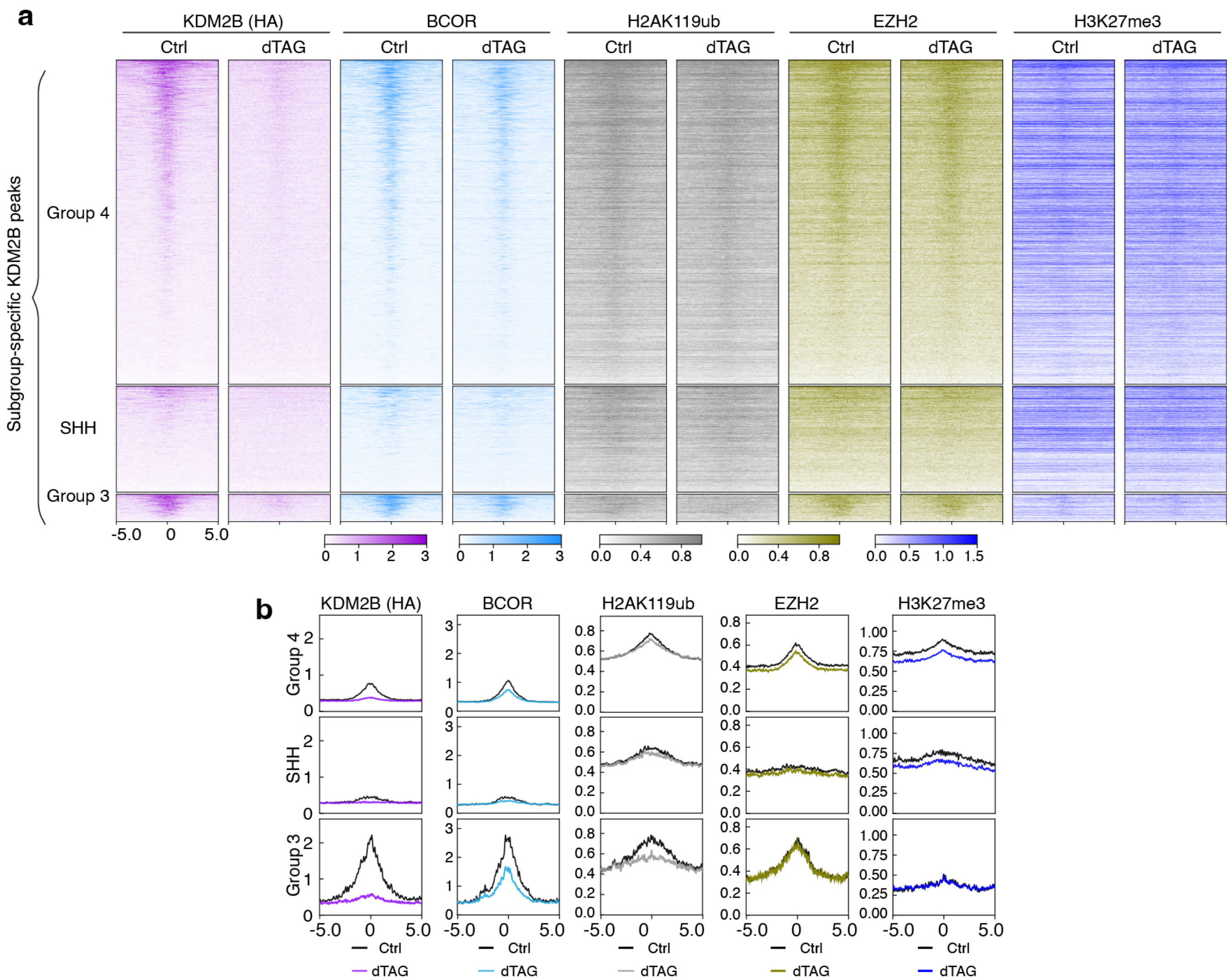

Rebuttal Letter Fig. 28. **a**, CUT&RUN heatmaps showing changes of KDM2B, BCOR, H2AK119ub, EZH2, and H3K27me3 signal at subgroup-specific KDM2B bound regions following KDM2B degradation in Group 3 D283 cells. Subgroup-specific KDM2B bound regions (Group 4, n = 3,591; Group 3, n = 307; SHH, n = 1,175) were determined by CUT&RUN analysis of KDM2B in MB PDXs (**Fig. 6b**). **b**, Profile plot showing average peak intensity of marks in **a**.

2. To directly assess EZH2 loss at functionally relevant KDM2B binding sites in Group 3 cellular context, we next focused on regions co-bound by KDM2B and EZH2 in D283 cells. At these co-occupied sites, we observed decreased BCOR, H2AK119ub, EZH2, and H3K27me3 following KDM2B degradation (Rebuttal Letter Fig. 29a,b), although these changes were modest compared to the pronounced losses observed at promoters of genes transcriptionally upregulated upon KDM2B depletion (Rebuttal Letter Fig. 29c,d).

Together, these analyses indicate that KDM2B-dependent PRC2 recruitment is locus-specific and context-dependent, rather than a uniform feature of all KDM2B-bound regions.

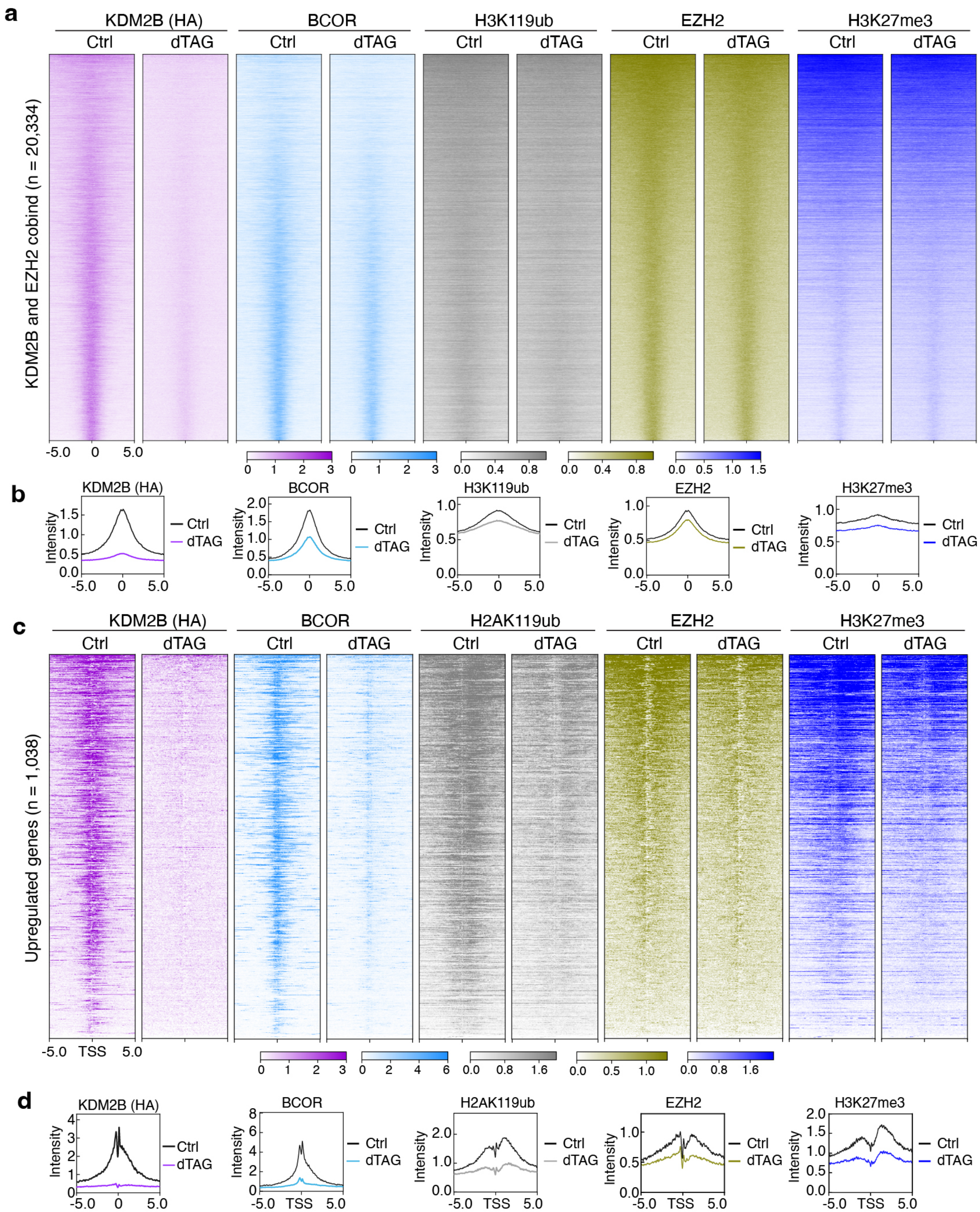

Rebuttal Letter Fig. 29. **a**, CUT&RUN heatmaps showing changes of KDM2B, BCOR, H2AK119ub, EZH2, and H3K27me3 at KDM2B and EZH2 co-bound regions following KDM2B degradation in D283. **b**, Profile plot showing average peak intensity of marks in **a**. **c,** CUT&RUN heatmaps showing changes of KDM2B, BCOR, H2AK119ub, EZH2, and H3K27me3 at the promoters of upregulated genes following KDM2B degradation in D283. **d**, Profile plots showing average peak intensity of marks in **c**.

d. If there is no strong association between PRC2 recruitment and KDM2B, please address alternative explanations for these effects in the discussion section.

Response:

1. We have updated the manuscript to incorporate additional data, including:
    a. The dynamics and specificity of KDM2B, EZH2, BCOR, and H2AK119ub chromatin occupancy following KDM2B degradation (Rebuttal Letter Fig. 25 and 26).
    b. The interaction between KDM2B and multiple components of the PRC1.1 and PRC2 complexes in both Group 3 and Group 4 cellular contexts (Rebuttal Letter Fig. 24).
    c. Increased expression of PRC1.1 and PRC2 subunits in Group 3 and Group 4 (Rebuttal Letter Fig. 27).
    d. Selective dependency of PRC1.1 and PRC2 in Group 3 (Rebuttal Letter Fig. 27).

These data support a model whereby KDM2B-dependent PRC1.1 ubiquitination activity recruits EZH2 preferentially at the promoters of KDM2B-repressed genes, providing in-depth mechanistic insight into how the KDM2B-PRC1.1-PRC2 axis regulates gene expression programs required for Group 3 and Group 4 MB maintenance.

e. As KDM2B is well-known to regulate K4me and K36me, is there a change of K36me over up-regulated genes upon KDM2B KO

Response:

1. The reviewer raises the important point that KDM2B is a demethylase for H3K4me3 and H3K36me2. Our data in the first version of the manuscript suggested that KDM2B regulates gene expression through PRC2-mediated H3K27me3, and we did not initially explore KDM2B demethylase activity[14,16]. In the original manuscript, we used our *KDM2B* targeting gRNAs to deplete KDM2B in Group 3 D283 cells. At seven days post-targeting of *KDM2B*, we isolated histones and performed quantitative mass spectrometry. We did not detect significant changes in global H3K36me2 abundance (Rebuttal Letter Fig. 30a), which was corroborated by immunoblotting (Rebuttal Letter Fig. 30b).

2. To determine whether KDM2B may regulate H3K36me2 chromatin occupancy, we performed CUT&RUN in Group 3 D283 KDM2B-dTAG cells five days post-dTAG treatment. We did not identify changes to H3K36me2 either globally or at the promoters of upregulated genes (Rebuttal Letter Fig. 30 c,d,g,h).

3. In contrast to the lack H3K36me2 changes, we observed slightly reduced H3K27me3 globally and profoundly reduced H3K27me3 at gene promoters upregulated upon KDM2B degradation (Rebuttal Letter Fig. 30 e,f,i,j). These findings suggest that KDM2B does not regulate gene expression through H3K36me2 demethylation in this cellular context, but instead primarily promotes H3K27me3.

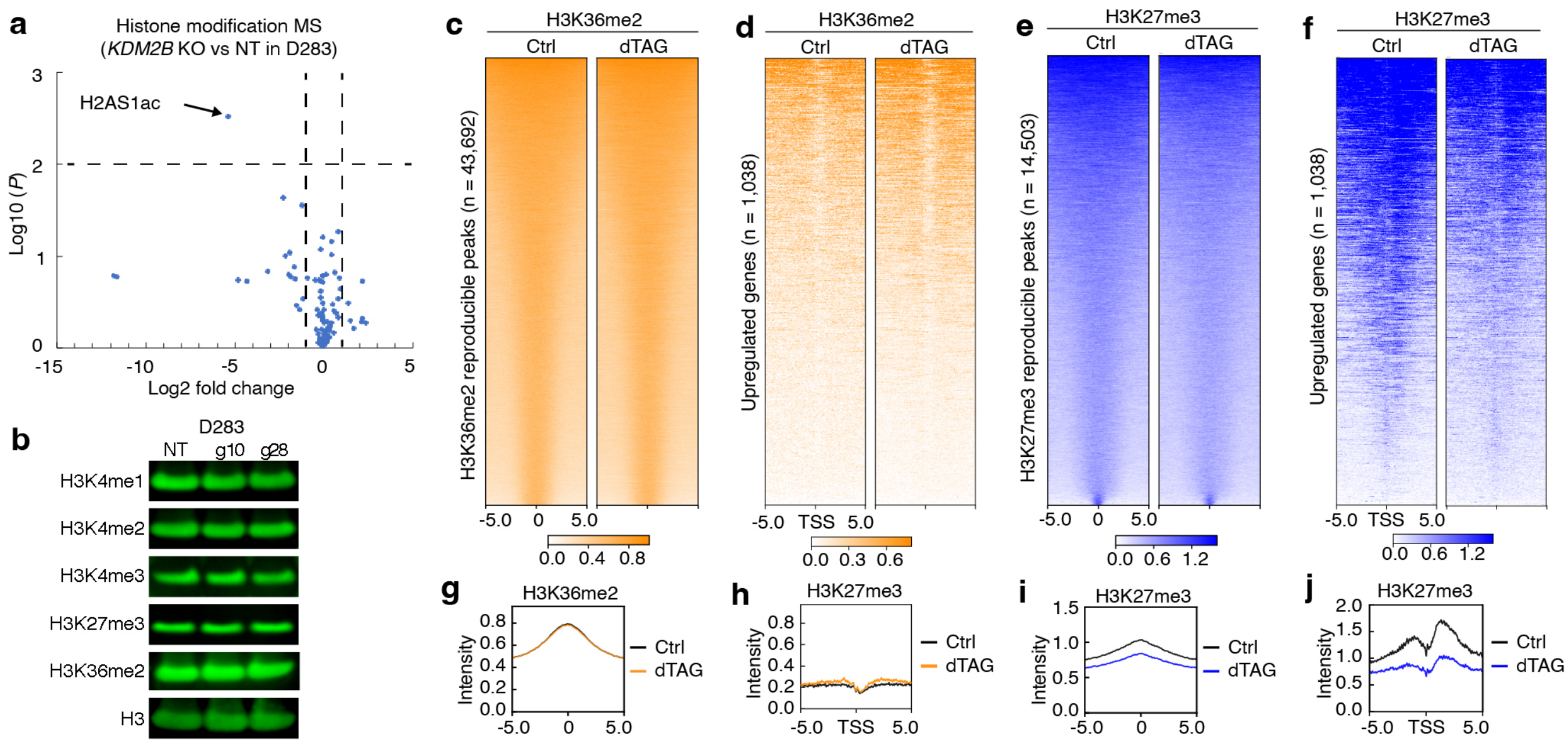

Rebuttal Letter Fig. 30 (from Fig. 5 and Extended Data Fig. 8). a, Volcano plot showing changes of total histone post-translational modifications (PTMs) determined by histone mass spectrometry analysis following *KDM2B* KO in D283. **b**, Immunoblot examining histone PTMs comparing *KDM2B* KO (g10 and g28) with non-targeting (NT) control in D283. Histone H3 was used as an internal control. **c** and **d,** CUT&RUN heatmaps showing H3K36me2 either globally (**c**) or at the promoters of upregulated genes (**d**) following KDM2B degradation in D283. **e** and **f**, CUT&RUN heatmap showing H3K27me3 either globally (**e**) or at promoters of upregulated genes (**f**) following KDM2B degradation in D283. **g-j** Profile plots showing average peak intensity of marks in **c-f**. In the global heatmap, 0 represents peak center.

4). Neurodevelopmental Gene Impact: While the authors proposed that the repression of the neurodevelopmental genes is the major effect of KDM2B-mediated bivalent states, the direct impact of KDM2B on neurodevelopmental genes should be solidified. It is unclear how enriched neurodevelopment genes are in the current analysis in figure 2F.

a. Authors should include a more standard GO analysis (e.g. DAVID, geneontology) with appropriate statistics to better support its enrichment of neurodevelopment genes and have the gene list provided. What percentage of enhBiv genes are neurodevelopment genes?

Response:

We appreciate the reviewer's suggestions to strengthen the link between KDM2B-dependent bivalent states and regulation of neurodevelopmental gene expression. We performed DAVID[29,30] and Enrichr[31] analysis for the Group 4 EnhBiv signature genes. Both tools identified 'Neurogenesis' and 'Transcription' gene sets within the top ten enriched biological processes (Rebuttal Letter Fig. 31a,b). We visualized the top ten enriched clusters from DAVID, including Neurogenesis (ranked 4), CNS development (ranked 9), and Transcription (ranked 6). 20% (n=134) of the 666 EnhBiv signature genes were annotated in the DAVID Neurogenesis gene set (Rebuttal Letter Fig. 31c). We added the updated enrichment analyses, including DAVID enrichment scores and node *P* values to describe

the significance of enrichment, to Fig. 2 and Extended Data Fig. 3. Results of DAVID analysis and the Group 4 EnhBiv neurogenesis gene list (from DAVID analysis) are provided as Supplemental Table 6 and 5, respectively.

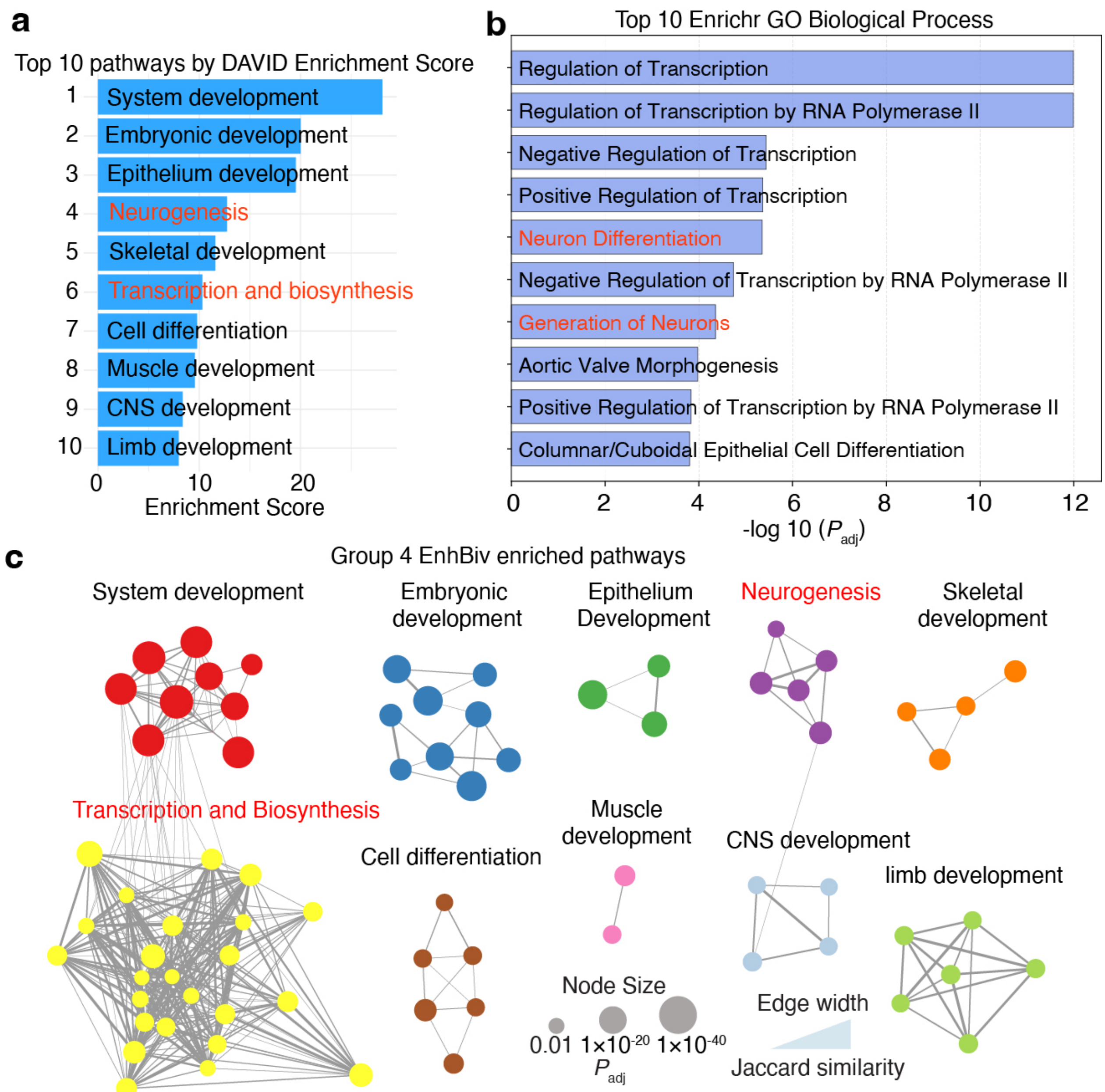

Rebuttal Letter Fig. 31 (from Fig. 2 and Extended Data Fig. 3). **a**, Top 10 enriched pathways of Group 4 EnhBiv signature genes (n=666) from DAVID analysis. Enrichment score represents the geometric mean of EASE scores (modified Fisher's exact test with Benjamini-Hochberg correction) for all GO Biological Process terms within each annotation cluster. **b**, Top 10 enriched pathways of Group 4 EnhBiv signature genes (n = 666) from Enrichr. *P* values were calculated using Fisher's exact test and adjusted using the Benjamini-Hochberg false discovery rate correction. **c**, Visualization of pathways in **a**. Nodes represent GO terms. Edge width represents Jaccard similarity. Node size indicates significance determined by modified Fisher's exact test with Benjamini-Hochberg correction.

b. In Figure 5, gene expression analyses should be subsetted to look specifically at neurodevelopmental enhancer bivalent signature genes instead of all enhancer bivalent signature genes. This would strengthen the link between KDM2B and repression of neurodevelopmental gene regulation.

Response:

We followed the reviewer's excellent suggestion to show enrichment for neurodevelopmental EnhBiv signature genes. The 134 genes with Group 4 enriched EnhBiv state in the neurogenesis cluster were significantly upregulated after KDM2B degradation in Group 3 and after *KDM2B* KO in Group 4 MB models (Rebuttal Letter Fig. 32).

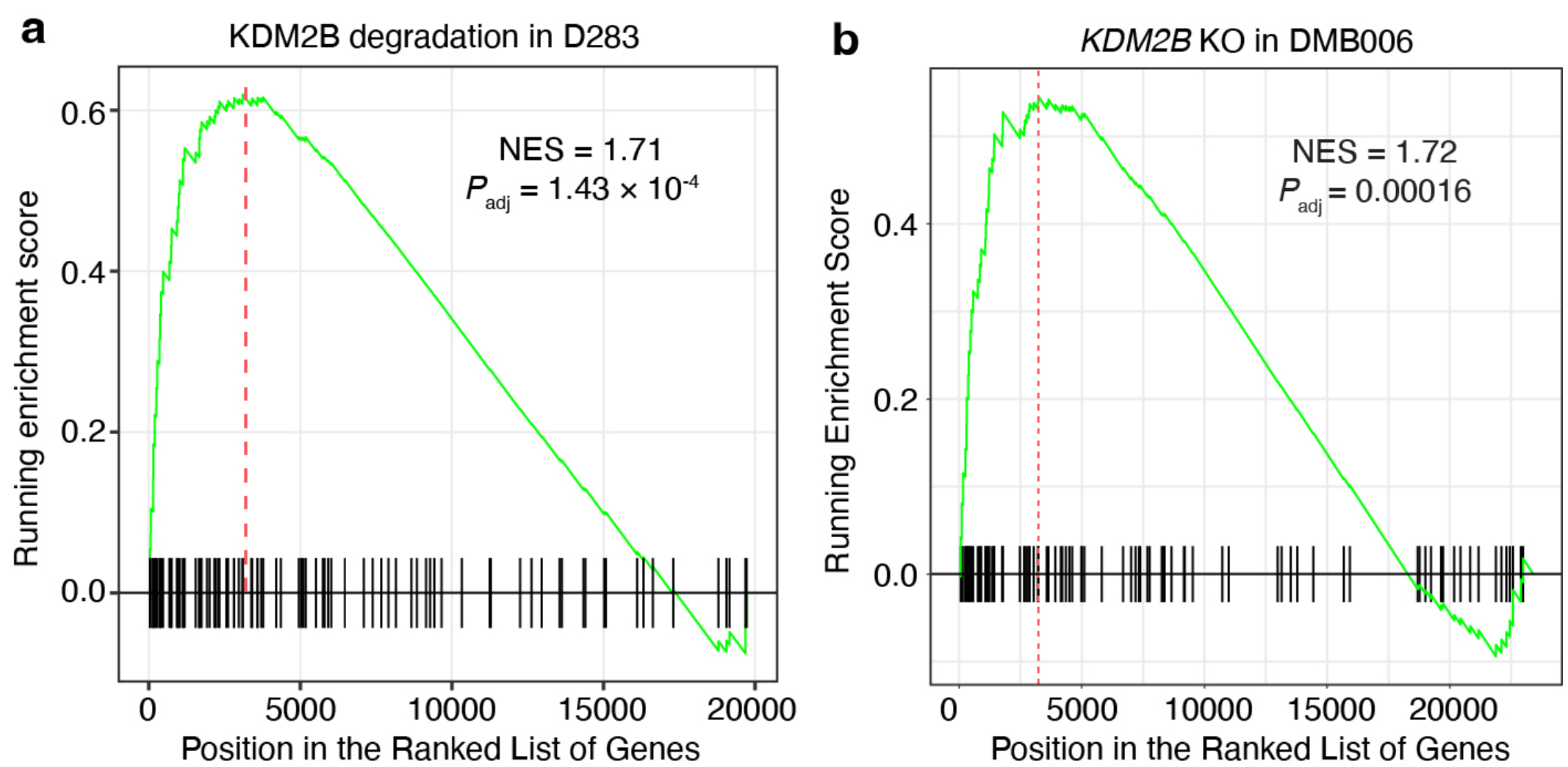

Rebuttal Letter Fig. 32 (from Fig. 5 and Extended Data Fig. 8). Enrichment analysis showing Group 4 EnhBiv-marked neurogenesis genes (n = 134) were predominantly upregulated following KDM2B degradation in D283 (**a**) or KO in DMB006 (**b**). Significance was determined by permutation test with normalized enrichment score (NES) > 1.5 and $P_{adj} < 0.05$ (Benjamini-Hochberg correction) considered statistically significant.

Minor Comments

1. Figure 1A: The coloration for the methylation score is confusing as it is similar to other colors used.

Response:

In the original Extended Data Fig. 1a, we showed both Group 3 and Group 4 methylation scores, which were inversely related. In the revised figure, we removed the Group 3 score and retained only the Group 4 score for clarity. As suggested by the reviewer, we changed the methylation score color to purple to avoid confusion with other color schemes used in this figure. In addition, we adjusted the colors of the six histone marks to match the color schemes used for histone tracks in the heatmaps and at individual gene loci throughout the figures (Rebuttal Letter Fig. 33).

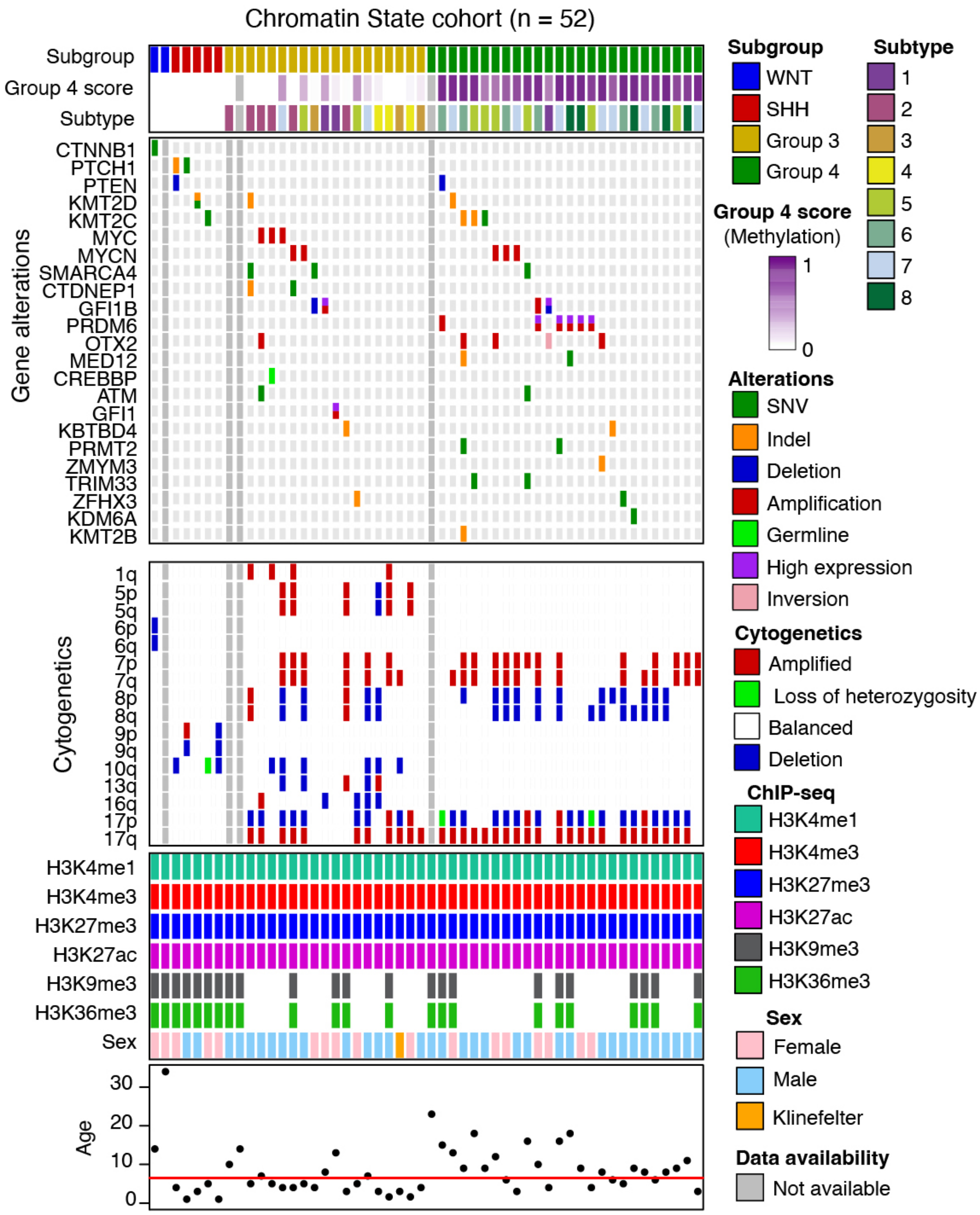

Rebuttal Letter Fig. 33 (From Extended Data Fig. 1). Oncoprint summarizing somatic alterations, cytogenetics, patient age and sex, and data availability of the chromatin state cohort. Red line indicates median age.

2. Figure 2D: Clarify the proportion of all promoters with the enhancer bivalent state in each group. Are Group 4 promoters broadly enriched for the enhancer bivalent state?

Response:

We quantified the number of EnhBiv- or TssBiv-enriched promoters using human MB patient ChIP-seq data. Enrichment was defined as > 20% state coverage in the TSS ± 5kb region of protein-coding genes (n=20,912). Consistent with their genomic coverage, the number of EnhBiv-enriched

promoters was substantially higher in Group 3 and 4 MB compared to WNT and SHH (Rebuttal Letter Fig. 34a). In contrast, the number of TssBiv-enriched promoters was similar across subgroups (Rebuttal Letter Fig. 34b).

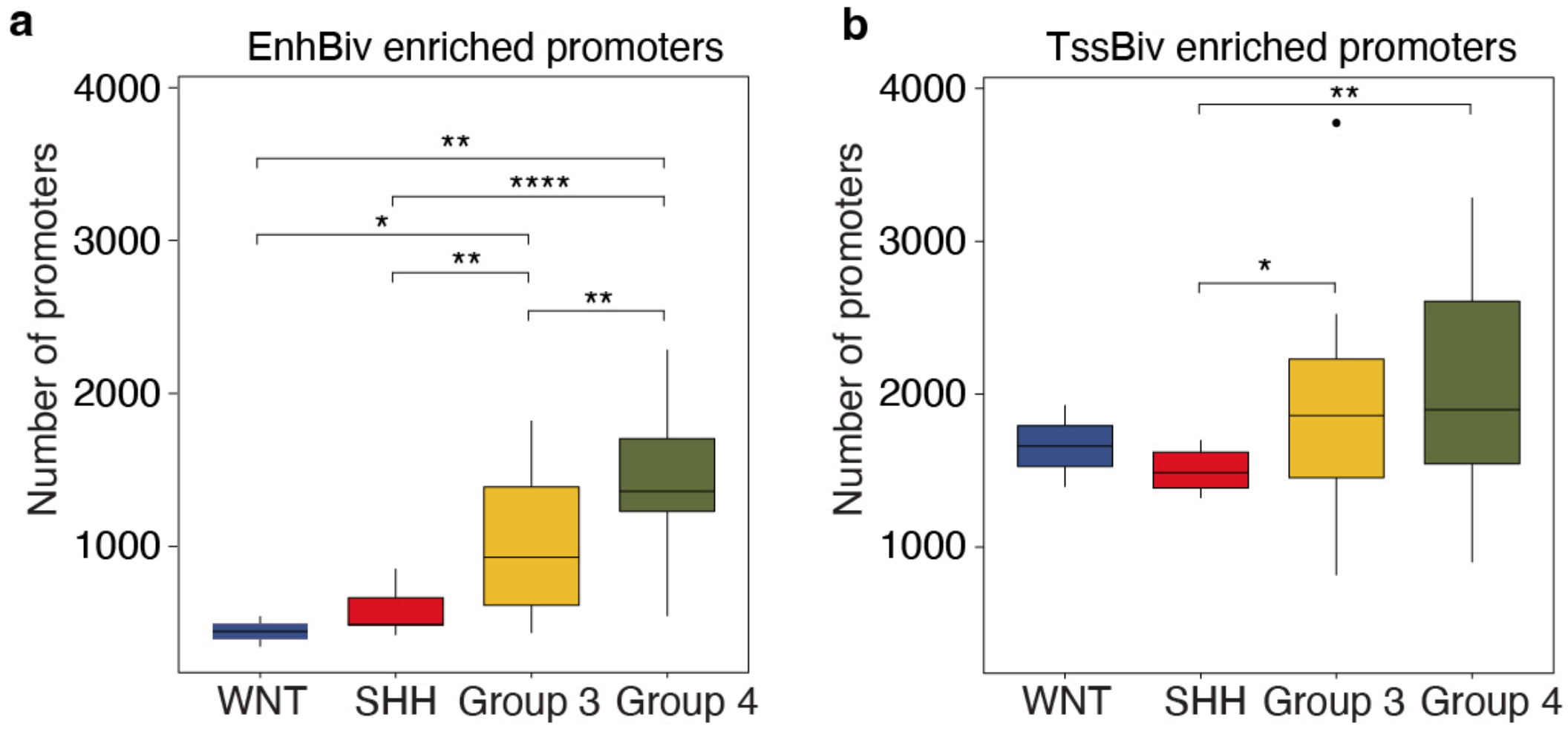

Rebuttal Letter Fig. 34 (From Extended Data Fig. 3). Box plot depicting the number of EnhBiv (**a**) and TssBiv (**b**) enriched promoters in MB patient samples. Boxes represent the IQR, center line indicates the median, and whiskers extend to the most extreme data points within 1.5 × IQR. *P* values were determined by two-tailed Wilcoxon test. *, $P < 0.05$; **, $P < 0.01$; ****, $P < 0.0001$.

3. Figure 2E: The changes are subtle, and the significance appears amplified by using each gene as a separate data point instead of averaging the gene set and comparing each sample. Please note this or use a different representation.

Response:
We apologize for the misunderstanding. As described in the figure legend, the original Fig. 2e is a plot of "Median expression of Group 4 EnhBiv signature genes in MB subgroups (WNT, n=15; SHH, n=46; Group 3, n=41; Group 4, n=65)" where each point describes average expression of Group 4 EnhBiv signature genes for a single sample. We have clarified this by editing the figure legend as below:

"**i**, Expression of Group 4 EnhBiv signature genes (n=666) in MB subgroups. Boxes represent the IQR, center line indicates the median, and whiskers extend to the most extreme data points within 1.5 × IQR. Dots represent samples (WNT, n=15; SHH, n=46; Group 3, n=41; Group 4, n=65). Statistical tests were conducted using the two-sided Wilcoxon test. **, $P < 0.01$; ****, $P < 0.0001$."

4. Figure 2H: The representation is hard to interpret. Increase the alpha and/or move red dots to the front or use a different representation to show density.

Response:
In the revised manuscript, we removed the analyses examining enrichment of Group 4 EnhBiv-targeted neurogenesis genes during human rhombic lip development (original Fig. 2g–l) to incorporate new studies suggested by the reviewers. These additions include substantially expanded mechanistic studies elucidating how KDM2B/PRC1.1/PRC2 cooperate to maintain

repressive transcriptional states required for Group 3/4 MB maintenance, as well as additional *in vitro* and *in vivo* experiments validating the selective dependency of KDM2B in Group 3 and Group 4 tumors. Together, these new studies significantly strengthen the mechanistic depth of the work, and we therefore removed the neurogenesis enrichment analyses to maintain a more focused narrative.

5. Figure S4D: Shouldn't this be labeled KDM2B wild type, not dTAG?

Response:
We used both KDM2B KO and dTAG systems in this study. To ensure the two approaches target the same KDM2B isoforms, we examined the efficiency of *KDM2B* KO gRNAs to deplete the endogenous KDM2B-HA-dTAG fusion protein in the absence of dTAG treatment. We have added more details to the figure legend to reduce confusion:
"**b**, Immunoblot showing depletion of endogenous KDM2B-HA-dTAG protein after CRISPR-mediated editing. KDM2B-HA-dTAG protein was detected using HA-Tag antibody. NV, no virus; NT, non-targeting control; g10 and g28, *KDM2B* KO gRNAs."

6. Figure 4K: Should the x-axis be dTag treatment, not infection?

Response:
We have corrected this error in the updated Extended Data Fig. 6l.

Overall, we are grateful to the reviewer for their helpful suggestions to improve our manuscript by validating KDM2B as a selective dependency in Group 3/4 MB, elucidating the mechanisms of how KDM2B recruits PRC1/PRC2 complexes, and strengthening the connection between this regulatory axis and Group 3/4 MB biology.

Reviewer #3:
Remarks to the Author:
The manuscript by Tao and colleagues utilizes the chromatin landscape definition to investigate and identify unique features and vulnerabilities in medulloblastoma (MB) subgroups. This builds on prior data and publications from their group and others. They find that the bivalent/poised enhancer (EnhBiv) chromatin signature was specifically enriched at Grp4-MB neurodevelopmental gene promoters. These EnhBiv spots are also enriched in KDM2B binding, which the authors find is highly expressed in Grp4-MB. Removal of KDM2B through different mechanisms inhibits in vitro and in vivo growth of MB models, showing their selective dependency on KDM2B.

Overall the manuscript is well written and presented, walking readers through the overall map of chromatin marks, differences among MB subgroups, and then focuses on the EnhBiv marks that are enriched in Grp3/4-MB. It then focuses on KDM2B, performing loss of function studies to demonstrate its role in supporting Grp3/4-MB tumor growth and maintenance, as well as mechanistic studies for its role at these EnhBiv locations, and repression of neurodevelopmental genes.

Response:

We thank Reviewer 3 for the thoughtful and constructive evaluation of our study and for the positive comments regarding the clarity of the presentation and the comprehensive experimental and computational analyses in this study. We appreciate the reviewer's recognition of the identification of subgroup-specific chromatin signatures and vulnerabilities, as well as the mechanistic studies investigating the role of KDM2B in supporting Group 3 and Group 4 medulloblastoma (MB) growth. This work is based on 254 ChIP-seq profiles, of which a subset of the H3K27ac (n = 23), H3K27me3 (n = 1) and H3K4me1 (n = 1) profiles were previously published[32] and the remaining 229 ChIP-seq profiles were newly generated as part of this study. In response to the reviewer's feedback, we performed additional computational analyses to examine the possible cause of high *KDM2B* expression in Group 4 and its association with genetic alterations. We also conducted extensive new experiments that rigorously validate *KDM2B* as a selective dependency in Group 3 and Group 4 MB and delineate sequential recruitment of PRC1.1 and PRC2 complexes as the mechanistic basis underlying KDM2B-associated enhancer bivalency and tumor maintenance. This mechanism was further supported by gain-of-function studies using KDM2B domain mutants. Together, these additions substantially strengthen the manuscript.

I didn't have any major concerns with the manuscript or data presented. The one main obvious question that remains from the manuscript is what causes the elevated expression (or maintained high expression) of KDM2B in Grp4-MB (and maybe in Grp3?). It is touched on in the discussion, but additional data related to KDM2B expression in human brain development may help further support how it comes about if there are no GOF activation mutations. Do the proposed cellular origin of Grp3/4-MBs display high expression of KDM2B? Is it a gene that just doesn't get turned off?

Response:

1. We thank the reviewer for this important question. To determine whether high *KDM2B* expression in Group 4-MB might be linked to its developmental origin, we examined *KDM2B* expression in the developing human cerebellum using bulk RNA-seq datasets[33]. We observed high *KDM2B* expression early in development that decreased during neuronal differentiation and brain maturation (Rebuttal Letter Fig. 35a), consistent with the important role of *KDM2B* in brain development[34-37].

2. We next leveraged a comprehensive human cerebellar single nucleus dataset[38] to examine *KDM2B* expression along the differentiation trajectories of the rhombic lip (RL), the anatomical structure where Group 3 and Group 4 MB originate (Rebuttal Letter Fig. 35b). During granule neuron (GN) lineage differentiation, granule neuron progenitors (GNPs) have the highest *KDM2B* expression, which decreases following the transition to GNs. *KDM2B* expression is not significantly changed during differentiation of the unipolar brush cell (UBC) lineage (Rebuttal Letter Fig. 35c-e). Based on these results, contextualized with the suspected UBC lineage origins of Group 3 and 4 MB, the elevated *KDM2B* expression in Group 4 MB cannot be explained solely by lineage-of-origin.

3. Consistent with high *KDM2B* expression in Group 4, we identified higher H3K4me1 and H3K4me3 signals at the transcriptional start sites (TSS) of *KDM2B* in both Group 3 and Group 4 relative to other subgroups (Rebuttal Letter Fig. 35f). However, the upstream mechanisms responsible for these histone modification differences and the extent to which they contribute to high *KDM2B* expression in Group 4 MB (and to a lesser extent, Group 3) is unclear.

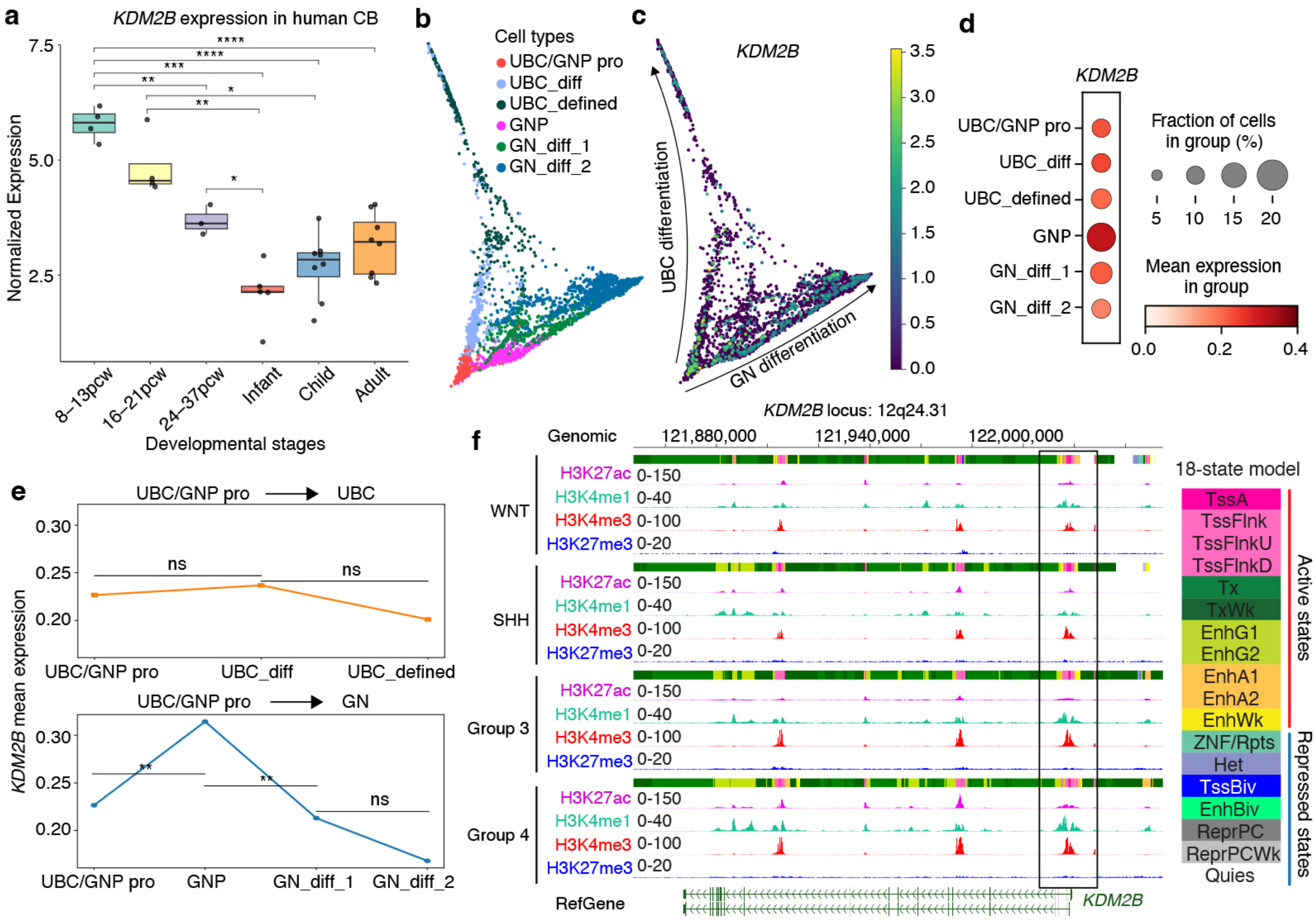

Rebuttal Letter Fig. 35 (from Extended Data Fig. 4 and 5). a, Box plot comparing *KDM2B* expression in human cerebellum samples across developmental stages (8-13 pcw, n = 4; 16-21 pcw, n = 4; 24-37 pcw, n = 3; Infant, n = 5; Child, n = 8; Adult, n = 8). Boxes represent the interquartile range (IQR; 25th–75th percentiles), center line indicates the median, and whiskers extend to the most extreme data points within 1.5 × IQR. Statistical significance was assessed using two-sided t-test and adjusted by the Holm method. *, $P_{adj} < 0.05$; **, $P_{adj} < 0.01$; ***, $P_{adj} < 0.001$; ****, $P_{adj} < 0.0001$. pcw, postconception weeks. **b** and **c**, UMAP showing human cerebellar RL-derived lineages (n = 4,672 cells) (**b**) and *KDM2B* expression (**c**) from a human cerebellar single-nucleus dataset. UBC, unipolar brush cell; GNP, granule neuron progenitor; GN, granule neuron; pro, progenitor; diff, differentiating. **d**, Dot plot showing *KDM2B* expression in RL-derived lineages. Dots were colored by mean expression and sized according to percentage of cells expressing *KDM2B*. **e**, Line plot showing mean *KDM2B* expression along UBC/GNP progenitor to UBC (top) and UBC/GNP progenitor to GN (bottom) differentiation trajectories. Statistical significance was determined by two-tailed Mann-Whitney U test. **, $P < 0.01$; n.s. represents not significant. **f,** Merged histone tracks and aggregated chromatin state at *KDM2B* locus in MB subgroups (WNT, n = 2; SHH, n = 5; Group 3, n = 19; Group 4, n = 26). Black rectangular indicates the region surrounding *KDM2B* TSS.

Moreover, does KDM2B expression correlate or overlap with samples that have other known mutations mentioned in the discussion (GFI, KMT2D, etc)? Again, this is mentioned in the discussion, so there may be some correlation (or anti-correlation) with expression or mutations.

Response:

We thank the reviewer for this excellent question. We examined the correlation between genetic alterations and *KDM2B* expression in Group 3 and Group 4 MB using a previously published dataset[27]. Overall, the gradient of *KDM2B* expression mirrored the biological continuum, with increasing expression along the gradient from Group 3 to Group 4 (Rebuttal Letter Fig. 36 a,b). We observed a significant correlation between several driver alterations and *KDM2B* expression. These included a positive correlation between high *KDM2B* expression and genetic alterations targeting chromatin-modifying genes, including *PRDM6*, *KDM6A*, and *KMT2C* in Group 4 MB (Rebuttal Letter Fig. 36a).

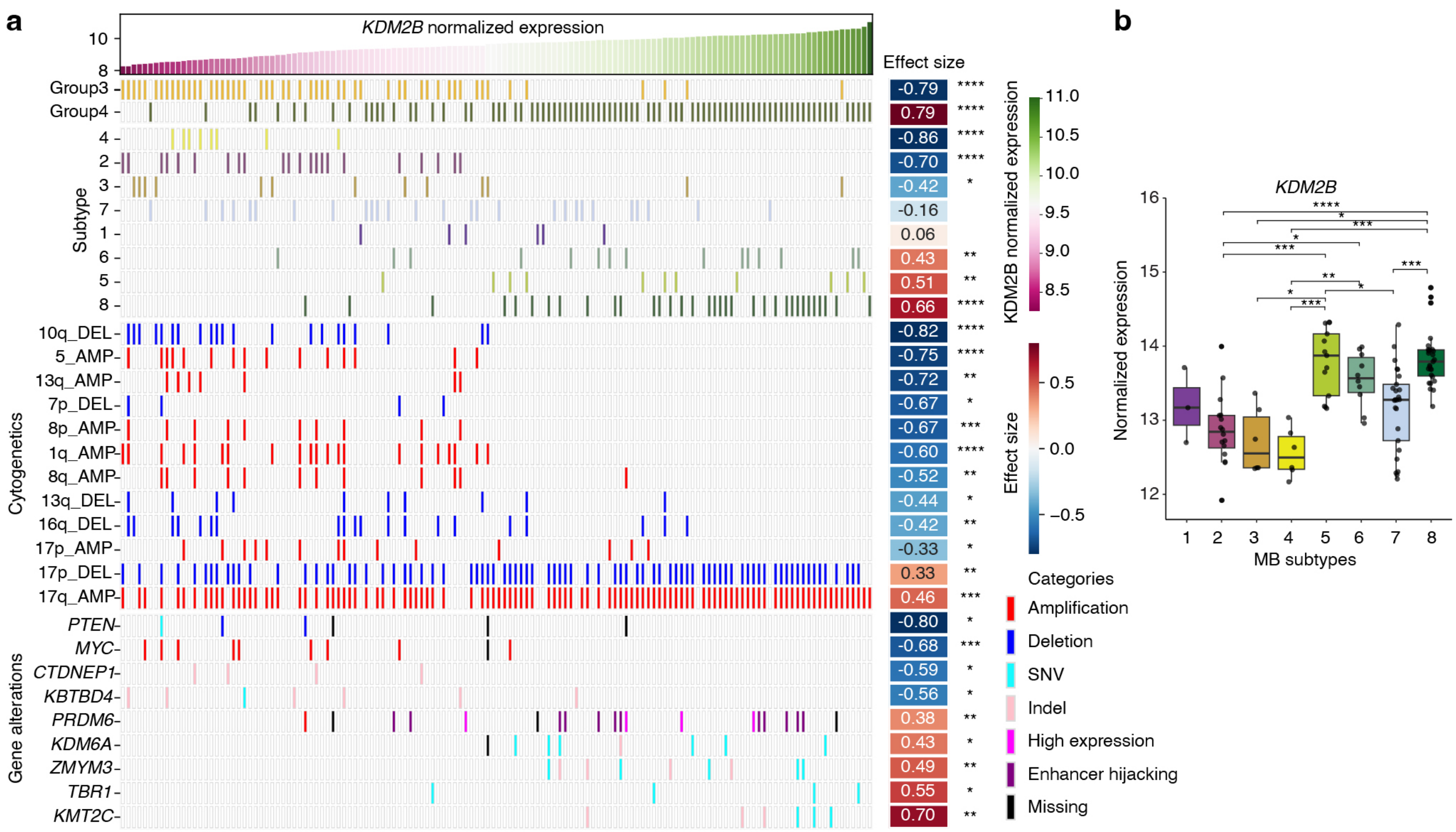

Rebuttal Letter Fig. 36 (from Extended Data Fig. 4). a, Oncoprint showing association of subgroups, subtypes, copy number alterations, and gene alterations with *KDM2B* expression in a cohort of Group 3- and Group 4-MB patients (n = 136). Samples were ordered by increasing *KDM2B* expression. Effect size > 0 indicates positive correlation between the alteration and *KDM2B* expression, while effect size < 0 indicates a negative correlation. Only alterations present in at least three samples with |effect size| > 0.2 and *P* < 0.05 are shown. Two-sided *P* values were determined by permutation testing (100,000 permutations). * $P < 0.05$; ** $P < 0.01$; *** $P < 0.001$; **** $P < 0.0001$. **b**, *KDM2B* expression in eight Group 3 and Group 4 subtypes (1, n = 3; 2, n = 16; 3, n = 6; 4, n = 6; 5, n = 13; 6, n = 10; 7, n = 25; 8, n = 27). Boxes represent IQR, center line indicates the median, and whiskers extend to the most extreme data points within 1.5 × IQR. Statistical significance was assessed using two-sided t-test, with *P* adjusted by the Holm method. * $P_{adj} < 0.05$; ** $P_{adj} < 0.01$; *** $P_{adj} < 0.001$; **** $P_{adj} < 0.0001$.

Last, one suggestion, would be gain of function studies to further support the activity of KDM2B. These may complement the more in depth LOF studies that the authors have performed, but also may be somewhat redundant and difficult to accomplish in a relevant model system.

Response:

1. We thank the reviewer for this important suggestion. To perform *KDM2B* gain-of-function studies, we cloned KDM2B with an HA tag at its C terminus into a doxycycline (Dox) inducible (Tet-On) lentiviral vector expressing a GFP reporter and tested this overexpression system in our Group 3 D283 KDM2B-dTAG cell line. After viral transduction and sorting GFP+ cells, we treated the cells with different concentrations of Dox in the presence of dTAG to ectopically induce KDM2B expression and degrade the endogenous KDM2B-dTAG protein. Immunoblotting showed that KDM2B expression was robustly induced by Dox and reached the highest expression at ~500 ng/ml (Rebuttal Letter Fig. 37a).

2. Since SHH MBs exhibit low genome coverage of EnhBiv and do not depend on *KDM2B* for growth, we tested whether *KDM2B* overexpression in SHH MB patient-derived xenograft (PDX) may change methylation or expression signatures and affect tumor cell growth. We transduced SHH MB PDX (SJNBT3) cells with the Tet-On *KDM2B* lentivirus and sorted GFP+ cells. We treated the cells with 0 or 500 ng/mL Dox for six days and collected cell pellets for immunoblotting, RNA-seq, and DNA methylation array. Immunoblotting confirmed robust Dox-induced KDM2B expression in this cell line (Rebuttal Letter Fig. 37b). Analysis of DNA methylation data indicated that *KDM2B* overexpression did not change subgroup identity (based on MNP v12.8) or clustering in UMAP space (Rebuttal Letter Fig. 37c,d). We only detected two differentially expressed genes in this experimental context, Dox-induced *KDM2B* and *SYNJ2BP-COX16* (Rebuttal Letter Fig. 37e). To check whether *KDM2B* overexpression affected cell growth, we performed a competition assay and found no impact on cell growth during 13 days of *in vitro* culture (Rebuttal Letter Fig. 37f). These data suggest that KDM2B regulates gene expression and cell growth in a cellular context-dependent manner.

3. Group 3 MB models have been generated from cerebellar neural progenitor cells (NPCs) by overexpressing *MYC* alone or in combination with other alterations, such as *GFI1/GFI1B* or *DNp53*[39-42]. To examine whether *KDM2B* overexpression can induce transformation, we transduced cerebellar NPCs isolated from neonatal mice (Postnatal day 6) with Tet-on *KDM2B* lentivirus and performed a competition assay. We treated the cells with Dox to induce *KDM2B* overexpression (Rebuttal Letter Fig. 37g) and followed the cell growth for 14 days *in vitro*. Overexpression of *KDM2B* did not significantly change cell growth *in vitro* (Rebuttal Letter Fig. 37h). Future *in vivo* studies examining the tumorigenic role of *KDM2B*, either alone or in combination with other relevant drivers, will be necessary to rigorously investigate the putative role of *KDM2B* in tumor initiation.

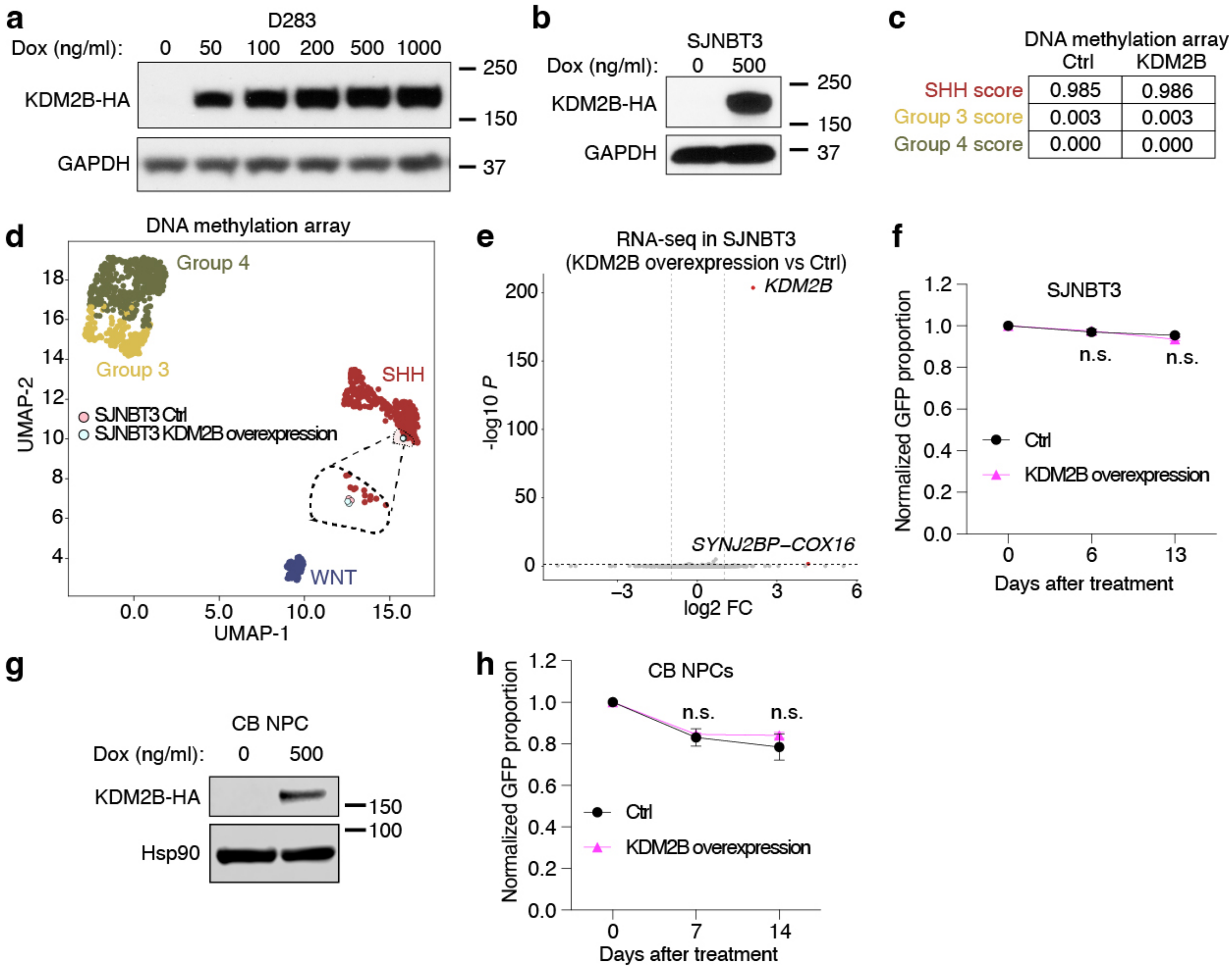

Rebuttal Letter Fig. 37. **a**, Immunoblot showing ectopically expressed KDM2B (detected by HA-Tag antibody). **b**, Immunoblot detecting Dox-induced KDM2B overexpression in SHH MB SJNBT3 cells transduced with Tet-on KDM2B lentivirus. **c**, DNA methylation-derived subgroup scores for SJNBT3 cells with or without *KDM2B* overexpression. **d**, UMAP depicting DNA methylation-based clustering of patient tumors and SJNBT3 cells with or without *KDM2B* overexpression. Clustering was based on 2,000 most variable probes. WNT, n = 104; SHH, n = 320; Group 3, n = 209; Group 4, n = 472. **e**, Volcano plot showing differentially expressed genes determined by RNA-seq in SJNBT3 cells with or without *KDM2B* overexpression. **f**, *In vitro* competition assay in SJNBT3 cells with or without *KDM2B* overexpression. Values represent mean ± standard deviation (SD) determined from two biological replicates. Adjusted *P* was generated by two-way ANOVA with Dunnett's correction. n.s. represents not significant. **g**, Immunoblot showing Dox-induced KDM2B overexpression in cerebellar neural progenitor cells (NPCs) transduced with Tet-on KDM2B lentivirus. **h**, *In vitro* competition assay in cerebellar NPCs with or without *KDM2B* overexpression. Values represent mean ± SD determined from two technical replicates. Adjusted *P* was generated by two-way ANOVA with Šidák correction. Experiments were repeated at least two times. n.s. represents not significant.

Or expression of the different isoforms which the authors discuss may have varying activities?

Response:

1. We thank the reviewer for the excellent suggestion to investigate the mechanisms underlying KDM2B-dependent regulation of gene expression and growth. *KDM2B* has many isoforms consisting of different domains, suggesting that these isoforms may have different functions. In publicly available datasets, *KDM2B* isoforms were annotated by short read sequencing, which is known to be challenging for determining the accurate sequences of isoforms. Many *KDM2B* isoforms have incomplete 5' or 3' sequences, preventing the cloning of these isoforms.

2. Instead of studying the function of different *KDM2B* isoforms, we cloned the long *KDM2B* isoform into a Dox-inducible lentiviral vector and generated mutations targeting either the JmjC demethylase domain or CXXC DNA-binding domain (Rebuttal Letter Fig. 38a) to functionally dissect the mechanisms underlying gene expression regulation and cell growth maintenance. We stably integrated these constructs in Group 3 D283 KDM2B-dTAG cell lines and titrated the Dox concentration to express these mutants at endogenous levels (Rebuttal Letter Fig. 38b). Co-IP confirmed that these mutations did not affect the interaction between KDM2B and PRC1.1 subunits (Rebuttal Letter Fig. 38c).

3. We performed competition assays to examine which KDM2B domains are required to maintain cell growth. Expression of wild-type, JmjC domain deletion, or JmjC domain mutant KDM2B fully rescued the growth defect caused by degradation of endogenous KDM2B, suggesting that the JmjC domain was not required for cell growth. In contrast, expressing the CXXC DNA-binding domain deletion mutant failed to rescue the growth defect (Rebuttal Letter Fig. 38d).

4. We also examined regulation of gene expression by KDM2B domain mutants. Depletion of KDM2B via dTAG treatment predominantly upregulated gene expression, which was largely rescued by re-expressing wild-type, JmjC domain deletion, or JmjC domain mutant KDM2B but not by expressing the CXXC DNA-binding domain deletion mutant (Rebuttal Letter Fig. 38e). These results suggest that DNA binding is required for KDM2B-regulated gene expression and Group 3 MB maintenance. These findings functionally validate our CUT&RUN results that CXXC DNA-binding domain dependent recruitment of PRC1.1 complex mediates subsequent recruitment of EZH2 and H3K27me3 deposition at KDM2B repressed genes.

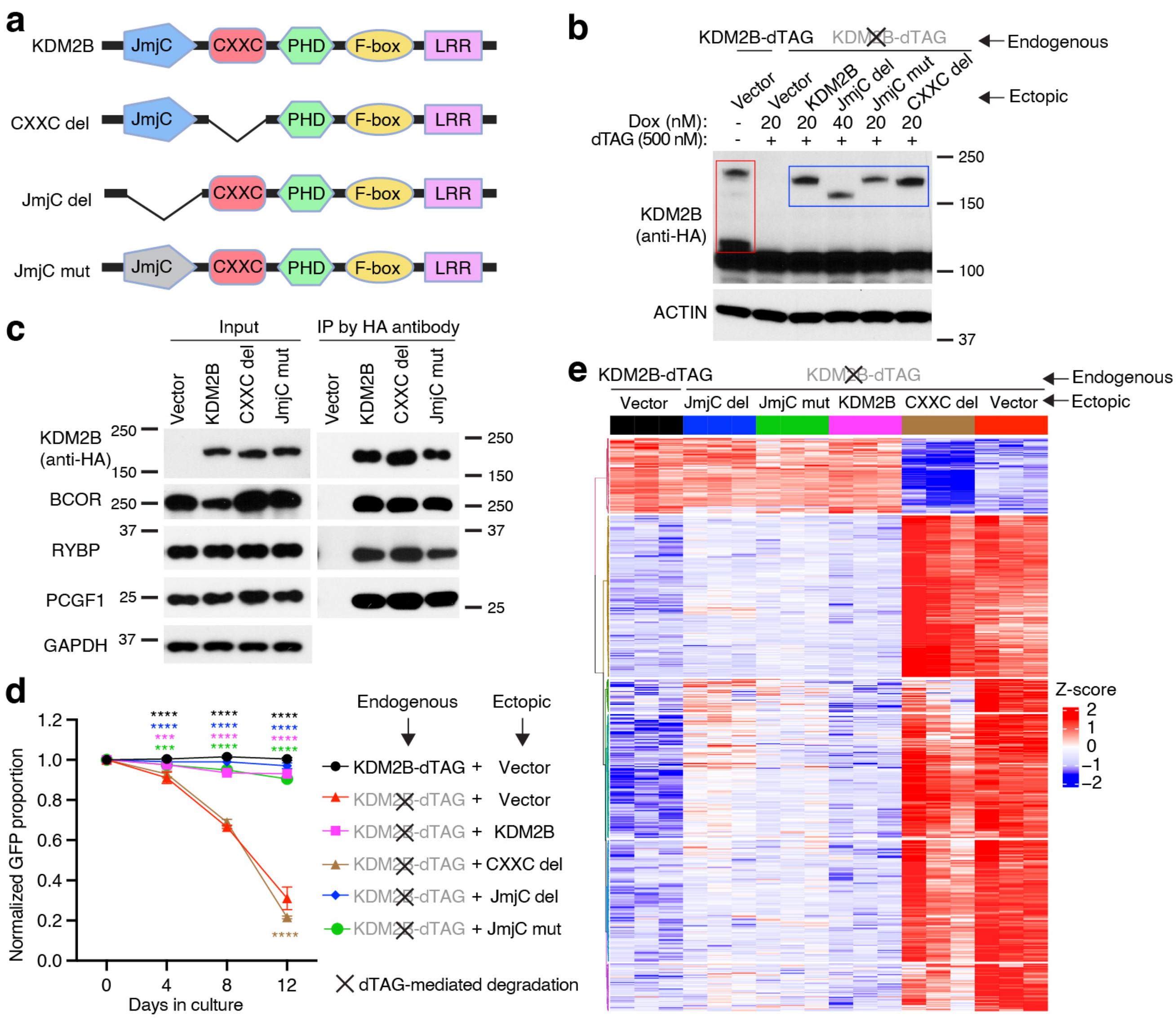

Rebuttal Letter Fig. 38 (from Fig. 7). a, Schematic depicting wild-type and mutant KDM2B. KDM2B protein domains: Jumonji C (JmjC) demethylase domain, CXXC DNA binding domain, Plant homeodomain (PHD) domain, F-box domain and Leucine-Rich Repeat (LRR) domain. Mutant KDM2B were generated by deleting entire CXXC (CXXC del) or JmjC (JmjC del) domain or by introducing mutations in JmjC domain to abolish its demethylase activity (JmjC mut). **b**, Immunoblot of HA-tagged endogenous KDM2B-dTAG (red rectangle) and Dox-induced ectopic wild-type or mutant KDM2B expression (blue rectangle) in D283 KDM2B-dTAG cells treated with or without dTAG or Dox. **c**, Co-immunoprecipitation of ectopically expressed wild-type or mutant KDM2B and endogenous PRC1 subunits (BCOR, RYBP, and PCGF1) in D283 KDM2B-dTAG cells treated with Dox in the presence of dTAG to ectopically induce KDM2B expression and degrade the endogenous KDM2B-dTAG protein. KDM2B was pulled down from nuclear extracts by HA-Tag antibody. **d**, *In vitro* competition assay examining growth rescue of wild-type or mutant KDM2B after degrading the endogenous KDM2B-dTAG protein in D283. Values represent mean ± SD determined from two technical replicates. Adjusted *P* comparing each condition with KDM2B degradation (red line) was generated by two-way ANOVA with Dunnett's correction. ***, $P_{adj} < 0.001$; ****, $P_{adj} < 0.0001$. Experiments were repeated at least two times. **e**, Heatmap showing gene expression changes following KDM2B degradation with or without rescue by wild-type or mutant KDM2B in D283 KDM2B-dTAG cells.

Overall the manuscript provides a detailed story that encompasses going from genetic/epigenetic profiling to mechanistic studies which reveal a therapeutic vulnerability (KDM2B). While targeting this vulnerability still remains a challenge, future research and advancements can address this and other challenges and questions that remain.

Response:
We thank the reviewer for their kind words regarding the significance and impact of these studies and helpful recommendations to elevate the quality of the paper. We have taken the feedback seriously and profoundly expanded mechanistic studies to elucidate how KDM2B/PRC1.1/PRC2 cooperate to maintain repressive transcriptional states required for Group 3/4 MB maintenance.

References:

1. Ernst, J. & Kellis, M. Chromatin-state discovery and genome annotation with ChromHMM. *Nat Protoc* **12**, 2478–2492 (2017).
2. Roadmap Epigenomics Consortium *et al.* Integrative analysis of 111 reference human epigenomes. *Nature* **518**, 317–30 (2015).
3. Filbin, M. & Monje, M. Developmental origins and emerging therapeutic opportunities for childhood cancer. *Nat Med* **25**, 367–376 (2019).
4. Zhu, J. *et al.* Genome-wide chromatin state transitions associated with developmental and environmental cues. *Cell* **152**, 642–54 (2013).
5. Zhao, S., Allis, C.D. & Wang, G.G. The language of chromatin modification in human cancers. *Nat Rev Cancer* **21**, 413–430 (2021).
6. Goulding, M.D., Chalepakis, G., Deutsch, U., Erselius, J.R. & Gruss, P. Pax-3, a novel murine DNA binding protein expressed during early neurogenesis. *EMBO J* **10**, 1135–47 (1991).
7. Ichi, S. *et al.* Role of Pax3 acetylation in the regulation of Hes1 and Neurog2. *Mol Biol Cell* **22**, 503–12 (2011).
8. Nakazaki, H. *et al.* Key basic helix-loop-helix transcription factor genes Hes1 and Ngn2 are regulated by Pax3 during mouse embryonic development. *Dev Biol* **316**, 510–23 (2008).
9. Hovestadt, V. *et al.* Resolving medulloblastoma cellular architecture by single-cell genomics. *Nature* **572**, 74–79 (2019).
10. Vladoiu, M.C. *et al.* Childhood cerebellar tumours mirror conserved fetal transcriptional programs. *Nature* **572**, 67–73 (2019).
11. Williamson, D. *et al.* Medulloblastoma group 3 and 4 tumors comprise a clinically and biologically significant expression continuum reflecting human cerebellar development. *Cell Rep* **40**, 111162 (2022).
12. Farcas, A.M. *et al.* KDM2B links the Polycomb Repressive Complex 1 (PRC1) to recognition of CpG islands. *Elife* **1**, e00205 (2012).
13. He, J. *et al.* Kdm2b maintains murine embryonic stem cell status by recruiting PRC1 complex to CpG islands of developmental genes. *Nat Cell Biol* **15**, 373–84 (2013).
14. Liang, G., He, J. & Zhang, Y. Kdm2b promotes induced pluripotent stem cell generation by facilitating gene activation early in reprogramming. *Nat Cell Biol* **14**, 457–66 (2012).
15. Capper, D. *et al.* DNA methylation-based classification of central nervous system tumours. *Nature* **555**, 469–474 (2018).

16. He, J., Kallin, E.M., Tsukada, Y. & Zhang, Y. The H3K36 demethylase Jhdm1b/Kdm2b regulates cell proliferation and senescence through p15(Ink4b). *Nat Struct Mol Biol* **15**, 1169–75 (2008).
17. Wong, S.J. *et al.* KDM2B Recruitment of the Polycomb Group Complex, PRC1.1, Requires Cooperation between PCGF1 and BCORL1. *Structure* **24**, 1795–1801 (2016).
18. Blackledge, N.P. *et al.* Variant PRC1 complex-dependent H2A ubiquitylation drives PRC2 recruitment and polycomb domain formation. *Cell* **157**, 1445–1459 (2014).
19. Wang, Z. *et al.* A Non-canonical BCOR-PRC1.1 Complex Represses Differentiation Programs in Human ESCs. *Cell Stem Cell* **22**, 235–251 e9 (2018).
20. Dharia, N.V. *et al.* A first-generation pediatric cancer dependency map. *Nat Genet* **53**, 529–538 (2021).
21. Smith, K.S. *et al.* Unified rhombic lip origins of group 3 and group 4 medulloblastoma. *Nature* **609**, 1012–1020 (2022).
22. Hendrikse, L.D. *et al.* Failure of human rhombic lip differentiation underlies medulloblastoma formation. *Nature* **609**, 1021–1028 (2022).
23. Haldipur, P. *et al.* Spatiotemporal expansion of primary progenitor zones in the developing human cerebellum. *Science* **366**, 454–460 (2019).
24. Tao, R. *et al.* Arrested development: the dysfunctional life history of medulloblastoma. *Genes Dev* **39**, 4–17 (2025).
25. Banito, A. *et al.* The SS18-SSX Oncoprotein Hijacks KDM2B-PRC1.1 to Drive Synovial Sarcoma. *Cancer Cell* **33**, 527–541 e8 (2018).
26. Cavalli, F.M.G. *et al.* Intertumoral Heterogeneity within Medulloblastoma Subgroups. *Cancer Cell* **31**, 737–754 e6 (2017).
27. Northcott, P.A. *et al.* The whole-genome landscape of medulloblastoma subtypes. *Nature* **547**, 311–317 (2017).
28. Cao, Q. *et al.* The central role of EED in the orchestration of polycomb group complexes. *Nat Commun* **5**, 3127 (2014).
29. Sherman, B.T. *et al.* DAVID: a web server for functional enrichment analysis and functional annotation of gene lists (2021 update). *Nucleic Acids Res* **50**, W216–W221 (2022).
30. Huang da, W., Sherman, B.T. & Lempicki, R.A. Systematic and integrative analysis of large gene lists using DAVID bioinformatics resources. *Nat Protoc* **4**, 44–57 (2009).
31. Xie, Z. *et al.* Gene Set Knowledge Discovery with Enrichr. *Curr Protoc* **1**, e90 (2021).
32. Lin, C.Y. *et al.* Active medulloblastoma enhancers reveal subgroup-specific cellular origins. *Nature* **530**, 57–62 (2016).
33. Miller, J.A. *et al.* Transcriptional landscape of the prenatal human brain. *Nature* **508**, 199–206 (2014).
34. Fukuda, T., Tokunaga, A., Sakamoto, R. & Yoshida, N. Fbxl10/Kdm2b deficiency accelerates neural progenitor cell death and leads to exencephaly. *Mol Cell Neurosci* **46**, 614–24 (2011).
35. Zhang, B. *et al.* KDM2B regulates hippocampal morphogenesis by transcriptionally silencing Wnt signaling in neural progenitors. *Nat Commun* **14**, 6489 (2023).
36. Gao, Y. *et al.* Impaired KDM2B-mediated PRC1 recruitment to chromatin causes defective neural stem cell self-renewal and ASD/ID-like behaviors. *iScience* **25**, 103742 (2022).

37. van Oirsouw, A.S.E. *et al.* KDM2B variants in the CxxC domain impair its DNA-binding ability and cause a distinct neurodevelopmental syndrome. *Hum Mol Genet* **34**, 1353–1367 (2025).
38. Sepp, M. *et al.* Cellular development and evolution of the mammalian cerebellum. *Nature* **625**, 788–796 (2024).
39. Tao, R. *et al.* MYC Drives Group 3 Medulloblastoma through Transformation of Sox2(+) Astrocyte Progenitor Cells. *Cancer Res* **79**, 1967–1980 (2019).
40. Northcott, P.A. *et al.* Enhancer hijacking activates GFI1 family oncogenes in medulloblastoma. *Nature* **511**, 428–34 (2014).
41. Pei, Y. *et al.* An animal model of MYC-driven medulloblastoma. *Cancer Cell* **21**, 155–67 (2012).
42. Kawauchi, D. *et al.* A mouse model of the most aggressive subgroup of human medulloblastoma. *Cancer Cell* **21**, 168–80 (2012).

Reviewer #1 (Remarks to the Author):

The authors have addressed my questions and concerns. I suggest that the rebuttal figures be appropriately integrated into the manuscript, as they would provide a more comprehensive presentation of the study and offer additional insight into the function of KDM2B in medulloblastoma.

Response:

We are grateful to the reviewer for their thoughtful suggestions throughout the review process, which have substantially improved the manuscript. Owing to the space limitations of *Nature Genetics* articles and the focus of the study, we did not initially include all data generated during revision. As requested, we have now incorporated several rebuttal-only figures into the manuscript. Specifically, we have added the enrichment analysis showing upregulation of Group 4 EnhBiv signature genes (n = 666) following KDM2B knockout in D283 cells (Rebuttal Letter Fig. 8a) and the Venn diagram depicting the overlap between Group 4 EnhBiv signature genes and differentially expressed genes following KDM2B knockout (Rebuttal Letter Fig. 8d). These analyses, which support a role of KDM2B in repressing the expression of Group 4 EnhBiv signature genes, are now included as Extended Data Fig. 7h,i. We have also added the CUT&RUN analysis performed in D283 cells following EZH2 inhibitor treatment (Rebuttal Letter Fig. 17) as Extended Data Fig. 9g, demonstrating that EZH2 contributes to EnhBiv formation at KDM2B target genes.

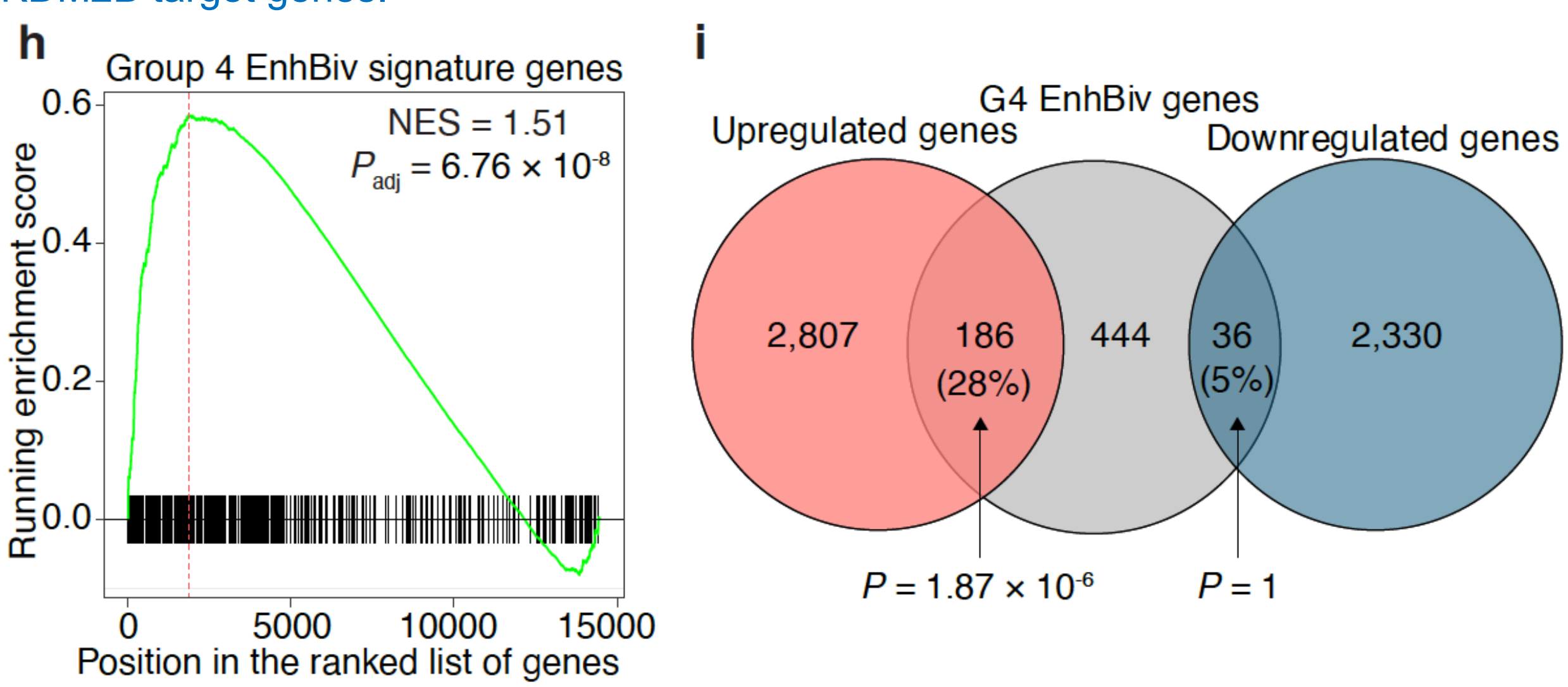

Extended Data Figure 7

h, Enrichment analysis showing upregulation of Group 4 EnhBiv signature genes (n = 666) following *KDM2B* knockout in D283. Significance was determined by the two-sided permutation test (n = 1,000). Gene sets with a normalized enrichment score (NES) > 1.5 and P_{adj} < 0.05 (Benjamini-Hochberg adjusted) were considered statistically significant. **i**, Venn diagram depicting overlap between 666 Group 4 EnhBiv signature genes and genes upregulated (log2FC > 0, FDR < 0.05) or downregulated (log2FC < 0, FDR < 0.05) upon *KDM2B* knockout in D283 cells. *P* was determined by the two-sided hypergeometric test.

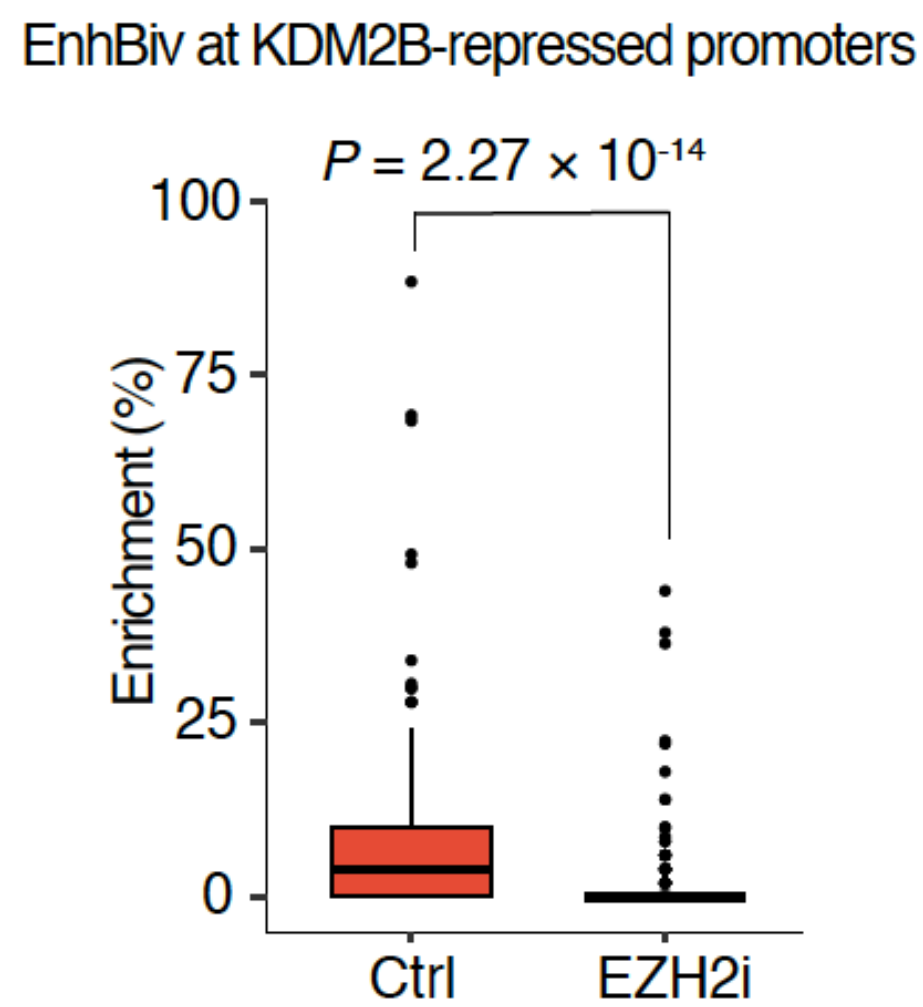

Extended Data Figure 9
g, Loss of EnhBiv state at KDM2B repressed promoters (genes upregulated upon KDM2B degradation) after treating D283 cells with EZH2 inhibitor (EZH2i) for 120 h. Only promoters (n = 141) with detected EnhBiv state enrichment are included in the analysis. Boxes represent the IQR, center line indicates the median, and whiskers extend to the most extreme data points within 1.5 × IQR. Dots indicate outliers beyond 1.5 × IQR. *P* value determined by the two-sided t-test was shown.

Reviewer #2 (Remarks to the Author):

This study by Northcott and colleagues is an important contribution to pediatric cancer and cancer epigenetics. I appreciate the authors extensive efforts to respond to reviewer comments. The core finding of KDM2B dependency in MB subtype is a strong and important result. The authors might consider softening mechanistic claims on downstream mechanisms, given the complexity of the area. I recommend acceptance to NG.

Response:
We sincerely thank the reviewer for the thoughtful and encouraging comments and for recognizing the significance of our findings. We agree that Polycomb-mediated chromatin regulation is highly complex and context dependent. Our study focuses specifically on KDM2B-dependent regulation of chromatin and gene expression in the context of medulloblastoma and does not exclude the contribution of additional regulatory mechanisms that are independent of KDM2B or operate in other cellular contexts. In response to the reviewer's suggestion, we have revised the Discussion to soften our mechanistic conclusions regarding downstream regulation (see underlined text below):

“We assessed Polycomb complex recruitment kinetics and dissected KDM2B domain-specific functions. Empowered by the dTAG system, we observed rapid loss of BCOR, H2AK119ub, and EZH2 at promoters of KDM2B repressed genes within hours of KDM2B degradation, indicating impaired recruitment of PRC1.1/PRC2 by KDM2B rather than indirect effects of prolonged depletion. This mechanism was validated by functional

studies of KDM2B domain mutants which determined a requirement for the CXXC domain, whereas demethylase activity was dispensable. Of note, our study focused on KDM2B-dependent gene regulation but does not exclude PRC1.1-independent mechanisms of Polycomb repression. Together, these findings provide mechanistic insights into how aberrant KDM2B-dependent recruitment of Polycomb promotes chromatin bivalency and sustains malignancy in Group 3- and 4-MB."

Reviewer #3 (Remarks to the Author):

The authors have done a thorough job of addressing the minor concerns I brought up during the initial review. I don't have any other comments or concerns. The revised manuscript includes new experimental data that strengthens the story for readers.

Response:

We sincerely thank the reviewer for the thoughtful suggestions that helped strengthen the manuscript during the revision process. We greatly appreciate the reviewer's positive assessment and support for the revised manuscript.
