# Supplementary material for: Divergent medulloblastoma chromatin states disclose KDM2B as a selective dependency
Source: Nat Genet. 2026 Sep 24;58(10):2590–604. doi: 10.1038/s41588-026-02745-1 (PMC13649585; doi:10.1038/s41588-026-02745-1)
Supplement: Supplementary file 5 [file 41588_2026_2745_MOESM5_ESM.pdf]

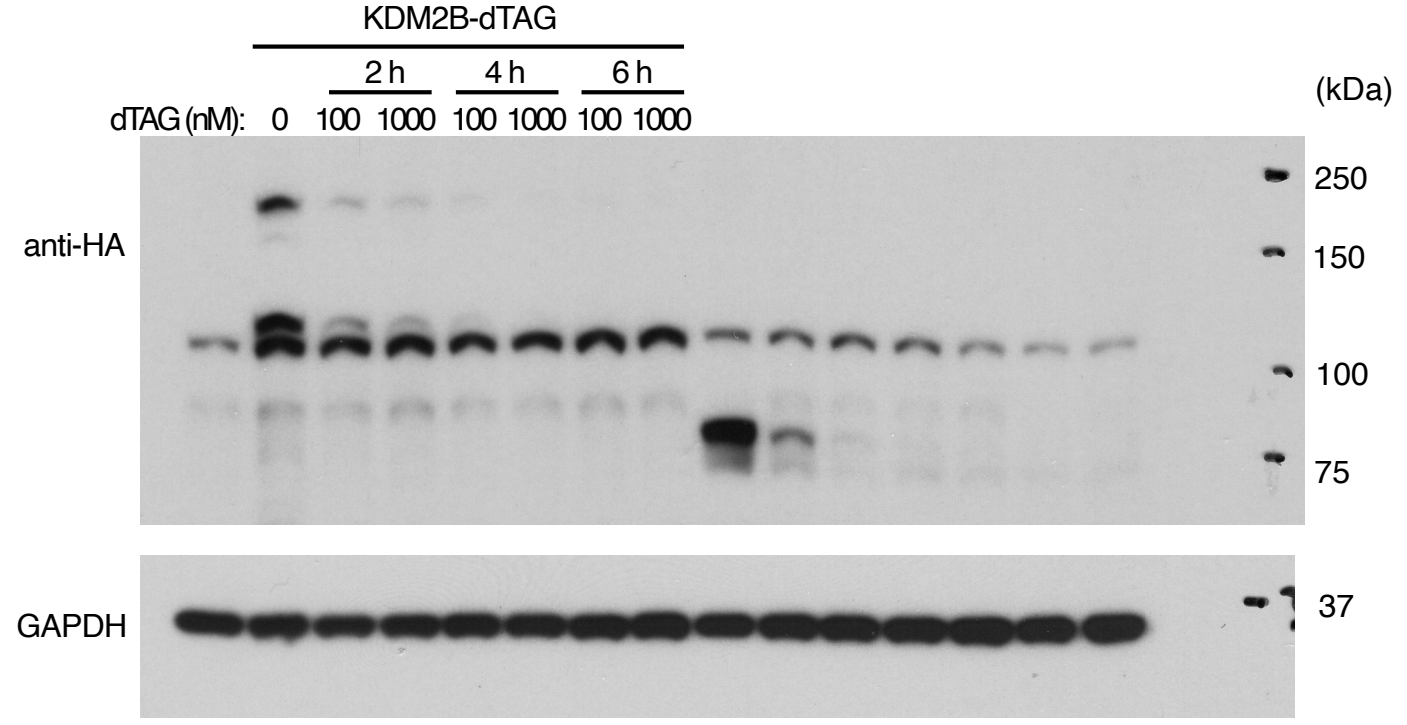
KDM2B-dTAG
2 h
4 h
6 h
dTAG (nM):
0
100
1000
100
1000
100
1000
(kDa)
anti-HA
250
150
100
75
GAPDH
37
