# Supplementary material for: Divergent medulloblastoma chromatin states disclose KDM2B as a selective dependency
Source: Nat Genet. 2026 Sep 24;58(10):2590–604. doi: 10.1038/s41588-026-02745-1 (PMC13649585; doi:10.1038/s41588-026-02745-1)
Supplement: Supplementary file 6 [file 41588_2026_2745_MOESM6_ESM.pdf]

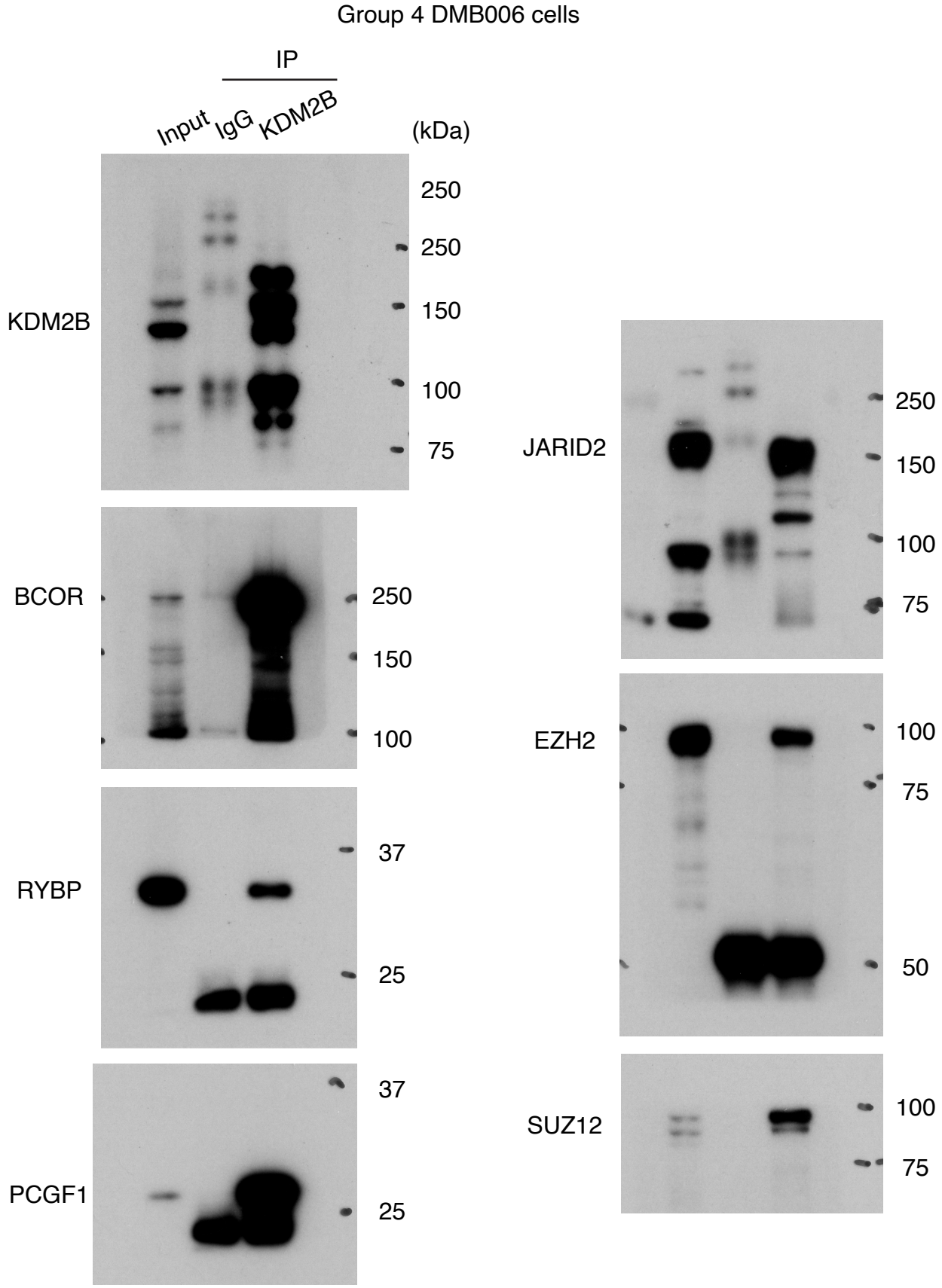
Group 4 DMB006 cells
IP
Input
IgG
KDM2B
(kDa)
KDM2B
250
250
150
100
75
BCOR
250
150
100
RYBP
37
25
PCGF1
37
25
JARID2
250
150
100
75
EZH2
100
75
50
SUZ12
100
75
