# Supplementary material for: Divergent medulloblastoma chromatin states disclose KDM2B as a selective dependency
Source: Nat Genet. 2026 Sep 24;58(10):2590–604. doi: 10.1038/s41588-026-02745-1 (PMC13649585; doi:10.1038/s41588-026-02745-1)
Supplement: Supplementary file 7 [file 41588_2026_2745_MOESM7_ESM.pdf]

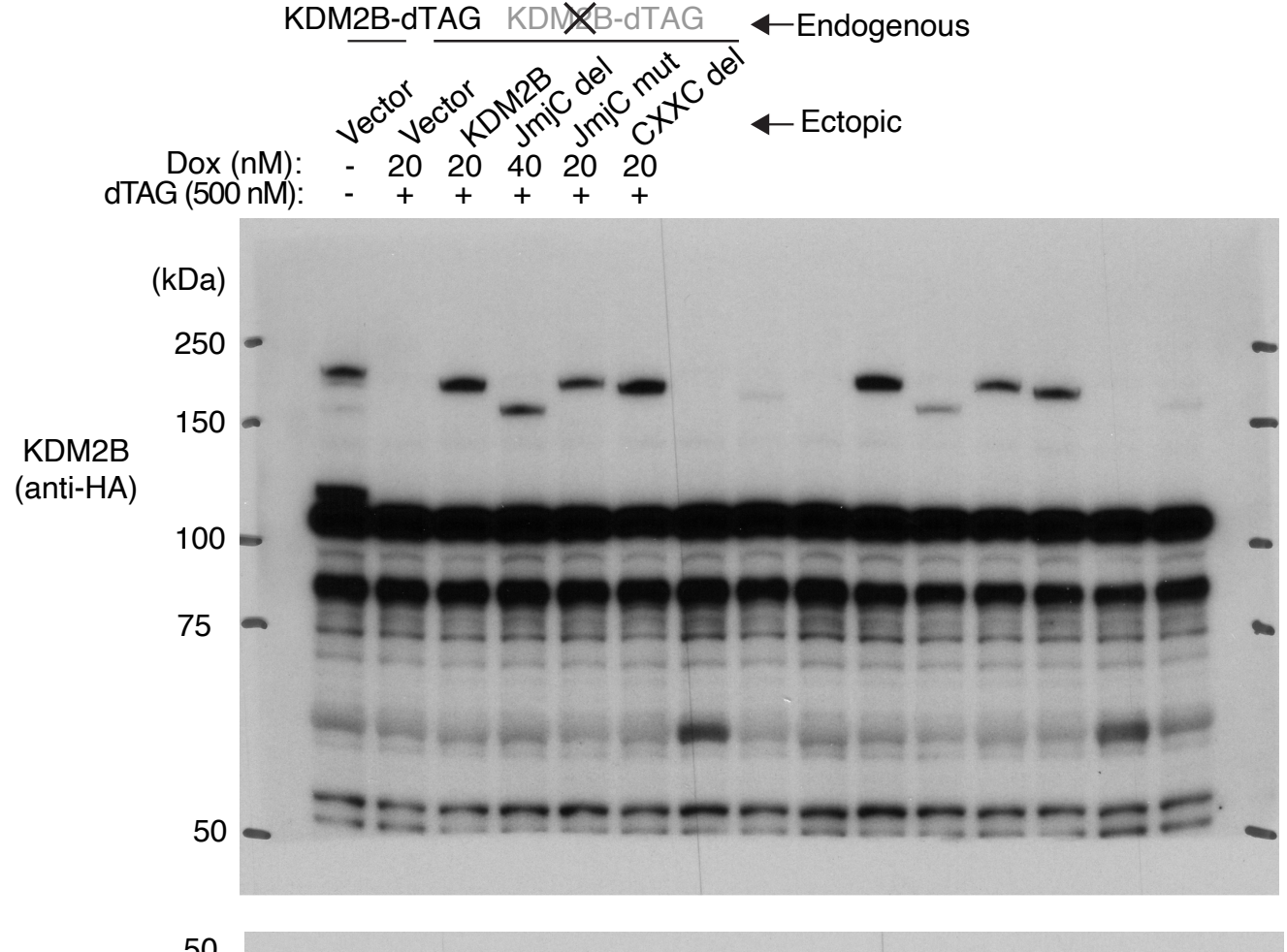
KDM2B-dTAG
KDM2B-dTAG
Endogenous
Vector
Vector
KDM2B
JmjC del
JmjC mut
CXXC del
Ectopic
Dox (nM): - 20 20 40 20 20
dTAG (500 nM): - + + + + +
(kDa)
250
150
100
75
50
KDM2B
(anti-HA)
50
ACTIN
37

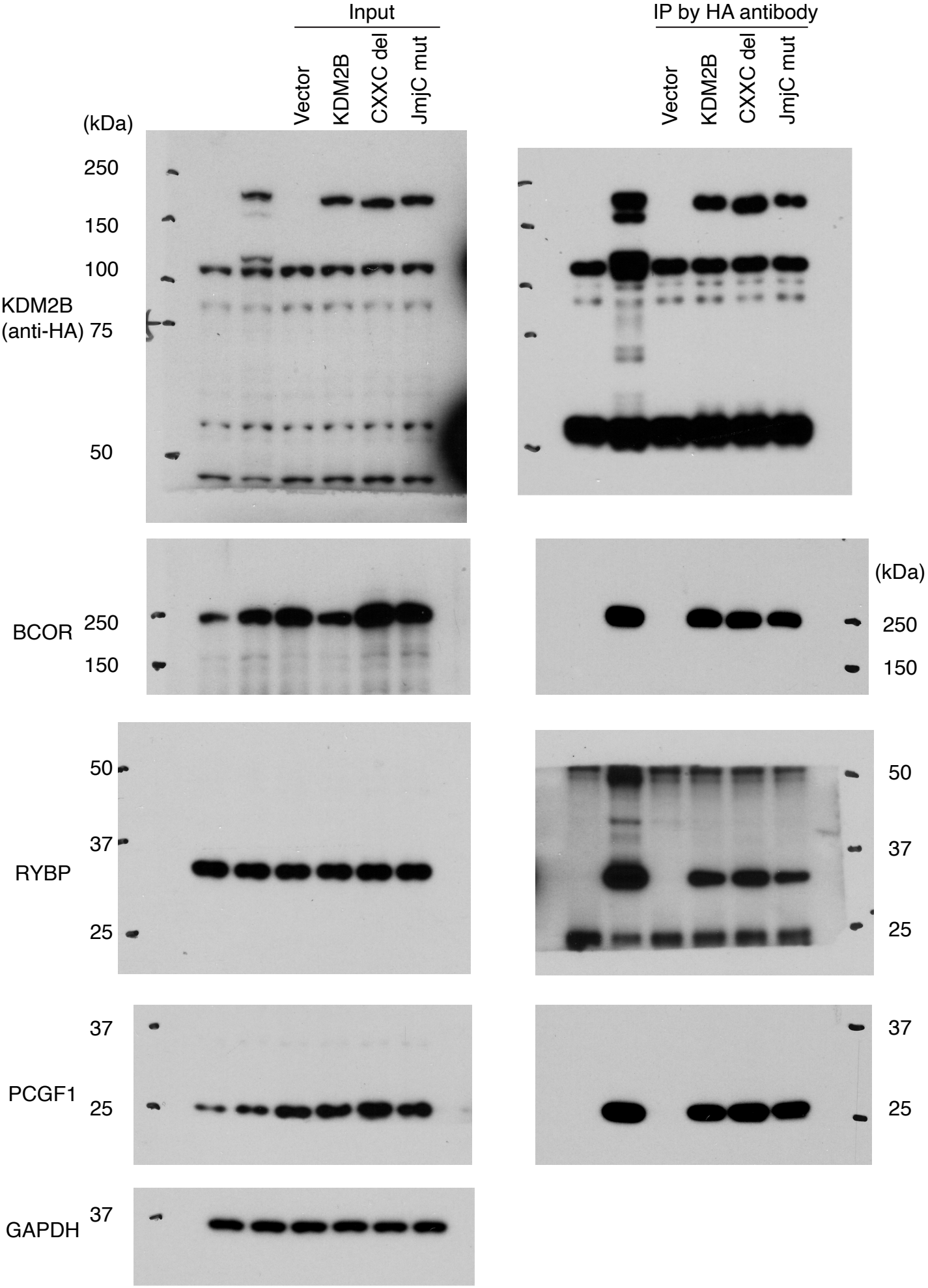
Input
IP by HA antibody
Vector
KDM2B
CXXC del
JmjC mut
(kDa)
250
150
100
75
50
KDM2B
(anti-HA)
BCOR
RYBP
PCGF1
GAPDH
37
25
