# Supplementary material for: Divergent medulloblastoma chromatin states disclose KDM2B as a selective dependency
Source: Nat Genet. 2026 Sep 24;58(10):2590–604. doi: 10.1038/s41588-026-02745-1 (PMC13649585; doi:10.1038/s41588-026-02745-1)
Supplement: Supplementary file 8 [file 41588_2026_2745_MOESM8_ESM.pdf]

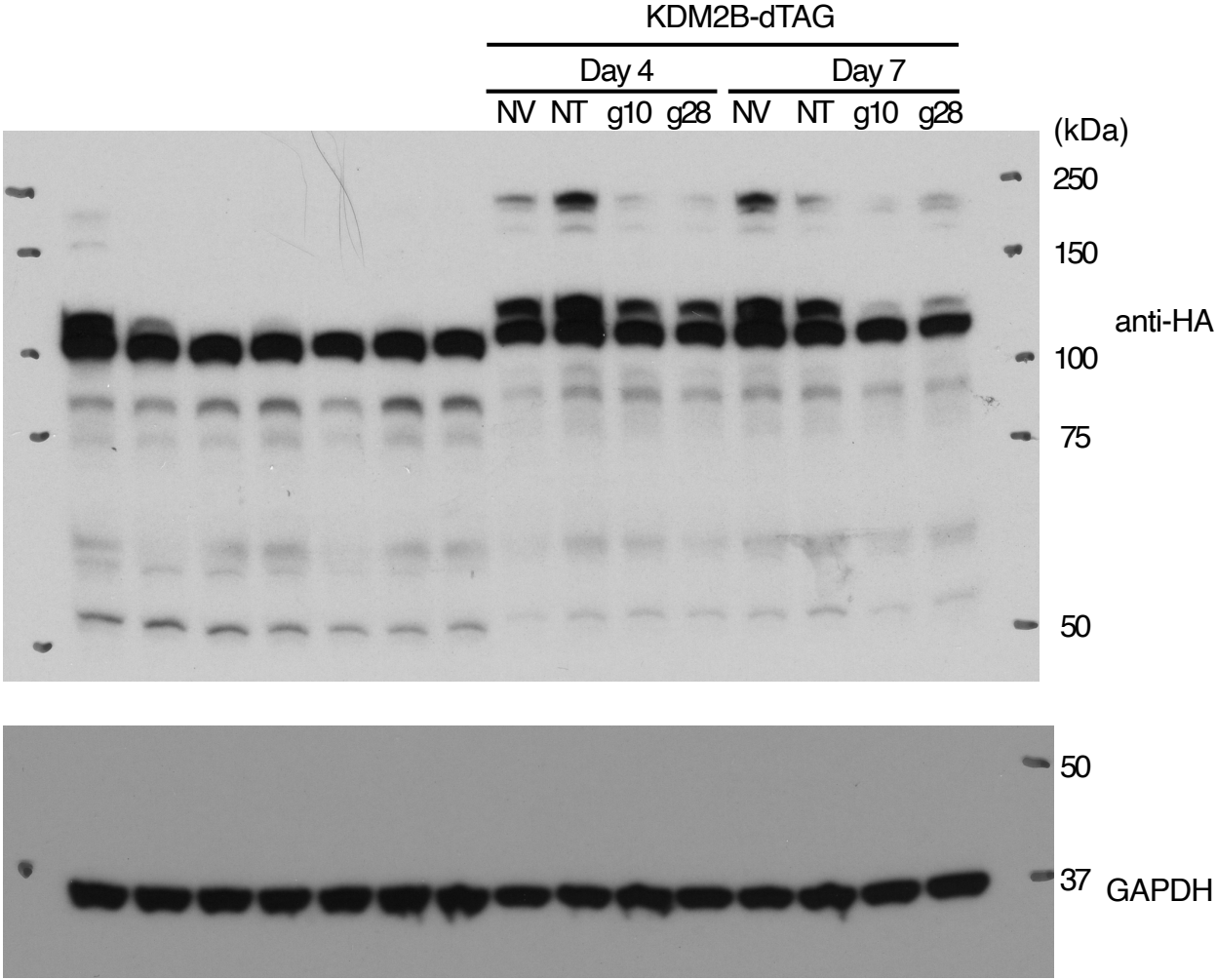

KDM2B-dTAG
Day 4
Day 7
NV NT g10 g28 NV NT g10 g28
(kDa)
250
150
100
75
50
anti-HA
50
37
GAPDH
