# Supplementary material for: Divergent medulloblastoma chromatin states disclose KDM2B as a selective dependency
Source: Nat Genet. 2026 Sep 24;58(10):2590–604. doi: 10.1038/s41588-026-02745-1 (PMC13649585; doi:10.1038/s41588-026-02745-1)
Supplement: Supplementary file 9 [file 41588_2026_2745_MOESM9_ESM.pdf]

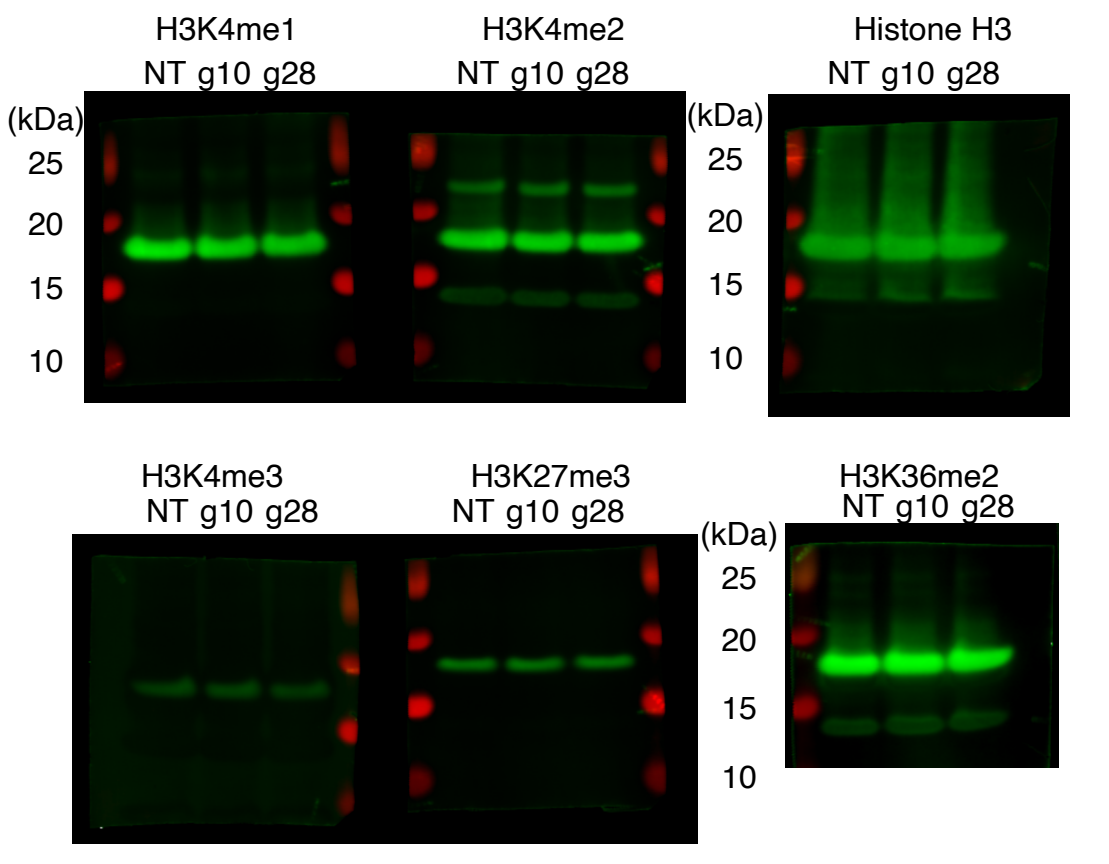
H3K4me1
NT g10 g28
H3K4me2
NT g10 g28
Histone H3
NT g10 g28
(kDa)
25
20
15
10
(kDa)
25
20
15
10
H3K4me3
NT g10 g28
H3K27me3
NT g10 g28
H3K36me2
NT g10 g28
(kDa)
25
20
15
10
